# Enantioselective [3 + 3] Annulation–Deoxalation Strategy for Rapid Access to δ-Oxoesters via N-Heterocyclic Carbene Catalysis

**DOI:** 10.1021/acs.orglett.3c04397

**Published:** 2024-02-07

**Authors:** Izabela Barańska, Liliana Dobrzańska, Zbigniew Rafiński

**Affiliations:** Faculty of Chemistry, Nicolaus Copernicus University in Torun, 7 Gagarin Street, 87-100 Torun, Poland

## Abstract

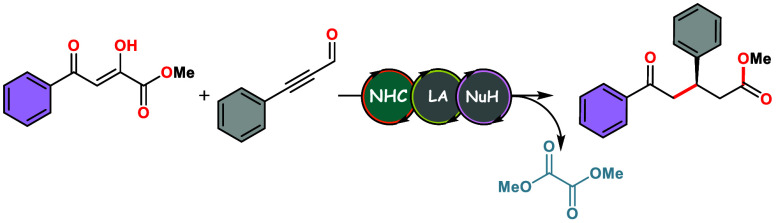

A new and unprecedented stereoselective synthetic approach to δ-oxoesters derivatives from readily available starting materials has been developed. This method, catalyzed by N-heterocyclic carbene, involves an annulation–deoxalation reaction of alkynyl aldehydes with 2,4-diketoesters and proceeds via the chiral α,β-unsaturated acylazolium intermediates. The annulation includes the *in situ* formation of dihydropyranones, which undergo ring-opening methanolysis with Lewis acid activation, followed by deoxalation to afford chiral 1,5-ketoesters in moderate to good yields.

Asymmetric synthesis of simple, chiral building blocks, which possess important functional groups for subsequent chemical and stereochemical diversification, is a crucial task in contemporary organic synthesis.^1^ Derivatives of 1,5-dicarbonyl compounds are widely applied in creating five- or six-membered ring heterocycles and polycyclic aromatic compounds due to their versatility in conversion into various complex organic systems.^2,3^ This underpins their extensive use in numerous fields, particularly in the generation of bioactive molecules. These privileged structural motifs, intriguing due to their easily transformable functional groups, are exploited in target-oriented synthesis. Traditionally, the asymmetric synthesis of 1,5-dicarbonyl systems has predominantly been focused on the Mukaiyama–Michael reaction. This strategy, involving a silyl enol ether and an α,β-unsaturated ketone or aldehyde, forms a robust method for creating chiral 1,5-dicarbonyl arrangements with high stereocontrol (Scheme 1, top).^4^ It effectively establishes carbon–carbon bonds under optimal conditions, offering high selectivity and activity. However, there is a notable lack of alternative methods beyond this reaction for asymmetric synthesis of 1,5-dicarbonyl systems, suggesting a need for further exploration and underlining the significance of this reaction.

**Scheme 1:**
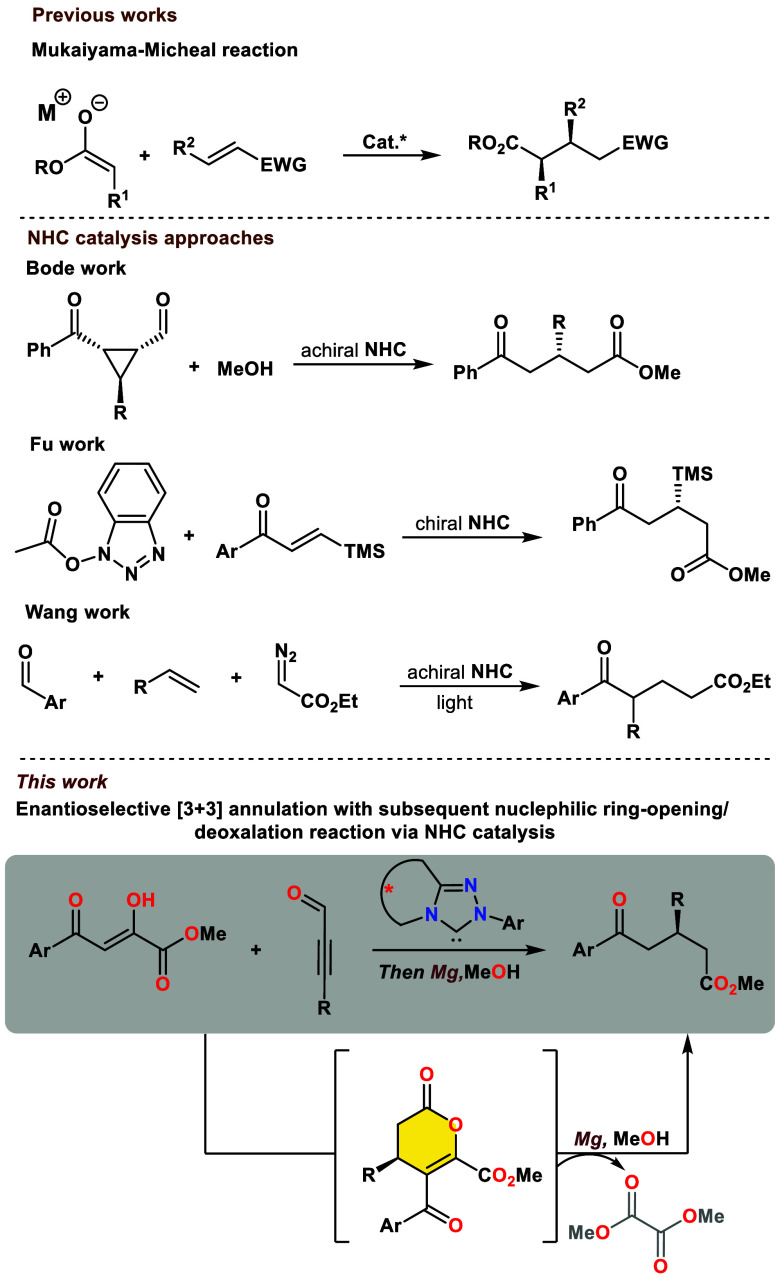
Organocatalytic Methods for the Synthesis of 1,5-Diketoesters: Previous Works and Our Strategy

In recent years, catalysis using N-heterocyclic carbenes (NHC) has shown remarkable utility in various transformations, activating a wide range of molecules and providing access to diverse carbocycles, heterocycles, and acyclic molecules with impressive enantioselectivity. Among these reactions, generating α,β-unsaturated acylazoliums from α,β-unsaturated aldehydes/acid derivatives is an important nonumpolung pathway.^5^ Given these advantages, NHC catalysis holds broad application prospects. However, its application in the synthesis of 1,5-ketoesters has been limited.^6^ In 2006, Bode and co-workers described the first NHC-catalyzed C–C bond-cleavage reaction, useful for synthesizing enantiomerically enriched esters from chiral formylcyclopropanes.^7^ This two-step process for obtaining enantioenriched 1,5-ketoesters is notable for its simplicity and mild conditions, but it necessitates the use of optically pure substrates (Scheme 1). More recently, Fu achieved efficient and elegant carbene-catalyzed formal [4 + 2] annulation to construct δ-keto-β-silyl carboxylic esters and amides using acetic esters and silyl enones, followed by nucleophilic ring-opening.^8^ Soon after, Wang and co-workers reported a three-component bisfunctionalization of unactivated olefins using the versatile diazo ester synthon in combination with NHC and photoredox catalysis (Scheme 1).^9^ Our study proposes an alternative approach, exploring the potential of organocatalysis in asymmetrically forming 1,5-diketoesters. This new method aims to diversify strategies for synthesizing 1,5-dicarbonyl compounds. Despite the success of the Mukaiyama–Michael reaction, the need for more varied methods in this field suggests opportunities for further research and development. In this context, we envisioned that an appropriately designed reaction model would lead to a product, which, similar to dihydrofumaric acid (DHP) and its derivatives, exhibiting electrophilic behavior, would undergo transformation through a deoxalation pathway.^10^ To address these challenges, herein, we disclose an NHC-catalyzed enantioselective [3 + 3] annulation with subsequent ring-opening by using a Lewis acid/nucleophile system, where the reaction proceeds with the deoxalation process to afford the enantiomerically enriched 1,5-ketoesters under mild conditions.

At the outset of our studies, simple and readily synthetically available methyl 2,4-dioxo-4-phenylbutanoate **1a** and 3-phenylpropiolaldehyde **2a** were selected as model substrates for the envisioned organocatalytic [3 + 3] annulation realized according to NHC activation. Additionally, we decided to conduct a detailed optimization process for each stage separately in order to better understand the course of the reaction and to have better control over the stereodifferentiating stage. Key results are summarized in Table 1 (see Supporting Information for details). Initially, the reaction was performed in toluene without base with the carbene generated from the chiral triazolium salts **A** at 40 °C. This effectiveness was attributed to the chloride counterion’s ability to act as a base, facilitating the generation of the active carbene. Interestingly, under these conditions using aminoindanol derived chiral triazolium salt **A**, the formation of dihydropyranones **3** was observed in 91% yield and 95:5 enantiomeric ratio (er) (Table 1, entry 1). The addition of proton sponge, with its strong basicity, low nucleophilicity, and ability to absorb protons, favorably impacted the reaction yield while maintaining the enantiomeric excess. In contrast, the use of other bases like DiPEA led to a simultaneous decrease in both yield and stereoselectivity (Table 1, entries 2 and 3). Notably, the reaction did not work in the presence of Mg(OTf)_2_. Moreover, the examination of several solvents such as THF, dioxane, MTBE, and others, did not improve the enantioselectivity.

**Table 1:**
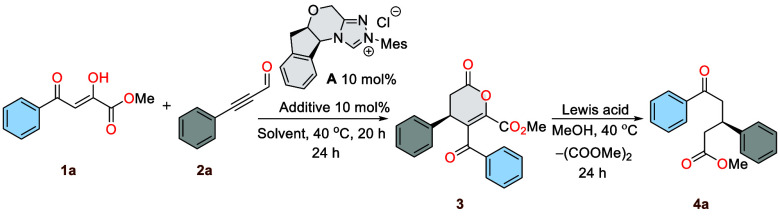
Reaction Condition Optimalization^a^

entry	NHC	solvent	additive	yield^b^ (%)	er^c^
1	A	toluene	none	91	95:5
2	A	toluene	DIPEA	75	82:18
3	A	toluene	PS	99(79)^d^	95:5
4	A	toluene	Sc(OTf)_3_	48	95:5
5	A	toluene	LiCl	83	95:5
6	A	toluene	Mg(OTf)_2_	NR	
7	A	DCM	PS	99	90:10
8	A	MTBE	PS	71	94:6
9	A	*o*-xylene	PS	81	95:5
10	A	*m*-xylene	PS	73	95:5
11	A	dioxane	PS	96	88:12
12	A	THF	PS	75	85:15
13	A	Et_2_O	PS	88	94:6
14	A	CMPE	PS	78	93:7
15	A	CF_3_C_6_H_5_	PS	85	92:8
16	A	F-C_6_H_5_	PS	79	93:7
		*II step*	*Lewis acid*		
17		MeOH	none	NR	
18		MeOH	Mg	99	95:5
19		MeOH	MgCl_2_	79	95:5
20		MeOH	Sc(OTf)_2_	82	95:5
21^e^,^f^	A	MeOH	Mg	94	95:5

a Initial conditions: **1a** (0.10 mmol), **2a** (0.15 mmol), NHC catalyst **A** (10 mol %), additive (10 mol %), 4A MS 20 mg in 1 mL of solvent.

b The ^1^H NMR yield of a crude product was determined with the aid of CH_2_Br_2_ as an internal standard.

c The HPLC analysis on a chiral stationary phase was used for determining er.

d Isolated yield.

e “one pot” procedure was performed, PS was used as the base, toluene was used as the solvent.

f Isolated yield of the product is provided. PS = proton sponge; 1,8-bis(dimethylamino)naphthalene.

Interestingly, the reaction in *ortho*- and *meta*-xylenes proceeded efficiently with the same level of stereoselectivity, albeit with a lower yield. Rapid reaction screening revealed that toluene and a proton sponge as a base was the best combination. In the second stage of optimization, we focused on opening the dihydropyranone ring using methanol as a nucleophile. Initial attempts with methanol alone did not yield the expected results, leading to a mixture of unidentifiable products. Employing magnesium as a Lewis acid in combination with methanol facilitated the synthesis of chiral δ-oxoesters through a deoxalation pathway. Furthermore, changing the magnesium source to magnesium chloride or scandium triflate resulted in a reduced yield of the product. Therefore, the reaction conditions shown in entries 3 and 18 were identified as the optimized conditions and combined into a “one pot” procedure (Table 1, entry 21). With the optimized reaction conditions in hand, the generation of α,γ-dioxoesters was then evaluated (Scheme 2). The effect of substituents on the aryl ring of methyl 2,4-dioxo-4-phenylbutanoate was initially explored.

**Scheme 2:**
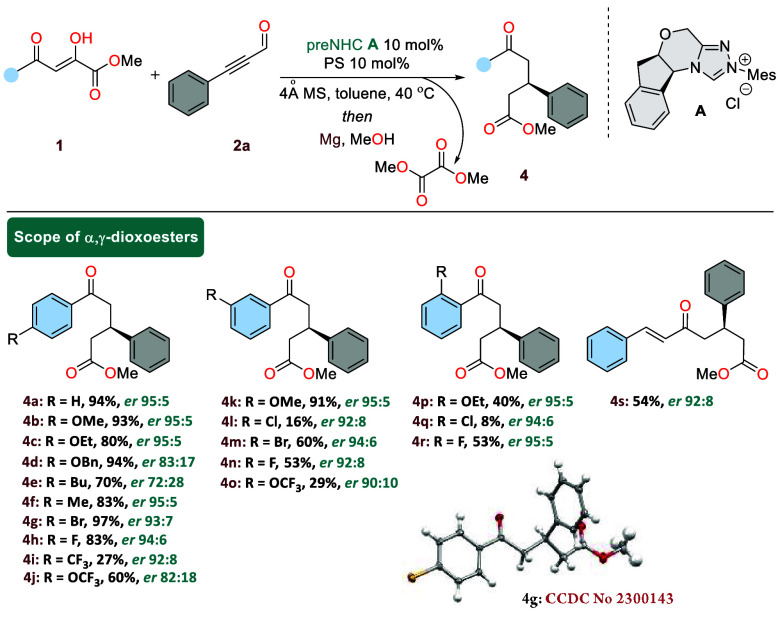
Variation of α,γ-Dioxoesters General conditions: **1** (0.2 mmol), **2a** (0.3 mmol), **A** (10 mol %), PS (10 mol %), 4 Å MS (50 mg), toluene (2.0 mL), 40 °C and 24 h followed by Mg (100 mol %) in MeOH (2.0 mL), 40 °C, 24 h.

Differently substituted methyl 2,4-dioxo-4-phenylbutanoate derivatives with both electron-withdrawing and electron-donating at the *para*-position of the aryl ring exhibited good compatibility and proceeded smoothly under the present NHC-catalyzed annulation–deoxalation to afford the corresponding optically active δ-oxoesters in good yields and with good enantiomeric ratios (**4a**–**4j**). Moreover, α,γ-dioxoesters having substituents at the 3-position of the aryl ring (**4k**–**4o**) as well as at the 2 position (**4p**–**4r**) underwent a smooth functionalization, and the desired products are formed in moderate to good yield with reasonable selectivity. Interestingly, with the a chloro moiety both in 3- and 2-positions resulted in a significant decrease in yield. Furthermore, the introduction of the more challenging styryl moiety for the **4s** was also successful under the optimized conditions, leading to the desired δ-oxoester in 54% yield with an enantiomeric ratio of 92:8. For the 4-bromo derivative **4g**, single-crystal X-ray analysis provided the final confirmation of the structure and stereochemistry, and compound **4g** has *R* configuration at the chiral carbon. To further demonstrate the generality and utility of our synthetic protocol, we next expanded our study to include a range of differently substituted ynals (Scheme 3). A variety of alkynyl aldehydes with diverse substitution patterns on the aromatic ring at the *para*-position revealed that both electron-donating and electron withdrawing groups work well, with all of the desired products obtained in good yields and high levels of optical purity (**4t**–**4z**).

**Scheme 3:**
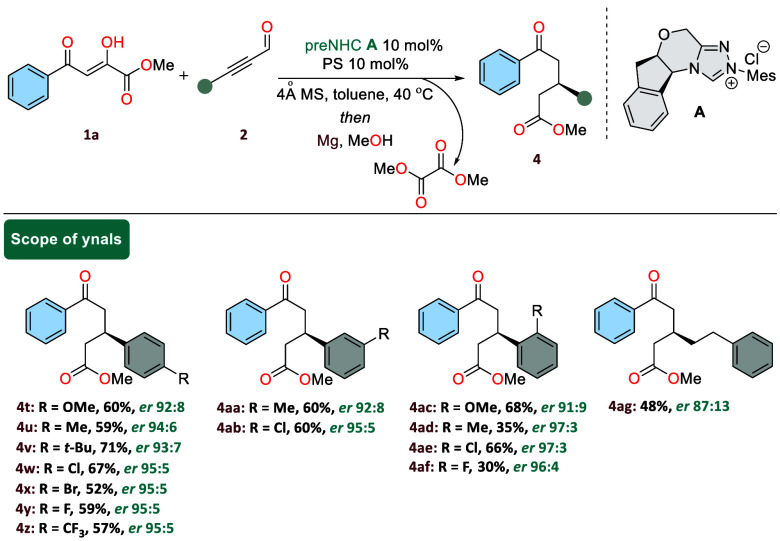
Scope of Ynals in the Reaction General conditions: **1a** (0.2 mmol), **2** (0.3 mmol), **A** (10 mol %), PS (10 mol %), 4 Å MS (50 mg), toluene (2.0 mL), 40 °C, and 24 h followed by Mg (100 mol %) in MeOH (2.0 mL), 40 °C, 24 h.

Similar trends were observed for the *meta*-substituted ynals, such as Me (**4aa**) and Cl (**4ab**), yielding the products with high selectivity and good yields.

Furthermore, substitutions at the *ortho*-position (**4ac**–**4af**) of the aromatic ring resulted in lower yields while maintaining enantioselectivity. Interestingly, for ynals substituted at both the *ortho*/*meta* positions with chloro groups (**4ae**, **4ab**), the reactions proceeded smoothly, maintaining yields comparable to those of chloro-substituted α,γ-dioxoesters. Notably, challenging aliphatic alkynyl aldehyde also proved effective under the optimized conditions, leading to the desired 1,5-ketoester in good yield, albeit with lower enantioselectivity.

A mechanistic rationalization for this unexpected annulation–deoxalation reaction is proposed, as illustrated in Scheme 4. The first stage [3 + 3] annulation process begins with the addition of carbene, generated upon deprotonation of triazolium salt **A**, to alkynyl aldehydes, resulting in α,β-unsaturated acylazolium **I** after a redox isomerization process. The direct conjugate addition of **1a** to **I**, followed by H-migration, yields adduct **III**, which then undergoes an intramolecular lactonization reaction to produce dihydropyranone **3** and regenerate the NHC catalyst. The following stage of this transformation involves the ring-opening of dihydropyranones **3** to **IV** using a magnesium–methanol system. It is hypothesized that the resultant magnesium ions function as weak Lewis acids **V**, activating the carbonyl group and facilitating the addition of a methoxide ion. The tetrahedral intermediate **VI** thus formed rearrangements to produce δ-ketoester **4a** and dimethyl oxalate.

**Scheme 4:**
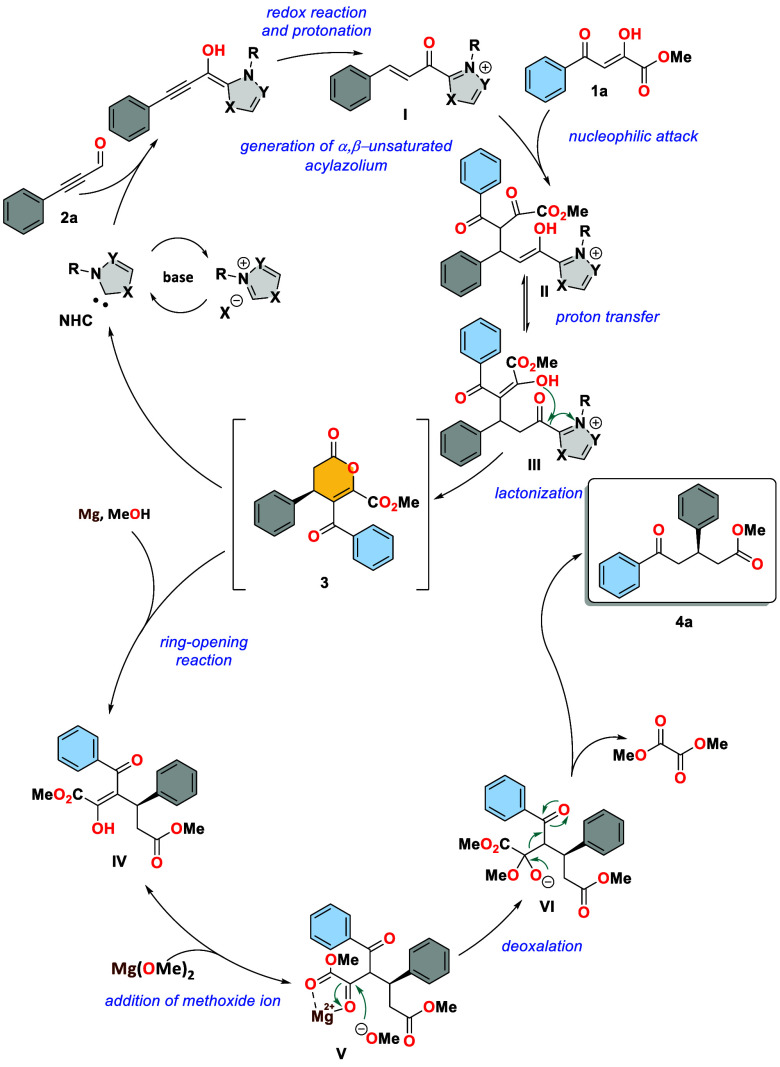
Plausible Mechanism for δ-Oxoester Formation

To demonstrate the practicality of the current protocol (Scheme 5), we increased the reaction scale 7-fold. To our delight, the reaction conducted on 1.35 mmol efficiently yielded the chiral δ-oxoester **4a** in 77% yield with 95:5 er.

**Scheme 5:**
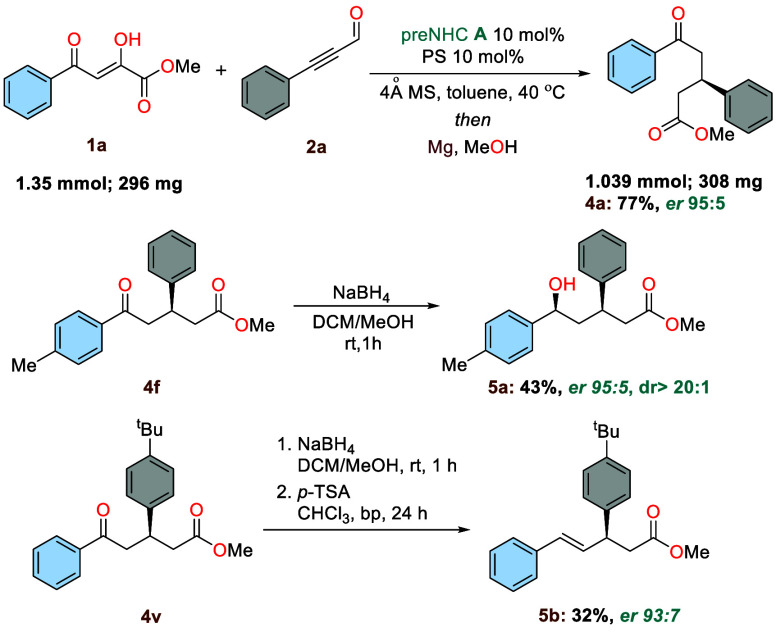
Scale-up Experiment and Functionalization of δ-Oxoesters

We also carried out functionalization of the synthesized chiral ketoesters. Reduction of **4f** using NaBH_4_ led to the formation of alcohol **5a** with high enantio- and diastereoselectivity (dr > 20:1, 95:5 er) and moderate yield. Furthermore, a two-step procedure involving NaBH_4_ reduction followed by dehydration using *p*-toluenesulfonic acid monohydrate led to the formation of the double bond in compound **5b** in 32% yield and 93:7 er.

In conclusion, we have developed a highly efficient NHC-catalyzed [3 + 3] annulation–deoxalation strategy of α,γ-dioxoesters with alkynyl aldehyde derivatives to prepare chiral 1,5-ketoesters with good yields and good to high enantiomeric ratios. This unique approach is particularly attractive due to its easy access to substrates and a transition-metal-free protocol which enhances the potential utilization value of the final products as simple building blocks. This study advances the development of a powerful strategy that combines nonumpolung catalysis of NHC and the deoxalation process through Lewis acid activation. Key features of this methodology include mild reaction conditions, a wide substrate scope, and novel application in the synthesis of chiral 1,5-ketoesters.

## Data Availability

The data underlying this study are available in the published article and its online Supporting Information.

## Supporting Information Available

Details on experimental procedures, characterization data, NMR spectra, and HPLC traces of all the δ-oxoesters (PDF)
