# Supplementary material for: Enantioselective [3 + 3] Annulation–Deoxalation Strategy for Rapid Access to δ-Oxoesters via N-Heterocyclic Carbene Catalysis
Source: Org Lett. 2024 Feb 7;26(6):1207–11. doi: 10.1021/acs.orglett.3c04397 (PMC10877609; doi:10.1021/acs.orglett.3c04397)
Supplement: Supplementary file 1 — ol3c04397_si_001.pdf [file ol3c04397_si_001.pdf]

Supporting Information

Enantioselective [3+3] Annulation-Deoxalation Strategy for Rapid Access to δ-Oxoesters via N-heterocyclic Carbene Catalysis

Izabela Barańska, Liliana Dobrzańska, Zbigniew Rafiński*

Faculty of Chemistry,
Nicolaus Copernicus University in Torun
7 Gagarin Street, 87-100 Torun, Poland
*Corespondence: payudo@umk.pl

List of contents

1. General Methods……………………………………………………………..S2
2. Reaction conditions optimization……………………………………………….S3
3. Synthetic Procedures……………………………………………………………S5
4. X-Ray Crystallography Data…………………………………………………..S31
5. NMR Spectra…………………………………………………………………..S33
6. HPLC Chromatograms………………………………………………………...S89
7. References……………………………………………………………………S124

1. General Methods

Presented reactions were carried out in dry glassware under an inert atmosphere of argon. Selected reactions were monitored by using thin-layer chromatography (TLC), which was visualized under a UV lamp (254 nm). Anhydrous solvents were prepared using an INERT PureSolv Solvent Purification System. Purification of the selected products was performed by column chromatography using a CombiFlash Rf+ Lumen system with UV-VIS and ELSD detectors. RediSepR$_f$ GOLD columns were used. NMR spectra were recorded on Bruker AMX 400 [400 MHz (^{1}H)] and Bruker AMX 700 [700 MHz (^{1}H)] spectrometers, using $CDCl_3$ as a solvent, and were reported in ppm relative to $CHCl_3$ residual peak (δ 7.24) for ^{1}H NMR and relative to the central $CDCl_3$ (δ 77.23) resonance for ^{13}C NMR. Coupling constants (*J*) are given in Hz. Structural assignments for **5a** compound were made with additional information from NOESY experiment (S85). Infrared spectra were recorded on an Alpha FT-IR spectrometer from Bruker with an ATR module. Mass spectra were recorded on a Synapt G2-S mass spectrometer (Waters) equipped with the electrospray (ESI) ion source and quadrupole-Time-of-flight (qTOF) mass analyzer. Melting points of the obtained products were measured on a Stuart SMP50 Melting Point automatic Apparatus. The enantiomeric excess of chiral products was determined using HPLC Agilent Technologies 1200 Series and chiral stationary phases: Phenomenex Lux Cellulose-1 (3 μm) and Phenomenex Lux Amylose-1 (3 μm). The diffraction data of the studied compound were collected on an XtaLAB Synergy-S Dualflex diffractometer equipped with monochromated CuKα radiation (λ = 1.54184 Å).

2. Reaction conditions optimization- screening of temperature and catalysts[a]

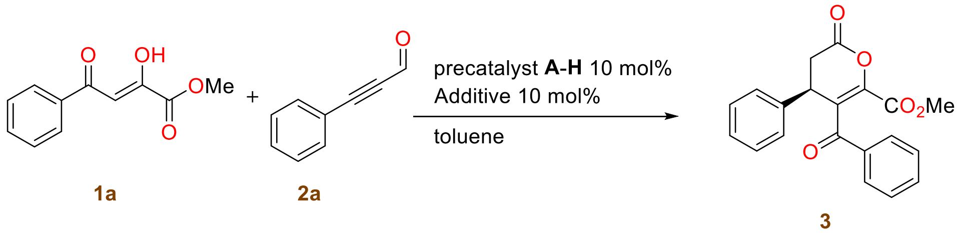

entry	temp. [°C]	preNHC	additive	yield[b] (%)	er[c] (%)
1	rt	A	-	-	-
2	50	A	-	79	94:6
3	40	A	-	91	95:5
4	40	B	-	12	50:50
5	40	C	-	75	63:37
6	40	D	-	32	64:36
7	40	E	-	32	64:36
8	40	F	-	57	76:24
9	40	G	-	45	76:24
10	40	H	-	48	82:18
**11**	**40**	**A**	**PS 10 mol%**	**99**	**95:5**

[a]Initial rection conditions: **1a** (0.10 mmol), **2a** (0.15 mmol), NHC catalyst **A-H** (10 mol%), 4Å MS 20 mg in 1 mL of toluene. [b]The ^1H NMR yield of crude product was determined with the aid of CH_2Br_2 as an internal standard. [c]The HPLC analysis on a chiral stationary phase was used for determining er.

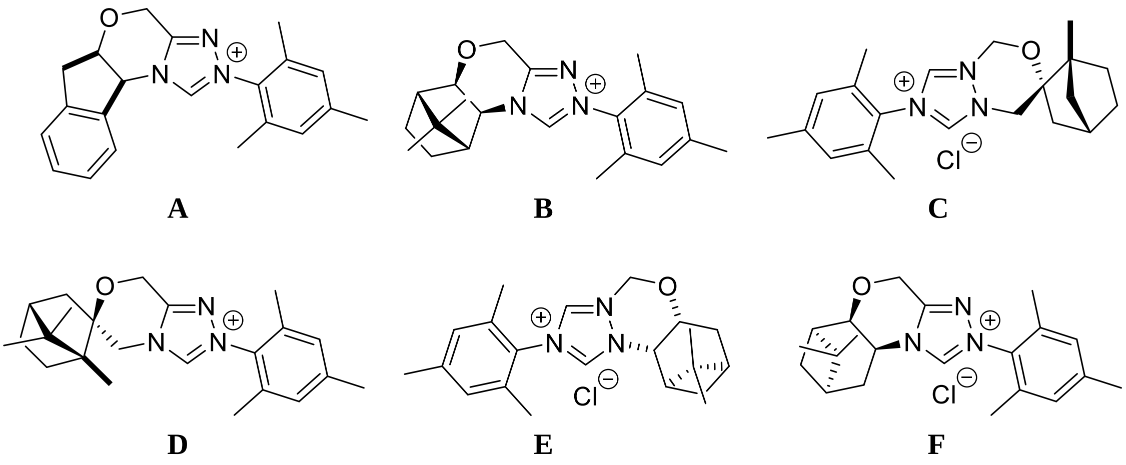

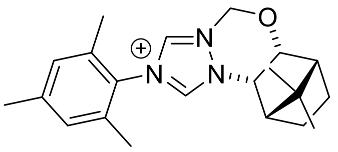

G

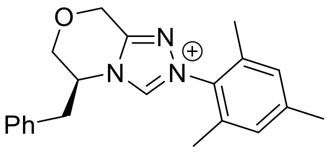

H

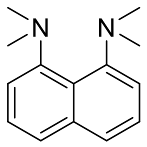

PS

(**PS** = Proton Sponge = 1,8-Bis(dimethylamino)naphthalene)

The remaining optimization steps are included in the main text of the manuscript.

3. Synthetic procedures

The synthesis and isolation of 3:

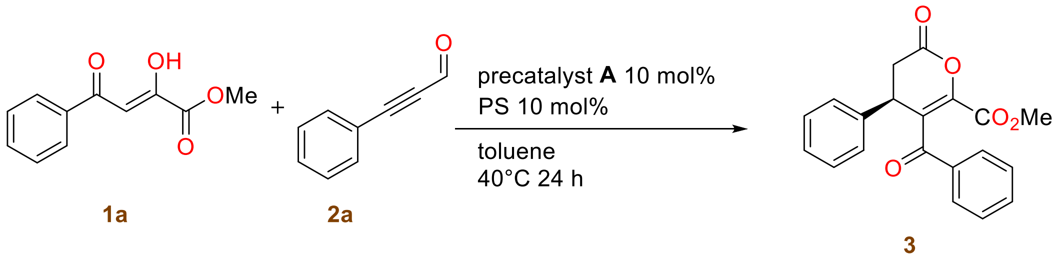

A round bottom flask was charged with triazolium salt **A** (7.76 mg; 0.02 mmol; 0.1 eq), proton sponge (4.29 mg; 0.02 mmol; 0.1 eq), and toluene (2 mL). Then the solution was allowed to stir at ambient temperature for 10 min under an inert argon atmosphere. The substrate **1** (41.24 mg; 0.2 mmol; 1 eq) and aldehyde **2a**[1] (39 mg; 0.3 mmol; 1.5 eq) were added, following the addition of molecular sieves (4Å) (40 mg). The stirring was continued at 40 °C for 24 h. After this time, toluene was evaporated, and the residue was purified by flash column chromatography (AcOEt/hex gradient 0% → 20 % AcOEt) to give **3** as a brown oil (53 mg; 0.16 mmol; 79% yield).

The ring-opening reaction of 3:

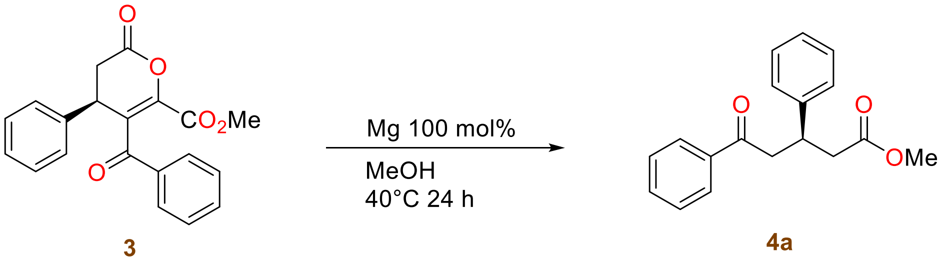

A round bottom flask was charged with **3** (53 mg; 0.16 mmol; 1.0 eq), magnesium (3.89 mg; 0.16 mmol; 1.0 eq), and methanol (1.6 mL). Then the solution was allowed to stir at 40 °C for 24 h under an inert argon atmosphere. After this time, methanol was evaporated, and the residue was dissolved in Et_2O and filtered through a PTFE 0.45 μm filter. After evaporation of solvent product **4a** was obtained as a yellow solid (42.35 mg; 0.15 mmol; 94% yield).

The synthesis of final products 4 were performed according to General „one-pot" Procedure 3.

General Procedures:

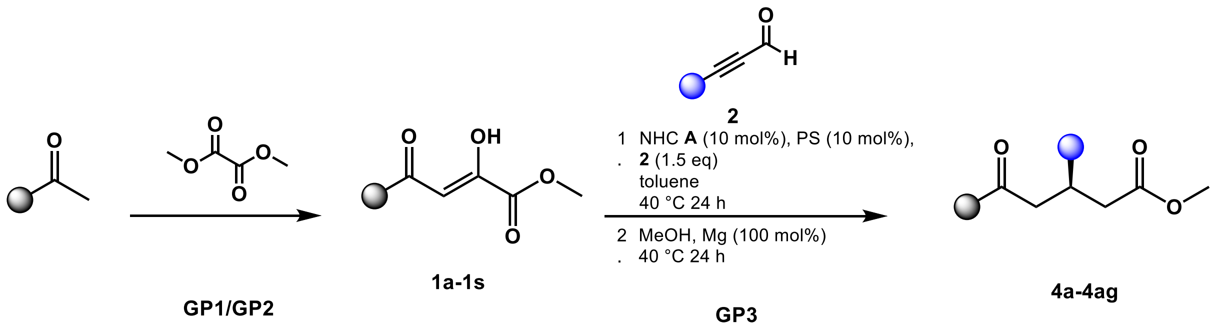

GP1. General Procedure 1:

Sodium (12 mmol; 1.21 eq) was slowly added to the dry methanol (1.7 M) at 0°C. After the sodium was dissolved, the dimethyl oxalate (10 mmol; 1.0 eq) and solution of acetophenone (10 mmol; 1.0 eq) in Et_2O (0.83 M) were added. The reaction was mixed at room temperature for 24 h and concentrated under vacuum. The residue was dissolved in Et_2O and water. The water phase was separated and placed in an ice bath, and 1M HCl was added to the pH=2. The precipitate was filtered off and washed with water. The final pure product **1** was obtained without further purification.

GP2. General Procedure 2:

The potassium *tert*-butoxide 1M in THF (13 mmol; 1.3 eq) was introduced to the two-necked flask equipped with a dropping funnel. The mixture was diluted with THF (0.27 M) and cooled to the 0°C. The solution of acetophenone (10 mmol; 1.0 eq) in THF (1.33 M) was dropped under the argon atmosphere. The reaction was carried out for 45 minutes at the same temperature. Dimethyl oxalate (12 mmol; 1.2 eq) was added portionwise. The reaction was carried out for 24 h at room temperature. The precipitate was filtered off, washed with Et_2O, and dried under the vacuum. Obtained salt was dissolved in water, and the 1M HCl was added dropwise to the pH=2. The solid was filtered off, washed with water, and dried under vacuum. The final pure product **1** was obtained without further purification.

GP3. General "one-pot" Procedure 3:

A round bottom flask was charged with triazolium salt **A** (0.02 mmol; 0.1 eq), proton sponge (0.02 mmol; 0.1 eq), and toluene (0.1 M). Then the solution was allowed to stir at ambient temperature for 10 min under an inert argon atmosphere. The substrate **1** (0.2 mmol; 1 eq) and aldehyde **2**[1] (0.3 mmol; 1.5 eq) were added, following the addition of molecular sieves (4Å) (40 mg). The stirring was continued at 40 °C for 24 h. After this time, toluene was evaporated, the residue was dissolved in methanol (0.1 M), and magnesium (0.2 mmol; 1.0 eq) was added. The reaction was carried out for another 24 h at 40°C. The solvent was evaporated and the crude was purified by flash column chromatography (if necessary) to give **4** as a pure product.

***The synthesis of racemic products 4** was performed with the use of triazolium salt **A** and ent-**A** in 1:1 molar ratio (0.05 eq : 0.05 eq).*

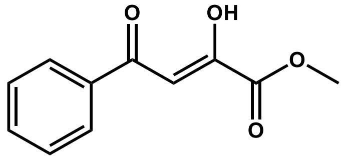

methyl (Z)-2-hydroxy-4-oxo-4-phenylbut-2-enoate (**1a**). GP1. A scale of 30 mmol, white solid, 3.32 g (16.1 mmol), isolated yield of 54%; ^{1}H NMR (400 MHz, $CDCl_3$) δ ppm 15.27 (s, 1H), 7.99 - 8.03 (m, 2H), 7.62 (tt, *J* = 7.3, 1.5 Hz, 1H), 7.49 - 7.55 (m, 2H) 7.10 (s, 1H), 3.95 (s, 3H); ^{13}C NMR (176 MHz, $CDCl_3$) δ ppm 190.7, 169.5, 162.6, 134.9, 133.8, 128.9, 127.9, 98.0, 53.1. IR ν_{max}: 3119, 3070, 2965, 1724, 1597, 1566, 1442, 1252, 1179, 1127, 937, 888, 841, 777, 702, 680, 629 cm^{-1}. mp 59.5 - 63.0 °C. The above analysis results correspond to the literature data.[2]

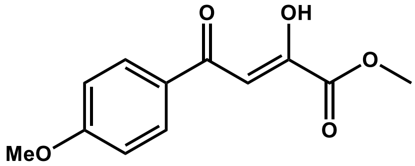

methyl (Z)-2-hydroxy-4-(4-methoxyphenyl)-4-oxobut-2-enoate (**1b**). GP1. A scale of 10 mmol, white solid, 1.78 g (7.5 mmol), isolated yield of 75%; ^{1}H NMR (700 MHz, $CDCl_3$) δ ppm 15.47 (s, 1H), 8.00 (d, *J* = 8.8 Hz, 2H), 7.05 (s, 1H), 7.00 (d, *J* = 8.8 Hz, 2H), 3.95 (s, 3H), 3.91 (s, 3H). ^{13}C NMR 190.4, 167.6, 164.4, 162.9, 130.3, 127.6, 114.2, 97.9, 55.6, 53.1 IR ν_{max}: 2969, 2851, 1753, 1594, 1429, 1247, 1177, 1131, 1116, 1056, 1016, 966, 925, 845, 819, 775, 609, 497 cm^{-1}. mp 96.1 - 97.8°C. The above analysis results correspond to the literature data.[3]

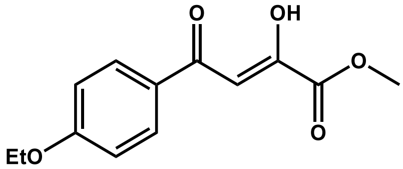

methyl (Z)-4-(4-ethoxyphenyl)-2-hydroxy-4-oxobut-2-enoate (**1c**). GP1. A scale of 10 mmol, yellow solid, 1.75 g (7.0 mmol), isolated yield of 70%; ^{1}H NMR (700 MHz, $CDCl_3$) δ ppm 8.00 - 7.99 (m, 2H), 7.05 (s, 1H), 6.99 - 6.97 (m, 2H), 4.79 (s, 1H), 4.14 (q, *J* = 7.0 Hz, 2H), 3.95 (s, 3H), 1.47 (t, *J* = 6.8 Hz, 3H). ^{13}C NMR (75.5 MHz, $CDCl_3$) δ 190.41, 167.56, 163.86, 162.97, 130.37, 127.44, 114.66, 97.90, 63.96, 53.14, 14.64. IR ν_{max}: 3115, 3050, 2988, 2940, 2886, 1722, 1598, 1443, 1390, 1249, 1176, 1113, 1041, 974, 925, 834, 806, 782, 618, 524, 503 cm^{-1}. mp 90.2 - 91.4 °C. The above analysis results correspond to the literature data.[2]

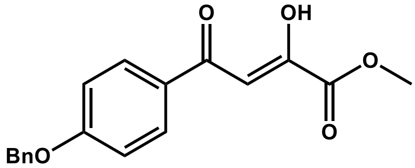

methyl (Z)-4-(4-benzyloxy)phenyl)-2-hydroxy-4-oxobut-2-enoate (**1d**). GP1. A scale of 10 mmol, white solid, 2.25 g (7.2 mmol), isolated yield of 72%; ^{1}H NMR (700 MHz, $CDCl_3$) δ ppm 15.45 (s, 1H), 8.02 (d, *J* = 9.0 Hz, 2H), 7.46 - 7.42 (m, 5H), 7.39 - 7.37 (m, 1H), 7.08 (d, *J* = 8.6 Hz, 2H), 7.06 (s, 1H), 5.18 (s, 2H), 3.96 (s, 3H). ^{13}C NMR (176.1 MHz, $CDCl_3$) δ ppm 190.2, 168.0, 163.5, 162.9, 136.0, 130.3, 128.7, 128.3, 128.0, 127.4, 115.1, 97.8, 70.3, 53.0. IR ν_{max}: 3032, 2964, 1740, 1593, 1511, 1432, 1383,

1320, 1301, 1257, 1233, 1171, 1112, 1005, 975, 916, 864, 845, 828, 774, 753, 714, 696, 630, 571, 501 cm^{-1}. mp dec. The above analysis results correspond to the literature data.[2]

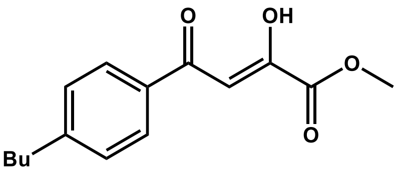

methyl (Z)-4-(4-butylphenyl)-2-hydroxy-4-oxobut-2-enoate (**1e**). GP1. A scale of 10 mmol, yellow solid, 1.70 g (6.5 mmol), isolated yield of 65%; ^{1}H NMR (700 MHz, $CDCl_3$) δ ppm 15.3 (s, 1H), 7.91 (d, *J* = 8.2 Hz, 2H), 7.30 (d, *J* = 8.2 Hz, 2H), 7.06 (s, 1H), 3.93 (s, 3H), 2.68 (t, *J* = 7.7 Hz, 2H), 1.64 - 1.60 (m, 2H), 1.36 (sxt, *J* = 7.3 Hz, 2H), 0.93 (t, *J* = 7.3 Hz, 3H). ^{13}C NMR (75.5 MHz, $CDCl_3$) δ ppm 190.8, 168.8, 162.8, 149.9, 132.4, 129.0, 128.0, 98.0, 53.1, 35.8, 33.1, 22.3, 13.8. IR v_{max}: 31173069, 3041, 2952, 2928, 2869, 2857, 1723, 1604, 1443, 1260, 1182, 1115, 965, 915, 899, 843, 781, 714, 695, 615, 508 cm^{-1}. mp 58.7 - 60.9°C. HRMS (ESI-TOF) m/z: $[M + H]^+$ calcd for $C_{15}H_{19}O_4$ 263.1283 found: 263.1279.

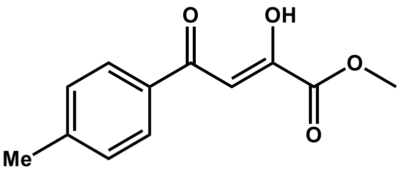

methyl (Z)-2-hydroxy-4-oxo-4-(p-tolyl)but-2-enoate (**1f**). GP1. A scale of 10 mmol, white solid, 0.89 g (4.0 mmol), isolated yield of 40%; ^{1}H NMR (700 MHz, $CDCl_3$) δ ppm 15.38 (s, 1H), 7.93 (d, *J* = 7.9 Hz, 2H), 7.33 (d, *J* = 7.9 Hz, 2H), 7.09 (s, 1H), 3.96 (s, 3H), 2.46 (s, 3H). ^{13}C NMR (75.5 MHz, $CDCl_3$) δ ppm 190.9, 168.7, 162.8, 145.1, 132.3, 129.7, 128.1, 98.0, 53.2, 21.8. IR v_{max}: 3119, 2961, 2854, 1724, 1607, 1443, 1262, 1184, 1114, 966, 914, 828, 782, 740, 703, 589, 531, 471 cm^{-1}. mp 82.3 - 84.3 °C. The above analysis results correspond to the literature data.[4]

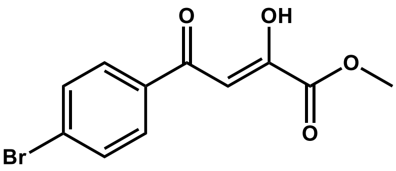

methyl (Z)-4-(4-bromophenyl)-2-hydroxy-4-oxobut-2-enoate (**1g**). GP2. A scale of 10 mmol, yellow solid, 1.41 g (4.9 mmol), isolated yield of 49%; ^{1}H NMR (700 MHz, $CDCl_3$) δ ppm 15.15 (s, 1H), 7.87-7.85 (m, 2H), 7.66-7.64 (m, 2H), 7.04 (s, 1H), 3.94 (s, 3H). ^{13}C NMR (176 MHz, $CDCl_3$) δ ppm 189.3, 169.7, 162.4, 133.6, 132.2, 129.2, 129.0, 97.8, 53.2. IR v_{max}: 3447, 3105, 2956, 1729, 1583, 1435, 1273, 1248, 1102, 1072, 1005, 966, 917, 840, 815, 767, 747, 464 cm^{-1}. mp 104.7 - 108.3 °C. The above analysis results correspond to the literature data.[4]

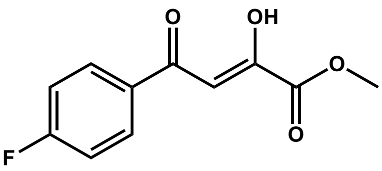

methyl (Z)-4-(4-fluorophenyl)-2-hydroxy-4-oxobut-2-enoate (**1h**). GP2. A scale of 10 mmol, yellow solid, 1.56 g (7.0 mmol), isolated yield of 70%; ^{1}H

NMR (700 MHz, $CDCl_3$) δ ppm 15.19 (s, 1H), 8.04-8.02 (m, 2H), 7.20-7.17 (m, 2H), 7.04 (s, 1H), 3.94 (s, 3H). ^{13}C NMR (176 MHz, $CDCl_3$) δ ppm 189.6, 168.9, 166.3 (d, *J* = 256.7 Hz), 162.6, 131.4 (d, *J* = 3.3 Hz), 130.6 (d, *J* = 8.2 Hz), 116.1 (d, *J* = 22.9 Hz), 97.9, 53.2. IR v_{max}: 3506, 3117, 2968, 1734, 1595, 1507, 1440, 1283, 1230, 1163, 1130, 1105, 1011, 972, 920, 848, 825, 803, 773, 589, 566, 493 cm^{-1}. mp 114.4 - 118.8 °C. The above analysis results correspond to the literature data.[4]

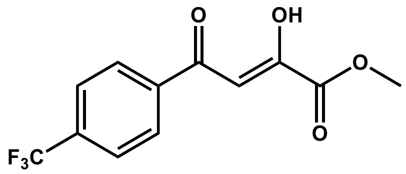

methyl (Z)-2-hydroxy-4-oxo-4-(4-trifluoromethyl)phenyl)but-2-enoate (**1i**). GP1. A scale of 10 mmol, white solid, 1.86 g (6.8 mmol), isolated yield of 68%; ^{1}H NMR (700 MHz, $CDCl_3$) δ ppm 15.10 (s, 1H), 8.12 (d, *J* = 8.2 Hz, 2H), 7.80 (d, *J* = 8.6 Hz, 2H), 7.12 (s, 1H), 3.99 (s, 3H). ^{13}C NMR (176.1 MHz, $CDCl_3$) δ ppm 188.5, 171.0, 162.2, 137.8, 134.9 (q, *J* = 33.2 Hz), 128.1, 125.8, 123.5 (q, *J* = 273.0 Hz), 98.0, 53.2. IR v_{max}: 3105, 2958, 1732, 1605, 1435, 1316, 1257, 1163, 1104, 1066, 1013, 970, 918, 833, 780, 724, 696, 509, 420 cm^{-1}. mp 104.0 - 106.5°C. The above analysis results correspond to the literature data.[5]

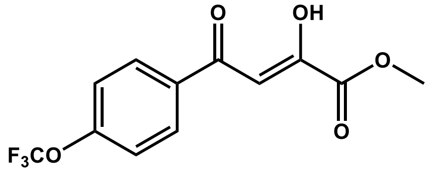

methyl (Z)-2-hydroxy-4-oxo-4-(4-(trifluoromethyl)phenyl)but-2-enoate (**1j**). GP1. A scale of 10 mmol, white solid, 1.42 g (4.9 mmol), isolated yield of 49%; ^{1}H NMR (700 MHz, $CDCl_3$) δ ppm 15.16 (s, 1H), 8.09 - 8.07 (m, 2H), 7.36 (d, *J* = 8.2 Hz, 2H), 7.08 (s, 1H), 3.98 (s, 3H). ^{13}C NMR (176 MHz, $CDCl_3$) δ ppm 189.1, 169.7, 162.4, 153.1, 133.2, 129.9, 120.6, 120.3 (q, *J* = 259.4 Hz), 97.9, 53.1. IR v_{max}: 3138, 2959, 1732, 1596, 1507, 1442, 1305, 1243, 1197, 1153, 1113, 973, 919, 831, 779, 697, 501 cm^{-1}. mp 76.5 - 77.8°C. The above analysis results correspond to the literature data.[3]

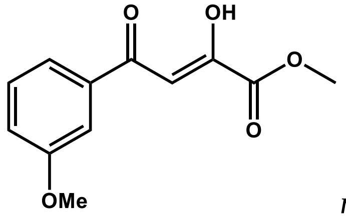

methyl (Z)-2-hydroxy-4-(3-methoxyphenyl)-4-oxobut-2-enoate (**1k**). GP2. A scale of 10 mmol, yellow solid, 1.52 g (6.4 mmol), isolated yield of 64%; ^{1}H NMR (700 MHz, $CDCl_3$) δ ppm 15.22 (s, 1H), 7.57-7.56 (m, 1H), 7.51 (dd, *J* = 2.6, 1.7 Hz, 1H), 7.41 (t, *J* = 8.0 Hz, 1H), 7.15 (ddd, *J* = 8.2, 2.6, 0.9 Hz, 1H), 7.06 (s, 1H), 3.94 (s, 3H), 3.87 (s, 3H). ^{13}C NMR (176 MHz, $CDCl_3$) δ ppm 190.7, 168.9, 162.6, 160.0, 136.3, 129.9, 120.4, 120.2, 112.3, 98.3, 55.4, 53.1. IR v_{max}: 3080, 2988, 2958, 2846, 1733, 1575, 1490, 1455, 1433, 1268, 1211, 1183, 1121, 1077, 1029, 994, 974, 878, 770, 709, 673, 623, 564 cm^{-1}. mp 85.8 - 87.8 °C. The above analysis results correspond to the literature data.[6]

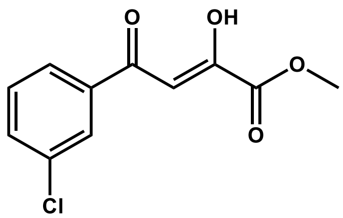

v*methyl (Z)-4-(3-chlorophenyl)-2-hydroxy-4-oxobut-2-enoate* (**1l**). GP2. A scale of 10 mmol, white solid, 2.70 g (7.6 mmol), isolated yield of 76%; ^{1}H NMR (700 MHz, $CDCl_3$) δ ppm 15.09 (s, 1H), 7.99 (t, *J* = 1.8 Hz, 1H), 7.90-7.89 (m, 1H), 7.60 (ddd, *J* = 7.9, 2.2, 0.9 Hz, 1H), 7.48 (t, *J* = 7.9 Hz, 1H), 7.07 (s, 1H), 3.98 (s, 3H). ^{13}C NMR (176 MHz, $CDCl_3$) δ ppm 189.1, 170.0, 162.4, 136.6, 135.3, 133.6, 130.2, 127.9, 125.9, 98.0, 53.2. IR v_{max}: 3530, 3070, 2972, 1731, 1719, 1602, 1562 , 1429, 1270, 1227, 1124, 1096, 1076, 979, 969, 898, 851, 800, 774, 682, 668, 639, 625 cm^{-1}. mp 78.9 - 79.6 °C. The above analysis results correspond to the literature data.[4]

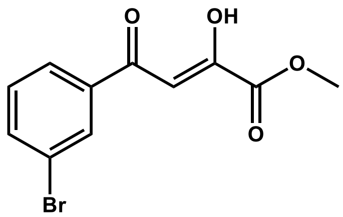

methyl (Z)-4-(3-bromophenyl)-2-hydroxy-4-oxobut-2-enoate (**1m**). GP2. A scale of 10 mmol, white solid, 2.44 g (8.6 mmol), isolated yield of 86%; ^{1}H NMR (700 MHz, $CDCl_3$) δ ppm 15.05 (s, 1H), 8.12 (d, *J* = 1.3 Hz, 1H), 7.91 (dd, *J* = 7.9, 0.9 Hz, 1H), 7.73 (dd, *J* = 7.7, 0.9 Hz, 1H), 7.39 (t, *J* = 8.0 Hz, 1H), 7.03 (d, *J* = 0.9 Hz, 1H), 3.95 (s, 3H). ^{13}C NMR (176 MHz, $CDCl_3$) δ ppm 189.0, 169.9, 162.4, 136.8, 136.5, 130.8, 130.4, 126.4, 123.2, 98.0, 53.2. IR v_{max}: 3506, 3065, 2964, 1731, 1718, 1603, 1558, 1432, 1275, 1228, 1125, 1067, 970, 872, 762, 667 cm^{-1}. mp 95.0 - 98.3 °C. The above analysis results correspond to the literature data.[4]

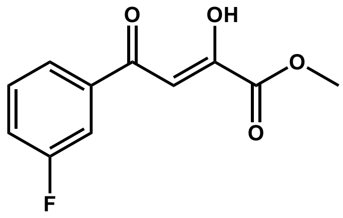

methyl (Z)-4-(3-fluorophenyl)-2-hydroxy-4-oxobut-2-enoate (**1n**). GP1. A scale of 10 mmol, white solid, 1.62 g (7.2 mmol), isolated yield of 71%; ^{1}H NMR (700 MHz, $CDCl_3$) δ ppm 15.08 (s, 1H), 7.78 (ddd, *J* = 7.7, 1.7, 1.0 Hz, 1H), 7.68 (ddd, *J* = 9.5, 2.6, 1.7 Hz, 1H), 7.49 (td, *J* = 8.0, 5.6 Hz, 1H), 7.31 (tdd, *J* = 8.2, 8.2, 2.6, 1.0 Hz, 1H), 7.04 (s, 1H), 3.95 (s, 3H). ^{13}C NMR (176 MHz, $CDCl_3$) δ ppm 189.2, 169.9, 163.0 (d, *J* = 246.9 Hz), 162.4, 137.1 (d, *J* = 6.5 Hz), 130.5 (d, *J* = 8.2 Hz), 123.6 (d, *J* = 3.3 Hz), 120.7 (d, *J* = 21.3 Hz), 114.6 (d, *J* = 22.9 Hz), 98.1, 53.2. IR v_{max}: 3472, 3083, 2967, 1745, 1574, 1427, 1252, 1183, 1129, 971, 934, 892, 823, 801, 717, 667, 646, 508, 427 cm^{-1}. mp 78.1 - 80.1 °C. The above analysis results correspond to the literature data.[4]

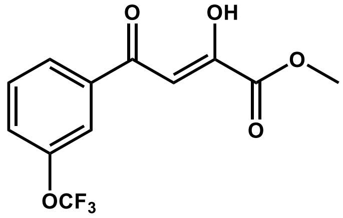

methyl (Z)-2-hydroxy-4-oxo-4-(3-trifluoromethoxy)phenyl)but-2-enoate (**1o**). GP1. A scale of 10 mmol, yellow solid, 1.13 g (3.9 mmol), isolated yield of 39%; ^{1}H NMR (700 MHz, $CDCl_3$) δ ppm 15.08 (s, 1H), 7.94 (dt, *J* = 7.7, 1.3 Hz, 1H), 7.85 (s, 1H), 7.58 (t, *J* = 8.0 Hz, 1H), 7.49 - 7.48 (m, 1H), 7.07 (s, 1H), 3.98 (s, 3H). ^{13}C NMR (176.1 MHz, $CDCl_3$) δ ppm 188.8, 170.0, 162.3, 149.7, 136.9, 130.4, 126.1, 125.9, 120.4 (q, *J* = 258.3 Hz), 120.2, 98.0, 53.2. IR ν_{max}: 3466, 3086, 2966, 1740, 1621, 1577, 1419, 1238, 1206, 1150, 1121, 948, 829, 739, 691, 632 cm^{-1}. mp 36.9 - 38.9°C. The above analysis results correspond to the literature data.[3]

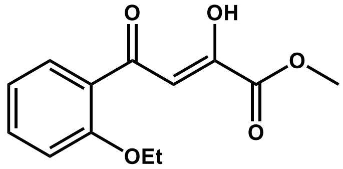

methyl (Z)-4-(2-ethoxyphenyl)-2-hydroxy-4-oxobut-2-enoate (**1p**). GP1. A scale of 10 mmol, yellow solid, 1.46 g (5.8 mmol), isolated yield of 58%; ^{1}H NMR (700 MHz, $CDCl_3$) δ ppm 15.19 (s, 1H), 7.92 (dd, *J* = 7.7, 1.7 Hz, 1H), 7.49 (ddd, *J* = 8.6, 7.1, 1.9 Hz, 1H), 7.47 (s, 1H), 7.04 (t, *J* = 8.2 Hz, 1H), 6.97 (d, *J* = 8.6 Hz, 1H), 4.17 (q, *J* = 6.9 Hz, 2H), 3.92 (s, 3H), 1.52 (t, *J* = 7.1 Hz, 3H). ^{13}C NMR (176 MHz, $CDCl_3$) δ ppm 190.2, 168.4, 163.0, 158.8, 134.6, 130.7, 124.5, 120.7, 112.8, 103.6, 64.5, 52.9, 14.5. IR ν_{max}: 3176, 2988, 2955, 2901, 1726, 1596, 1577, 1487, 1450, 1432, 1239, 1160, 1119, 1104, 1027, 926, 836, 792, 770, 752, 627, 605, 547, 530 cm^{-1}. mp 77.0 - 79.9 °C. The above analysis results correspond to the literature data.[7]

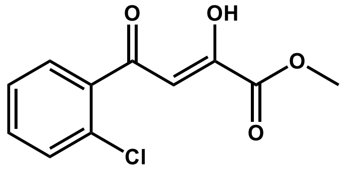

methyl (Z)-4-(2-chlorophenyl)-2-hydroxy-4-oxobut-2-enoate (**1q**). GP1. A scale of 10 mmol, white solid, 1.63 g (6.8 mmol), isolated yield of 68%; ^{1}H NMR (700 MHz, $CDCl_3$) δ ppm 14.60 (s, 1H), 7.64 (dd, *J* = 7.7, 1.7 Hz, 1H), 7.47 (dd, *J* = 8.2 Hz, 1.5 Hz, 1H), 7.45 (ddd, *J* = 8.2, 7.3, 1.7 Hz, 1H), 7.39-7.36 (m, 1H), 6.97 (s, 1H), 3.93 (s, 3H). ^{13}C NMR (75.5 MHz, $CDCl_3$) δ ppm 193.0, 167.3, 162.3, 135.7, 132.9, 132.1, 131.0, 130.3, 127.2, 103.2, 53.3. IR ν_{max}: 3099, 3058, 2964, 1725, 1590, 1431, 1269, 1118, 1042, 968, 918, 838, 784, 765, 754, 733, 679, 622, 546, 509, 473 cm^{-1}. mp 78.9 - 80.9 °C. The above analysis results correspond to the literature data.[4]

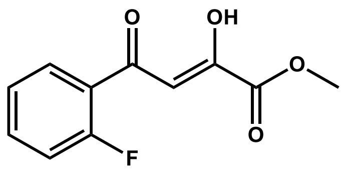

methyl (Z)-4-(2-fluorophenyl)-2-hydroxy-4-oxobut-2-enoate (**1r**). GP1. A scale of 10 mmol, yellow solid, 1.62 g (7.2 mmol), isolated yield of 72%; ^{1}H NMR (700 MHz, $CDCl_3$) δ ppm 15.09 (s, 1H), 7.96 (td, *J* = 7.5, 1.9 Hz, 1H), 7.58 - 7.55 (m, 1H), 7.30 - 7.27 (m, 1H), 7.18 (ddd, *J* = 11.5, 8.3, 0.9 Hz, 1H), 7.12 (d, *J* = 1.3 Hz, 1H), 3.94 (s, 3H). ^{13}C NMR (176 MHz, $CDCl_3$) δ ppm 187.5, 169.8, 162.4 (d, *J* = 4.9 Hz), 160.9, 135.1 (d, *J* = 9.8 Hz), 130.5, 124.7 (d, *J* = 3.3 Hz), 123.5 (d, *J* = 9.8 Hz), 116.8 (d, *J* = 22.9 Hz), 102.3 (d, *J* = 11.5 Hz), 53.1. IR v_{max}: 3151, 3114, 3085, 2961, 1727, 1589, 1484, 1432, 1271, 1149, 1070, 970, 922, 848, 816, 767, 741, 684, 616, 536 cm^{-1}. mp 74.6 - 76.4 °C. The above analysis results correspond to the literature data.[5]

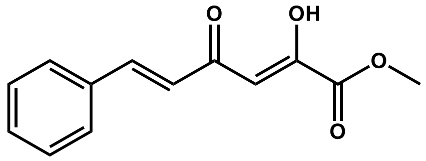

methyl (2Z,5E)-2-hydroxy-4-oxo-6-phenylhexa-2,5-dienoate (**1s**). GP1. A scale of 10 mmol, yellow solid, 1.52 g (6.5 mmol), isolated yield of 65%; ^{1}H NMR (700 MHz, $CDCl_3$) δ ppm 14.86 (br. s, 1H), 7.77 (d, *J* = 15.9 Hz, 1H), 7.61 - 7.59 (m, 2H), 7.45 - 7.44 (m, 3H), 6.69 (d, *J* = 15.8 Hz, 1H), 6.58 (s, 1H), 3.95 (s, 3H). ^{13}C NMR (101 MHz, $CDCl_3$) δ ppm 185.2, 173.9, 162.5, 143.4, 134.3, 130.9, 129.1, 128.5, 123.0, 100.7, 53.1. IR v_{max}: 3060, 2954, 1732, 1630, 1580, 1558, 1436, 1252, 1111, 981, 970, 877, 786, 758, 699, 556, 515, 486 cm^{-1}. mp 68.0 - 70.0 °C. The above analysis results correspond to the literature data.[8]

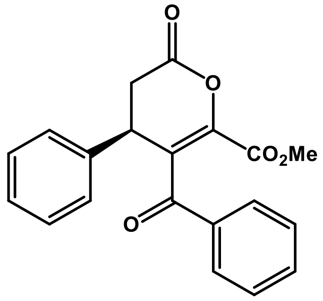

methyl (R) 5-benzoyl-2-oxo-4-phenyl-3,4-dihydro-2H-pyran-6-carboxylate (**3**). Synthetic procedure (S5) (a scale of 0.20 mmol); brown oil (53 mg; 0.16 mmmol; 79% yield). *er* 95:5 (Phenomenex Lux Amylose-1 3µm column, n–hexane/i–PrOH = 80:20, flow rate 1.0 mL/min, λ = 254 nm); ^{1}H NMR (700 MHz, $CDCl_3$) δ ppm 7.64 (dd, *J* = 8.6, 1.1 Hz, 2H), 7.49 (tt, *J* = 7.5, 1.1 Hz, 1H), 7.35 - 7.33 (m, 2H), 7.27 - 7.26 (m, 1H), 7.25 - 7.22 (m, 2H), 7.13 (dd, *J* = 8.0, 1.5 Hz, 2H), 4.18 (dd, *J* = 7.7, 3.0 Hz, 1H), 3.57 (s, 3H), 3.17 (dd, *J* = 15.9, 7.7 Hz, 1H), 3.03 (dd, *J* = 16.4, 3.0 Hz, 1H). ^{13}C NMR (101 MHz, $CDCl_3$) δ ppm 193.0, 164.9, 160.2, 140.2, 137.4, 136.1, 133.6, 129.4, 128.7, 128.6, 128.6, 128.4, 127.1, 52.7, 41.2, 35.1. IR v_{max}: 2935, 2834, 1586, 1469, 1437, 1255, 1224, 1115, 1090, 1051, 772, 712 cm^{-1}. HRMS (ESI-TOF) m/z: $[MNa]^+$ calcd for $C_{20}H_{16}NaO_5$ 359.0895 found: 359.0891

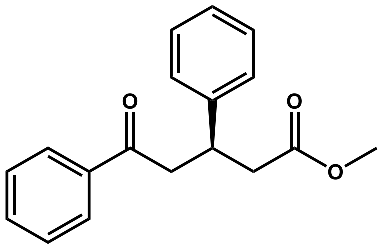

methyl (S)-5-oxo-3,5-diphenylpentanoate (**4a**). GP3. A scale of 0.1 mmol, yellow solid, 26.5 mg (0.094 mmol), isolated yield of 94% (dissolving the crude in Et_2O and filtering through a PTFE 0.45 μm filter gave pure product **4a**); *er* 95:5 (Phenomenex Lux Amylose-1 3μm column, n–hexane/i–PrOH = 80:20, flow rate 1.0 mL/min, λ = 254 nm); ^{1}H NMR (700 MHz, $CDCl_3$) δ ppm 7.94 (dd, *J* = 8.4, 1.3 Hz, 2H), 7.58 - 7.56 (m, 1H), 7.47 - 7.45 (m, 2H), 7.32 - 7.28 (m, 4H), 7.24 - 7.21 (m, 1H), 3.91 (quin, *J* = 7.0 Hz, 1H), 3.61 (s, 3H), 3.42 (dd, *J* = 16.7, 7.0 Hz, 1H), 3.37 (dd, *J* = 16.7, 6.8 Hz, 1H), 2.84 (dd, *J* = 15.4, 7.0 Hz, 1H), 2.72 (dd, *J* = 15.4, 7.9 Hz, 1H). ^{13}C NMR (176 MHz, $CDCl_3$) δ ppm 198.1, 172.2, 143.4, 137.0, 133.0, 128.6, 128.6, 128.1, 127.3, 126.8, 51.5, 44.5, 40.6, 37.6. IR $ν_{max}$: 3027, 3003, 2950, 2926, 2885, 1735, 1720, 1680, 1596, 1437, 1357, 1319, 1264, 1174, 1148, 1030, 1014, 856, 744, 704, 687, 618, 576, 558 cm^{-1}. mp 67.0 - 69.0 °C. HRMS (ESI-TOF) m/z: $[MNa]^+$ calcd for $C_{18}H_{18}O_3Na$ 305.1154 found: 305.1151.

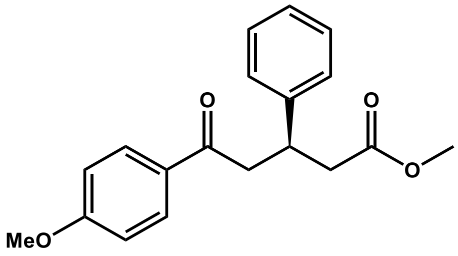

methyl (S)-5-(4-(methoxyphenyl)-5-oxo-3-phenylpentanoate (**4b**). GP3. A scale of 0.2 mmol, orange solid, 58.00 mg (0.19 mmol), isolated yield of 93% (dissolving the crude in Et_2O and filtering through a PTFE 0.45 μm filter gave pure product **4b**); *er* 95:5 (Phenomenex Lux Amylose-1 3μm column, n–hexane/i–PrOH = 80:20, flow rate 1.0 mL/min, λ = 254 nm); ^{1}H NMR (700 MHz, $CDCl_3$) δ ppm 7.93 (d, *J* = 8.8 Hz, 2H), 7.32 - 7.28 (m, 4H), 7.23 - 7.20 (m, 1H), 6.93 (d, *J* = 8.8 Hz, 2H), 3.88 (s, 3H), 3.60 (s, 3H), 3.35 (dd, *J* = 16.3, 7.3 Hz, 1H), 3.30 (dd, *J* = 16.7, 6.8 Hz, 1H), 2.84 (dd, *J* = 15.4, 7.0 Hz, 1H), 2.71 (dd, *J* = 15.4, 7.9 Hz, 1H). ^{13}C NMR (101 MHz, $CDCl_3$) δ ppm 196.7, 172.3, 163.5, 143.5, 130.3, 130.1, 128.6, 127.3, 126.7, 113.7, 55.4, 51.5, 44.2, 40.6, 37.7. IR $ν_{max}$: 2948, 2883, 2841, 1727, 1667, 1598, 1572, 1510, 1456, 1424, 1255, 1214, 1171, 1108, 1049, 901, 845, 812, 763, 701, 612, 563, 513 cm^{-1}. mp 99.0 - 102.0 °C. HRMS (ESI-TOF) m/z: $[MNa]^+$ calcd for $C_{19}H_{20}O_4Na$ 335.1259 found: 335.1268.

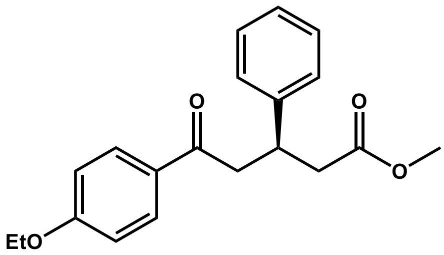

methyl (S)-5-(4-(ethoxyphenyl)-5-oxo-3-phenylpentanoate (**4c**). GP3. A scale of 0.2 mmol, beige solid, 52.27 mg (0.16 mmol), isolated yield of 80% (flash column chromatography AcOEt/hex gradient 0% → 20 % AcOEt); *er* 95:5

(Phenomenex Lux Amylose-1 3μm column, n–hexane/i–PrOH = 80:20, flow rate 1.0 mL/min, λ = 254 nm); ^{1}H NMR (700 MHz, $CDCl_3$) δ ppm 7.91 (d, *J* = 8.8 Hz, 2H), 7.32 - 7.28 (m, 4H), 7.23 - 7.21 (m, 1H), 6.91 (d, *J* = 8.8 Hz, 2H), 4.10 (q, *J* = 7.0 Hz, 2H), 3.89 (quin, *J* = 7.3 Hz, 1H), 3.60 (s, 3H), 3.35 (dd, *J* = 16.7, 7.3 Hz, 1H), 3.30 (dd, *J* = 16.3, 6.8 Hz, 1H), 2.84 (dd, *J* = 15.4, 7.0 Hz, 1H), 2.70 (dd, *J* = 15.9, 7.9 Hz, 1H), 1.45 (t, *J* = 7.0 Hz, 3H). ^{13}C NMR (176 MHz, $CDCl_3$) δ ppm 196.6, 172.3, 162.9, 143.5, 130.4, 129.9, 128.6, 127.3, 126.7, 114.2, 63.7, 51.5, 44.2, 40.6, 37.7, 14.6. IR v_{max} : 2992, 2936, 2874, 1731, 1673, 1602, 1574, 1428, 1312, 1267, 1215, 1180, 1157, 1115, 1085, 1019, 983, 920, 843, 807, 765, 702, 616, 588, 563, 541 cm^{-1}. mp 82.0 - 84.0 °C. HRMS (ESI-TOF) m/z: $[MNa]^+$ calcd for $C_{20}H_{22}O_4Na$ 349.1416 found: 349.1419.

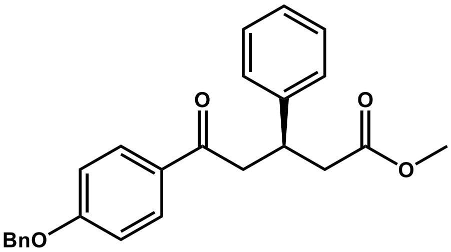

methyl (S)-5-(4-(benzyloxy)phenyl)-5-oxo-3-phenylpentanoate (**4d**). GP3. A scale of 0.2 mmol, white solid, 77.66 mg (0.19 mmol), isolated yield of 94% (dissolving the crude in Et_2O and filtering through a PTFE 0.45 μm filter gave pure product **4d**); *er* 83:17 (Phenomenex Lux Amylose-1 3μm column, n–hexane/i–PrOH = 70:30, flow rate 1.0 mL/min, λ = 254 nm); ^{1}H NMR (700 MHz, $CDCl_3$) δ ppm 7.93 - 7.92 (m, 2H), 7.45 - 7.41 (m, 4H), 7.38 - 7.36 (m, 1H), 7.32 - 7.28 (m, 4H), 7.23 - 7.21 (m, 1H), 7.02 - 7.00 (m, 2H), 5.14 (s, 2H), 3.89 (quin, *J* = 7.3 Hz, 1H), 3.60 (s, 3H), 3.35 (dd, *J* = 16.3, 7.3 Hz, 1H), 3.30 (dd, *J* = 16.7, 6.8 Hz, 1H), 2.84 (dd, *J* = 15.4, 6.8 Hz, 1H), 2.71 (dd, *J* = 15.9, 7.9 Hz, 1H). ^{13}C NMR (101 MHz, $CDCl_3$) δ ppm 196.6, 172.3, 162.6, 143.5, 136.2, 130.4, 130.3, 128.7, 128.6, 128.2, 127.4, 127.3, 126.7, 114.6, 70.2, 51.5, 44.2, 40.6, 37.7. IR v_{max} : 3031, 2950, 2883, 1728, 1667, 1598, 1573, 1510, 1496, 1454, 1421, 1357, 1312, 1257, 1214, 1174, 1148, 984, 837, 810, 742, 696, 555, 517 cm^{-1}. mp 86.0 - 90.0 °C. HRMS (ESI-TOF) m/z: $[MNa]^+$ calcd for $C_{25}H_{24}O_4Na$ 411.1572 found: 411.1578.

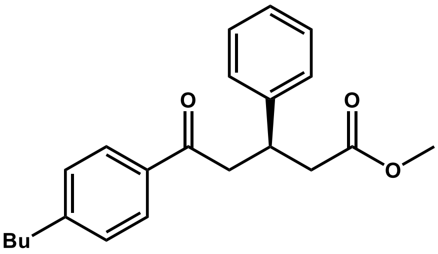

methyl (S)-5-(4-butylphenyl)-5-oxo-3-phenylpentanoate (**4e**). GP3. A scale of 0.2 mmol, colorless oil, 47 mg (0.14 mmol), isolated yield of 70% (flash column chromatography AcOEt/hex gradient 0% → 20 % AcOEt); *er* 72:28 (Phenomenex Lux Amylose-1 3μm column, n–hexane/i–PrOH = 90:10, flow rate 1.0 mL/min, λ = 254 nm); ^{1}H NMR (700 MHz, $CDCl_3$) δ ppm 7.87 - 7.85 (m, 2H), 7.32 - 7.28 (m, 4H), 7.26 (d, *J* = 8.4 Hz, 2H), 7.23 - 7.21 (m, 1H), 3.90 (quin, *J* = 7.0 Hz, 1H), 3.60 (s, 3H), 3.38 (dd, *J* = 16.7, 7.0 Hz, 1H), 3.34 (dd, *J* = 16.7, 7.0 Hz, 1H), 2.84 (dd, *J* = 15.4, 7.0 Hz, 1H), 2.71 (dd, *J* = 15.4, 7.9 Hz, 1H), 2.67 (t, *J* = 7.7 Hz, 2H), 1.65 - 1.60 (m, 2H), 1.37 (sex, *J* = 7.5 Hz, 2H), 0.95 (t, *J*

= 7.5 Hz, 3H). ^{13}C NMR (75.5 MHz, $CDCl_3$) δ ppm 197.7, 172.3, 148.8, 143.4, 134.6, 128.6, 128.6, 128.2, 127.3, 126.7, 51.5, 44.4, 40.5, 37.5, 35.6, 33.2, 22.3, 13.9. IR ν_{max} : 3029, 2954, 2930, 2859, 1734, 1680, 1605, 1495, 1454, 1436, 1413, 1363, 1267, 1218, 1178, 1150, 1118, 1005, 988, 885, 823, 762, 699, 555 cm^{-1}. HRMS (ESI-TOF) m/z: $[MNa]^+$ calcd for $C_{22}H_{26}O_3Na$ 361.1780 found: 361.1782.

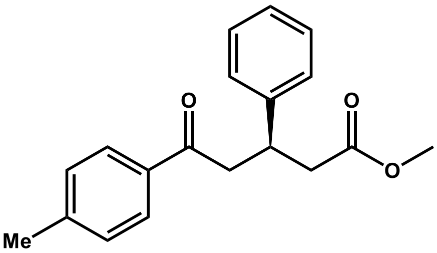

methyl (S)-5-oxo-3-phenyl-5-(p-tolyl)pentanoate (**4f**). GP3. A scale of 0.2 mmol, white solid, 49.00 mg (0.17 mmol), isolated yield of 83% (flash column chromatography AcOEt/hex gradient 0% → 20 % AcOEt); *er* 95:5 (Phenomenex Lux Cellulose-1 3µm column, n–hexane/i–PrOH = 90:10, flow rate 1.0 mL/min, λ = 254 nm); ^{1}H NMR (700 MHz, $CDCl_3$) δ ppm 7.84 (d, J = 8.4 Hz, 2H), 7.32 - 7.28 (m, 4H), 7.25 (d, J = 7.9 Hz, 2H), 7.23 - 7.21 (m, 1H), 3.90 (quin, J = 7.0 Hz, 1H), 3.60 (s, 3H), 3.38 (dd, J = 16.7, 7.5 Hz, 1H), 3.33 (dd, J = 16.7, 7.0 Hz, 1H), 2.84 (dd, J = 15.4, 7.0 Hz, 1H), 2.71 (dd, J = 15.4, 7.9 Hz, 1H), 2.42 (s, 3H). ^{13}C NMR (75.5 MHz, $CDCl_3$) δ ppm 197.7, 172.3, 143.9, 143.4, 134.4, 129.2, 128.6, 128.2, 127.3, 126.7, 51.5, 44.4, 40.5, 37.5, 21.6. IR ν_{max} : 3030, 2997, 2951, 2883, 1729, 1673, 1605, 1496, 1433, 1377, 1268, 1255, 1221, 1199, 1184, 1161, 1047, 1017, 1006, 856, 808, 763, 700, 560, 498, 452 cm^{-1}. mp 80.0 - 82.5 °C. HRMS (ESI-TOF) m/z: $[MNa]^+$ calcd for $C_{19}H_{20}O_3Na$ 319.1310 found: 319.1313.

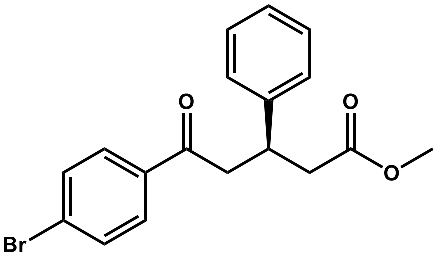

methyl (S)-(4-bromophenyl)-5-oxo-3-phenylpentanoate (**4g**). GP3. A scale of 0.2 mmol, yellow solid, 70.4 mg (0.19 mmol), isolated yield of 97% (dissolving the crude in Et_2O and filtering through a PTFE 0.45 µm filter gave pure product **4g**); *er* 93:7 (Phenomenex Lux Amylose-1 3µm column, n–hexane/i–PrOH = 80:20, flow rate 1.0 mL/min, λ = 254 nm); ^{1}H NMR (700 MHz, $CDCl_3$) δ ppm 7.77 (d, J = 8.8 Hz, 2H), 7.57 (d, J = 8.4 Hz, 2H), 7.30 - 7.27 (m, 2H), 7.24 (dd, J = 7.9, 1.3 Hz, 2H), 7.21 - 7.19 (m, 1H), 3.85 (quin, J = 7.0 Hz, 1H), 3.59 (s, 3H), 3.37 (dd, J = 17.2, 6.8 Hz, 1H), 3.29 (dd, J = 16.7, 7.0 Hz, 1H), 2.80 (dd, J = 15.4, 7.3 Hz, 1H), 2.69 (dd, J = 15.9, 7.5 Hz, 1H). ^{13}C NMR (176 MHz, $CDCl_3$) δ ppm 197.1, 172.2, 143.1, 135.7, 131.9, 129.6, 128.6, 128.2, 127.3, 126.9, 51.5, 44.5, 40.5, 37.6. IR ν_{max}: 3061, 3030, 2952, 1732, 1684, 1584, 1495, 1436, 1397, 1359, 1264, 1214, 1151, 1072, 986, 883, 811, 763, 699, 614, 563, 445 cm^{-1}. mp 59.7 - 61.9 °C. HRMS (ESI-TOF) m/z: $[MNa]^+$ calcd for $C_{18}H_{17}O_3NaBr$ 383.0259 found: 383.0258.

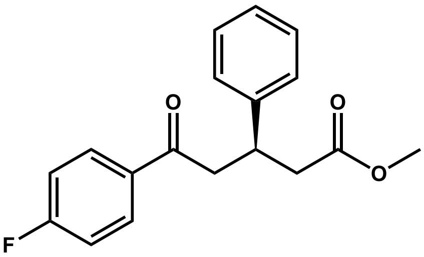

methyl (S)-(4-fluorophenyl)-5-oxo-3-phenylpentanoate (**4h**). GP3. A scale of 0.1 mmol, orange oil, 25.0 mg (0.083 mmol), isolated yield of 83% (dissolving the crude in Et_2O and filtering through a PTFE 0.45 μm filter gave pure product **4h**); *er* 94:6 (Phenomenex Lux Amylose-1 3μm column, n–hexane/i–PrOH = 80:20, flow rate 1.0 mL/min, λ = 254 nm); ^{1}H NMR (700 MHz, $CDCl_3$) δ ppm 7.98 - 7.95 (m, 2H), 7.32 - 7.30 (m, 2H), 7.28 - 7.27 (m, 2H), 7.24 - 7.21 (m, 1H), 7.14 - 7.11 (m, 2H), 3.88 (quin, *J* = 7.0 Hz, 1H), 3.62 (s, 3H), 3.40 (dd, *J* = 16.7, 7.0 Hz, 1H), 3.32 (dd, *J* = 16.7, 7.0 Hz, 1H), 2.83 (dd, *J* = 15.9, 7.3 Hz, 1H), 2.72 (dd, *J* = 15.4, 7.5 Hz, 1H). ^{13}C NMR (176 MHz, $CDCl_3$) δ ppm 196.5, 172.2, 165.7 (d, *J* = 255.0 Hz), 143.2, 133.4, 130.7 (d, *J* = 8.2 Hz), 128.6, 127.3, 126.9, 115.6 (d, *J* = 22.9 Hz), 51.5, 44.4, 40.5, 37.6. IR $ν_{max}$: 3065, 3030, 2952, 1732, 1682, 1595, 1506, 1436, 1410, 1363, 1225, 1154, 1010, 989, 834, 761, 699, 555 cm^{-1}. HRMS (ESI-TOF) m/z: $[MNa]^+$ calcd for $C_{18}H_{17}O_3NaF$ 323.1059 found: 323.1060.

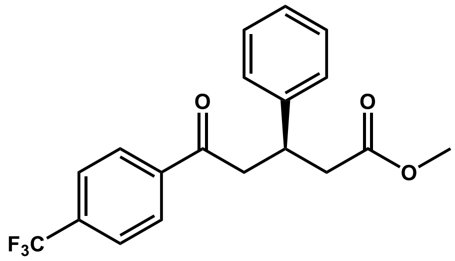

methyl (S)-5-oxo-3-phenyl-5-(4-(trifluoromethyl)phenyl)pentanoate (**4i**). GP3. A scale of 0.2 mmol, orange oil, 18.76 mg (0.054 mmol), isolated yield of 27% (flash column chromatography AcOEt/hex gradient 0% → 20 % AcOEt); *er* 92:8 (Phenomenex Lux Amylose-1 3μm column, n–hexane/i–PrOH = 80:20, flow rate 1.0 mL/min, λ = 254 nm); ^{1}H NMR (700 MHz, $CDCl_3$) δ ppm 8.01 (d, *J* = 8.4 Hz, 2H), 7.70 (d, *J* = 7.9 Hz, 2H), 7.30 - 7.28 (m, 2H), 7.26 - 7.24 (m, 2H), 7.22 - 7.20 (m, 1H), 3.87 (quin, *J* = 7.0 Hz, 1H), 3.60 (s, 3H), 3.44 (dd, *J* = 16.7, 7.0 Hz, 1H), 3.35 (dd, *J* = 17.2, 7.3 Hz, 1H), 2.81 (dd, *J* = 15.9, 7.5 Hz, 1H), 2.71 (dd, *J* = 15.9, 7.3 Hz, 1H). ^{13}C NMR (176 MHz, $CDCl_3$) δ ppm 197.2, 172.2, 143.0, 139.7, 134.4 (q, *J* = 32.7 Hz), 128.7, 128.4, 127.2, 127.0, 125.6 (q, *J* = 3.3 Hz), 123.6 (q, *J* = 273.0 Hz), 51.5, 44.8, 40.5, 37.6. IR $ν_{max}$: 3065, 3031, 2953, 1733, 1690, 1495, 1437, 1410, 1322, 1267, 1165, 1124, 1109, 1064, 1014, 991, 962, 832, 765, 700, 607, 539 cm^{-1}. HRMS (ESI-TOF) m/z: $[MNa]^+$ calcd for $C_{19}H_{17}O_3NaF_3$ 373.1027 found: 373.1031.

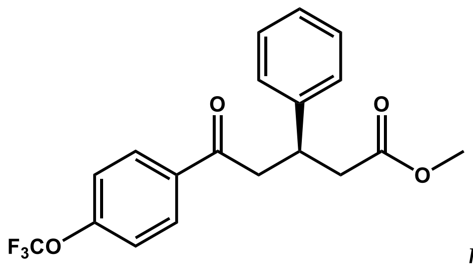

methyl (S)-5-oxo-3-phenyl-5-(4-trifluoromethoxy)phenyl)pentanoate (**4j**). GP3. A scale of 0.25 mmol, yellow oil, 55 mg (0.15 mmol), isolated yield of 60% (flash column chromatography AcOEt/hex gradient 0% → 20 % AcOEt); *er* 82:18 (Phenomenex Lux Amylose-1 3μm column, n–hexane/i–PrOH = 90:10, flow rate 1.0 mL/min, λ = 254 nm); ^{1}H NMR (400 MHz, $CDCl_3$) δ ppm 7.98 - 7.94 (m, 2H), 7.31 - 7.18 (m, 7H), 3.86 (quin, *J* = 7.0 Hz, 1H), 3.60 (s, 3H), 3.40 (dd, *J* = 16.7, 6.8 Hz, 1H), 3.31 (dd, *J* = 16.7, 7.0 Hz, 1H), 2.81 (dd, *J* = 15.6, 7.3 Hz, 1H), 2.70 (dd, J = 15.6, 7.3 Hz, 1H). ^{13}C NMR (75.5 MHz, $CDCl_3$) δ ppm 196.6, 172.2, 152.6, 143.0, 135.1, 130.1, 128.6, 127.2, 126.9, 120.3, 120.2 (q, *J* = 258.5 Hz), 51.6, 44.5, 40.4, 37.5. IR ν_{max} : 3031, 2953, 1734, 1687, 1602, 1497, 1454, 1437, 1413, 1366, 1251, 1205, 1155, 1013, 989, 880, 849, 762, 728, 699, 543 cm^{-1}. HRMS (ESI-TOF) m/z: $[MNa]^+$ calcd for $C_{19}H_{17}O_4F_3Na$ 389.0977 found: 389.0985.

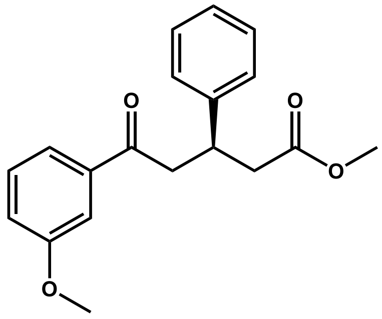

methyl (S)-5-(3-methoxyphenyl)-5-oxo-3-phenylpentanoate (**4k**). GP3. A scale of 0.3 mmol, brown solid, 85.5 mg (0.27 mmol), isolated yield of 91% (dissolving the crude in Et_2O and filtering through a PTFE 0.45 μm filter gave pure product **4k**); *er* 95:5 (Phenomenex Lux Cellulose-1 3μm column, n–hexane/i–PrOH = 90:10, flow rate 1.0 mL/min, λ = 254 nm); ^{1}H NMR (700 MHz, $CDCl_3$) δ ppm 7.51 - 7.49 (m, 1H), 7.44 (dd, *J* = 2.6, 1.8 Hz, 1H), 7.34 (t, *J* = 7.9 Hz, 1H), 7.30 - 7.26 (m, 4H), 7.21 - 7.18 (m, 1H), 7.08 (ddd, *J* = 8.4, 2.6, 0.9 Hz, 1H), 3.88 (quin, *J* = 7.3 Hz, 1H), 3.82 (s, 3H), 3.58 (s, 3H), 3.38 (dd, *J* = 16.7, 7.0 Hz, 1H), 3.32 (dd, *J* = 17.2, 7.0 Hz, 1H), 2.81 (dd, *J* = 15.4, 7.0 Hz, 1H), 2.69 (dd, *J* = 15.6, 7.7 Hz, 1H). ^{13}C NMR (176 MHz, $CDCl_3$) δ ppm 197.9, 172.2, 159.9, 143.4, 138.4, 129.5, 128.6, 127.3, 126.8, 120.7, 119.6, 112.4, 55.4, 51.5, 44.7, 40.6, 37.6. IR ν_{max} : 2954, 2900, 2835, 1731, 1680, 1581, 1487, 1453, 1432, 1414, 1372, 1276, 1255, 1183, 1156, 1082, 999, 890, 861, 780, 756, 700, 664, 627, 602, 562, 528, 506 cm^{-1}. mp 53.1 - 57.4 °C. HRMS (ESI-TOF) m/z: $[MNa]^+$ calcd for $C_{19}H_{20}O_4Na$ 335.1259 found: 335.1258.

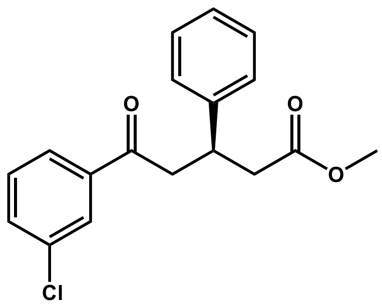

methyl (S)-5-(3-chlorophenyl)-5-oxo-3-phenylpentanoate (**4l**). GP3. A scale of 0.2 mmol, yellow solid, 10.4 mg (0.033 mmol), isolated yield of 16% (flash column chromatography AcOEt/hex gradient 0% → 20 % AcOEt); *er* 92:8 (Phenomenex Lux Cellulose-1 3μm column, n–hexane/i–PrOH = 80:20, flow rate 1.0 mL/min, λ = 254 nm); ^{1}H NMR (700 MHz, $CDCl_3$) δ ppm 7.87 (t, *J* = 1.8 Hz, 1H), 7.79 (ddd, *J* = 7.9, 1.8, 0.9 Hz, 1H), 7.51 (ddd, *J* = 7.9, 2.2, 0.9 Hz, 1H), 7.38 (t, *J* = 7.9 Hz, 1H), 7.30 - 7.28 (m, 2H), 7.26 - 7.24 (m, 2H), 7.22 - 7.19 (m, 1H), 3.86 (quin, *J* = 7.0 Hz, 1H), 3.60 (s, 3H), 3.38 (dd, *J* = 16.7, 7.0 Hz, 1H), 3.31 (dd, *J* = 17.2, 7.0 Hz, 1H), 2.80 (dd, *J* = 15.4, 7.5 Hz, 1H), 2.70 (dd, *J* = 15.9, 7.5 Hz, 1H). ^{13}C NMR (176 MHz, $CDCl_3$) δ ppm 196.8, 172.1, 143.1, 138.6, 135.0, 132.9, 129.9, 128.6, 128.2, 127.3, 126.9, 126.1, 51.5, 44.6, 40.5, 37.5. IR ν_{max} : 3035, 2959, 2912, 1720, 1679, 1570, 1439, 1418, 1374, 1358, 1347, 1263, 1232, 1214, 1144, 992, 807, 764, 701, 683, 620, 560, 462 cm^{-1}. mp 81.5 - 85.7 °C. HRMS (ESI-TOF) m/z: $[MNa]^+$ calcd for $C_{18}H_{17}O_3NaCl$ 339.0764 found: 339.0769.

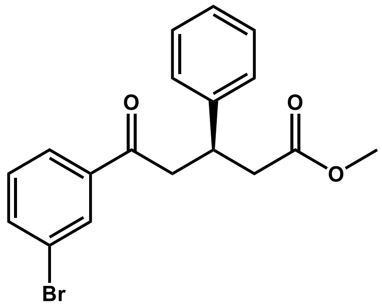

methyl (S)-5-(3-bromophenyl)-5-oxo-3-phenylpentanoate (**4m**). GP3. A scale of 0.2 mmol, yellow solid, 43.0 mg (0.12 mmol), isolated yield of 60% (flash column chromatography AcOEt/hex gradient 0% → 20 % AcOEt); *er* 94:6 (Phenomenex Lux Cellulose-1 3μm column, n–hexane/i–PrOH = 90:10, flow rate 1.0 mL/min, λ = 254 nm); ^{1}H NMR (700 MHz, $CDCl_3$) δ ppm 8.02 (t, *J* = 1.8 Hz, 1H), 7.84 - 7.83 (m, 1H), 7.67 - 7.66 (m, 1H), 7.32 (t, *J* = 7.9 Hz, 1H), 7.30 - 7.28 (m, 2H), 7.26 - 7.24 (m, 2H), 7.22 - 7.19 (m, 1H), 3.86 (quin, *J* = 7.3 Hz, 1H), 3.60 (s, 3H), 3.37 (dd, *J* = 17.2, 6.8 Hz, 1H), 3.30 (dd, *J* = 16.7, 7.0 Hz, 1H), 2.80 (dd, *J* = 15.9, 7.5 Hz, 1H), 2.70 (dd, *J* = 15.8, 7.5 Hz, 1H). ^{13}C NMR (176 MHz, $CDCl_3$) δ ppm 196.7, 172.1, 143.1, 138.7, 135.9, 131.2, 130.1, 128.7, 127.3, 126.9, 126.5, 123.0, 51.5, 44.6, 40.5, 37.5. IR ν_{max} : 3068, 3034, 2984, 2945, 1730, 1682, 1597, 1562, 1496, 1436, 1365, 1271, 1209, 1154, 1088, 1068, 1031, 992, 891, 807, 766, 561 cm^{-1}. mp 90.1 - 92.0 °C. HRMS (ESI-TOF) m/z: $[MNa]^+$ calcd for $C_{18}H_{17}O_3NaBr$ 383.0259 found: 383.0264.

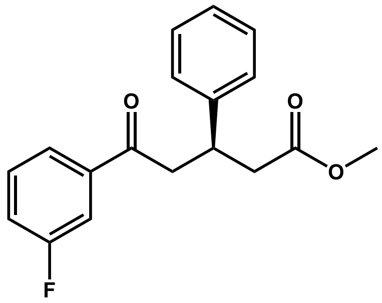

methyl (S)-5-(3-fluorophenyl)-5-oxo-3-phenylpentanoate (**4n**). GP3. A scale of 0.2 mmol, orange solid, 32 mg (0.11 mmol), isolated yield of 53% (flash column chromatography AcOEt/hex gradient 0% → 20 % AcOEt); *er* 92:8 (Phenomenex Lux Cellulose-1 3μm column, n–hexane/i–PrOH = 90:10, flow rate 1.0 mL/min, λ = 254 nm); ^{1}H NMR (700 MHz, $CDCl_3$) δ ppm 7.73 - 7.72 (m, 1H), 7.62 - 7.60 (m, 1H), 7.44 (td, *J* = 8.0, 5.5 Hz, 1H), 7.33 - 7.30 (m, 2H), 7.28 - 7.25 (m, 3H), 7.24 - 7.22 (m, 1H), 3.89 (quin, *J* = 7.3 Hz, 1H), 3.62 (s, 3H), 3.41 (dd, *J* = 16.7, 7.0 Hz, 1H), 3.34 (dd, *J* = 16.7, 7.0 Hz, 1H), 2.83 (dd, *J* = 15.6, 7.3 Hz, 1H), 2.73 (dd, *J* = 15.4, 7.5 Hz, 1H). ^{13}C NMR (75.5 MHz, $CDCl_3$) δ ppm 196.8, 172.2, 162.8 (d, *J* = 247.7 Hz), 143.1, 138.9 (d, *J* = 5.9 Hz), 130.2 (d, *J* = 7.6 Hz), 128.7, 127.3, 126.9, 123.8, 120.1 (d, *J* = 21.5 Hz), 114.8 (d, J = 22.1 Hz), 51.6, 44.6, 40.5, 37.4. IR v_{max} : 3072, 3036, 2952, 2911, 1722, 1679, 1586, 1495, 1434, 1358, 1267, 1240, 1143, 1085, 977, 850, 767, 701, 680, 626, 527 cm^{-1}. mp 55.1 - 57.3 °C. HRMS (ESI-TOF) m/z: $[MNa]^+$ calcd for $C_{18}H_{17}O_3FNa$ 323.1059 found: 323.1055.

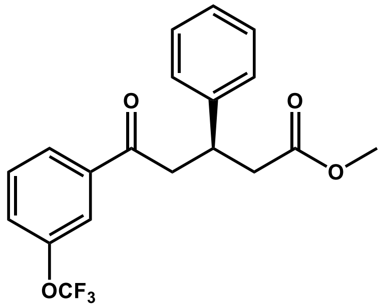

methyl (S)-5-oxo-3-phenyl-5-(3-(trifluoromethoxy)phenyl)pentanoate (**4o**). GP3. A scale of 0.25 mmol, yellow oil, 27 mg (0.074 mmol), isolated yield of 29% (flash column chromatography AcOEt/hex gradient 0% → 20 % AcOEt); *er* 90:10 (Phenomenex Lux Cellulose-1 3μm column, n–hexane/i–PrOH = 90:10, flow rate 1.0 mL/min, λ = 254 nm); ^{1}H NMR (700 MHz, $CDCl_3$) δ ppm 7.87 - 7.86 (m, 1H), 7.76 (s, 1H), 7.50 (t, *J* = 7.9 Hz, 1H), 7.43 - 7.41 (m, 1H), 7.33 - 7.31 (m, 2H), 7.29 - 7.27 (m, 2H), 7.24 - 7.22 (m, 1H), 3.89 (quin, *J* = 7.5 Hz, 1H), 3.62 (s, 3H), 3.42 (dd, *J* = 16.7, 6.8 Hz, 1H), 3.35 (dd, *J* = 16.7, 7.3 Hz, 1H), 2.83 (dd, *J* = 15.4, 7.3 Hz, 1H), 2.73 (dd, *J* = 15.9, 7.5 Hz, 1H). ^{13}C NMR (101 MHz, $CDCl_3$) δ ppm 196.6, 172.2, 149.5, 143.0, 138.8, 130.1, 128.7, 127.3, 126.9, 126.3, 125.3, 120.4, 120.4 (q, *J* = 258.2 Hz), 51.6, 44.6, 40.5, 37.5. IR v_{max} : 3031, 2953, 1734, 1689, 1586, 1495, 1439, 1366, 1251, 1210, 1150, 1027, 1001, 887, 844, 792, 762, 699, 632, 561, 538, 482 cm^{-1}. HRMS (ESI-TOF) m/z: $[MNa]^+$ calcd for $C_{19}H_{17}O_4F_3Na$ 389.0977 found: 389.0974.

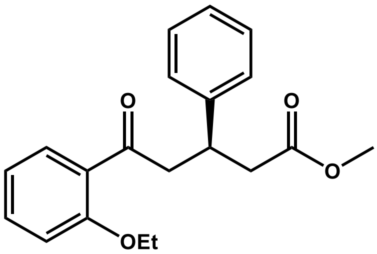

methyl (S)-5-(2-ethoxyphenyl)-5-oxo-3-phenylpentanoate (**4p**). GP3. A scale of 0.2 mmol, yellow oil, 26 mg (0.080 mmol), isolated yield of 40% (flash column chromatography AcOEt/hex gradient 0% → 20 % AcOEt); *er* 95:5 (Phenomenex Lux Amylose-1 3μm column, n–hexane/i–PrOH = 90:10, flow rate 1.0 mL/min, λ = 254 nm); ^{1}H NMR (700 MHz, $CDCl_3$) δ ppm 7.56 (dd, *J* = 7.9, 1.8 Hz, 1H), 7.43 (ddd, *J* = 8.5, 7.3, 1.8 Hz, 1H), 7.31 - 7.29 (m, 1H), 7.27 - 7.24 (m, 3H), 7.22 - 7.18 (m, 1H), 6.96 (td, *J* = 7.5 0.9 Hz, 1H), 6.93 (d, *J* = 8.4 Hz, 1H), 4.14 (qd, *J* = 7.0, 1.8 Hz, 2H), 3.83 - 3.88 (m, 1H), 3.59 (s, 3H), 3.45 (dd, *J* = 7.5, 3.5 Hz, 2H), 2.78 (dd, *J* = 15.0, 6.6 Hz, 1H), 2.66 (dd, *J* = 15.4, 8.4 Hz, 1H), 1.46 (t, *J* = 7.0 Hz, 3H). ^{13}C NMR (176 MHz, $CDCl_3$) δ ppm 200.6, 172.3, 157.8, 143.7, 133.3, 130.3, 128.5, 128.4, 127.4, 126.5, 120.6, 112.2, 64.1, 51.4, 49.8, 41.0, 37.6, 14.7. IR v_{max} : 3063, 3029, 2980, 2950, 1734, 1671, 1596, 1488, 1473, 1449, 1288, 1237, 1160, 1120, 1083, 990, 923, 754, 699, 526 cm^{-1}. HRMS (ESI-TOF) m/z: $[MNa]^+$ calcd for $C_{20}H_{22}O_4Na$ 349.1416 found: 349.1422.

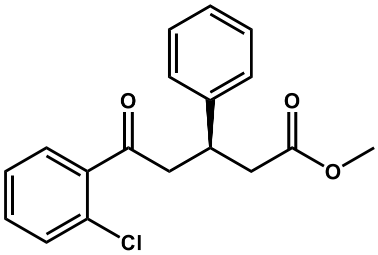

methyl (S)-5-(2-chlorophenyl)-5-oxo-3-phenylpentanoate (**4q**). GP3. A scale of 0.2 mmol, pink oil, 5 mg (0.016 mmol), isolated yield of 8% (flash column chromatography AcOEt/hex gradient 0% → 20 % AcOEt); *er* 94:6 (Phenomenex Lux Cellulose-1 3μm column, n–hexane/i–PrOH = 90:10, flow rate 1.0 mL/min, λ = 254 nm); ^{1}H NMR (700 MHz, $CDCl_3$) δ ppm 7.37 (dd, *J* = 7.5, 0.9 Hz, 1H), 7.35 - 7.33 (m, 1H), 7.28 - 7.18 (m, 7H), 3.82 (quin, *J* = 7.5 Hz, 1H), 3.59 (s, 3H), 3.40 (dd, *J* = 17.2, 6.8 Hz, 1H), 3.31 (dd, *J* = 17.2, 7.5 Hz, 1H), 2.78 (dd, *J* = 15.4, 7.0 Hz, 1H), 2.68 (dd, *J* = 15.4, 7.9 Hz, 1H). ^{13}C NMR (75.5 MHz, $CDCl_3$) δ ppm 201.5, 172.1, 142.7, 139.4, 131.6, 130.7, 130.4, 128.8, 128.6, 127.4, 126.9, 126.8, 51.6, 48.7, 40.6, 37.6. IR v_{max} : 3062, 3029, 2951, 2849, 1735, 1699, 1590, 1494, 1454, 1434, 1411, 1365, 1324, 1263, 1214, 1193, 1155, 1111, 1067, 1032, 1014, 992, 959, 700, 646 cm^{-1}. HRMS (ESI-TOF) m/z: $[MNa]^+$ calcd for $C_{18}H_{17}O_3NaCl$ 339.0764 found: 339.0763.

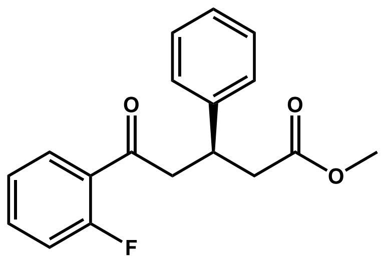

methyl (S)-5-(2-fluorophenyl)-5-oxo-3-phenylpentanoate (**4r**). GP3. A scale of 0.2 mmol, beige solid, 32 mg (0.11 mmol), isolated yield of 53% (flash column chromatography AcOEt/hex gradient 0% → 20 % AcOEt); *er* 95:5 (Phenomenex Lux Amylose-1 3μm column, n–hexane/i–PrOH = 90:10, flow rate 1.0 mL/min, λ = 254 nm); ^{1}H NMR (700 MHz, $CDCl_3$) δ ppm 7.77 (td, *J* = 7.7, 1.8 Hz, 1H), 7.53 - 7.50 (m, 1H), 7.32 - 7.27 (m, 4H), 7.23 - 7.20 (m, 2H), 7.14 (dd, *J* = 11.4, 0.9 Hz, 1H), 3.90 (quin, *J* = 7.0 Hz, 1H), 3.61 (s, 3H), 3.41 (dd, *J* = 7.0, 2.6 Hz, 2H), 2.80 (dd, *J* = 15.9, 6.8 Hz, 1H), 2.71 (dd, *J* = 15.4, 7.9 Hz, 1H). ^{13}C NMR (176 MHz, $CDCl_3$) δ ppm 196.5 (d, *J* = 4.0 Hz), 172.2, 161.8 (d, *J* = 254.1 Hz), 143.3, 134.5 (d, *J* = 9.0 Hz), 130.7 (d, J = 2.5 Hz), 128.6, 127.4, 126.8, 125.7 (d, *J* = 13.1 Hz), 124.5 (d, *J* = 3.4 Hz), 116.6 (d, *J* = 23.8 Hz), 51.6, 49.4 (d, *J* = 7.2 Hz), 40.7, 37.3. IR v_{max} : 3031, 3001, 2950, 2900, 2841, 1739, 1720, 1683, 1607, 1478, 1452, 1434, 1305, 1275, 1229, 1144, 992, 903, 837, 717, 538, 511 cm^{-1}. mp 74.0 - 76.2 °C. HRMS (ESI-TOF) m/z: $[MNa]^+$ calcd for $C_{18}H_{17}O_3FNa$ 323.1059 found: 323.1067.

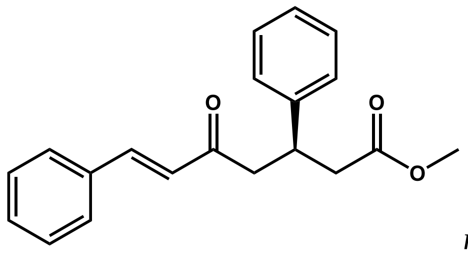

methyl (S,E)-5-oxo-3,7-diphenylhept-6-enoate (**4s**). GP3. A scale of 0.2 mmol, yellow solid, 33.26 mg (0.11 mmol), isolated yield of 54% (flash column chromatography AcOEt/hex gradient 0% → 20 % AcOEt); *er* 92:8 (Phenomenex Lux Cellulose-1 3μm column, n–hexane/i–PrOH = 90:10, flow rate 1.0 mL/min, λ = 254 nm); ^{1}H NMR (400 MHz, $CDCl_3$) δ ppm 7.53 - 7.49 (m, 3H), 7.40 - 7.37 (m, 3H), 7.32 - 7.25 (m, 4H), 7.22 - 7.18 (m, 1H), 6.67 (d, *J* = 16.1 Hz, 1H), 3.80 (quin, *J* = 7.3 Hz, 1H), 3.60 (s, 3H), 3.12 - 3.00 (m, 2H), 2.79 (dd, *J* = 15.6, 6.9 Hz, 1H), 2.68 (dd, *J* = 15.6, 7.9 Hz, 1H). ^{13}C NMR (101 MHz, $CDCl_3$) δ ppm 198.1, 172.3, 143.2, 142.9, 134.4, 130.5, 128.9, 128.6, 128.3, 127.3, 126.9, 126.1, 51.6, 46.8, 40.5, 37.7. IR v_{max}: 3060, 3025, 2953, 1731, 1642, 1495, 1427, 1382, 1330, 1262, 1189, 1078, 1049, 968, 843, 762, 696, 555, 491, 465, 409 cm^{-1}. mp 92.6 - 94.0°C. HRMS (ESI-TOF) m/z: $[MNa]^+$ calcd for $C_{20}H_{20}O_3Na$ 331.1310 found: 331.1316.

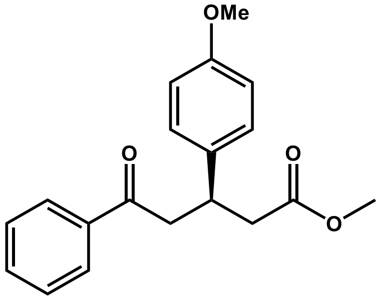

methyl (S)-3-(4-methoxyphenyl)-5-oxo-5-phenylpentanoate (**4t**). GP3. A scale of 0.2 mmol, beige solid, 37.8 mg (0.12 mmol), isolated yield of 60% (flash column chromatography AcOEt/hex gradient 0% → 20 % AcOEt); *er* 92:8 (Phenomenex Lux Amylose-1 3μm column, n–hexane/i–PrOH = 90:10, flow rate 1.0 mL/min, λ = 254 nm); ^{1}H NMR (400 MHz, $CDCl_3$) δ ppm 7.93 - 7.90 (m, 2H), 7.56 - 7.52 (m, 1H), 7.45 - 7.41 (m, 2H), 7.18 (d, *J* = 8.5 Hz, 2H), 6.82 (d, *J* = 8.8 Hz, 2H), 3.83 (quin, *J* = 7.2 Hz, 1H), 3.76 (s, 3H), 3.59 (s, 3H), 3.37 (dd, *J* = 16.7, 7.0 Hz, 1H), 3.30 (dd, *J* = 16.7, 7.0 Hz, 1H), 2.79 (dd, *J* = 16.9, 15.3 Hz, 1H), 2.66 (dd, *J* = 15.3, 7.8 Hz, 1H). ^{13}C NMR (75.5 MHz, $CDCl_3$) δ ppm 198.3, 172.3, 158.3, 136.9, 135.3, 133.0, 128.5, 128.2, 128.0, 114.0, 55.1, 51.5, 44.7, 40.8, 36.8. IR ν_{max}: 2906, 2839, 1724, 1673, 1597, 1581, 1515, 1434, 1350, 1301, 1252, 1220, 1179, 1148, 1109, 993, 979, 936, 760, 691, 560 cm^{-1}. mp 61.0 - 64.7 °C. HRMS (ESI-TOF) m/z: $[MNa]^+$ calcd for $C_{19}H_{20}O_4Na$ 335.1259 found: 335.1268.

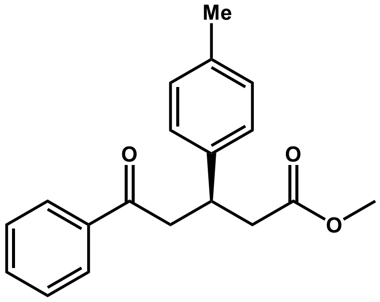

methyl (S)-5-oxo-5-phenyl-3-(p-tolyl)pentanoate (**4u**). GP3. A scale of 0.2 mmol, white solid, 35.00 mg (0.12 mmol), isolated yield of 59% (flash column chromatography AcOEt/hex gradient 0% → 20 % AcOEt); *er* 94:6 (Phenomenex Lux Amylose-1 3μm column, n–hexane/i–PrOH = 90:10, flow rate 1.0 mL/min, λ = 254 nm); ^{1}H NMR (400 MHz, $CDCl_3$) δ ppm 7.93 - 7.91 (m, 2H), 7.57 - 7.52 (m, 1H), 7.46 - 7.42 (m, 2H), 7.15 (d, *J* = 8.2 Hz, 2H), 7.09 (d, *J* = 7.9 Hz, 2H), 3.85 (quin, *J* = 7.3 Hz, 1H), 3.59 (s, 3H), 3.38 (dd, *J* = 16.7, 7.0 Hz, 1H), 3.31 (dd, *J* = 16.7, 7.0 Hz, 1H), 2.80 (dd, *J* = 15.6, 7.0 Hz, 1H), 2.67 (dd, *J* = 15.6, 7.6 Hz, 1H), 2.30 (s, 3H). ^{13}C NMR (75.5 MHz, $CDCl_3$) δ ppm 198.2, 172.4, 140.3, 136.9, 136.3, 133.0, 129.3, 128.5, 128.1, 127.1, 51.5, 44.6, 40.6, 37.1, 20.99. IR ν_{max}: 3010, 2948, 2897, 1724, 1675, 1597, 1580, 1516, 1436, 1351, 1263, 1218, 1149, 1067, 995, 976, 883, 817, 760, 722, 702, 666, 558 cm^{-1}. mp 85.2 - 86.6 °C. HRMS (ESI-TOF) m/z: $[MNa]^+$ calcd for $C_{19}H_{20}O_3Na$ 319.1310 found: 319.1307.

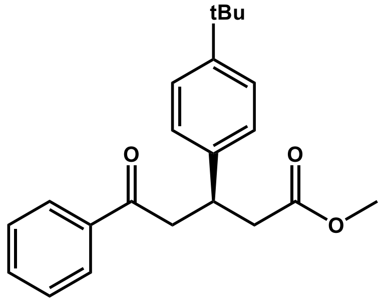

methyl (S)-3-(4-(tert-butyl)phenyl)-5-oxo-5-phenylpentanoate (**4v**). GP3. A scale of 0.2 mmol, beige solid, 48.14 mg (0.14 mmol), isolated yield of 71% (flash column chromatography AcOEt/hex gradient 0% → 20 % AcOEt); *er* 93:7 (Phenomenex Lux Amylose-1 3μm column, n–hexane/i–PrOH = 90:10, flow rate 1.0 mL/min, λ = 254 nm); ^{1}H NMR (400 MHz, $CDCl_3$) δ ppm 7.93 - 7.91 (m, 2H), 7.56 - 7.52 (m, 1H), 7.45 - 7.41 (m, 2H), 7.30 (d, *J* = 8.2 Hz, 2H), 7.19 (d, *J* = 8.2 Hz, 2H), 3.86 (quin, *J* = 7.0 Hz, 1H), 3.59 (s, 3H), 3.41 - 3.30 (m, 2H), 2.80 (dd, *J* = 15.3, 7.2 Hz, 1H), 2.69 (dd, *J* = 15.3, 7.5 Hz, 1H), 1.29 (s, 9H). ^{13}C NMR (75.5 MHz, $CDCl_3$) δ ppm 198.3, 172.4, 149.4, 140.3, 136.9, 133.0, 128.5, 128.0, 126.9, 125.5, 51.5, 44.6, 40.5, 36.9, 34.3, 31.3. IR $ν_{max}$: 2971, 2949, 2906, 2870, 1725, 1682, 1596, 1510, 1448, 1361, 1259, 1118, 1064, 1018, 977, 854, 830, 755, 690, 586 cm^{-1}. mp 93.1 - 95.2 °C. HRMS (ESI-TOF) m/z: $[MNa]^+$ calcd for $C_{22}H_{26}O_3Na$ 361.1780 found: 361.1785.

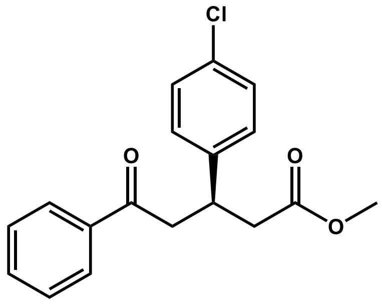

methyl (S)-3-(4-chlorophenyl)-5-oxo-5-phenylpentanoate (**4w**). GP3. A scale of 0.2 mmol, yellow solid, 42.61 mg (0.13 mmol), isolated yield of 67% (flash column chromatography AcOEt/hex gradient 0% → 20 % AcOEt); *er* 95:5 (Phenomenex Lux Amylose-1 3μm column, n–hexane/i–PrOH = 90:10, flow rate 1.0 mL/min, λ = 254 nm); ^{1}H NMR (400 MHz, $CDCl_3$) δ ppm 7.92 - 7.89 (m, 2H), 7.57 - 7.53 (m, 1H), 7.46 - 7.42 (m, 2H), 7.26 - 7.19 (m, 4H), 3.87 (quin, *J* = 7.2 Hz, 1H), 3.59 (s, 3H), 3.38 (dd, *J* = 17.0, 6.9 Hz, 1H), 3.31 (dd, *J* = 17.0, 7.2 Hz, 1H), 2.80 (dd, *J* = 15.6, 6.8 Hz, 1H), 2.66 (dd, *J* = 15.6, 7.9 Hz, 1H). ^{13}C NMR (75.5 MHz, $CDCl_3$) δ ppm 197.7, 172.0, 141.8, 136.7, 133.2, 132.5, 128.7, 128.7, 128.6, 128.0, 51.6, 44.3, 40.4, 36.8. IR $ν_{max}$: 3069, 2949, 2898, 2848, 1731, 1682, 1595, 1490, 1440, 1417, 1368, 1305, 1270, 1251, 1182, 1153, 1088, 1049, 1012, 1002, 986, 932, 855, 775, 722, 686, 608, 568, 547, 471, 448 cm^{-1}. mp 68.5 - 71.0 °C. HRMS (ESI-TOF) m/z: $[MNa]^+$ calcd for $C_{18}H_{17}O_3NaCl$ 339.0764 found: 339.0768.

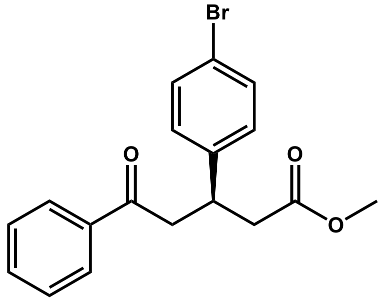

methyl (S)-3-(4-bromophenyl)-5-oxo-5-phenylpentanoate (**4x**). GP3. A scale of 0.2 mmol, white solid, 37.70 mg (0.10 mmol), isolated yield of 52% (flash column chromatography AcOEt/hex gradient 0% → 20 % AcOEt); *er* 95:5 (Phenomenex Lux Amylose-1 3μm column, n–hexane/i–PrOH = 90:10, flow rate 1.0 mL/min, λ = 254 nm); ^{1}H NMR (400 MHz, $CDCl_3$) δ ppm 7.92 - 7.89 (m, 2H), 7.58 - 7.53 (m, 1H), 7.47 - 7.39 (m, 4H), 7.15 (d, *J* = 8.2 Hz, 2H), 3.86 (quin, *J* = 7.2 Hz, 1H), 3.60 (s, 3H), 3.38 (dd, *J* = 17.3, 6.9 Hz, 1H), 3.30 (dd, *J* = 17.0, 7.3 Hz, 1H), 2.80 (dd, *J* = 15.6, 6.8 Hz, 1H), 2.66 (dd, *J* = 15.6, 8.1 Hz, 1H). ^{13}C NMR (75.5 MHz, $CDCl_3$) δ ppm 197.7, 172.0, 142.3, 136.7, 133.2, 131.7, 129.1, 128.6, 128.0, 120.6, 51.6, 44.2, 40.3, 36.9. IR ν_{max} : 3060, 3027, 2950, 1732, 1682, 1596, 1488, 1448, 1435, 1410, 1361, 1266, 1213, 1176, 1151, 1073, 1009, 960, 929, 822, 751, 689, 541 cm^{-1}. mp 76.0 - 79.3 °C. HRMS (ESI-TOF) m/z: $[MNa]^+$ calcd for $C_{18}H_{17}O_3NaBr$ 383.0259 found: 383.0262.

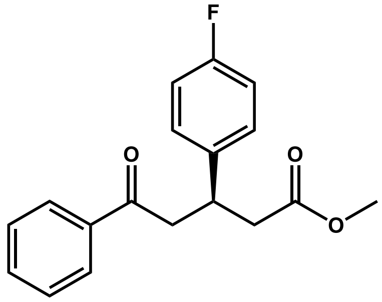

methyl (S)-3-(4-fluorophenyl)-5-oxo-5-phenylpentanoate (**4y**). GP3. A scale of 0.2 mmol, brown solid, 35.5 mg (0.12 mmol), isolated yield of 59% (flash column chromatography AcOEt/hex gradient 0% → 20 % AcOEt); *er* 95:5 (Phenomenex Lux Amylose-1 3μm column, n–hexane/i–PrOH = 90:10, flow rate 1.0 mL/min, λ = 254 nm); ^{1}H NMR (400 MHz, $CDCl_3$) δ ppm 7.92 - 7.90 (m, 2H), 7.57 - 7.53 (m, 1H), 7.46 - 7.42 (m, 2H), 7.25 - 7.21 (m, 2H), 7.00 - 6.95 (m, 2H), 3.88 (quin, *J* = 7.3 Hz, 1H), 3.59 (s, 3H), 3.38 (dd, *J* = 17.0, 6.8 Hz, 1H), 3.30 (dd, *J* = 17.0, 7.2 Hz, 1H), 2.80 (dd, *J* = 15.3, 6.9 Hz, 1H), 2.66 (dd, *J* = 15.6, 7.9 Hz, 1H). ^{13}C NMR (75.5 MHz, $CDCl_3$) δ ppm 197.9, 172.1, 161.6 (d, *J* = 245.0 Hz), 138.9 (d, *J* = 2.8 Hz), 136.8, 133.2, 128.82 (d, *J* = 8.0 Hz), 128.6, 128.0, 115.4 (d, *J* = 21.1 Hz), 51.6, 44.5, 40.6, 36.8. IR ν_{max}: 2950, 1738, 1677, 1596, 1508, 1439, 1362, 1266, 1217, 1153, 1099, 1068, 996, 884, 836, 755, 706, 683, 554 cm^{-1}. mp 66.3 - 69.9 °C. HRMS (ESI-TOF) m/z: $[MNa]^+$ calcd for $C_{18}H_{17}O_3FNa$ 323.1059 found: 323.1068.

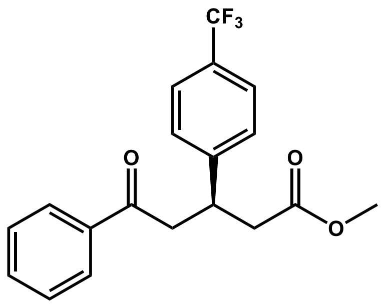

methyl (S)-5-oxo-5-phenyl-3-(4-trifluoromethyl)phenyl)pentanoate (**4z**). GP3. A scale of 0.2 mmol, yellow solid, 40.11 mg (0.11 mmol), isolated yield of 57% (flash column chromatography AcOEt/hex gradient 0% → 20 % AcOEt); *er* 95:5 (Phenomenex Lux Amylose-1 3µm column, n–hexane/i–PrOH = 90:10, flow rate 1.0 mL/min, λ = 254 nm); ^{1}H NMR (400 MHz, $CDCl_3$) δ ppm 7.92 - 7.90 (m, 2H), 7.57 - 7.54 (m, 3H), 7.46 - 7.39 (m, 4H), 3.96 (quin, *J* = 7.2 Hz, 1H), 3.60 (s, 3H), 3.43 (dd, *J* = 17.3, 6.8 Hz, 1H), 3.36 (dd, *J* = 17.3, 7.3 Hz, 1H), 2.84 (dd, *J* = 15.6, 6.9 Hz, 1H), 2.71 (dd, *J* = 15.9, 7.9 Hz, 1H). ^{13}C NMR (101 MHz, $CDCl_3$) δ ppm 197.5, 171.9, 147.5, 136.7, 133.3, 129.1 (q, *J* = 32.4 Hz), 128.7, 128.0, 127.8, 125.6 (q, *J* = 3.9 Hz), 124.1 (q, *J* = 272.0 Hz), 51.7, 44.1, 40.2, 37.2. IR v_{max}: 2953, 1724, 1678, 1618, 1599, 1439, 1328, 1264, 1219, 1151, 1122, 1109, 1073, 1018, 857, 837, 759, 747, 735, 713, 689, 631, 608, 547 cm^{-1}. mp 83.3 - 84.9 °C. HRMS (ESI-TOF) m/z: $[MNa]^+$ calcd for $C_{19}H_{17}O_3NaF_3$ 373.1027 found: 373.1029.

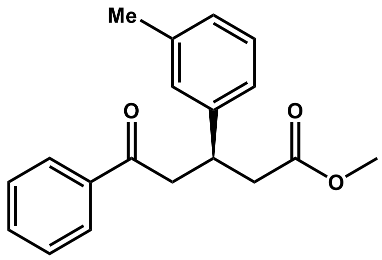

methyl (S)-5-oxo-5-phenyl-3-(m-tolyl)pentanoate (**4aa**). GP3. A scale of 0.2 mmol, yellow oil, 35.30 mg (0.12 mmol), isolated yield of 60% (flash column chromatography AcOEt/hex gradient 0% → 20 % AcOEt); *er* 92:8 (Phenomenex Lux Cellulose-1 3µm column, n–hexane/i–PrOH = 90:10, flow rate 1.0 mL/min, λ = 254 nm); ^{1}H NMR (400 MHz, $CDCl_3$) δ ppm 7.94 - 7.91 (m, 2H), 7.56 - 7.52 (m, 1H), 7.46 - 7.41 (m, 2H), 7.18 (t, *J* = 7.5 Hz, 1H), 7.07 - 7.05 (m, 2H), 7.03 - 7.00 (m, 1H), 3.85 (quin, *J* = 7.2 Hz, 1H), 3.59 (s, 3H), 3.39 (dd, *J* = 17.0, 7.2 Hz, 1H), 3.32 (dd, *J* = 17.0, 6.9 Hz, 1H), 2.81 (dd, *J* = 15.3, 7.2 Hz, 1H), 2.69 (dd, *J* = 15.3, 7.8, 1H), 2.32 (s, 3H). ^{13}C NMR (101 MHz, $CDCl_3$) δ ppm 198.2, 172.4, 143.3, 138.1, 137.0, 133.1, 128.6, 128.5, 128.1, 128.1, 127.6, 124.2, 51.5, 44.6, 40.6, 37.4, 21.5. IR v_{max}: 3025, 2950, 2920, 1732, 1682, 1597, 1447, 1436, 1361, 1266, 1210, 1159, 1001, 883, 784, 753, 689, 444 cm^{-1}. HRMS (ESI-TOF) m/z: $[MNa]^+$ calcd for $C_{19}H_{20}O_3Na$ 319.1310 found: 319.1312.

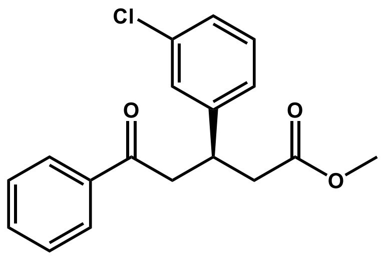

methyl (S)-3-(3-chlorophenyl)-5-oxo-5-phenylpentanoate (**4ab**). GP3. A scale of 0.2 mmol, white solid, 38.00 mg (0.12 mmol), isolated yield of 60% (flash column chromatography AcOEt/hex gradient 0% → 20 % AcOEt); *er* 95:5 (Phenomenex Lux Amylose-1 3μm column, n–hexane/i–PrOH = 90:10, flow rate 1.0 mL/min, λ = 254 nm); ^{1}H NMR (400 MHz, $CDCl_3$) δ ppm 7.93 - 7.90 (m, 2H), 7.57 - 7.53 (m, 1H), 7.46 - 7.42 (m, 2H), 7.26 - 7.25 (m, 1H), 7.23 - 7.15 (m, 3H), 3.87 (quin, *J* = 7.2 Hz, 1H), 3.60 (s, 3H), 3.39 (dd, *J* = 17.0, 7.0 Hz, 1H), 3.32 (dd, *J* = 17.0, 7.0 Hz, 1H), 2.80 (dd, *J* = 15.6, 6.9 Hz, 1H), 2.67 (dd, *J* = 15.6, 7.9 Hz, 1H). ^{13}C NMR (101 MHz, $CDCl_3$) δ ppm 197.7, 172.0, 145.5, 136.7, 134.4, 133.3, 129.9, 128.6, 128.0, 127.5, 127.0, 125.8, 51.7, 44.2, 40.3, 37.1. IR ν_{max}: 2906, 2844, 1720, 1675, 1594, 1571, 1420, 1261, 1216, 1149, 1081, 976, 878, 821, 800, 755, 687, 618, 595, 509, 468 cm^{-1}. mp 52.4 - 54.4 °C. HRMS (ESI-TOF) m/z: $[MNa]^+$ calcd for $C_{18}H_{17}O_3NaCl$ 339.0764 found: 339.0762.

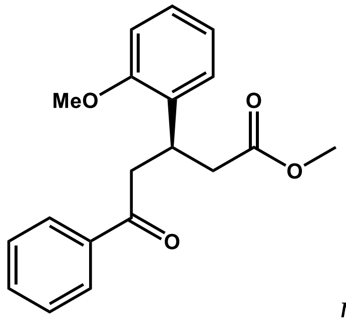

methyl (S)-3-(2-methoxyphenyl)-5-oxo-5-phenylpentanoate (**4ac**). GP3. A scale of 0.2 mmol, colorless oil, 42.70 mg (0.14 mmol), isolated yield of 68% (flash column chromatography AcOEt/hex gradient 0% → 20 % AcOEt); *er* 91:9 (Phenomenex Lux Cellulose-1 3μm column, n–hexane/i–PrOH = 80:20, flow rate 1.0 mL/min, λ = 254 nm); ^{1}H NMR (400 MHz, $CDCl_3$) δ ppm 7.97 - 7.94 (m, 2H), 7.55 - 7. 51 (m, 1H), 7.46 - 7.41 (m, 2H), 7.22 - 7.16 (m, 2H), 6.88 (td, *J* = 7.3, 1.2 Hz, 1H), 6.84 (d, *J* = 7.9 Hz, 1H), 4.14 (quin, *J* = 7.0 Hz, 1H), 3.81 (s, 3H), 3.58 (s, 3H), 3.41 - 3.40 (m, 2H), 2.86 (dd, *J* = 15.6, 7.3 Hz, 1H), 2.80 (dd, *J* = 15.6, 7.3 Hz, 1H). ^{13}C NMR (101 MHz, $CDCl_3$) δ ppm 198.9, 172.8, 157.2, 137.1, 132.9, 131.0, 128.5, 128.5, 128.1, 127.8, 120.6, 110.8, 55.3, 51.4, 42.9, 38.4, 33.3. IR ν_{max}: 3062, 3000, 2950, 2838, 1732, 1681, 1598, 1493, 1437, 1240, 1116, 1074, 1026, 751, 690, 501 cm^{-1}. HRMS (ESI-TOF) m/z: $[MNa]^+$ calcd for $C_{19}H_{20}O_4Na$ 335.1259 found: 335.1263.

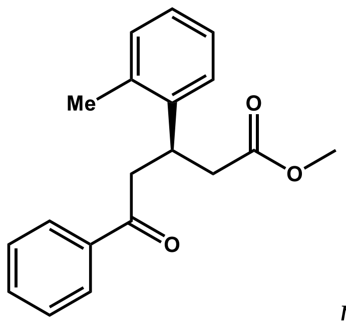

methyl (S)-5-oxo-5-phenyl-3-(o-tolyl)pentanoate (**4ad**). GP3. A scale of 0.2 mmol, green oil, 20.93 mg (0.071 mmol), isolated yield of 35% (flash column chromatography AcOEt/hex gradient 0% → 20 % AcOEt); *er* 97:3 (Phenomenex Lux Amylose-1 3μm column, n–hexane/i–PrOH = 90:10, flow rate 1.0 mL/min, λ = 254 nm); ^{1}H NMR (400 MHz, $CDCl_3$) δ ppm 7.93 - 7.90 (m, 2H), 7.59 - 7.54 (m, 1H), 7.48 - 7.43 (m, 2H), 7.23 - 7.15 (m, 3H), 7.13 - 7.10 (m, 1H), 4.18 (quin, *J* = 7.2 Hz, 1H), 3.60 (s, 3H), 3.43 - 3.31 (m, 2H), 2.80 (dd, *J* = 15.6, 7.2 Hz, 1H), 2.70 (dd, *J* = 15.3, 7.6 Hz, 1H), 2.45 (s, 3H). ^{13}C NMR (101 MHz, $CDCl_3$) δ ppm 198.3, 172.4, 141.6, 136.9, 136.0, 133.1, 130.7, 128.6, 128.0, 126.5, 126.3, 125.4, 51.5, 44.3, 40.2, 32.4, 19.6. IR v_{max}: 3061, 3021, 2951, 1732, 1682, 1597, 1491, 1447, 1435, 1360, 1267, 1206, 1153, 986, 888, 752, 727, 689, 561, 456 cm^{-1}. HRMS (ESI-TOF) m/z: $[MNa]^+$ calcd for $C_{19}H_{20}O_3Na$ 319.1310 found: 319.1309.

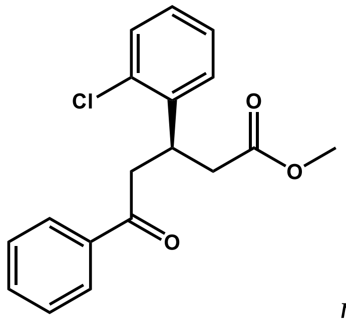

methyl (S)-3-(2-chlorophenyl)-5-oxo-5-phenylpentanoate (**4ae**). GP3. A scale of 0.2 mmol, colorless oil, 42.00 mg (0.13 mmol), isolated yield of 66% (flash column chromatography AcOEt/hex gradient 0% → 20 % AcOEt); *er* 97:3 (Phenomenex Lux Cellulose-1 3μm column, n–hexane/i–PrOH = 90:10, flow rate 1.0 mL/min, λ = 254 nm); ^{1}H NMR (400 MHz, $CDCl_3$) δ ppm 7.96 - 7.93 (m, 2H), 7.57 - 7.53 (m, 1H), 7.46 - 7.42 (m, 2H), 7.36 (dd, *J* = 7.6, 1.5 Hz, 1H), 7.30 (dd, *J* = 7.6, 1.8 Hz, 1H), 7.21 (td, *J* = 7.3, 1.5 Hz, 1H), 7.14 (td, *J* = 7.3, 1.8, 1H), 4.37 (quin, *J* = 7.0 Hz, 1H), 3.59 (s, 3H), 3.43 (dd, *J* = 7.0, 1.2 Hz, 2H), 2.87 (dd, *J* = 15.9, 7.0 Hz, 1H), 2.79 (dd, *J* = 15.6, 7.5 Hz, 1H). ^{13}C NMR (101 MHz, $CDCl_3$) δ ppm 197.9, 172.1, 140.4, 136.8, 133.7, 133.2, 130.1, 128.6, 128.1, 127.9, 127.0, 51.6, 42.8, 38.5, 34.1. IR v_{max}: 3061, 2951, 1732, 1682, 1597, 1580, 1476, 1436, 1362, 1267, 1209, 1153, 1054, 1035, 1001, 988, 890, 845, 750, 689, 554, 459 cm^{-1}. HRMS (ESI-TOF) m/z: $[MNa]^+$ calcd for $C_{18}H_{17}O_3NaCl$ 339.0764 found: 339.0769.

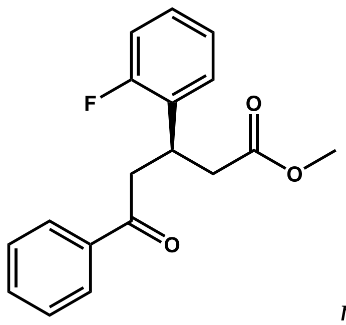

methyl (S)-3-(2-fluorophenyl)-5-oxo-5-phenylpentanoate (**4af**). GP3. A scale of 0.2 mmol, colorless oil, 17.74 mg (0.06 mmol), isolated yield of 30% (flash column chromatography AcOEt/hex gradient 0% → 20 % AcOEt); *er* 96:4 (Phenomenex Lux Cellulose-1 3μm column, n–hexane/i–PrOH = 90:10, flow rate 1.0 mL/min, λ = 254 nm); ^{1}H NMR (400 MHz, $CDCl_3$) δ ppm 7.94 - 7.92 (m, 2H), 7.57 - 7.52 (m, 1H), 7.46 - 7.41 (m, 2H), 7.29 (td, *J* = 7.6, 1.8 Hz, 1H), 7.21 - 7.16 (m, 1H), 7.07 (td, *J* = 7.6, 1.2 Hz, 1H), 7.01 (ddd, *J* = 11.2, 8.2, 1.2 Hz, 1H), 4.08 (quin, *J* = 7.2 Hz, 1H), 3.59 (s, 3H), 3.44 (d, *J* = 7.0 Hz, 2H), 2.87 (dd, *J* = 15.6, 7.0 Hz, 1H), 2.78 (dd, *J* = 15.6, 7.9 Hz, 1H). ^{13}C NMR (101 MHz, $CDCl_3$) δ ppm 198.0, 172.2, 161.0 (d, *J* = 245.8 Hz), 136.8, 133.1, 129.8 (d, *J* = 13.9 Hz), 129.7 (d, *J* = 5.4 Hz), 128.6, 128.4 (d, *J* = 8.5 Hz), 128.1, 124.2 (d, *J* = 3.9 Hz), 115.8 (d, *J* = 22.4 Hz), 51.6, 42.8 (d, *J* = 1.5 Hz), 38.8 (d, *J* = 1.5 Hz), 32.8. IR ν_{max}: 3063, 2952, 1733, 1682, 1581, 1492, 1448, 1364, 1212, 1174, 1154, 1103, 890, 829, 752, 689, 616, 565, 503 cm^{-1}. HRMS (ESI-TOF) m/z: $[MNa]^+$ calcd for $C_{18}H_{17}O_3NaF$ 323.1059 found: 323.1062.

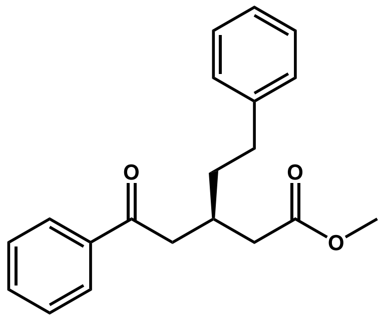

methyl (S)-5-oxo-3-phenethyl-5-phenylpentanoate (**4ag**). GP3. A scale of 0.2 mmol, yellow oil, 29.50 mg (0.095 mmol), isolated yield of 48% (flash column chromatography AcOEt/hex gradient 0% → 20 % AcOEt); *er* 87:13 (Phenomenex Lux Cellulose-1 3μm column, n–hexane/i–PrOH = 90:10, flow rate 1.0 mL/min, λ = 254 nm); ^{1}H NMR (400 MHz, $CDCl_3$) δ ppm 7.97 - 7.94 (m, 2H), 7.58 - 7.54 (m, 1H), 7.48 - 7.44 (m, 2H), 7.28 - 7.24 (m, 2H), 7.19 - 7.15 (m, 3H), 3.66 (s, 3H), 3.15 (dd, *J* = 16.7, 7.0 Hz, 1H), 3.02 (dd, *J* = 16.7, 6.5 Hz, 1H), 2.70 - 2.61 (m, 3H), 2.50 - 2.48 (m, 2H), 1.80 - 1.73 (m, 2H). ^{13}C NMR (101 MHz, $CDCl_3$) δ ppm 199.3, 173.1, 141.8, 137.1, 133.1, 128.6, 128.4, 128.3, 128.1, 125.9, 51.5, 42.6, 38.3, 36.0, 33.2, 31.3. IR ν_{max}: 3061, 3026, 2949, 2859, 1731, 1682, 1597, 1495, 1448, 1436, 1369, 1256, 1212, 1156, 1002, 890, 749, 690, 573, 485 cm^{-1}. HRMS (ESI-TOF) m/z: $[MNa]^+$ calcd for $C_{20}H_{22}O_3Na$ 333.1467 found: 333.1471.

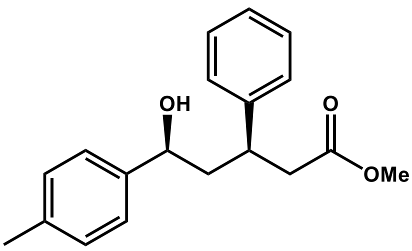

methyl (3R,5S)-5-hydroxy-3,5-diphenylpentanoate (**5a**). A round bottom flask was charged with **4f** (0.13 mmol; 40 mg) and dissolved in dichloromethane (1 mL) and methanol (0.5 mL). The solution was cooled to 0°C and $NaBH_4$ (0.19 mmol; 7.3 mg) was added. The reaction was carried out at room temperature for 1h and quenched with HCl 1M. The phases were separated and the water phase was extracted with ethyl acetate (3x). The combined organic extracts were dried with anhydrous $MgSO_4$. After evaporation of solvents and purification by flash column chromatography (AcOEt/hex grandient 0% → 20 % AcOEt) the pure product **5a** was obtained as a colorless oil (0.058 mmol; 17.38 mg; isolated yield of 43%). *dr* > 20:1. ^1H NMR (400 MHz, $CDCl_3$) δ ppm 7.35 - 7.18 (m, 9H), 4.46 (t, *J* = 7.0 Hz, 1H), 3.57 (s, 3H), 3.10 - 3.03 (m, 1H), 2.70 (dd, *J* = 15.6, 7.0 Hz, 1H), 2.60 (dd, *J* = 15.3 Hz, 7.8 Hz, 1H), 2.38 (s, 3H), 2.23 (ddd, *J* = 13.5, 9.1, 6.8 Hz, 1H), 2.10 (ddd, *J* = 13.5, 7.3, 5.9 Hz, 1H). ^{13}C NMR (101 MHz, $CDCl_3$) δ ppm 172.7, 143.5, 140.7, 137.6, 129.2, 128.6, 127.5, 126.7, 126.3, 72.4, 51.5, 44.8, 41.5, 38.8, 21.1. IR v_{max}: 3413, 3027, 2946, 2924, 1733, 1436, 1191, 1148, 1054, 1017, 819, 763, 699, 616, 549, 536 cm^{-1}. HRMS (ESI-TOF) m/z: $[MNa]^+$ calcd for $C_{19}H_{22}NaO_3$ 321.1467 found: 321.1467.

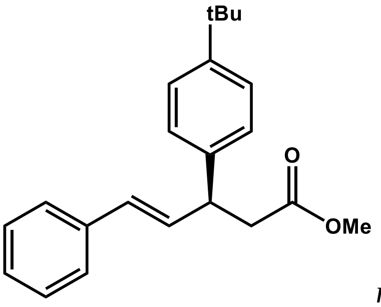

methyl (S,E)-3-phenyl-5-(p-tolyl)pent-4-enoate (**5b**). A round bottom flask was charged with **4v** (0.135 mmol; 40 mg) and dissolved in dichloromethane (1 mL) and methanol (0.5 mL). The solution was cooled to 0°C and $NaBH_4$ (0.19 mmol; 7.3 mg) was added. The reaction was carried out at room temperature for 1h and after this time pTSA·H_2O (0.193 mmol; 37 mg) was added. The solvent was evaporated, the residue was dissolved in $CHCl_3$ (2 mL) and another portion of pTSA·H_2O (0.135 mmol; 26 mg) was added. The reaction was carried out at boiling point for 24 h. The mixture was cooled to room temperature followed by the addition of water. The phases were separated and the water phase was extracted with chloroform (2x). The organic phase was washed with water and brine. After drying with $MgSO_4$ and evaporation of the solvent the crude was purified by flash column chromatography (EtOAc/hex grandient 0% → 20 % EtOAc) to give the final product **5b** as a colorless oil (0.043 mmol; 12 mg; isolated yield of 32%). *er* 93:7 (Phenomenex Lux Cellulose-1 3µm column, n–hexane/i–PrOH = 95:5, flow rate 0.5 mL/min, λ = 254 nm); ^1H NMR (700 MHz, $CDCl_3$) δ ppm 7.37 - 7.35 (m, 4H), 7.31 - 7.29 (m, 2H), 7.22 (d, *J* = 8.0 Hz, 3H), 6.47 (d, *J* = 16.0 Hz, 1H), 6.35 (dd, *J* = 15.5, 7.7 Hz, 1H), 4.04 (q, *J* = 7.5 Hz, 1H), 3.66 (s, 3H), 2.87 (dd, *J* = 15.5, 8.5 Hz, 1H), 2.83 (dd, *J* = 14.9, 6.9 Hz, 1H), 1.33 (s, 9H). ^{13}C

NMR (176 MHz, $CDCl_3$) δ ppm 172.3, 149.6, 139.6, 137.2, 132.1, 130.0, 128.5, 127.3, 127.1, 126.3, 125.6, 51.6, 44.6, 40.6, 34.4, 31.4. IR v_{max}: 2953, 2916, 2849, 1740, 1471, 1463, 1389, 1365, 1159, 964, 829, 750, 720, 693, 574 cm^{-1}. HRMS (ESI-TOF) m/z: $[MNa]^+$ calcd for $C_{22}H_{26}NaO_2$ 345.1830 found: 345.1831.

4. X-Ray Crystallography Data

Sample preparation: A single crystal of **4g** was obtained by slow evaporation from EtOAc/hex solution.

Crystal structure determination: Single-crystal X-ray diffraction data for **4g** were collected on an XtaLAB Synergy-S Dualflex diffractometer equipped with monochromated CuKα radiation (λ = 1.54184 Å). The crystal was coated with Paratone-N oil and mounted on a loop. Data collection was carried out at 100(2) K to minimize solvent loss, possible structural disorder and thermal motion effects. Data frames were processed (unit cell determination, intensity data integration, correction for Lorentz and polarisation effects, and empirical absorption correction) by using the corresponding diffractometer's software package.[8] The structure was solved by using direct method with SHELXS-2018/3[9] and refined by using full-matrix least-squares method based on F^2 by using SHELXL-2018/3.[10] The programs Mercury[11] and POV-Ray[12] were both used to prepare molecular graphics. All non-hydrogen atoms were refined anisotropically. All hydrogen atoms were positioned geometrically with C-H = 0.95 Å (aromatic), 0.98 (methyl), 0.99 Å (methylene), 1.00 (methanetriyl) and refined as riding, with Uiso(H) = 1.2 Ueq (C) or 1.5 Ueq (C) for methyl groups.

A summary of the data collection and structure refinement parameters are provided in Table S1.

Table S1. Crystal data and details of the refinement parameters for **4g**.

Compound reference	**4g**
Chemical formula	$C_{18}H_{17}BrO_3$
Formula Mass	361.22
Crystal system	Orthorhombic
Space group	*P*212121
a/Å	5.85650(10)
b/Å	14.32460(10)
c/Å	74.8922(8)
α/°	90
β/°	90
γ/°	90
Unit cell volume/Å^3	6282.86(13)
Temperature/K	100(2)
No. of formula units per unit cell, *Z*	16
Radiation type	CuKα
No. of reflections measured	68230
No. of independent reflections	13018
R_{int}	0.0464
Final $R1^a$ values ($I > 2\sigma(I)$)	0.0277
Final $wR(F^2)^b$ values ($I > 2\sigma(I)$)	0.0598
Final $R1^a$ values (all data)	0.0295
Final $wR(F^2)^b$ values (all data)	0.0605
Goodness of fit on F^2	1.064
Flack parameter	-0.025(6)

$^aR_1 = \sum\|F_o| - |F_c\|/\sum|F_o|$.

$^b wR_2 = \{\sum[w(F_o^2 - F_c^2)^2]/\sum[w(F_o^2)^2]\}^{1/2}$.

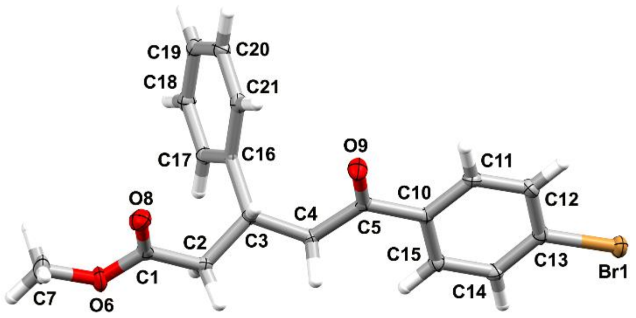

Figure S1. Molecular structure of **4g**, atomic displacement plot shown with 50% probability.

5. NMR Spectra

^{1}H NMR 400 MHz $CDCl_3$

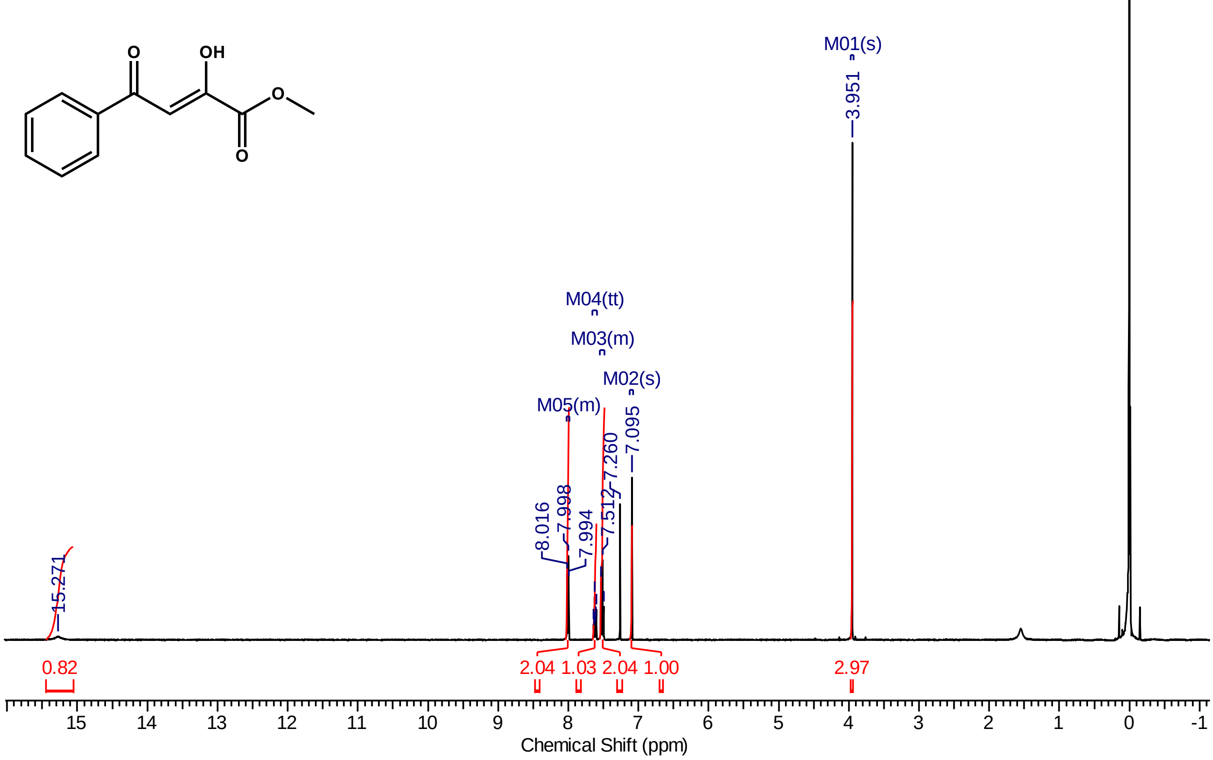

$^{13}C\{^{1}H\}$ NMR 176 MHz $CDCl_3$

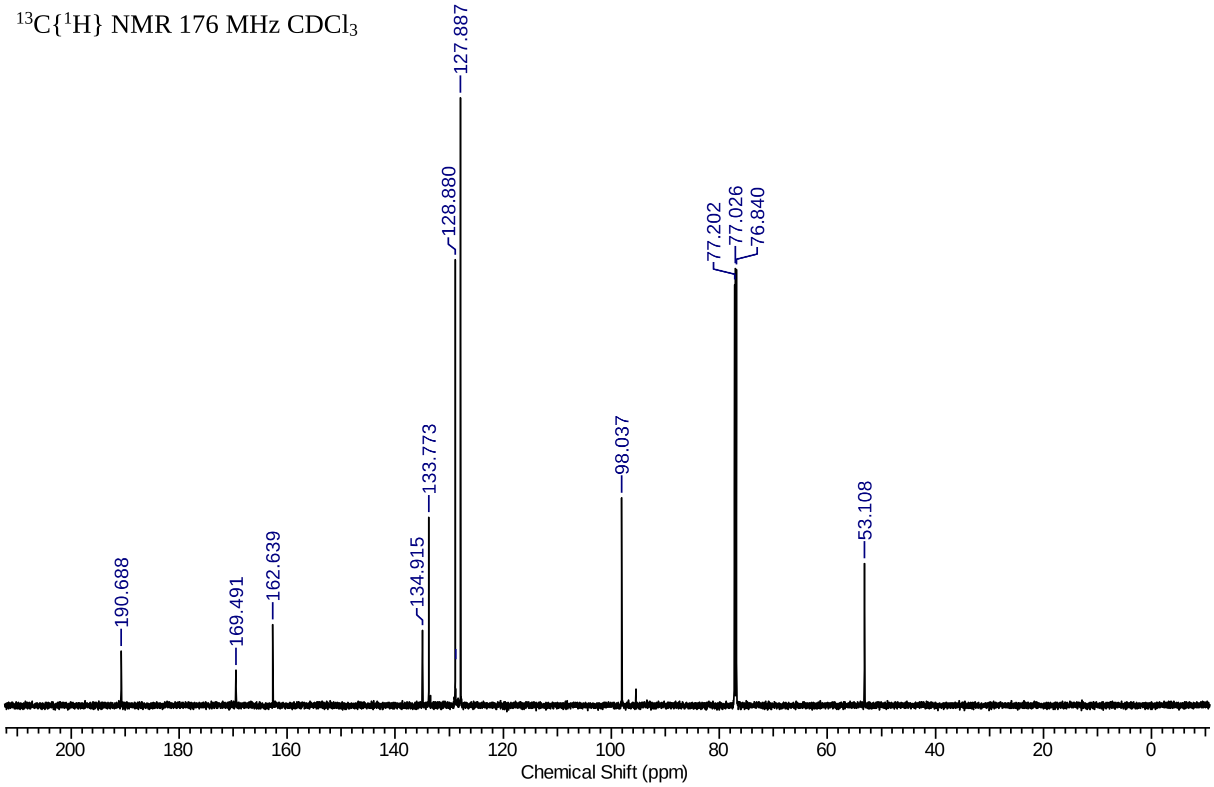

Figure S2. ^{1}H and ^{13}C NMR spectra of compound **1a**.

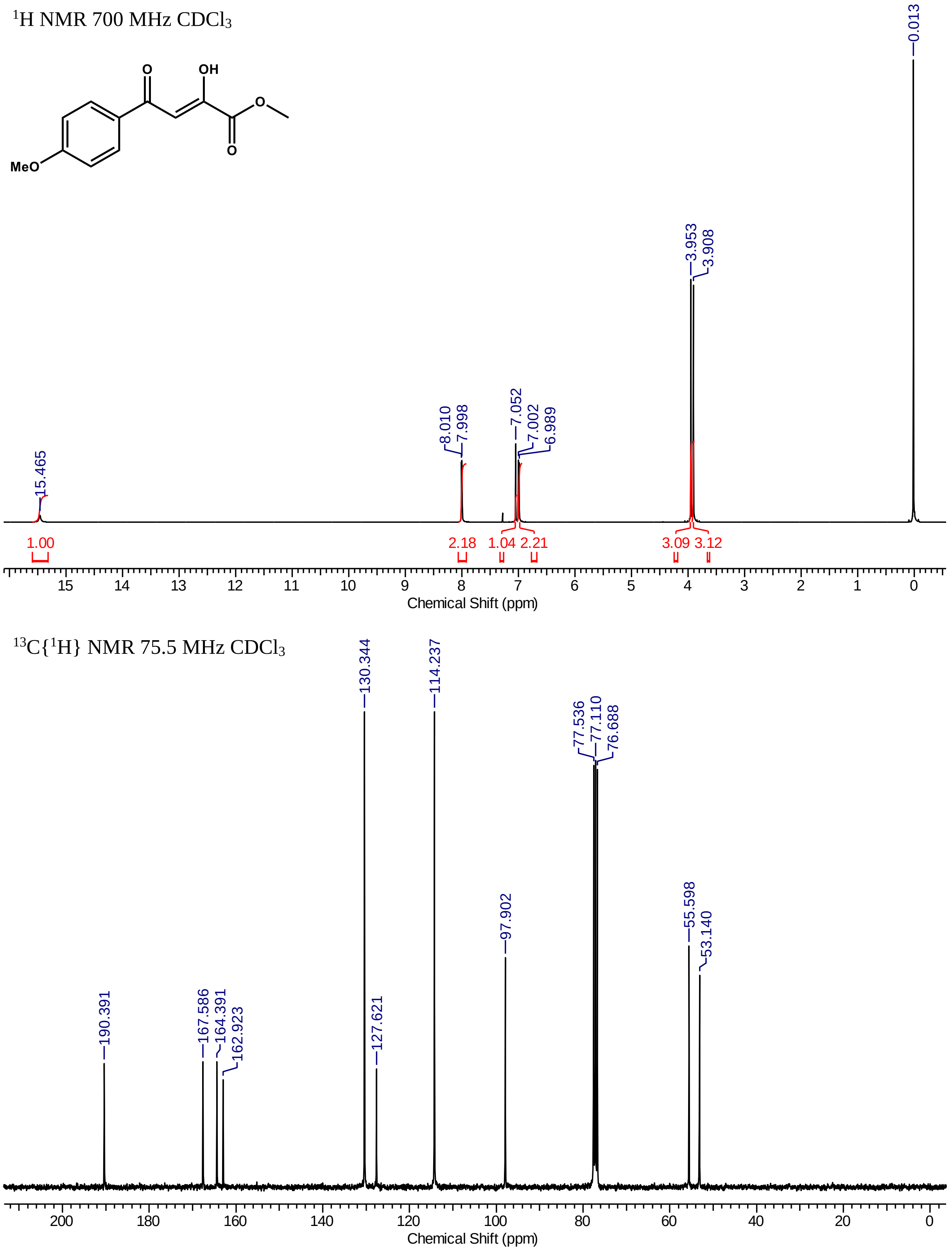

Figure S3. ^{1}H and ^{13}C NMR spectra of compound **1b**.

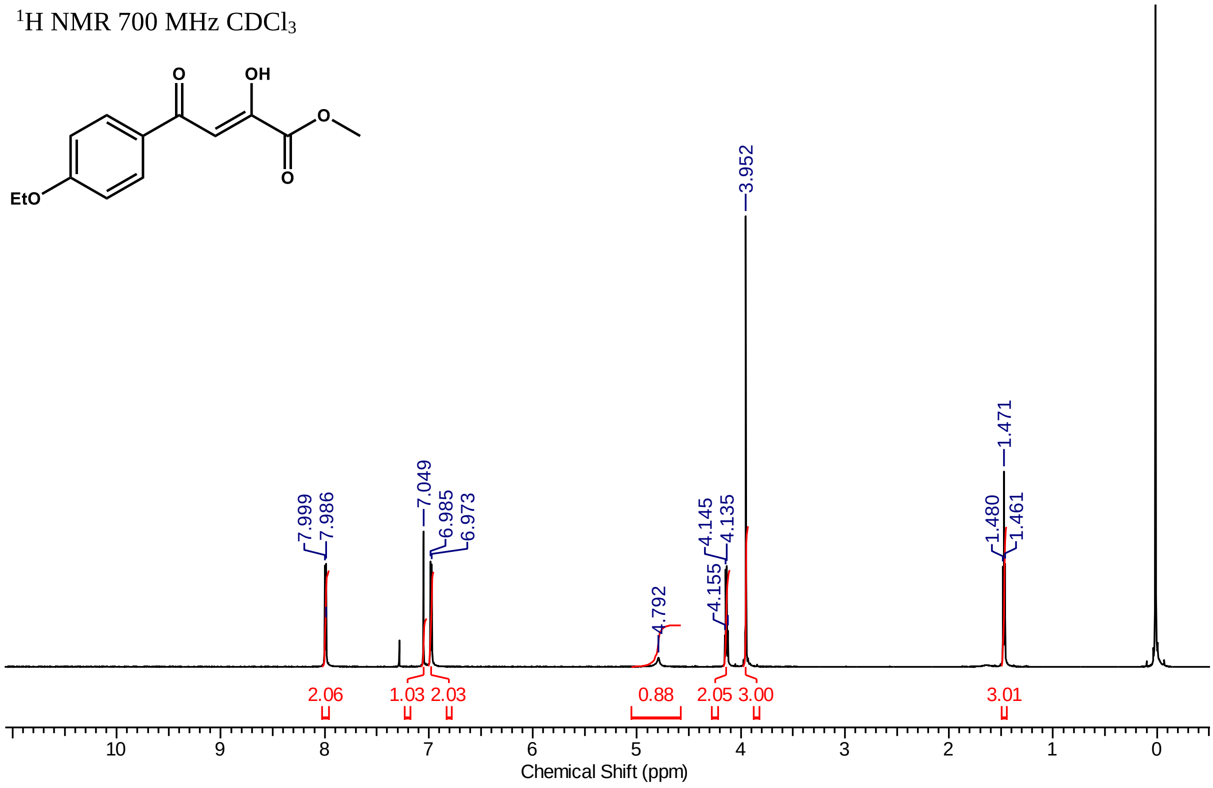

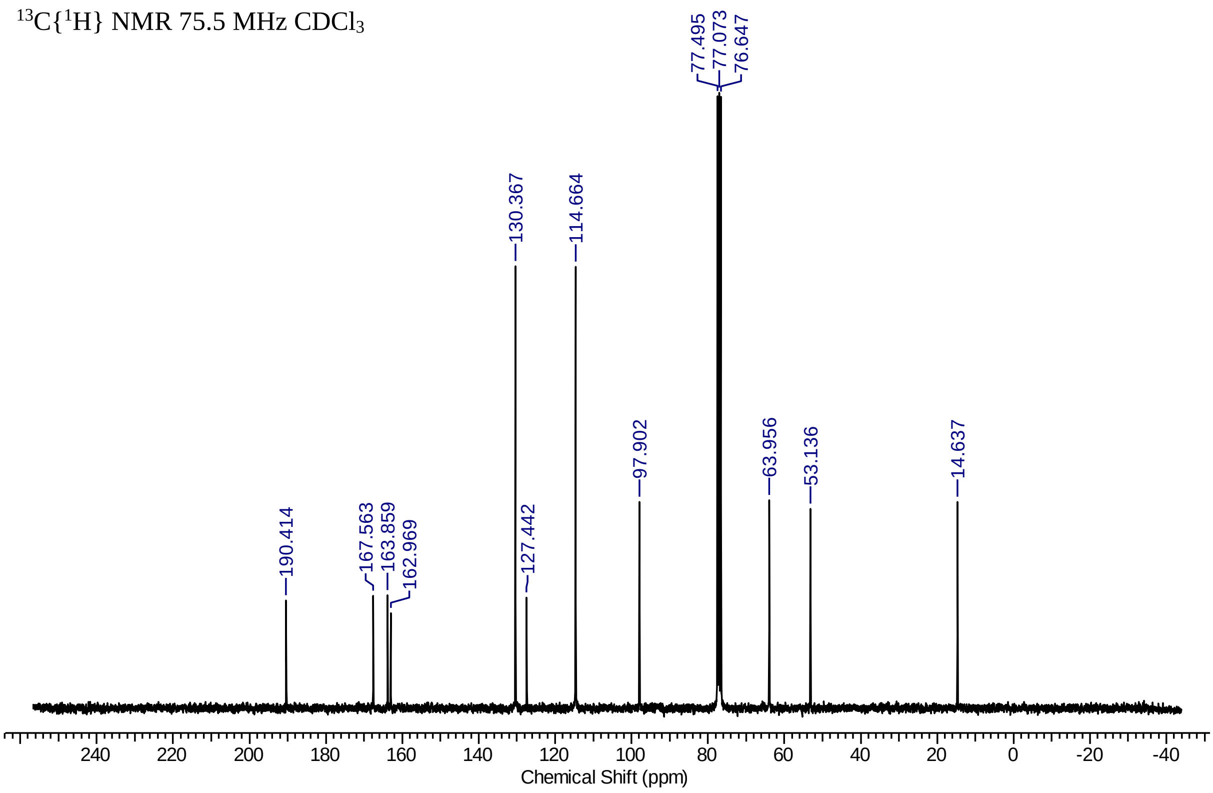

Figure S4. ${}^{1}H$ and ${}^{13}C$ NMR spectra of compound **1c**.

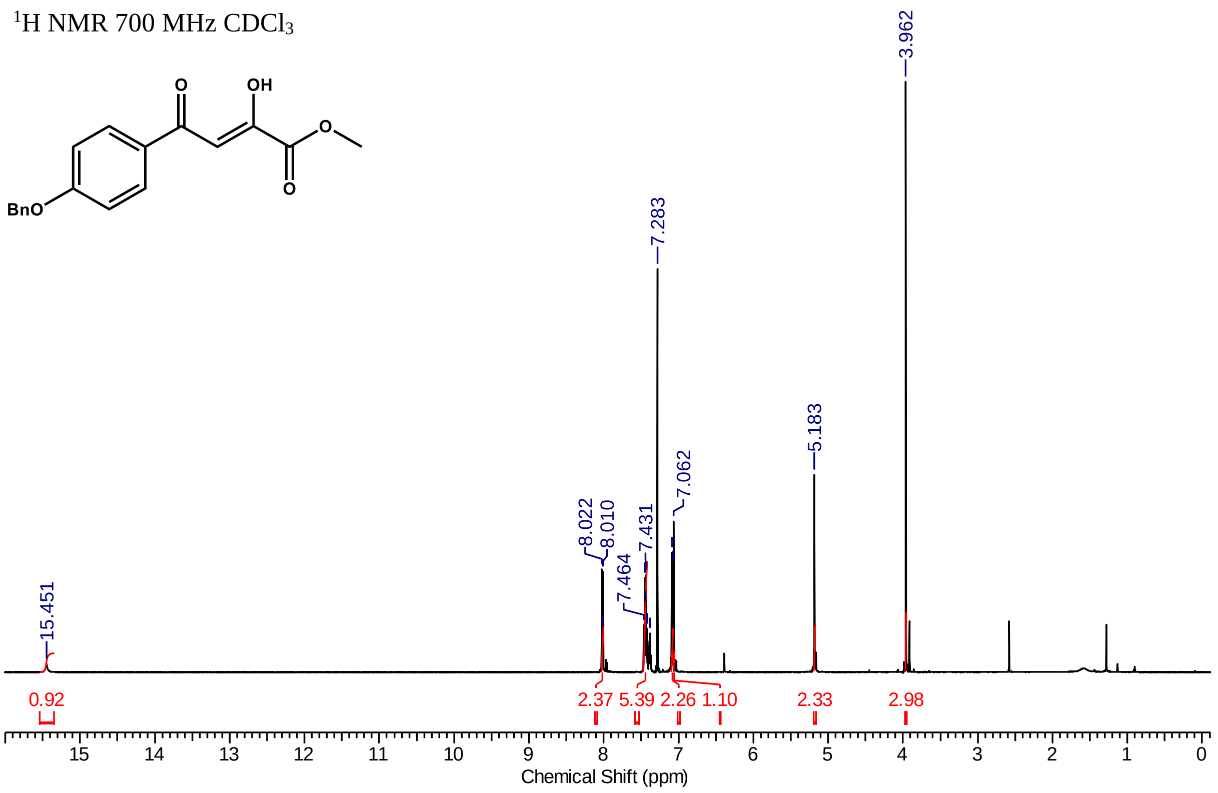

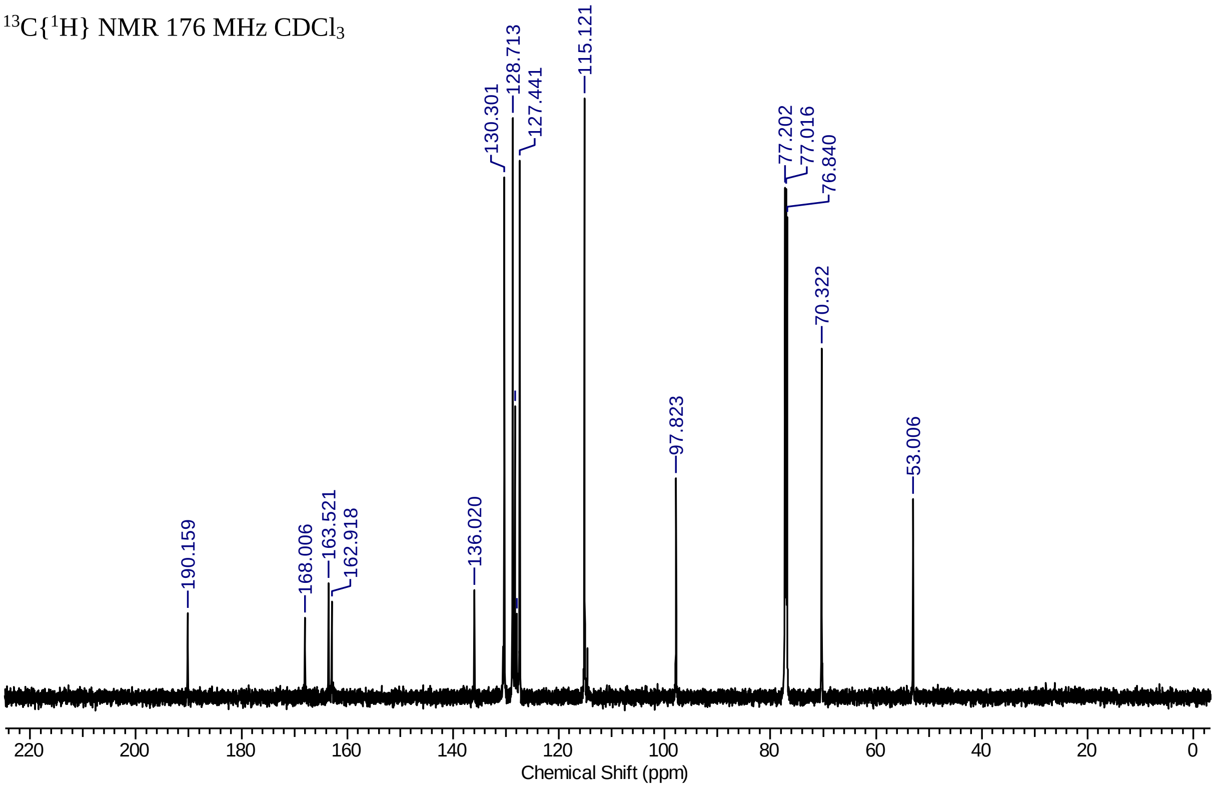

Figure S5. ^{1}H and ^{13}C NMR spectra of compound **1d**.

^{1}H NMR 700 MHz $CDCl_3$

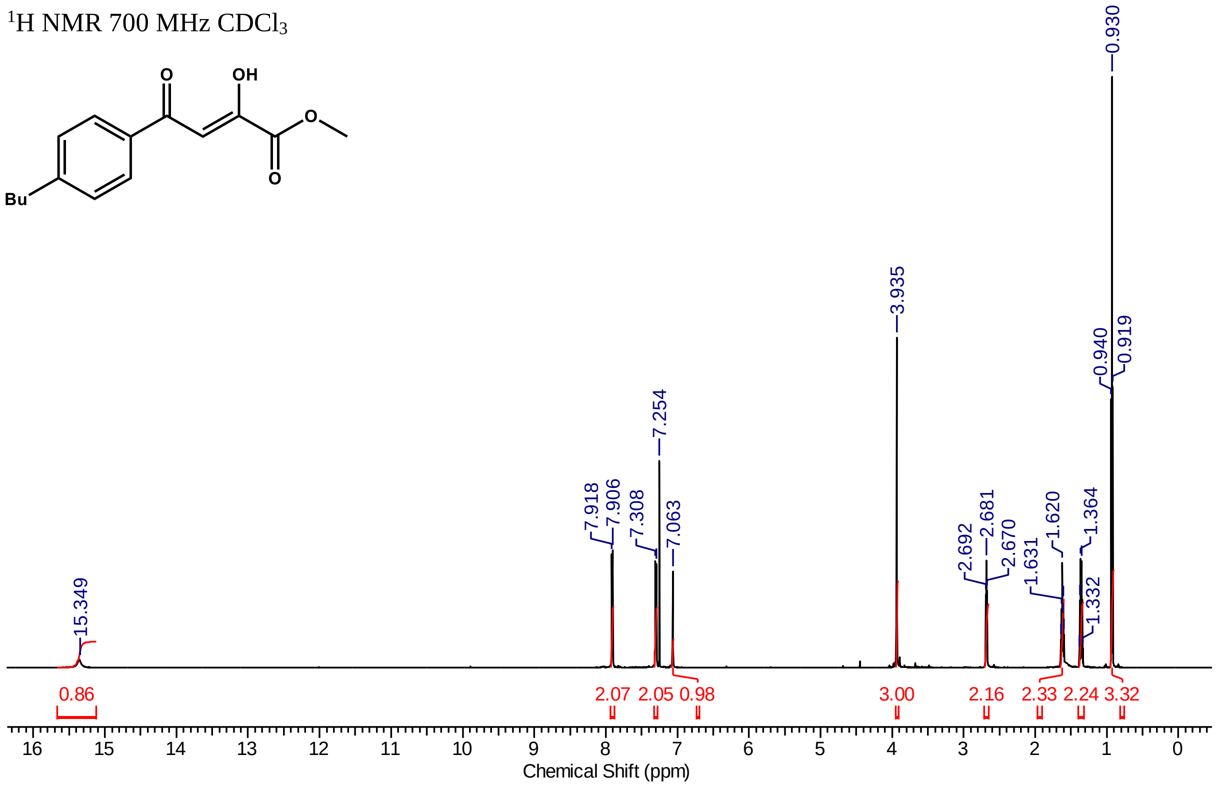

$^{13}C\{^{1}H\}$ NMR 75.5 MHz $CDCl_3$

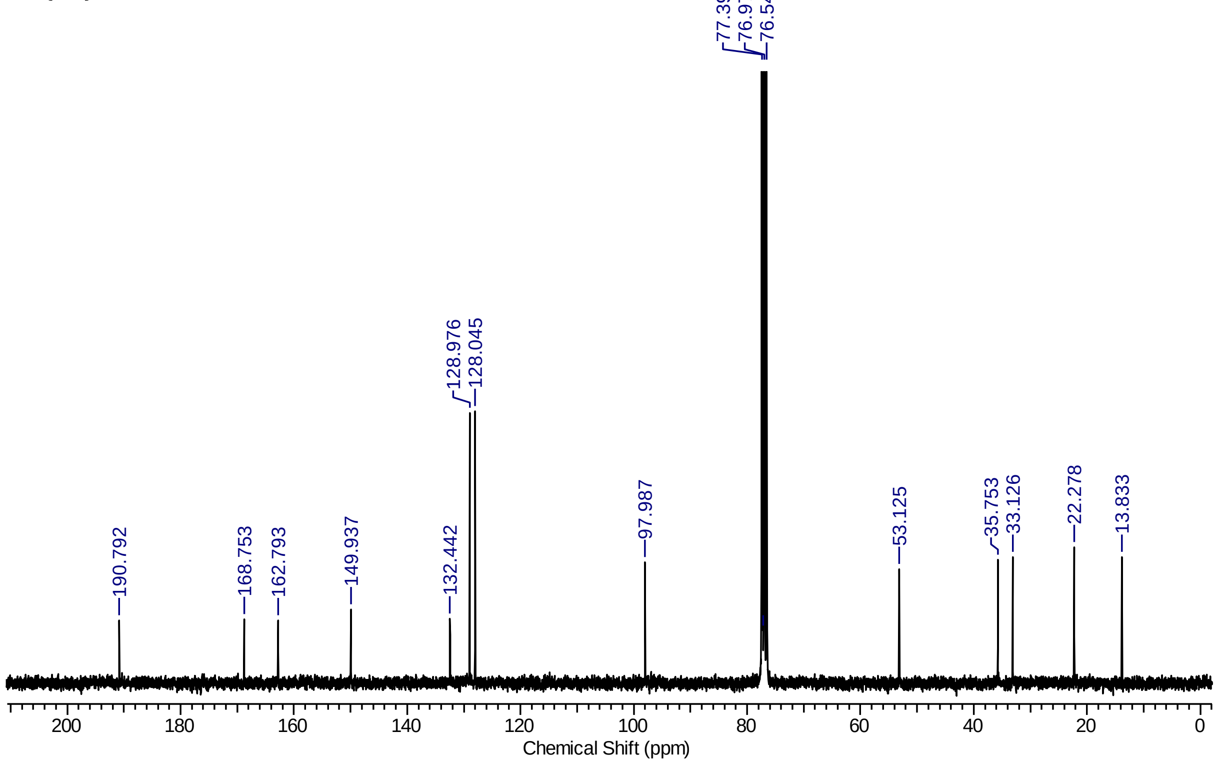

Figure S6. ^{1}H and ^{13}C NMR spectra of compound **1e**.

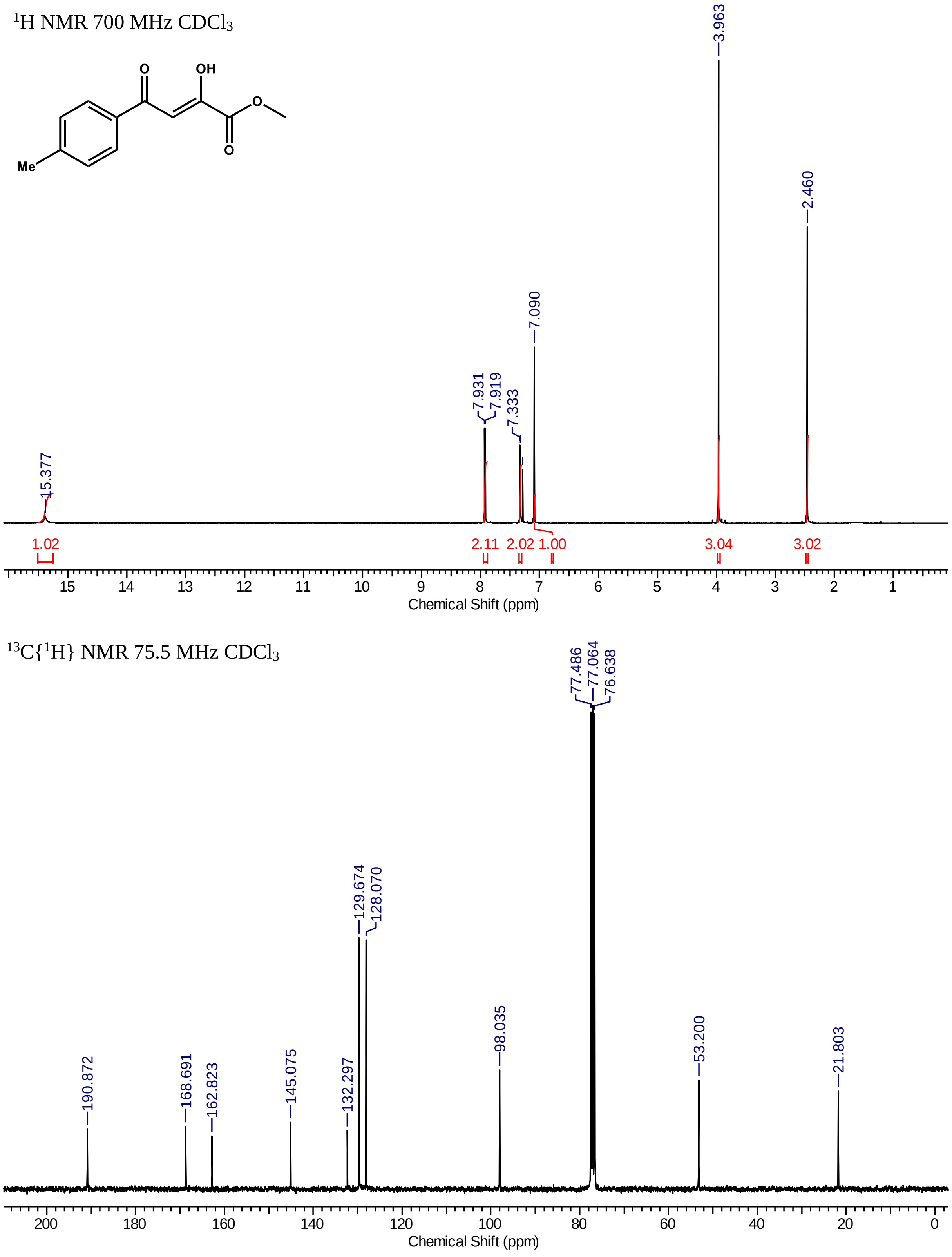

Figure S7. ^{1}H and ^{13}C NMR spectra of compound **1f**.

^{1}H NMR 700 MHz $CDCl_3$

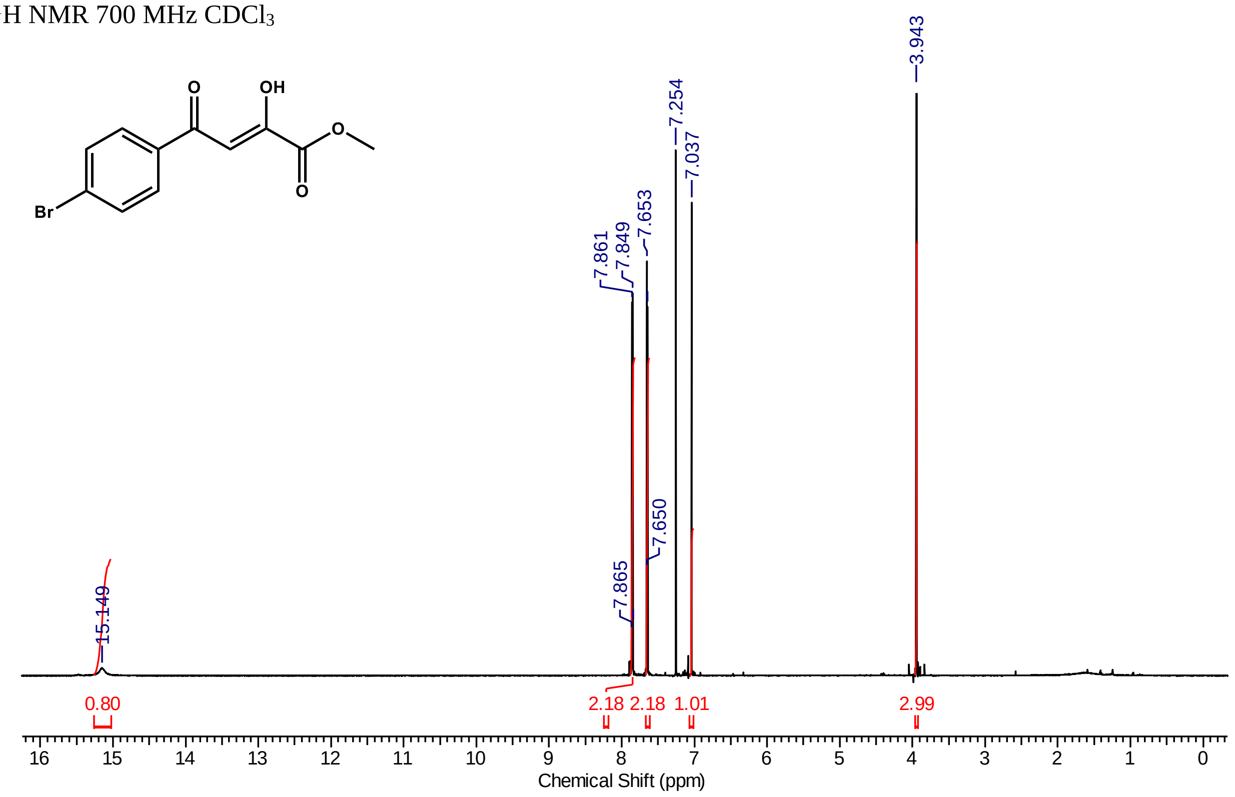

$^{13}C\{^{1}H\}$ NMR 176 MHz $CDCl_3$

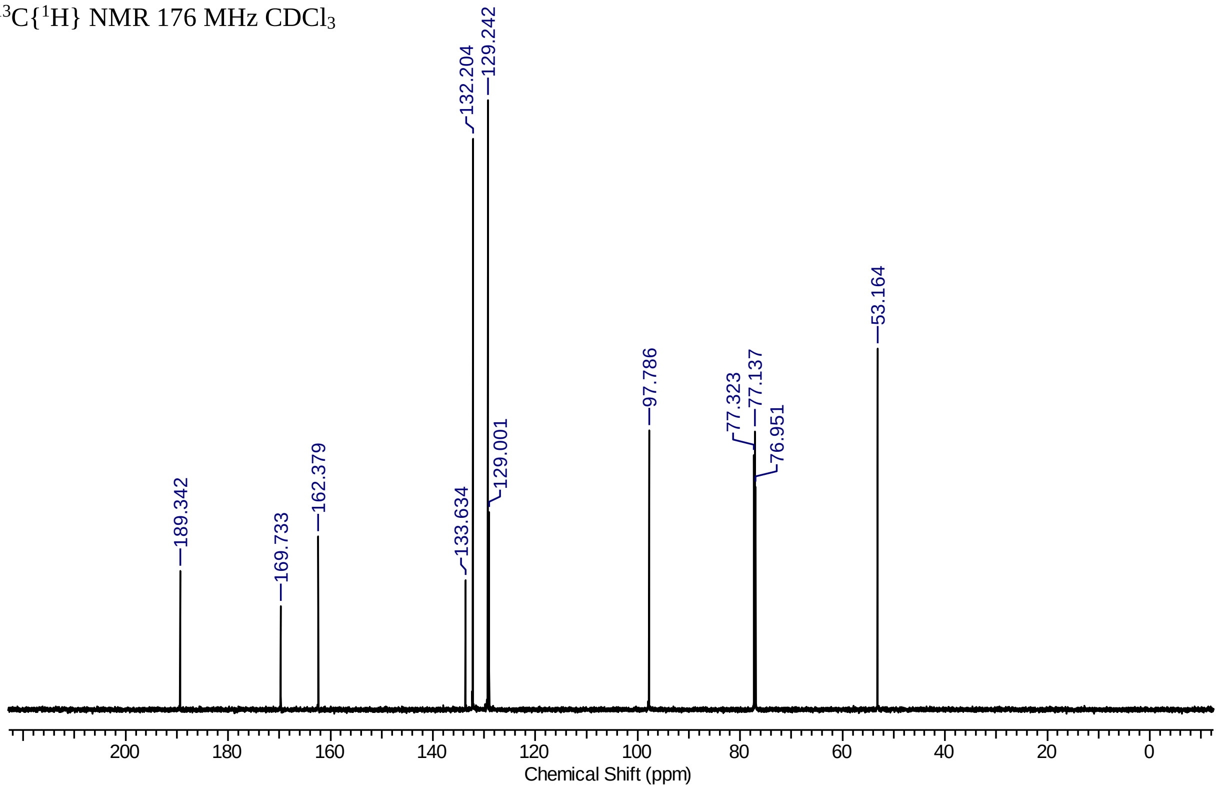

Figure S8. ^{1}H and ^{13}C NMR spectra of compound **1g**.

^1H NMR 700 MHz $CDCl_3$

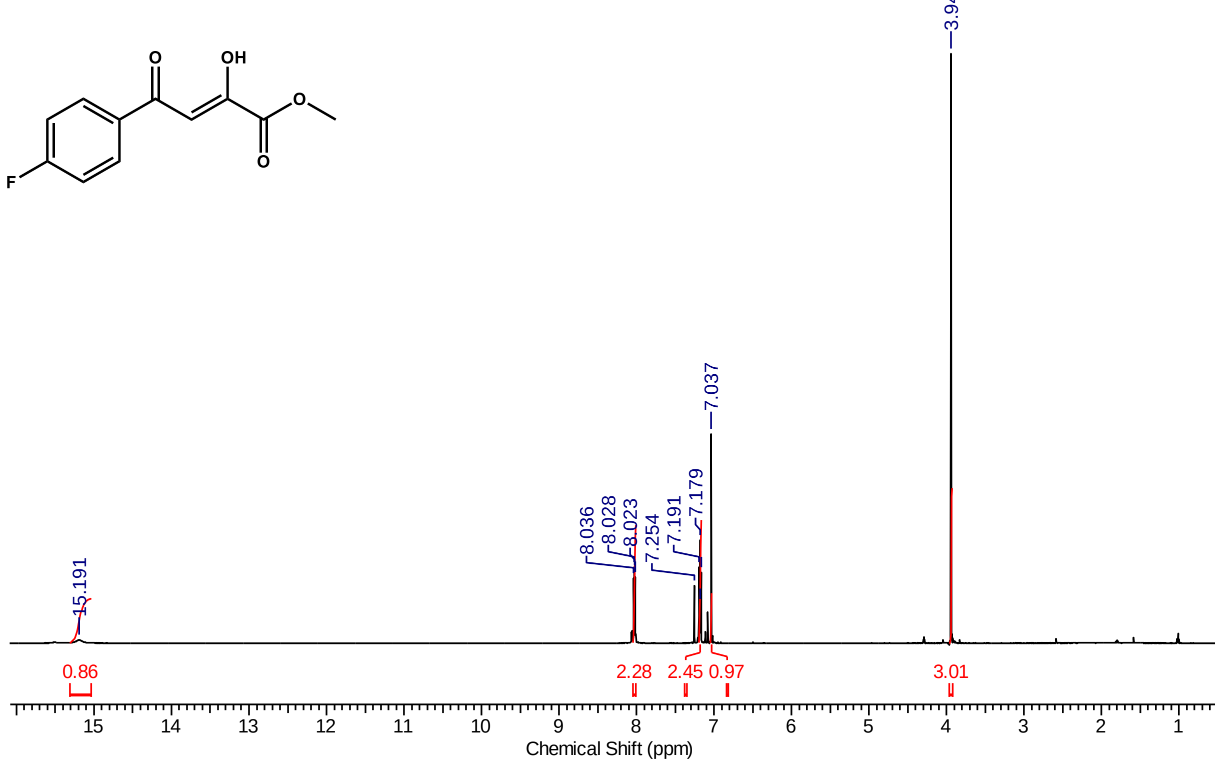

$^{13}C\{^1H\}$ NMR 176 MHz $CDCl_3$

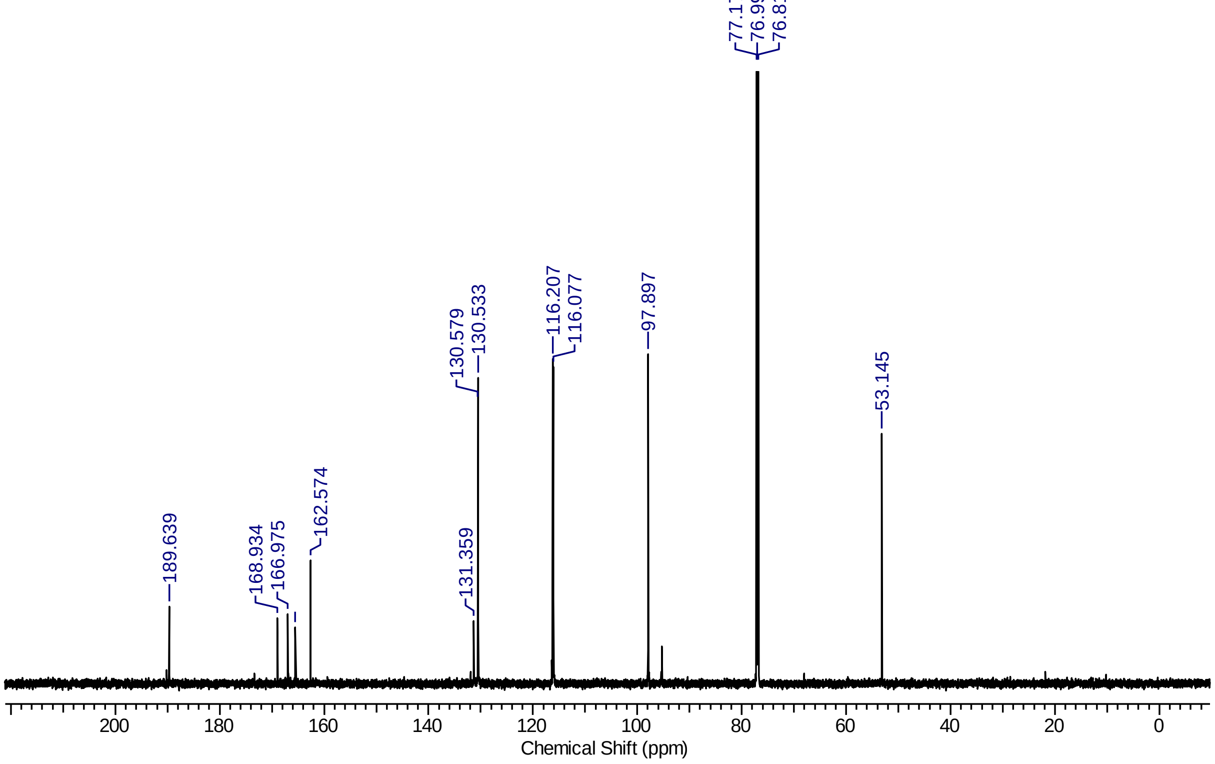

Figure S9. ^1H and ^{13}C NMR spectra of compound **1h**.

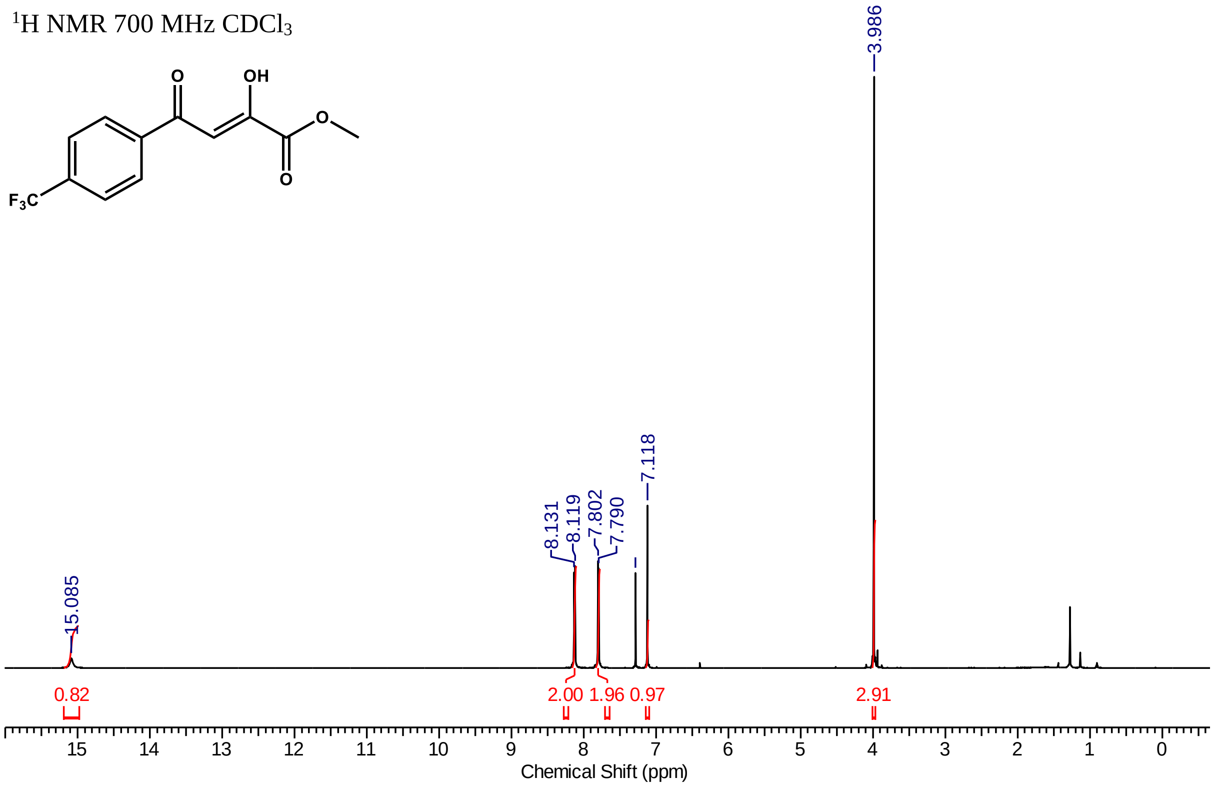

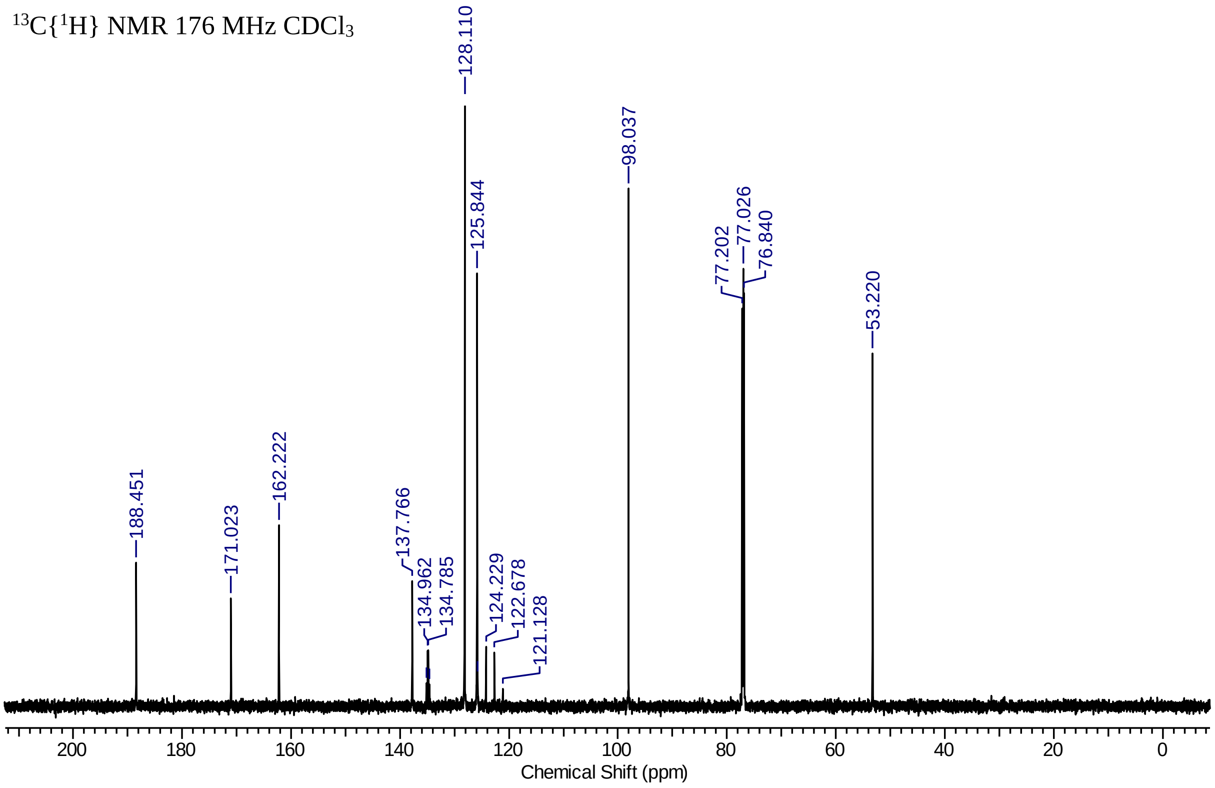

Figure S10. ^{1}H and ^{13}C NMR spectra of compound **1i**.

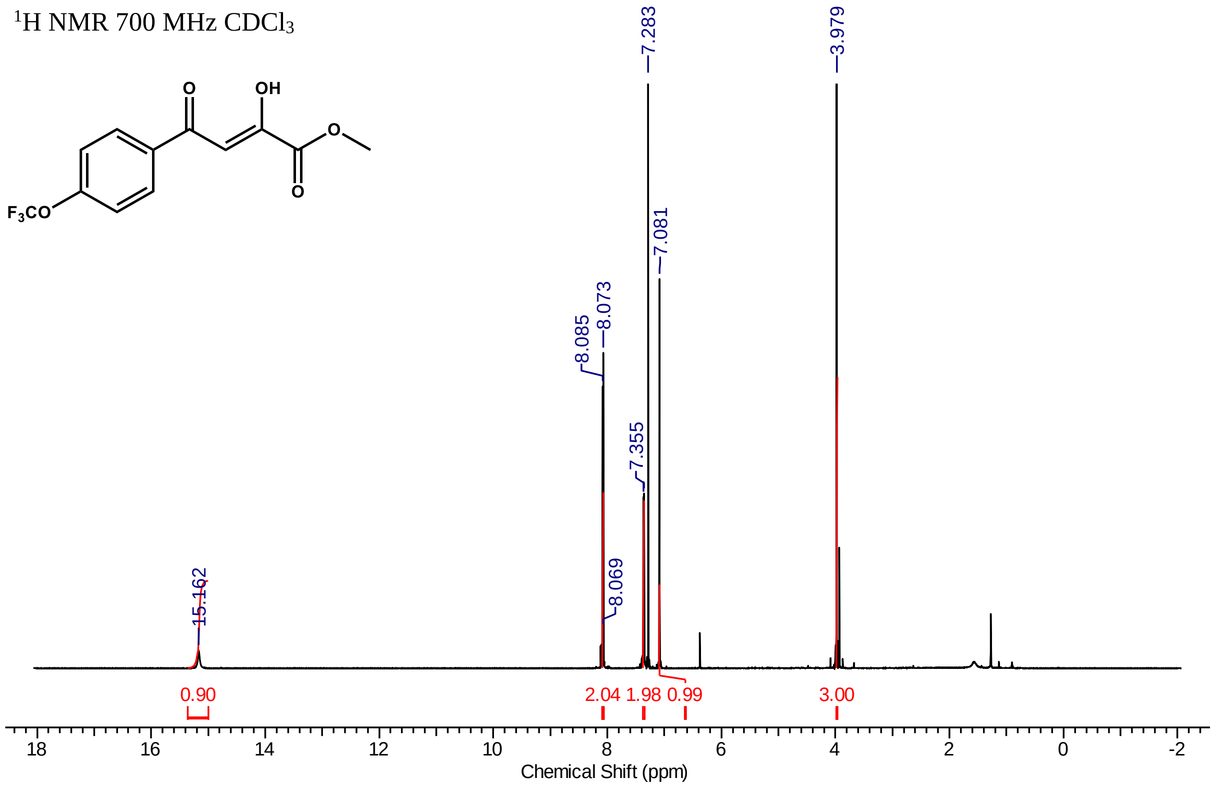

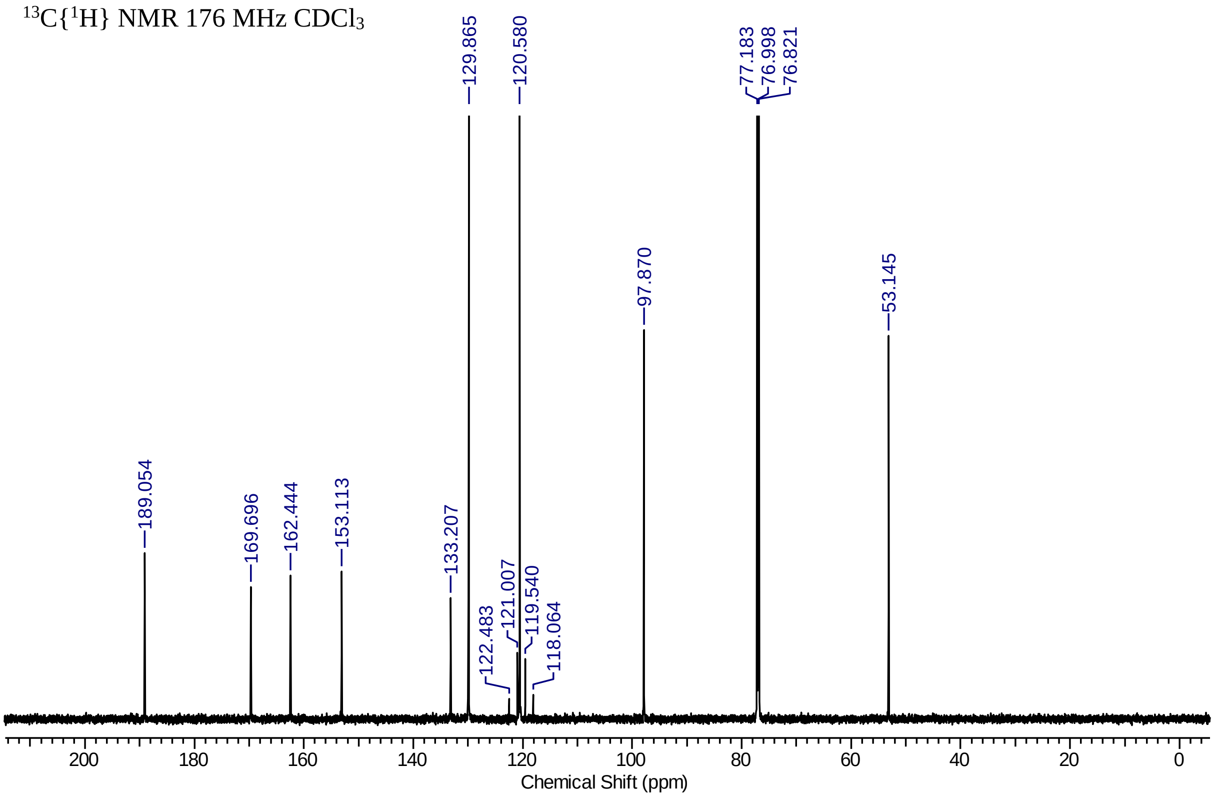

Figure S11. ^{1}H and ^{13}C NMR spectra of compound **1j**.

^1H NMR 700 MHz $CDCl_3$

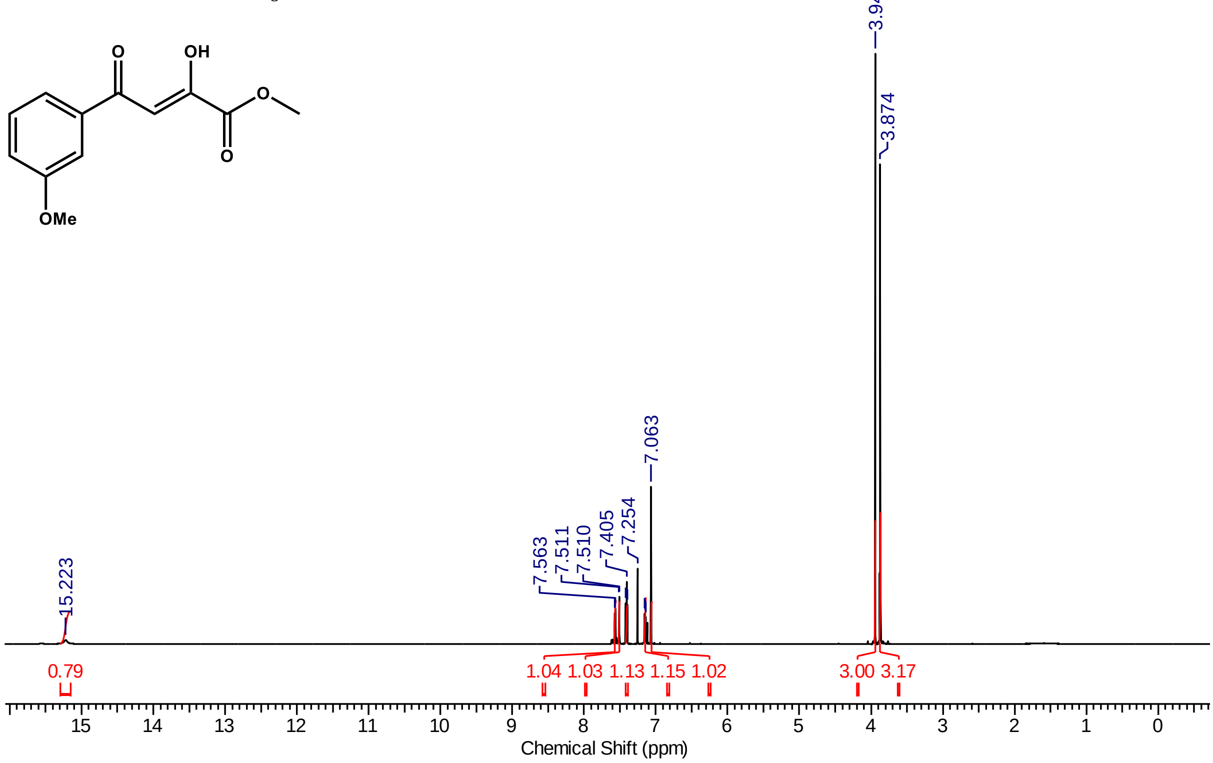

$^{13}C\{^1H\}$ NMR 176 MHz $CDCl_3$

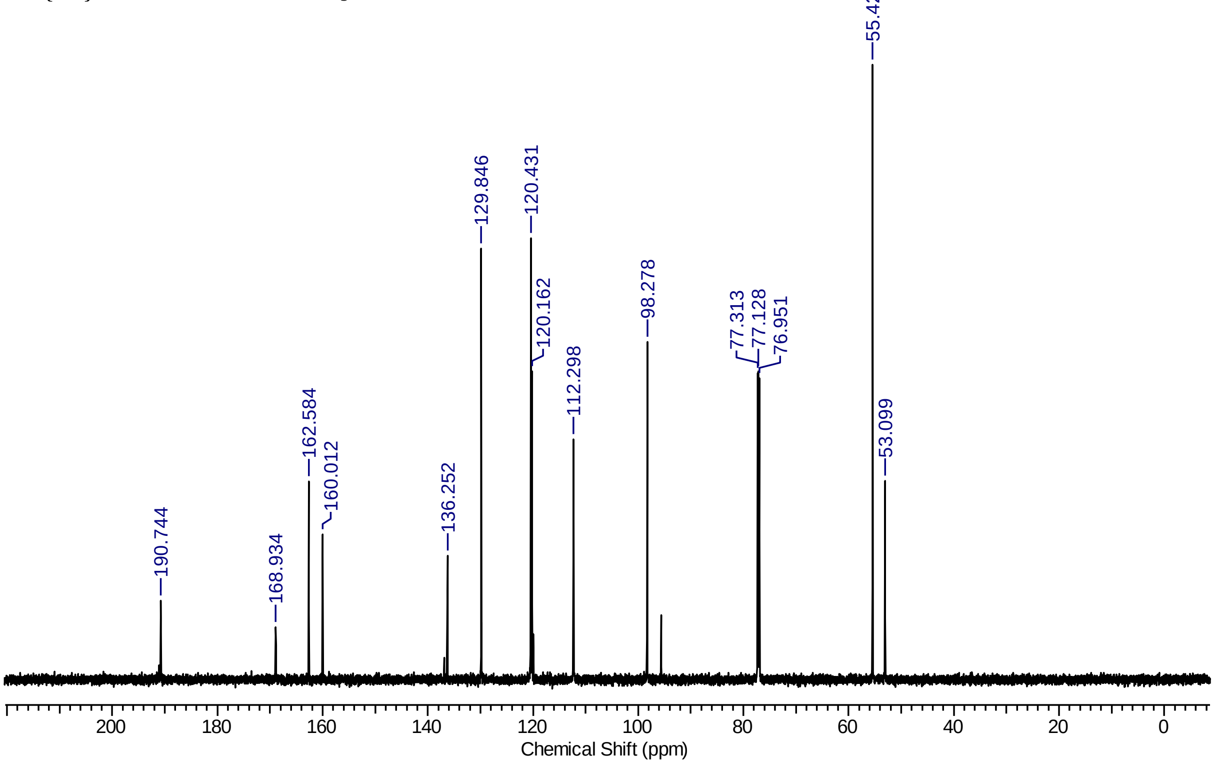

Figure S12. ^1H and ^{13}C NMR spectra of compound **1k**.

^{1}H NMR 700 MHz $CDCl_3$

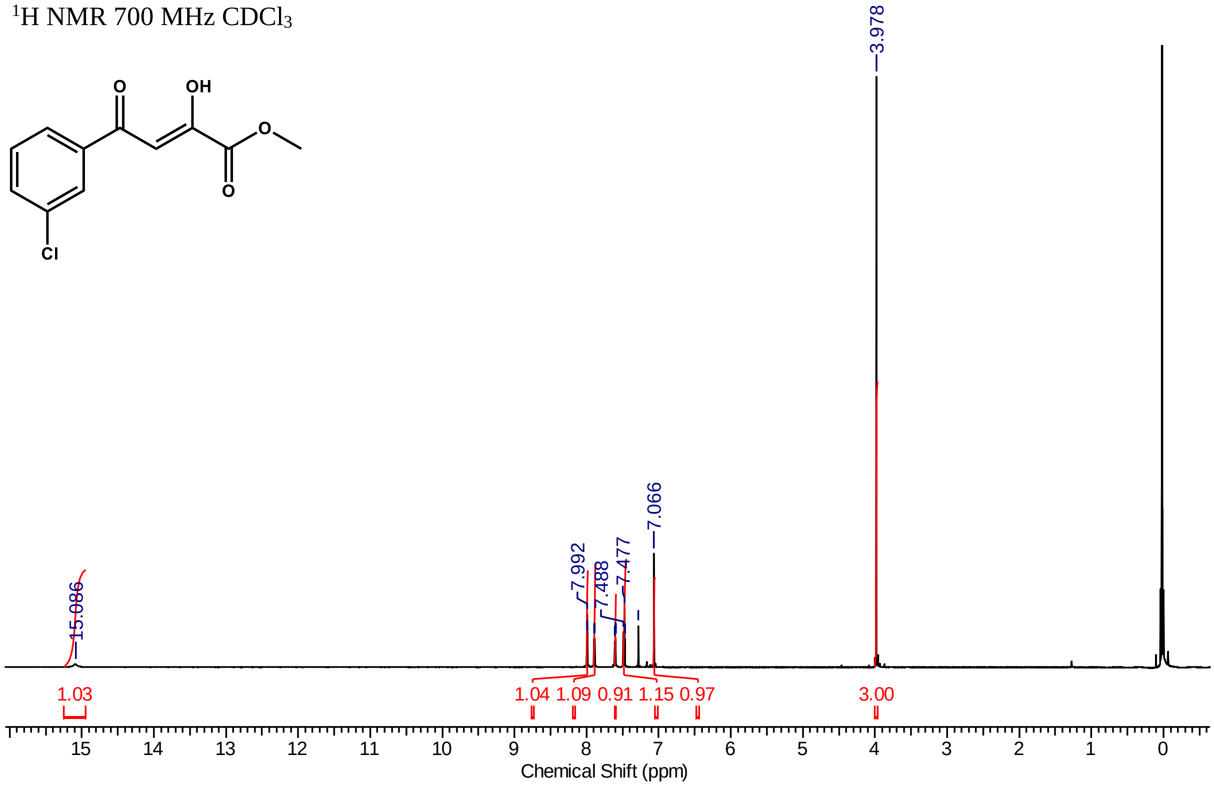

^{13}C{^{1}H} NMR 176 MHz $CDCl_3$

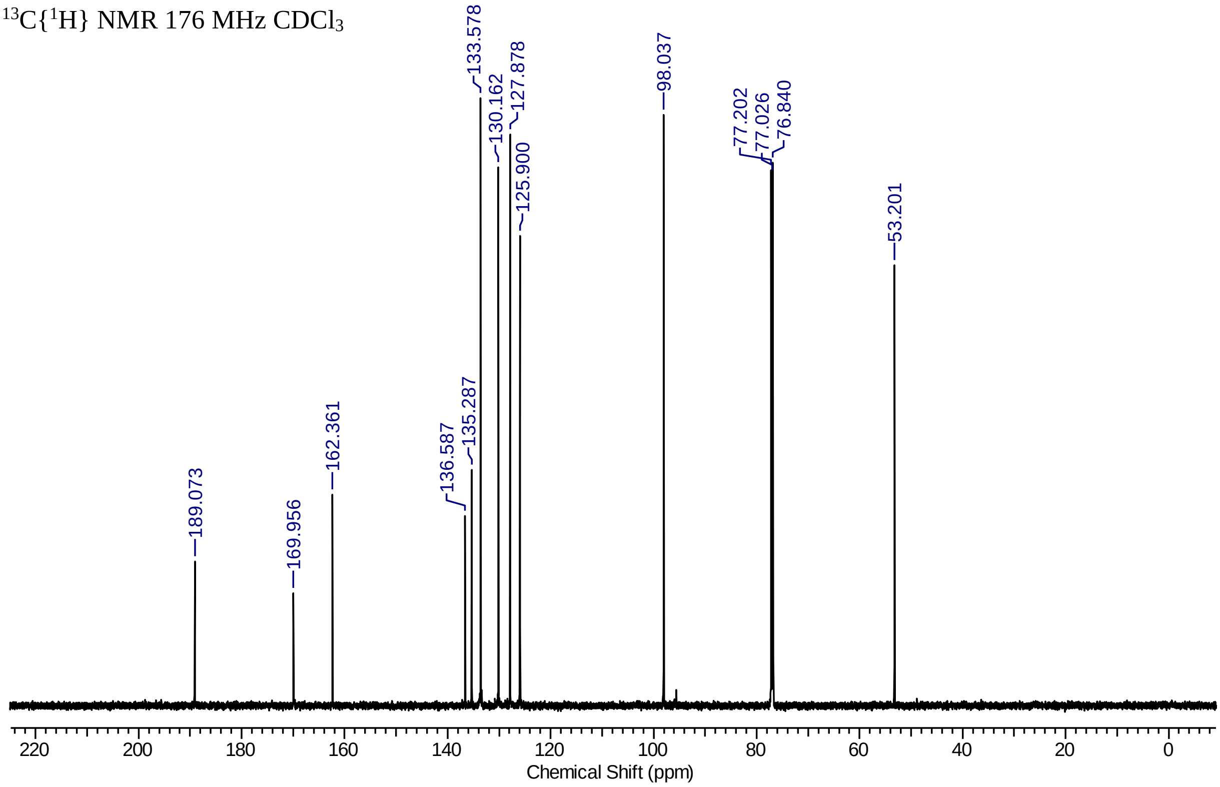

Figure S13. ^{1}H and ^{13}C NMR spectra of compound **1l**.

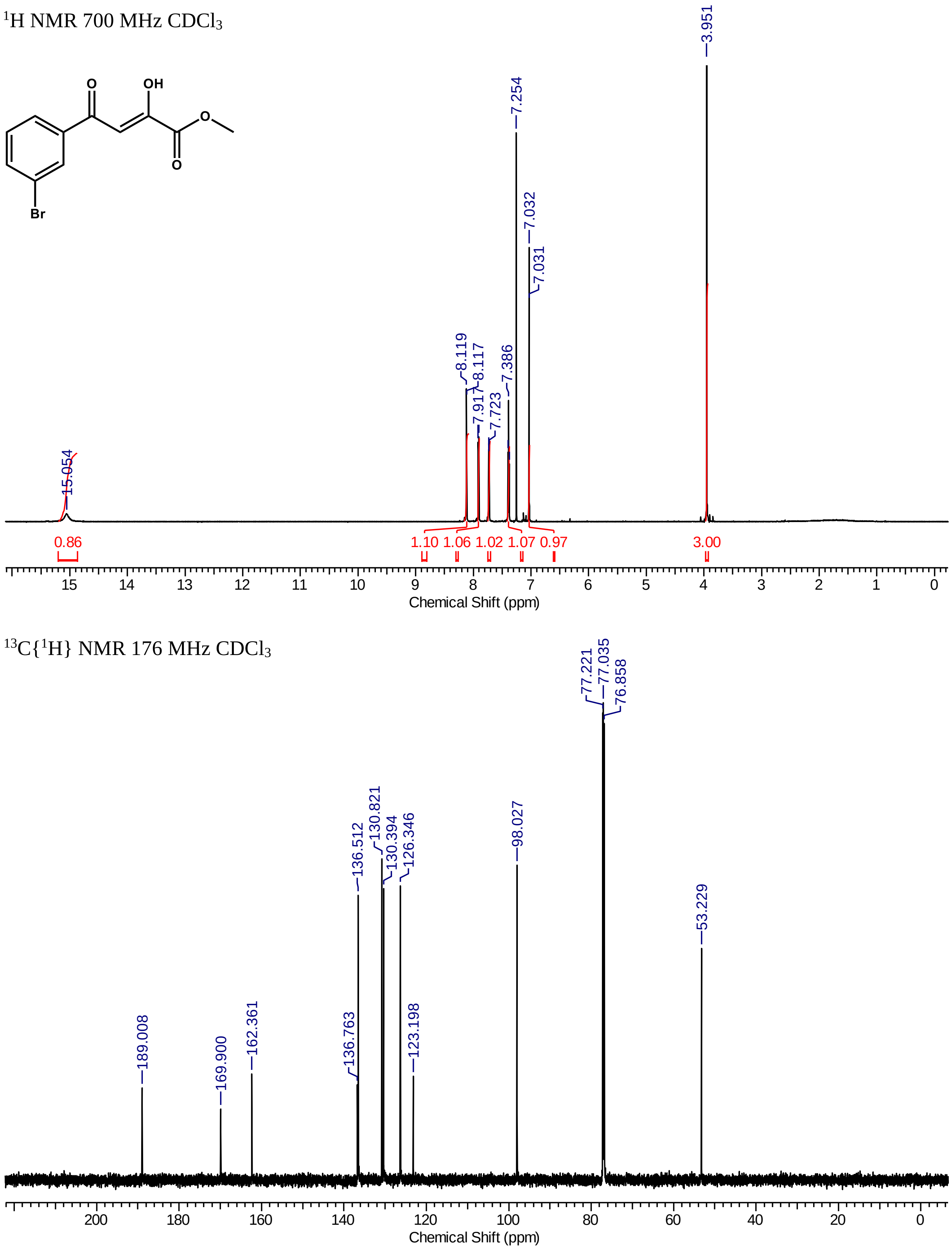

Figure S14. ^{1}H and ^{13}C NMR spectra of compound **1m**.

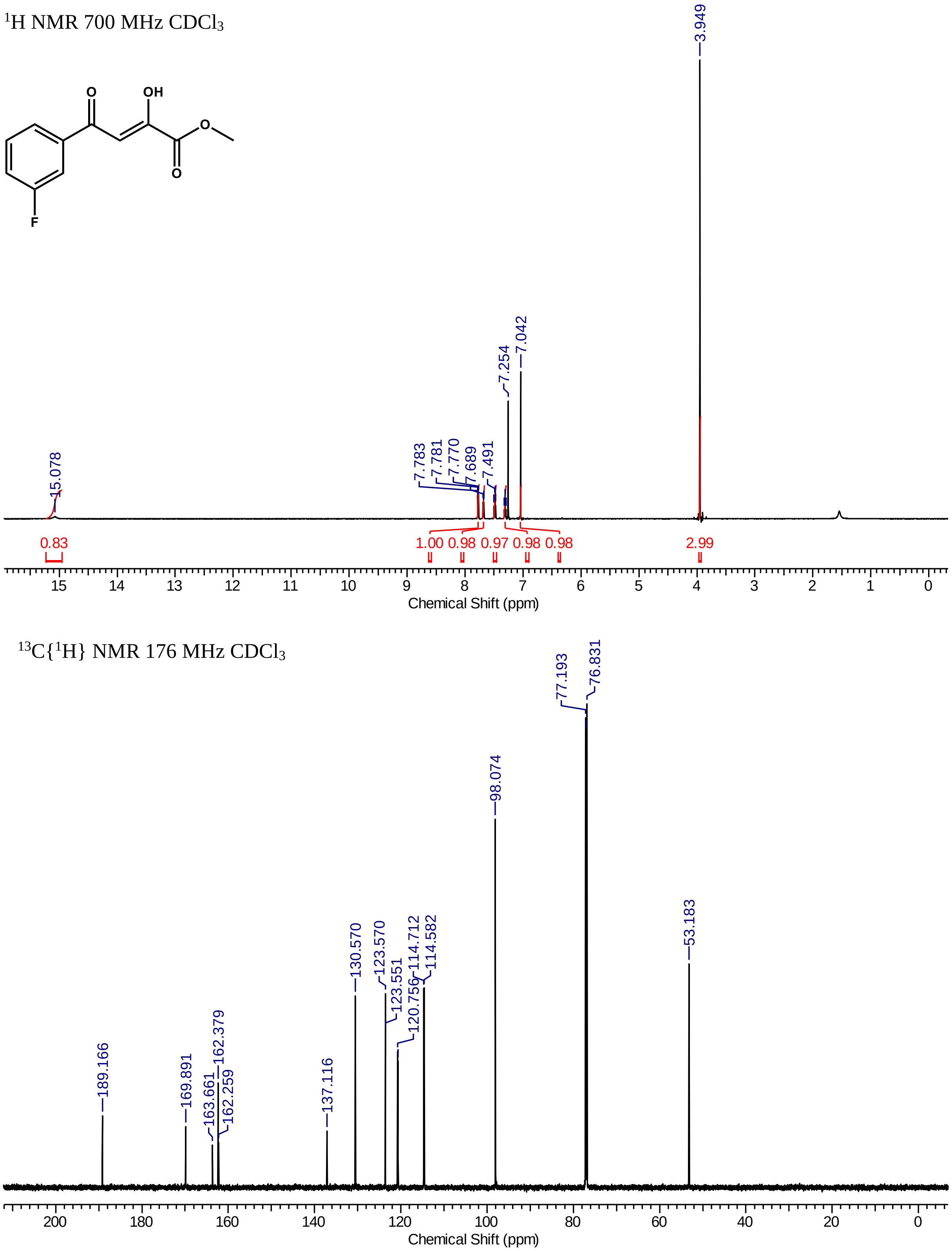

Figure S15. ^{1}H and ^{13}C NMR spectra of compound **1n**.

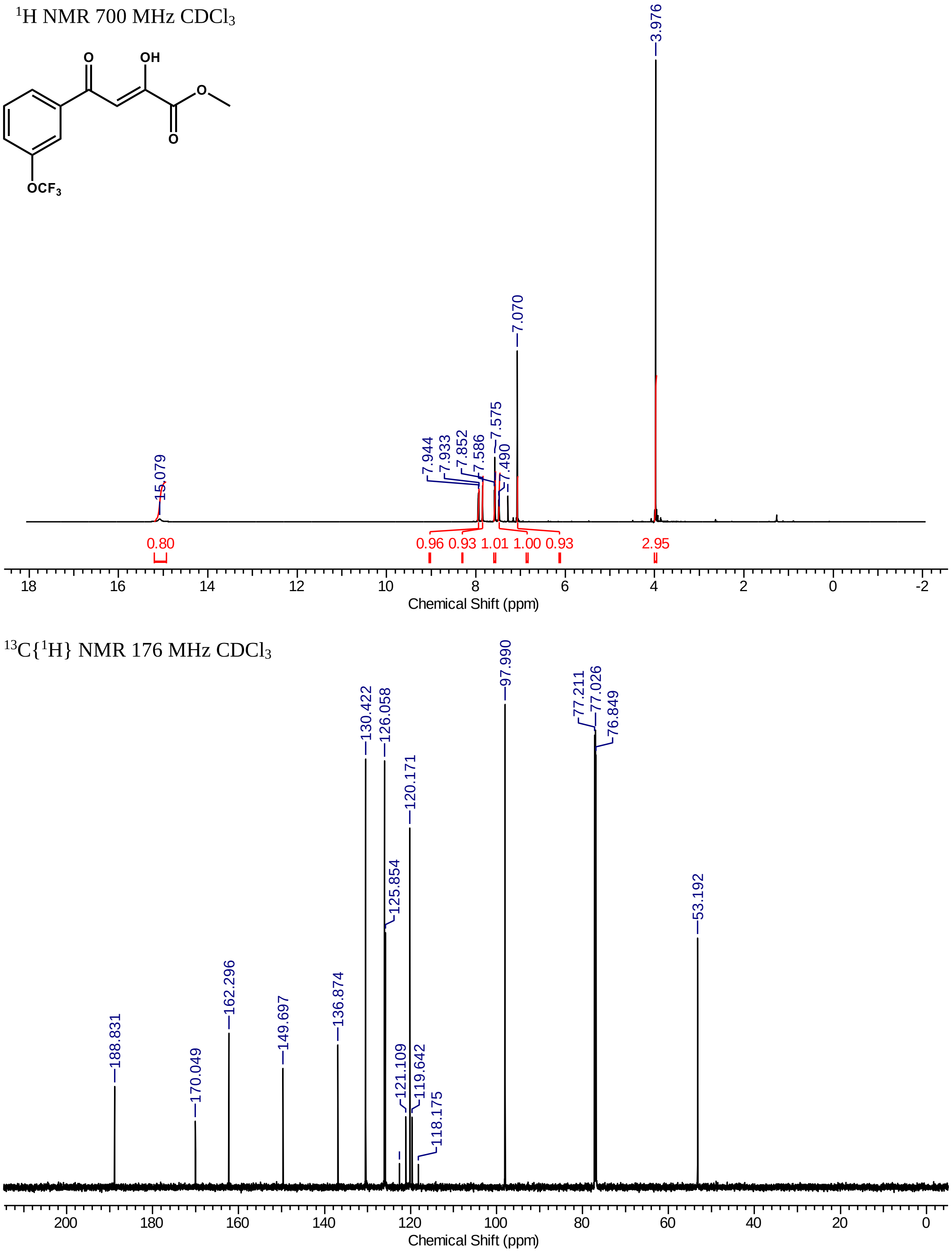

Figure S16. ^{1}H and ^{13}C NMR spectra of compound **1o**.

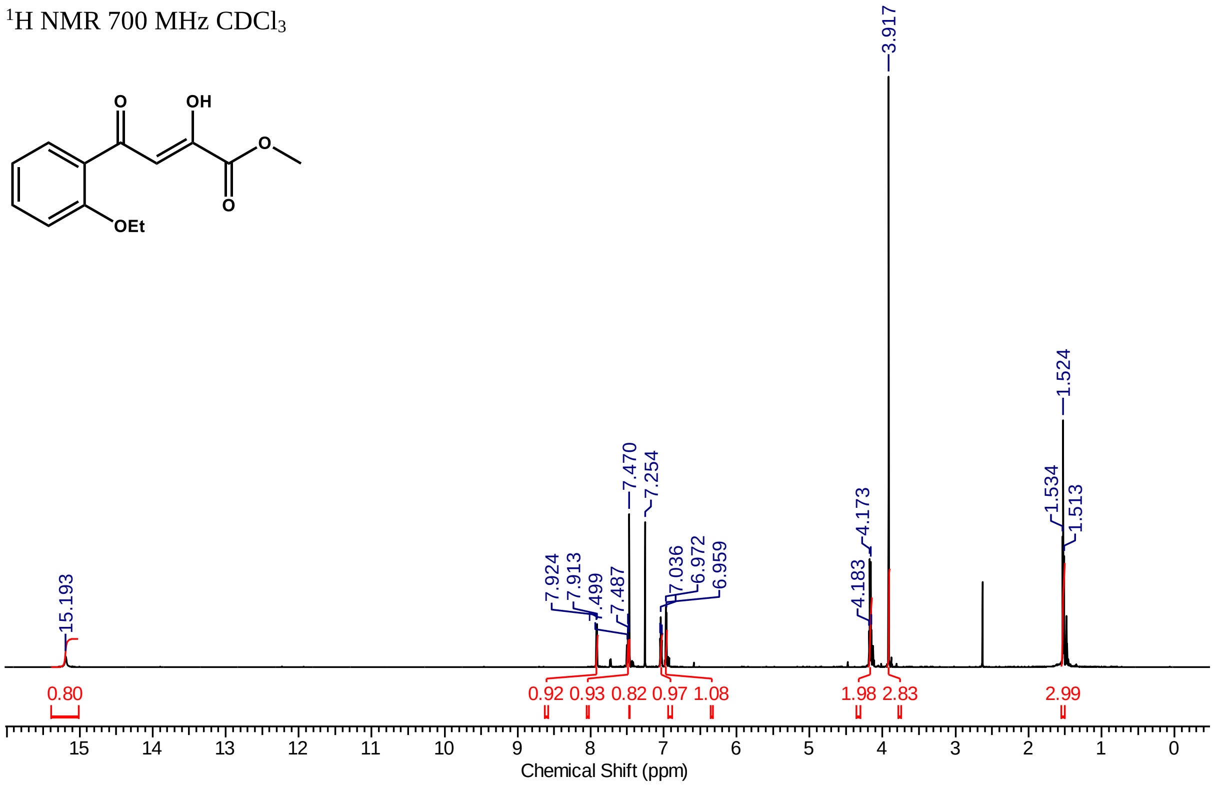

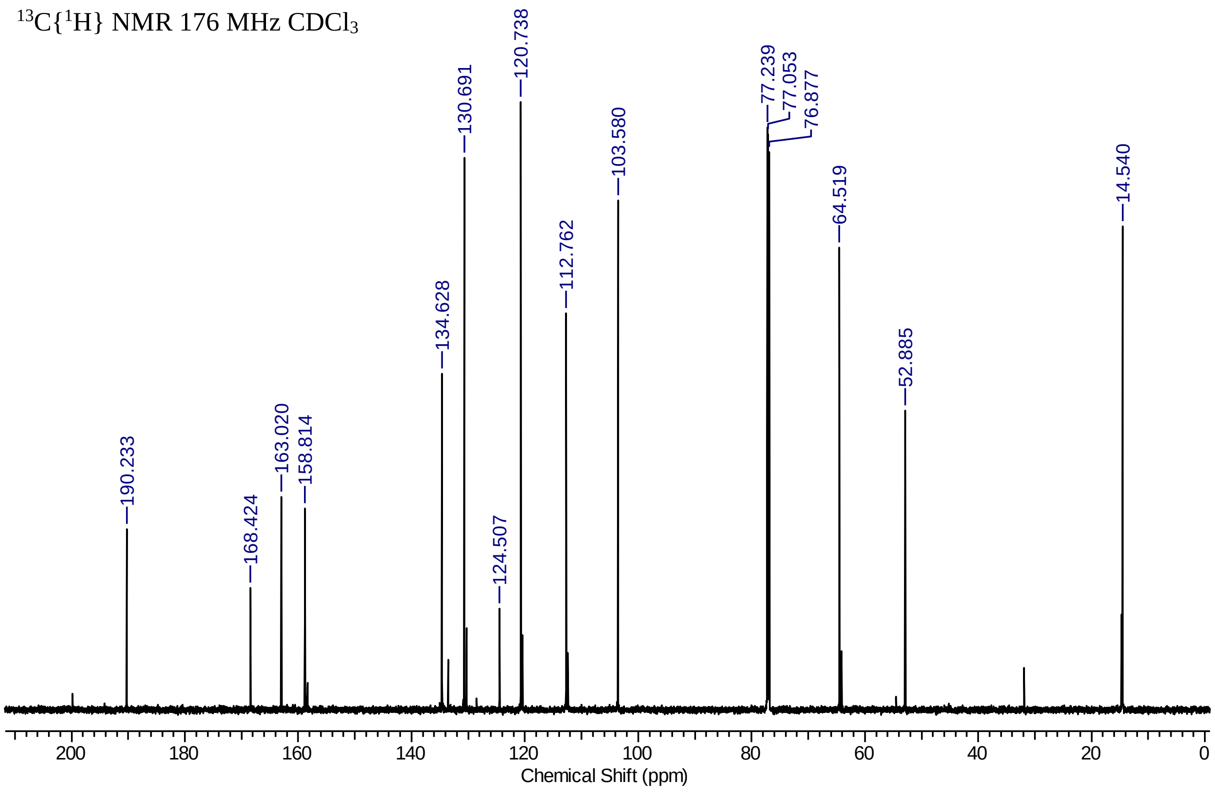

Figure S17. ^{1}H and ^{13}C NMR spectra of compound **1p**.

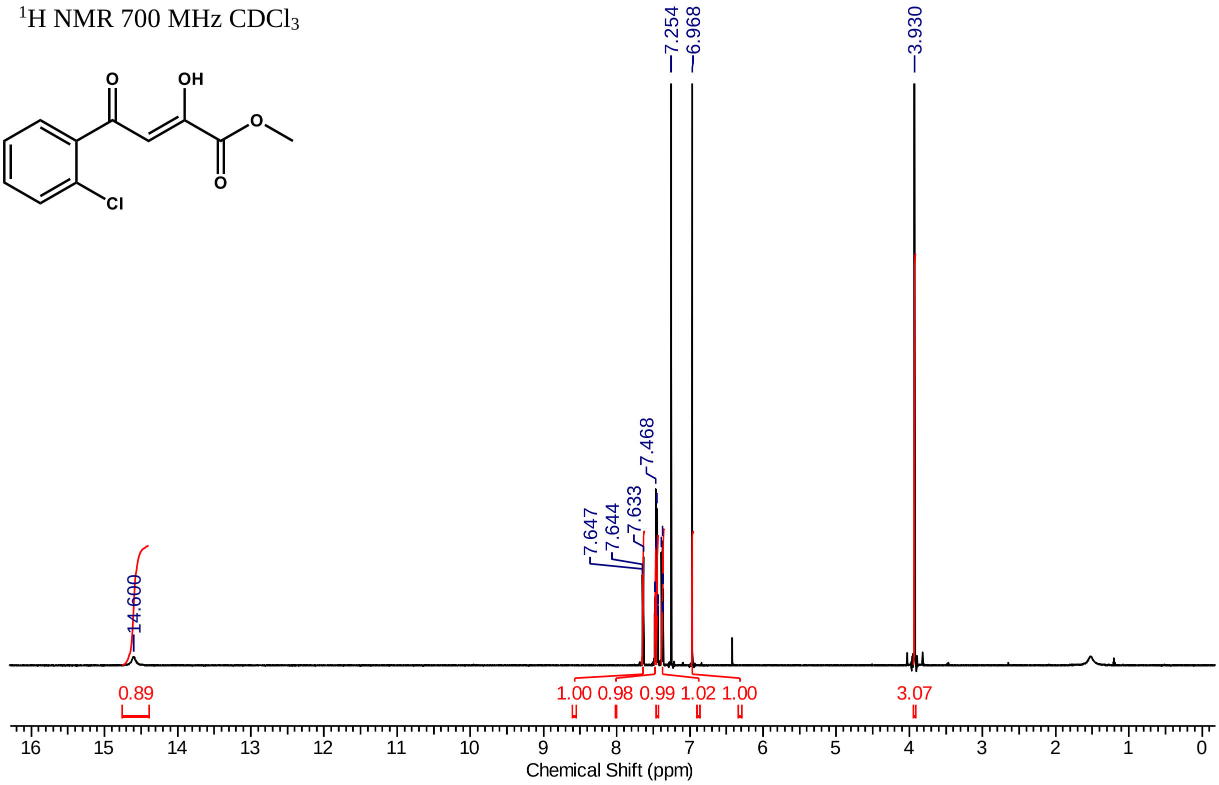

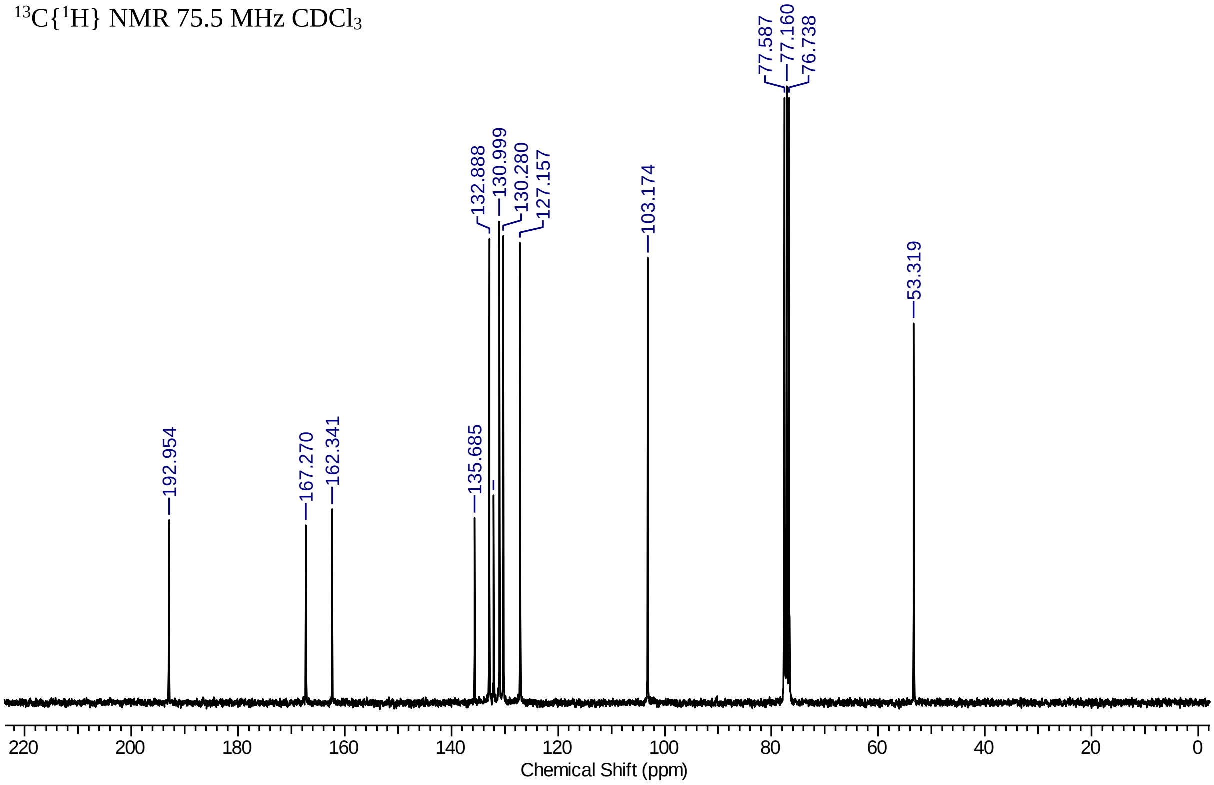

Figure S18. ^{1}H and ^{13}C NMR spectra of compound **1q**.

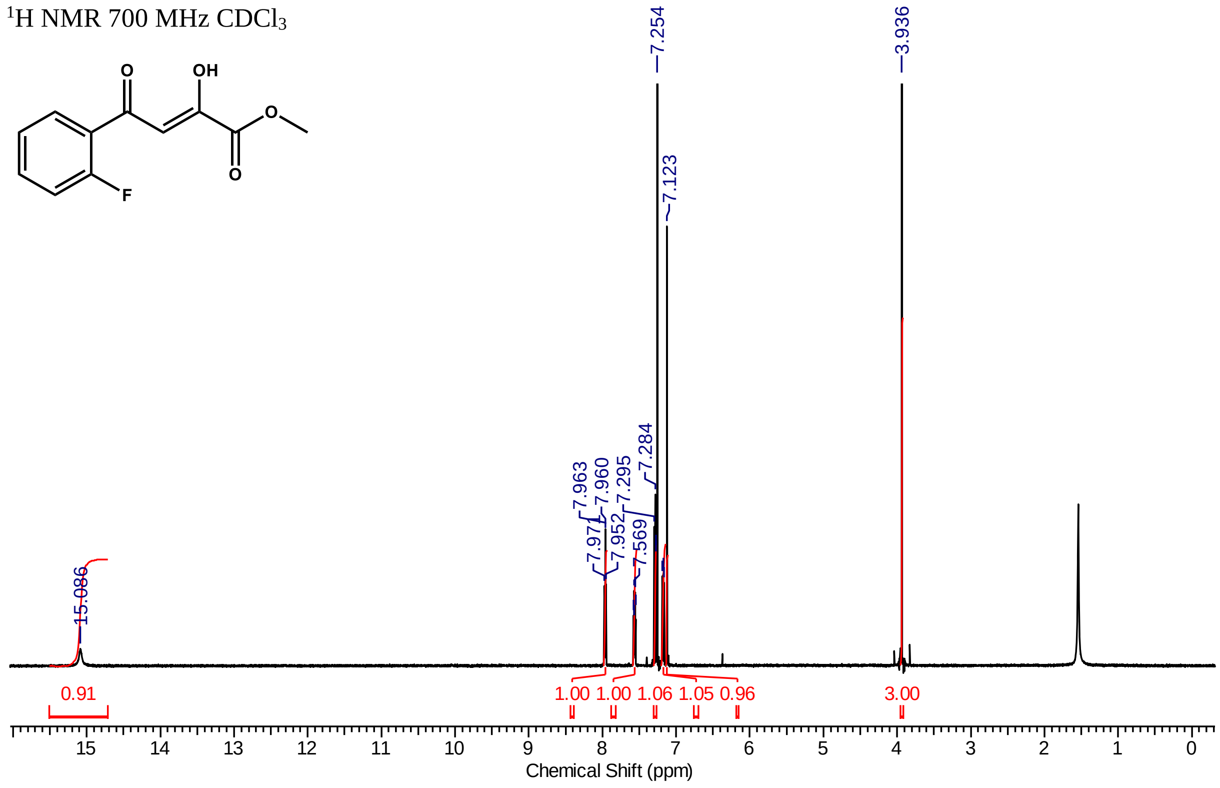

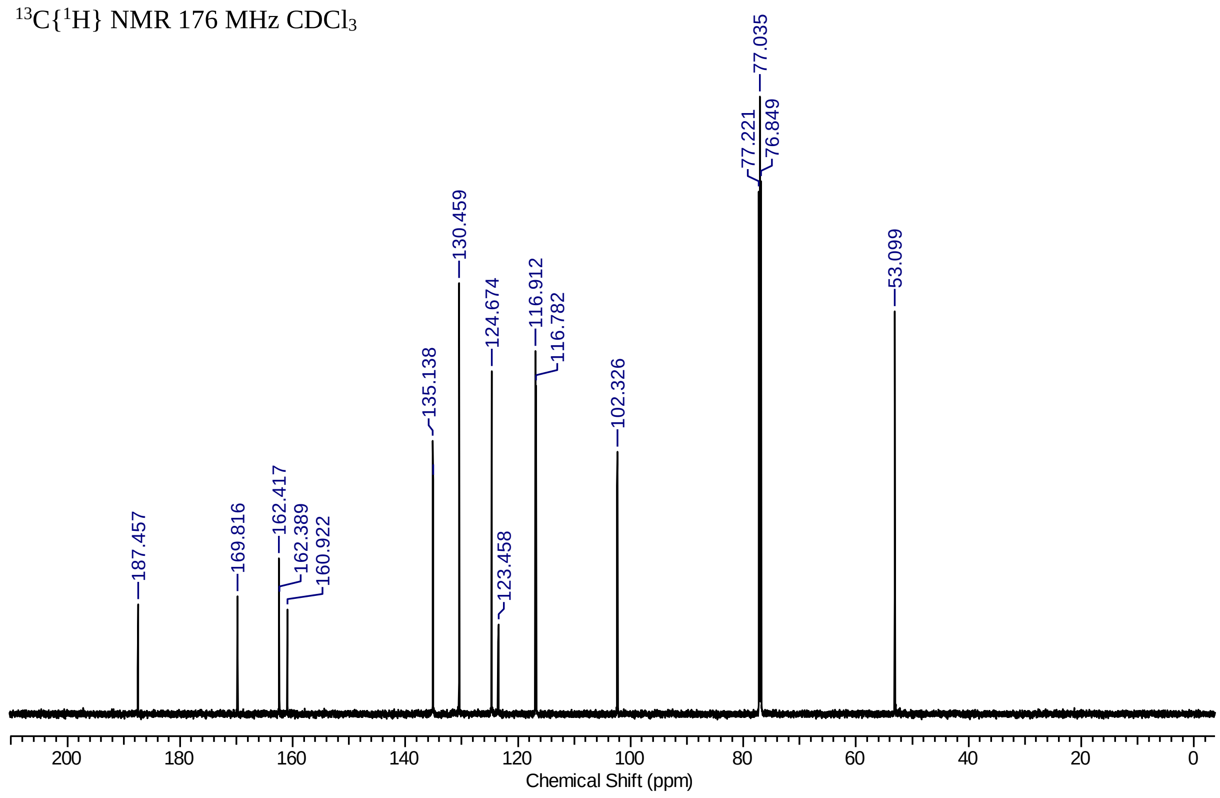

Figure S19. ^{1}H and ^{13}C NMR spectra of compound **1r**.

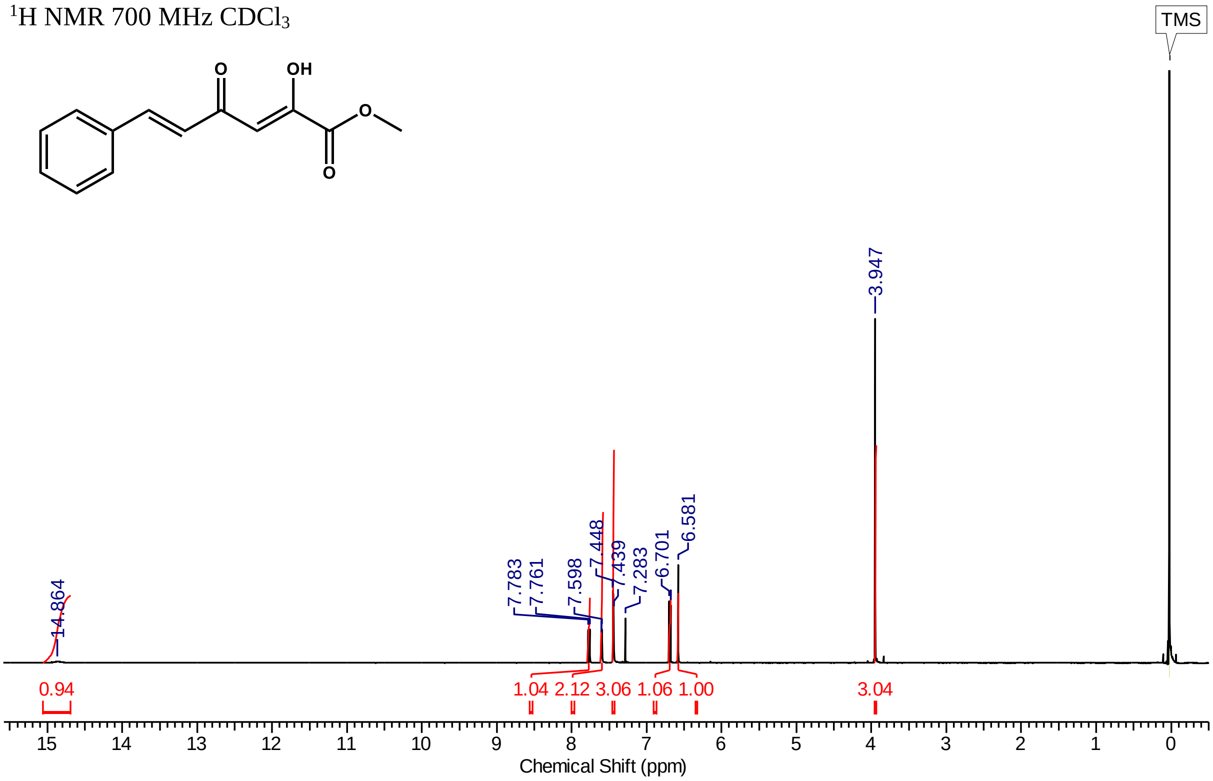

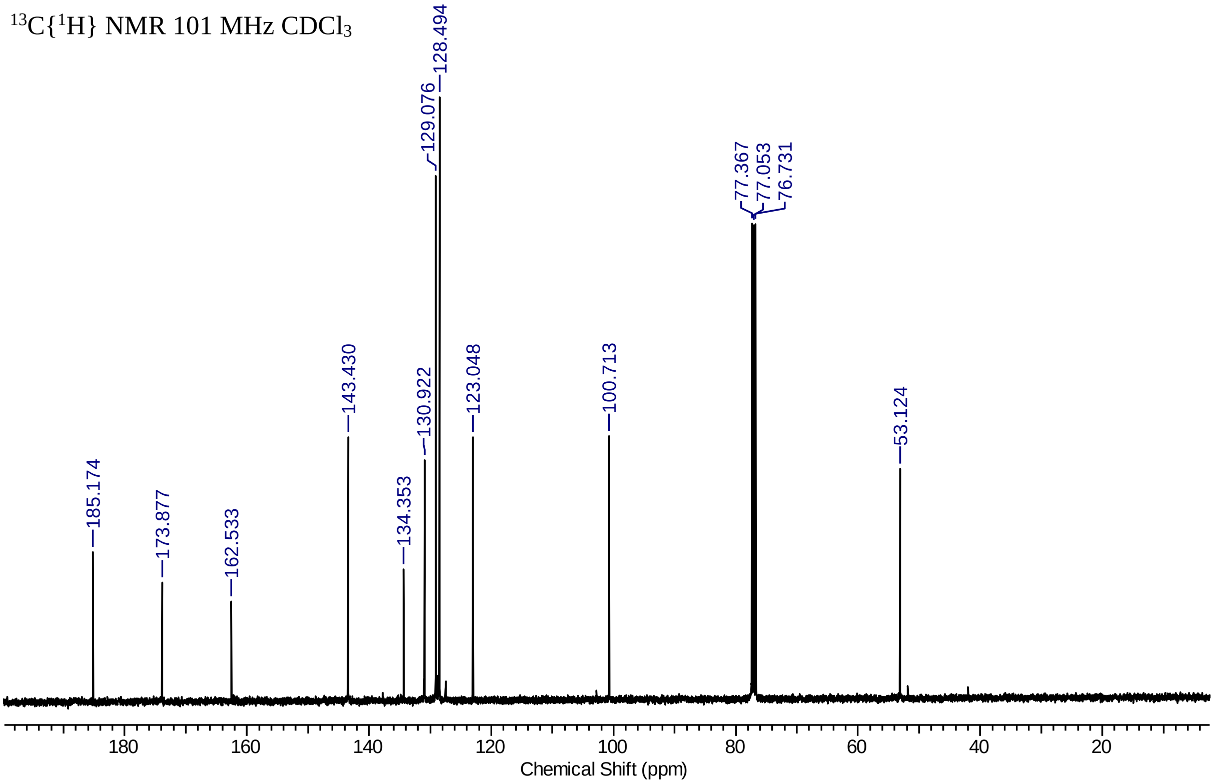

Figure S20. ^{1}H and ^{13}C NMR spectra of compound **1s**.

^{1}H NMR 700 MHz $CDCl_3$

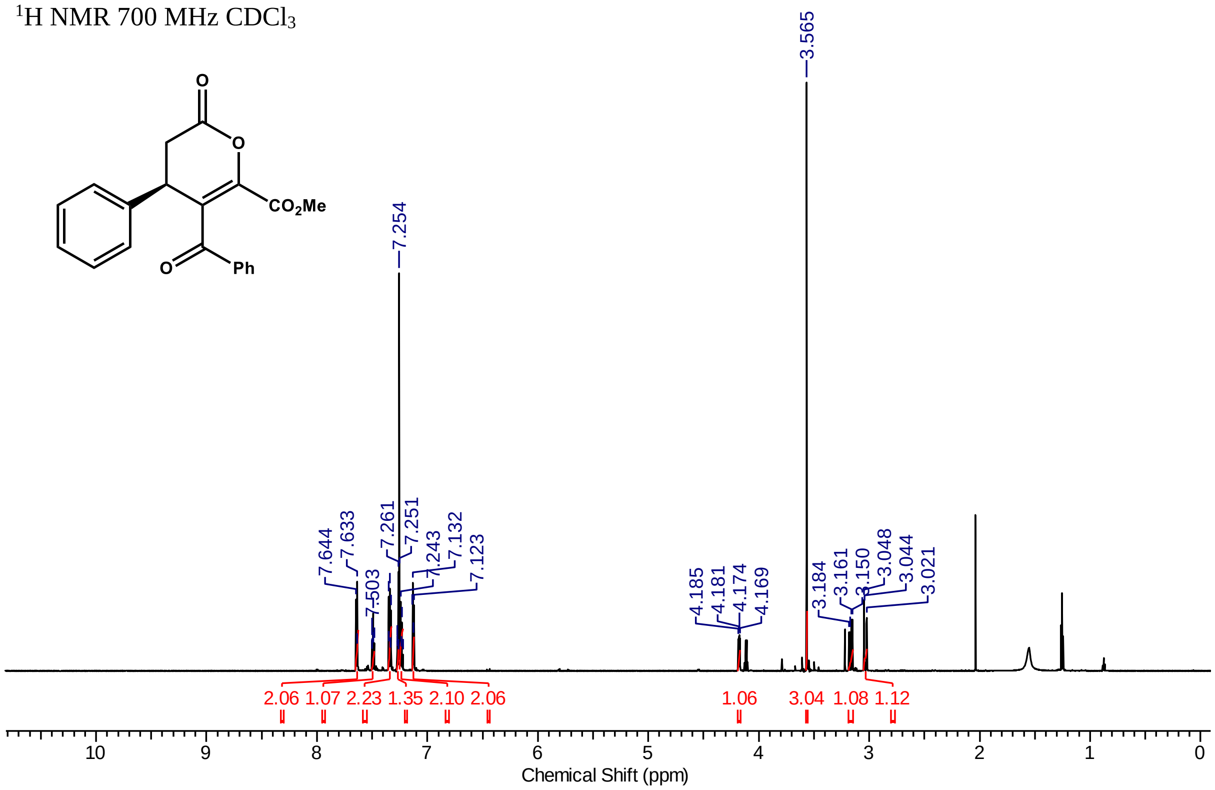

$^{13}C\{^1H\}$ NMR 101 MHz $CDCl_3$

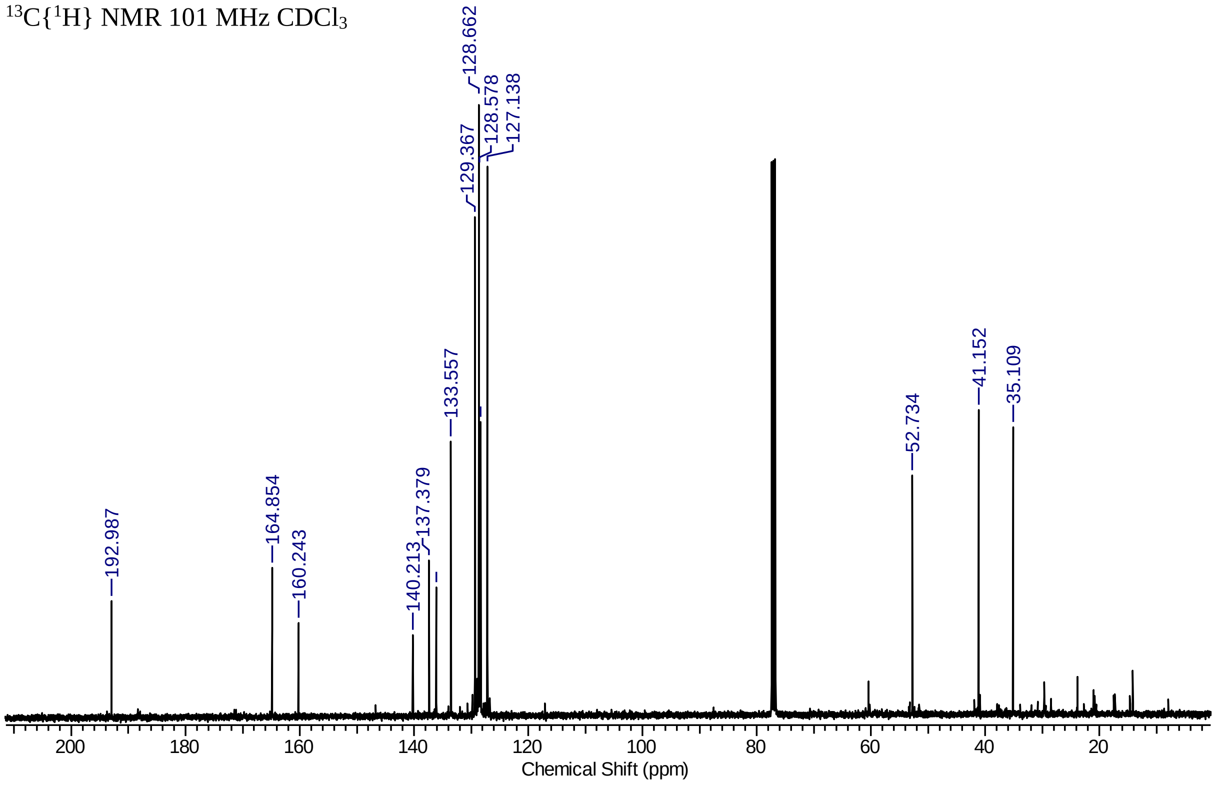

Figure S21. ^{1}H and ^{13}C NMR spectra of compound **3**.

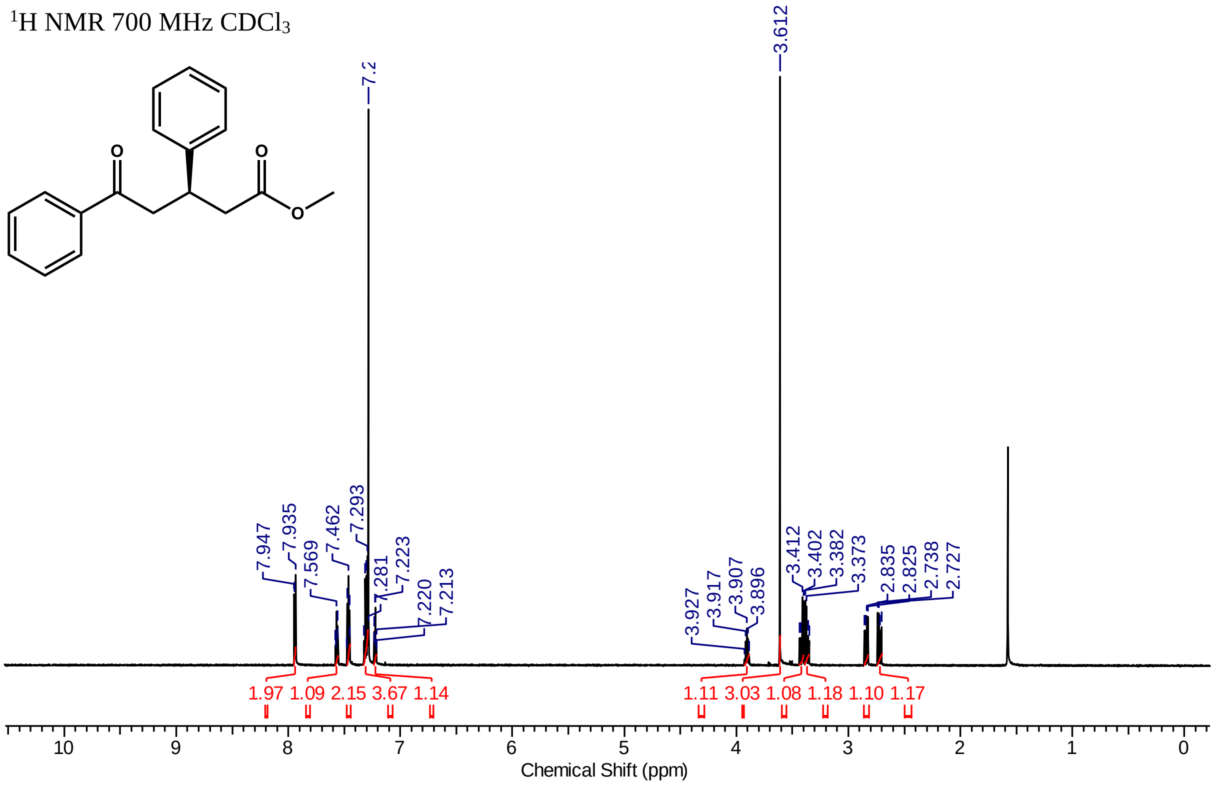

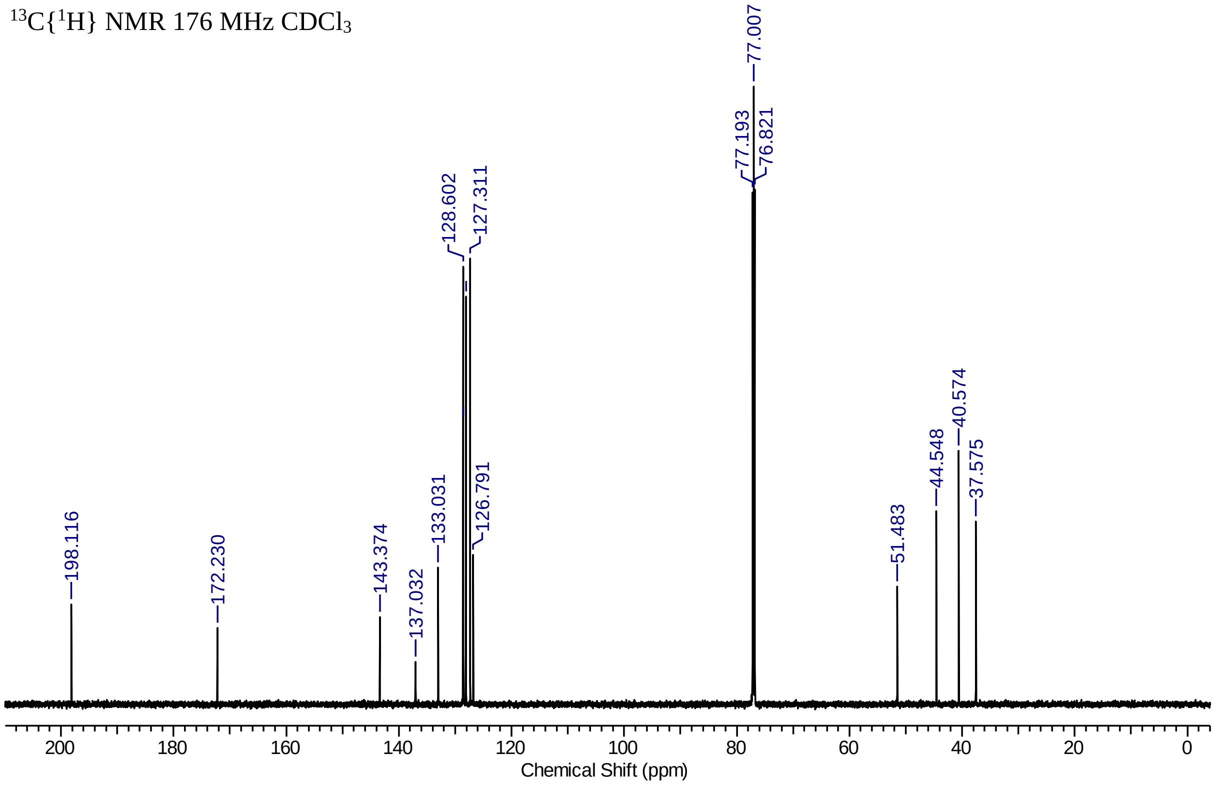

Figure S22. ^{1}H and ^{13}C NMR spectra of compound **4a**.

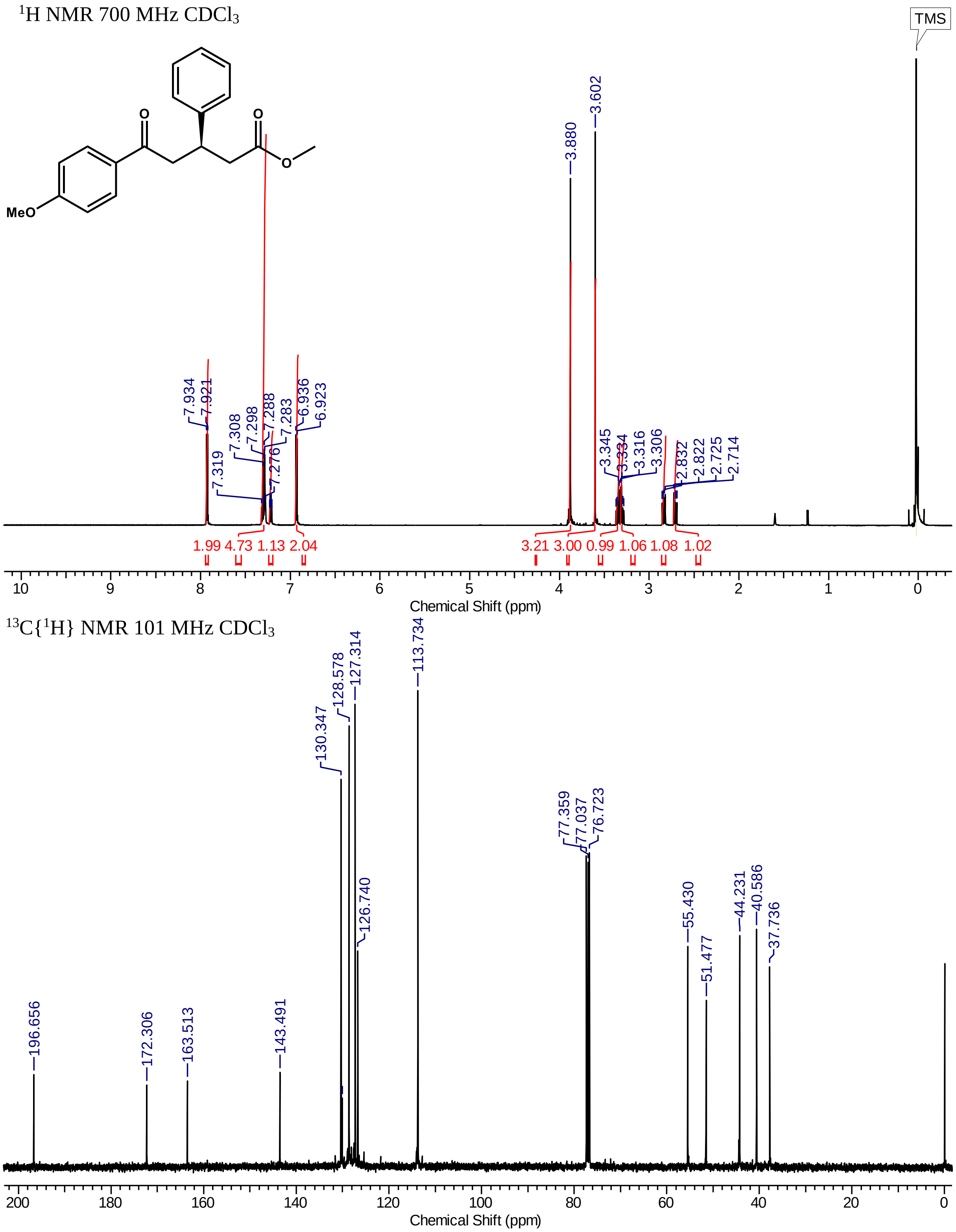

Figure S23. ^{1}H and ^{13}C NMR spectra of compound **4b**.

^{1}H NMR 700 MHz $CDCl_3$

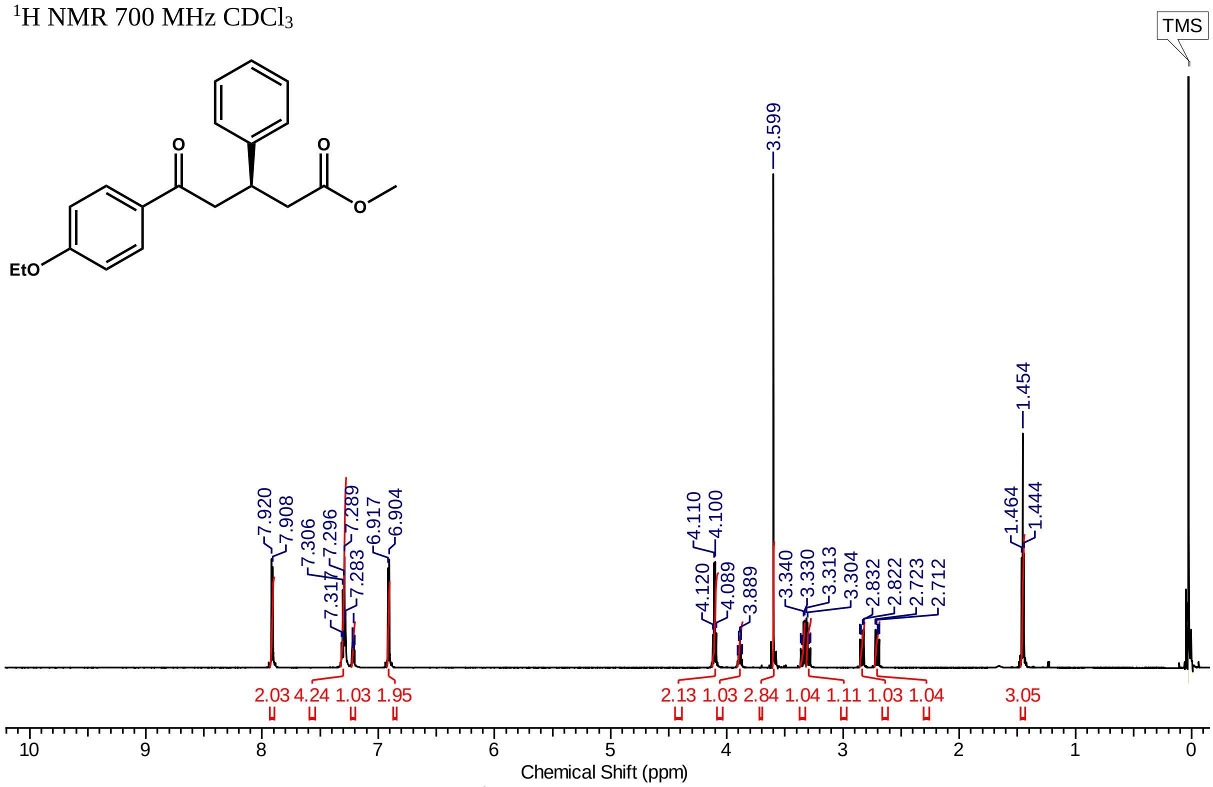

$^{13}C\{^{1}H\}$ NMR 176 MHz $CDCl_3$

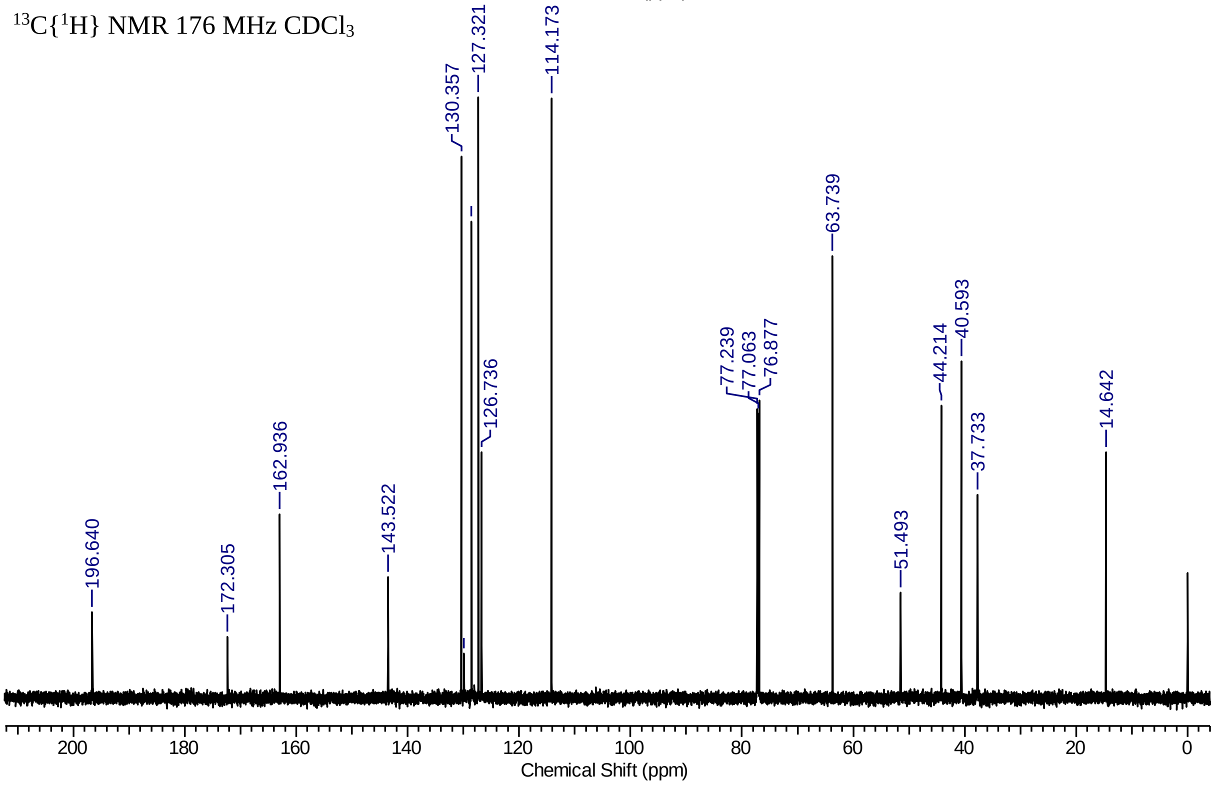

Figure S24. ^{1}H and ^{13}C NMR spectra of compound **4c**.

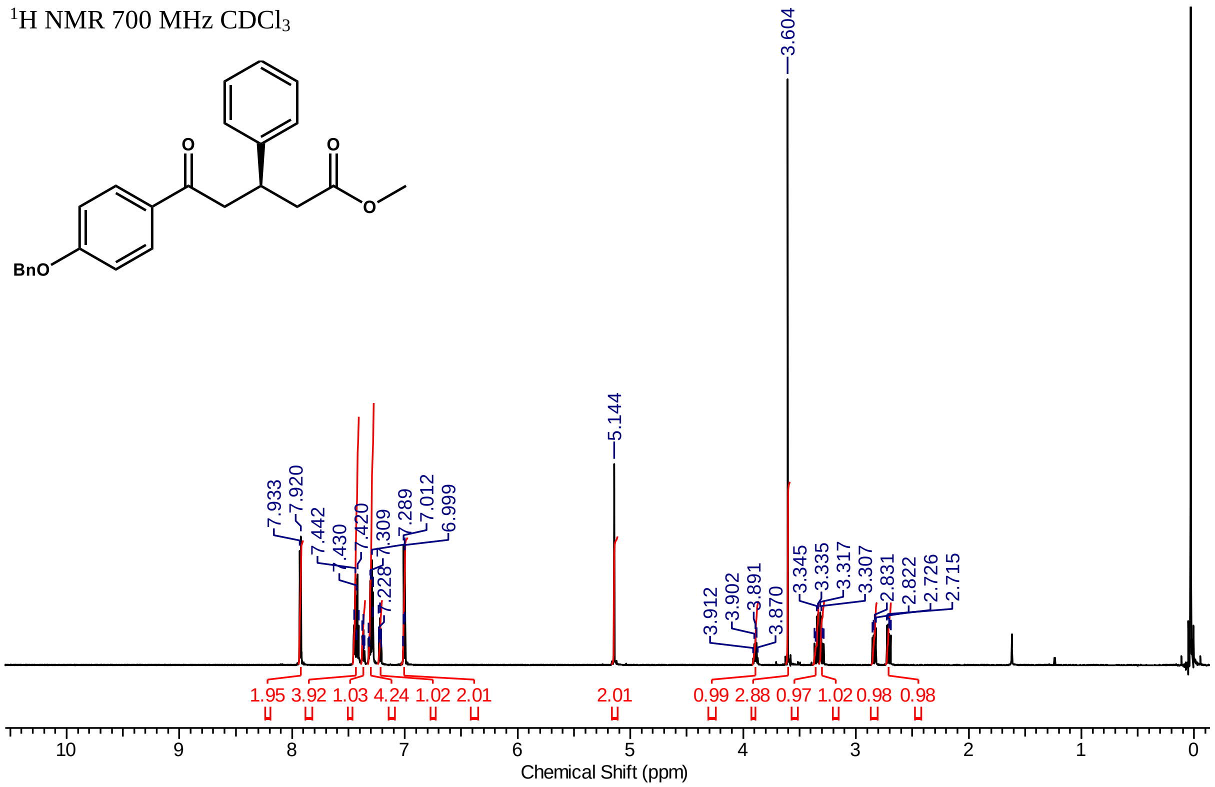

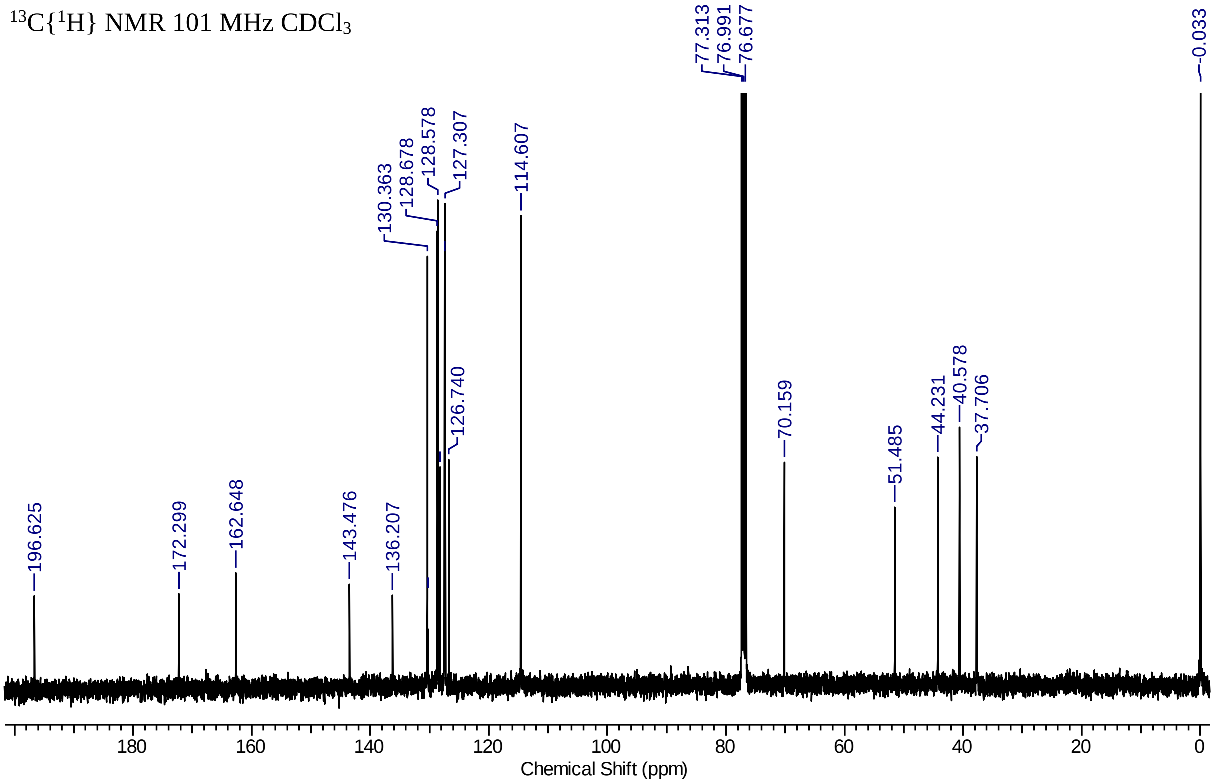

Figure S25. ^{1}H and ^{13}C NMR spectra of compound **4d**.

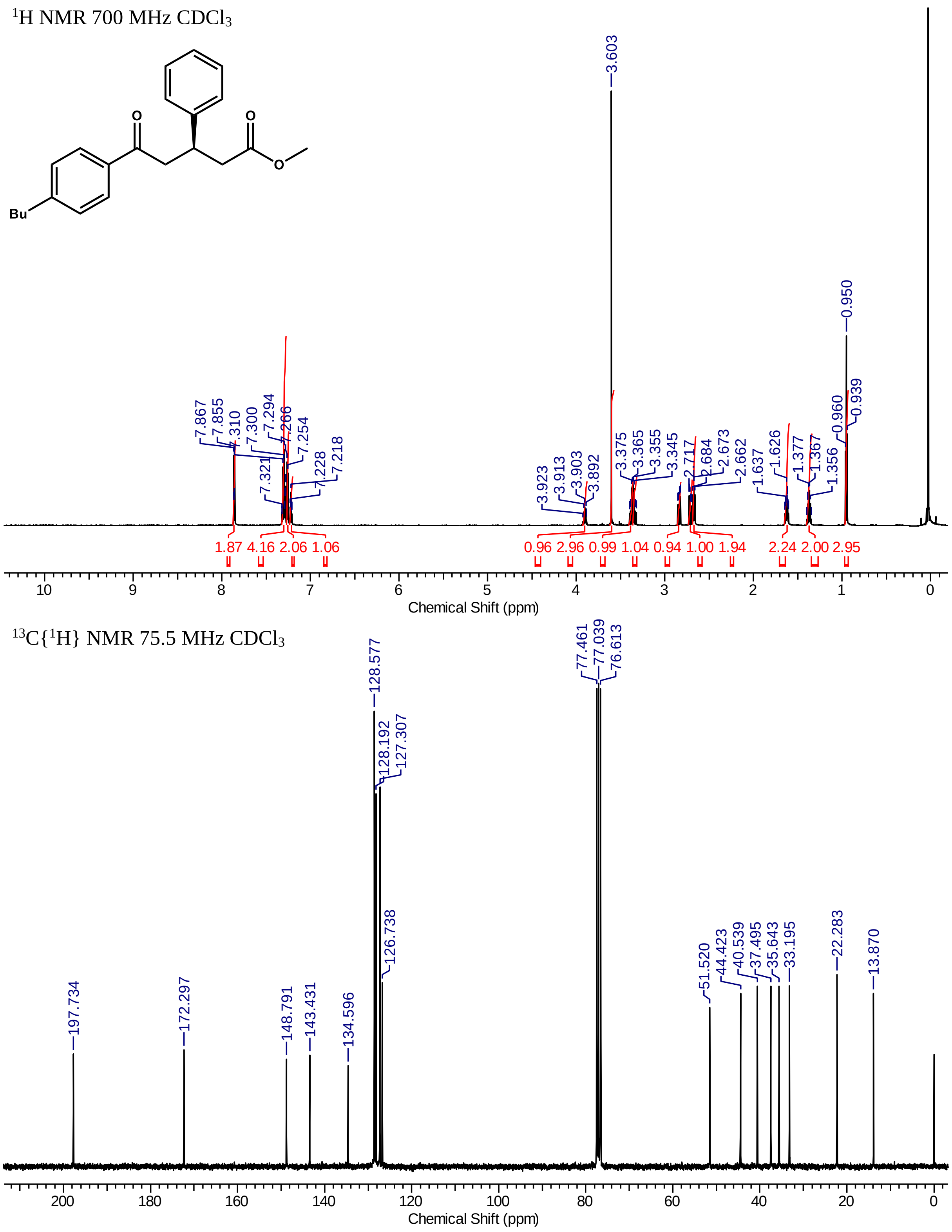

Figure S26. ^{1}H and ^{13}C NMR spectra of compound **4e**.

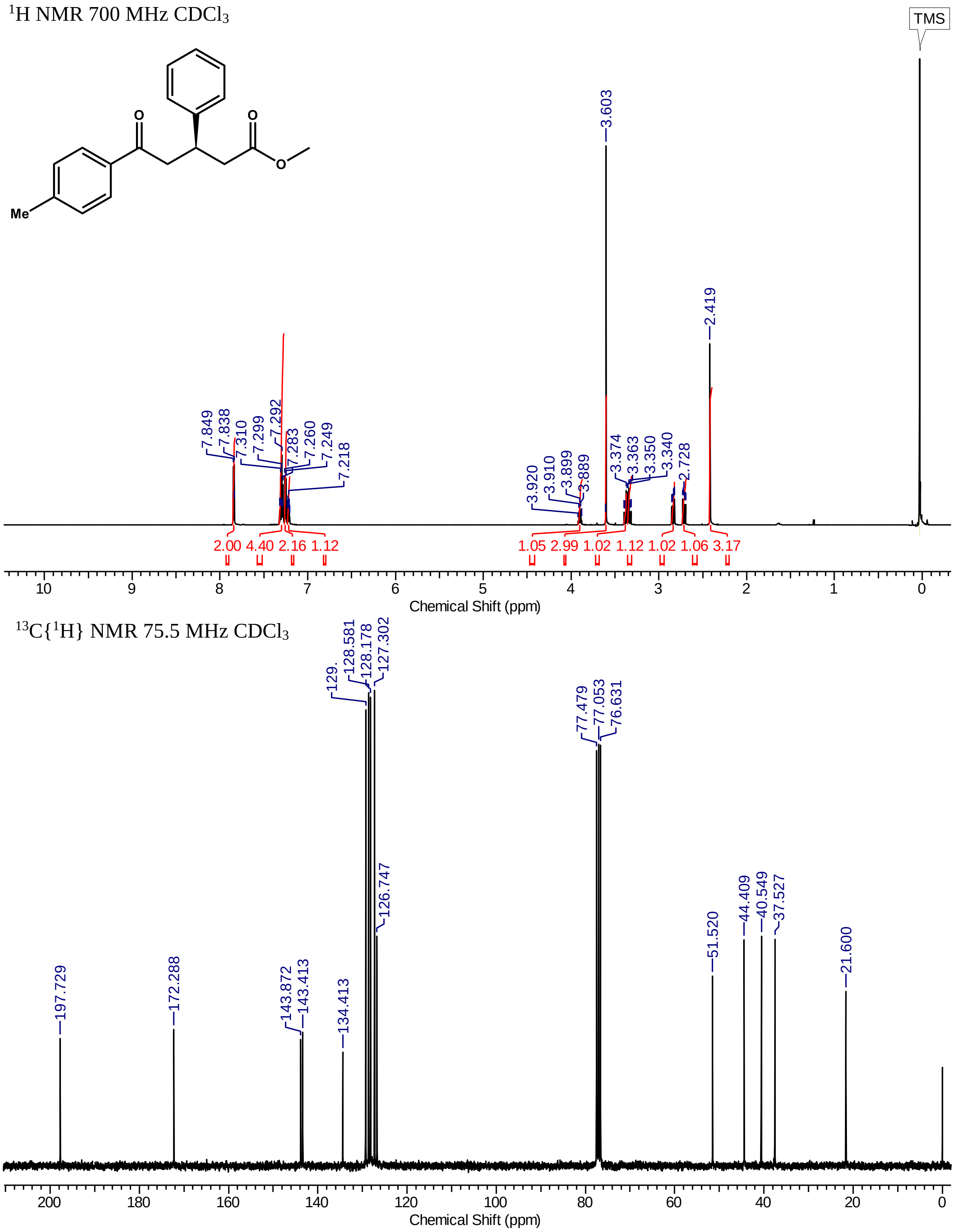

Figure S27. ^{1}H and ^{13}C NMR spectra of compound **4f**.

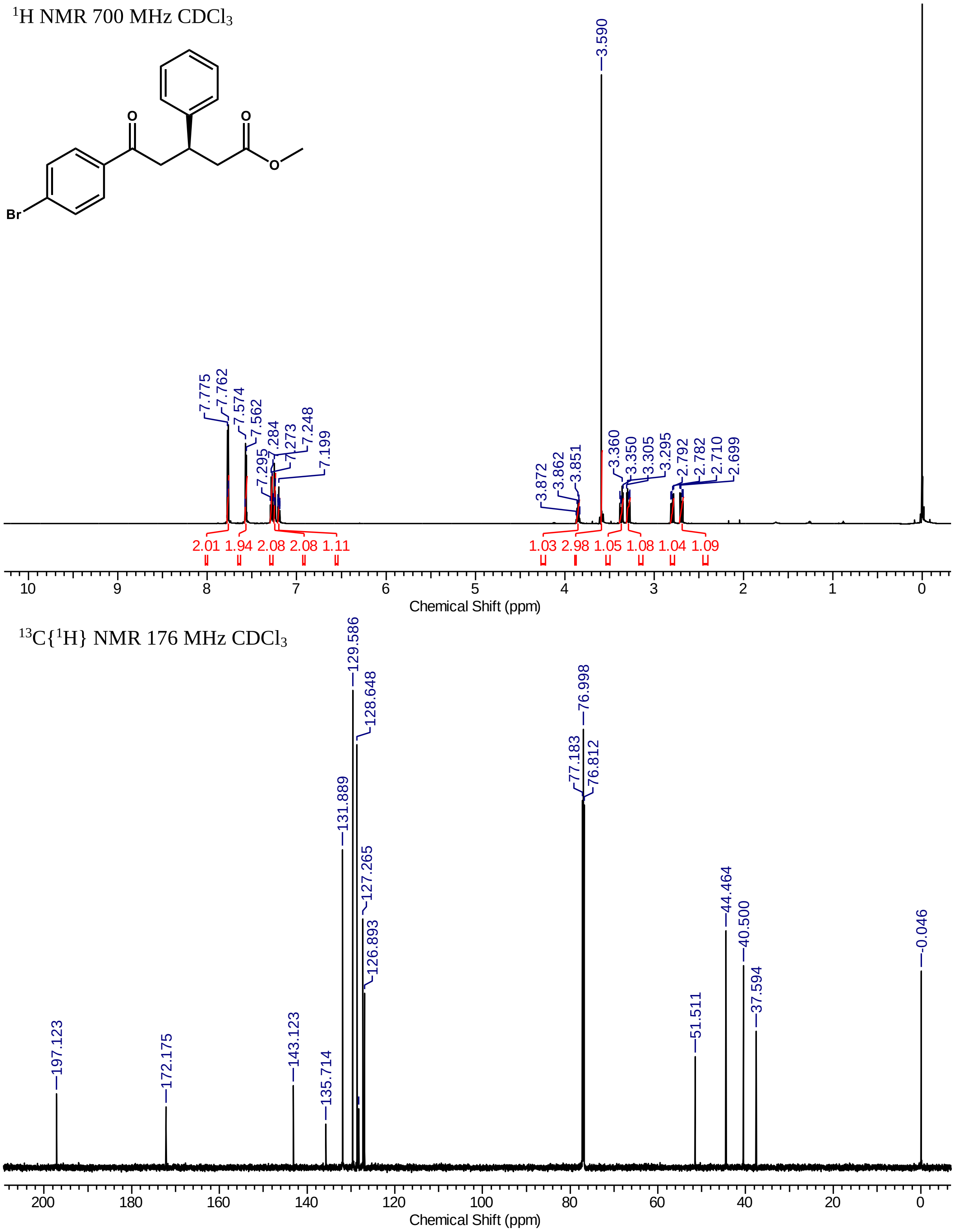

Figure S28. ^{1}H and ^{13}C NMR spectra of compound **4g**.

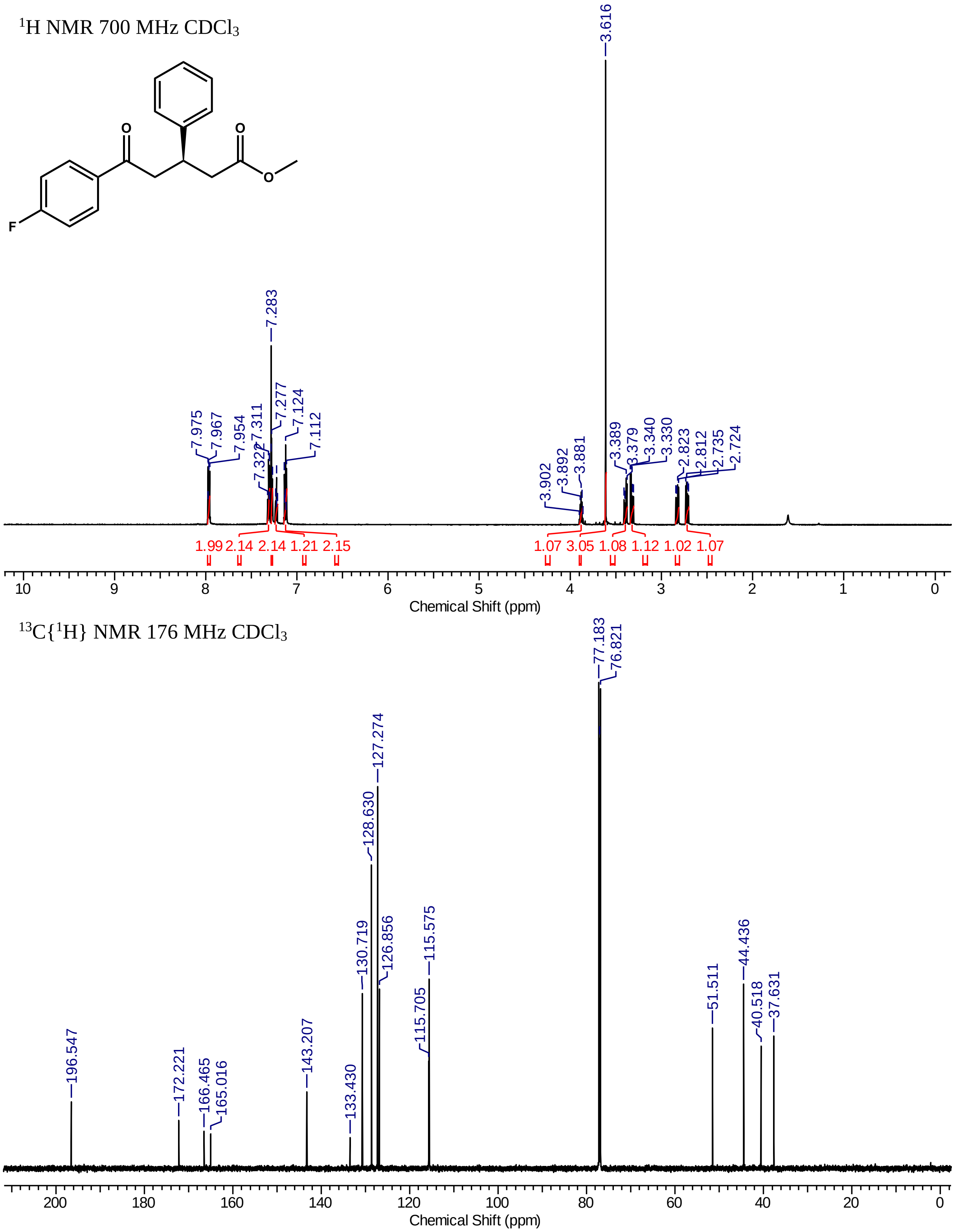

Figure S29. ^{1}H and ^{13}C NMR spectra of compound **4h**.

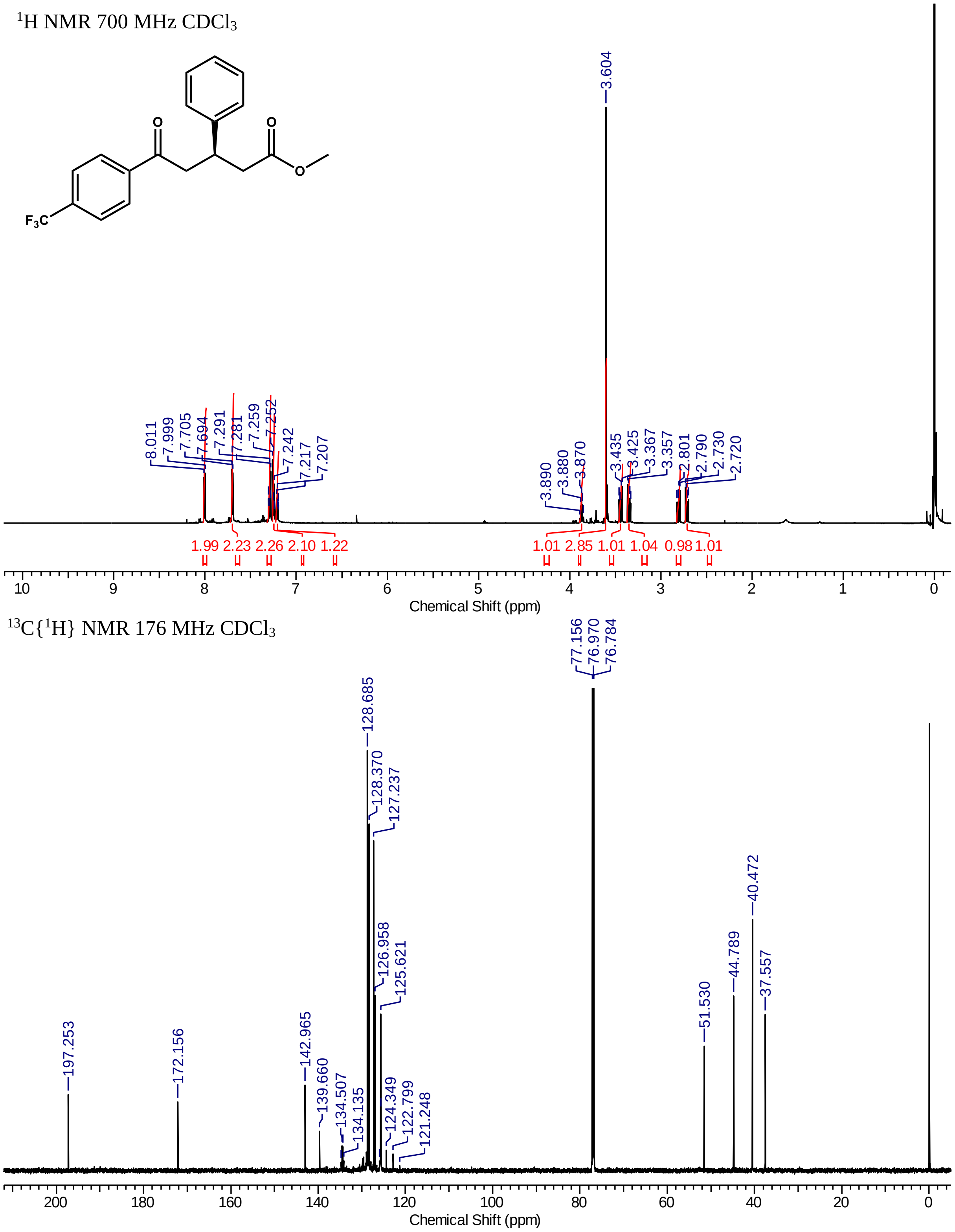

Figure S30. ^{1}H and ^{13}C NMR spectra of compound **4i**.

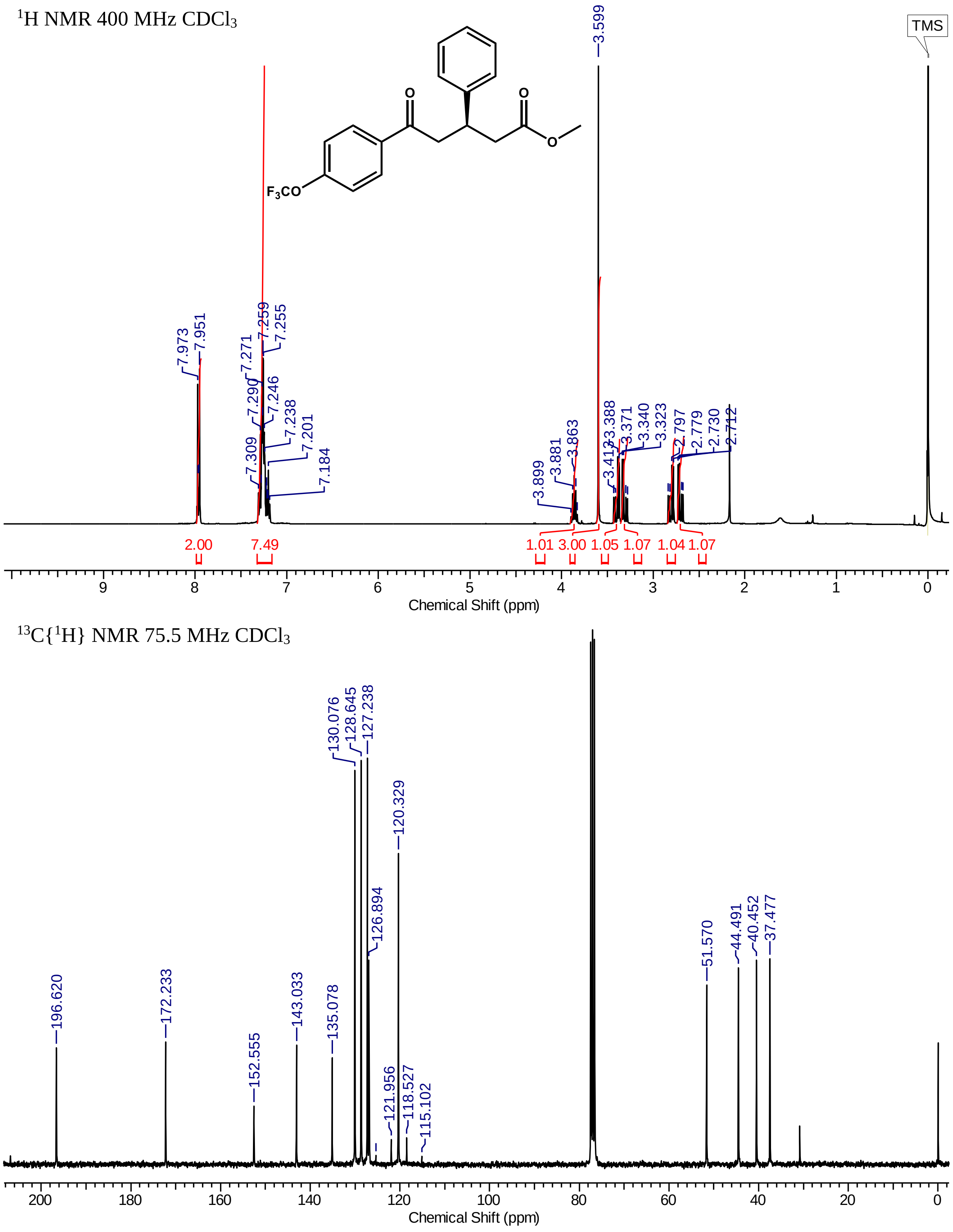

Figure S31. ^{1}H and ^{13}C NMR spectra of compound **4j**.

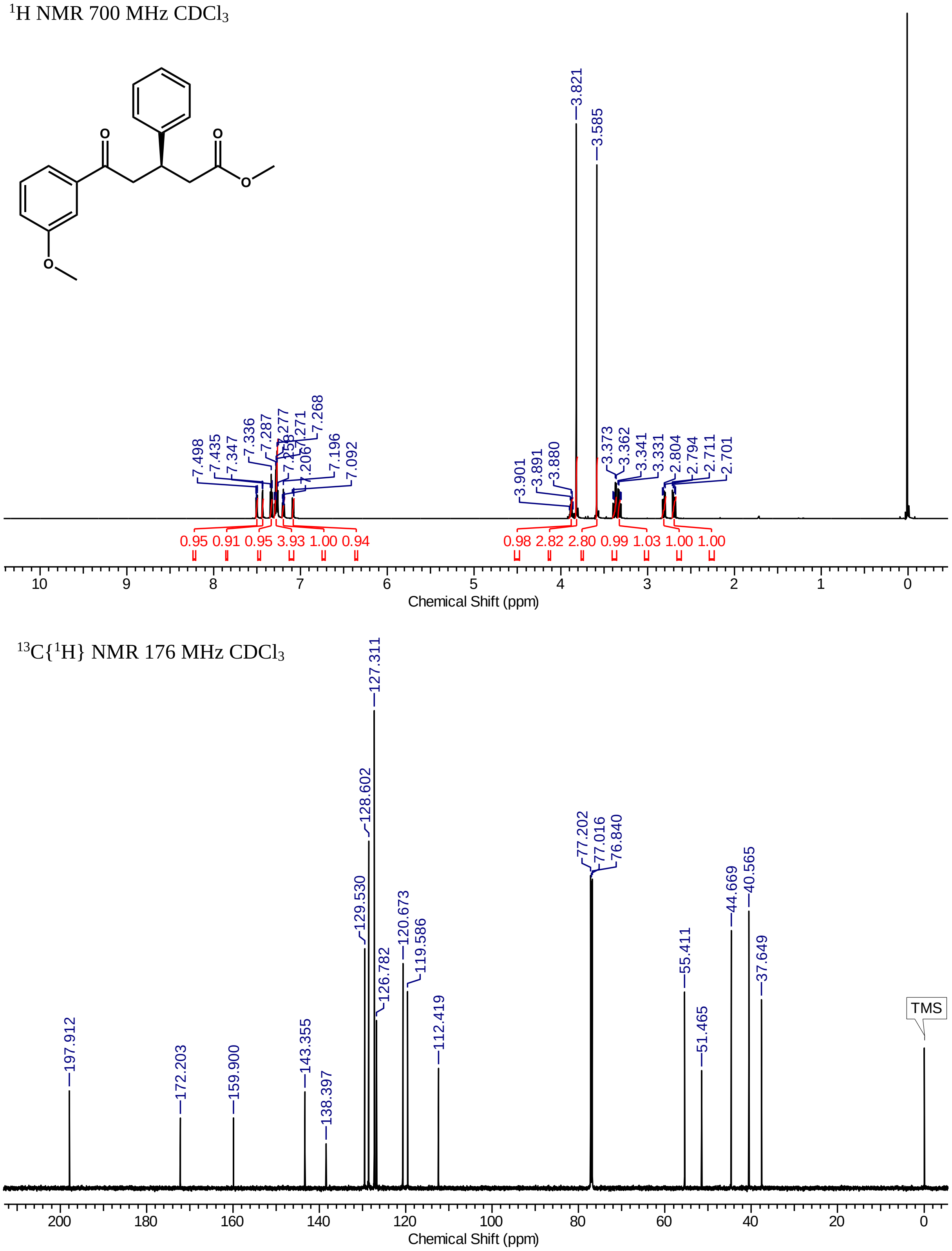

Figure S32. ^{1}H and ^{13}C NMR spectra of compound **4k**.

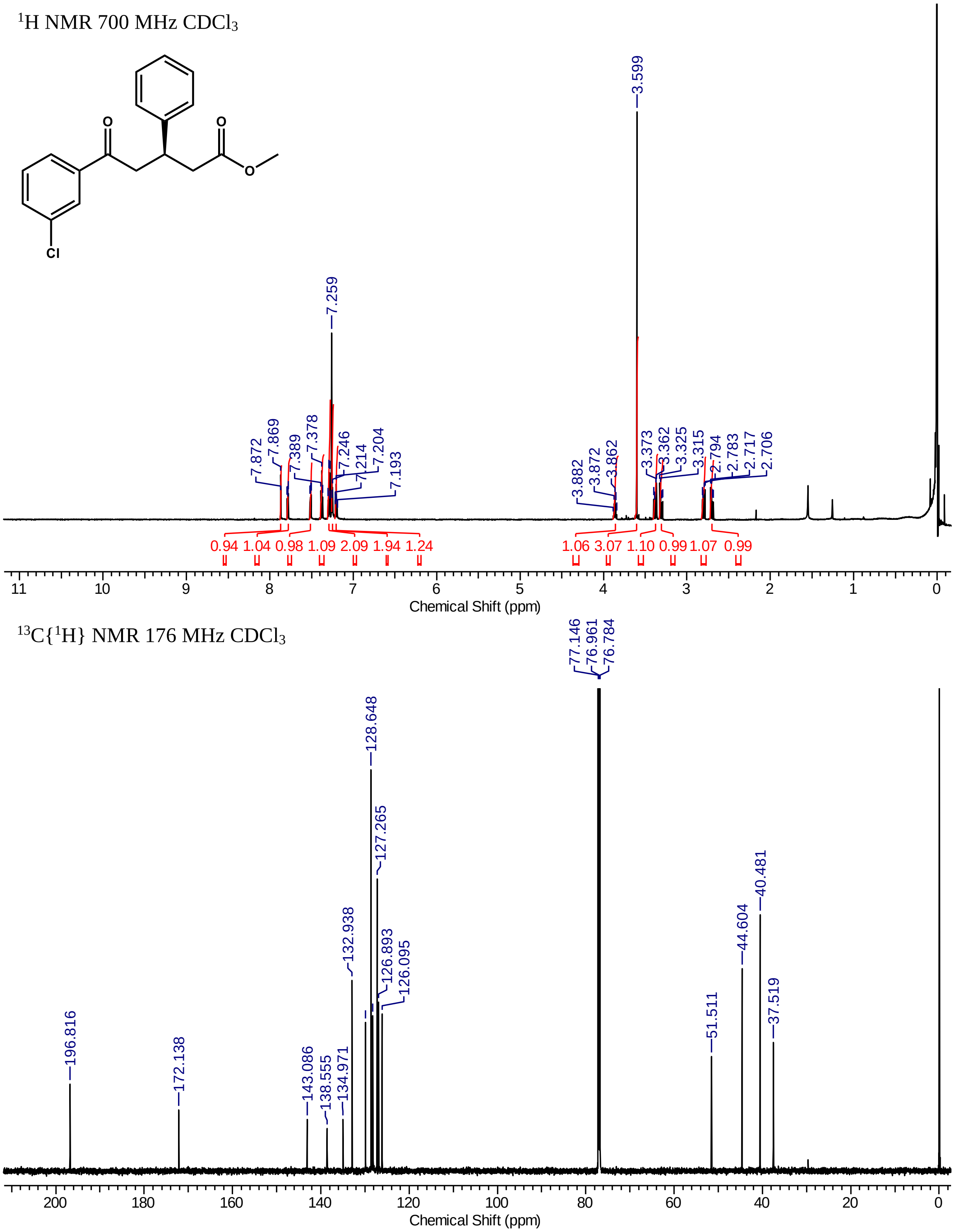

Figure S33. ^{1}H and ^{13}C NMR spectra of compound **4l**.

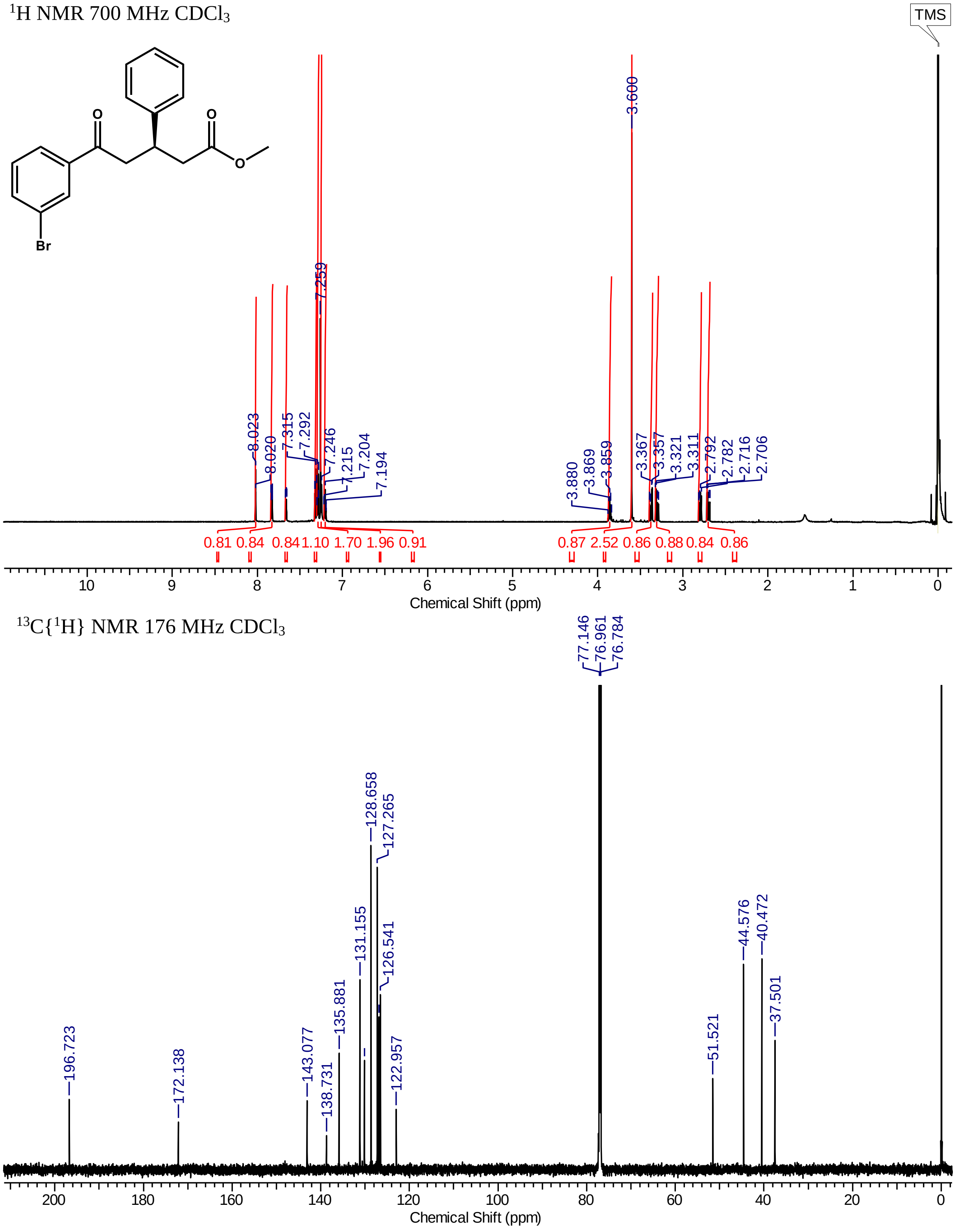

Figure S34. ^{1}H and ^{13}C NMR spectra of compound **4m**.

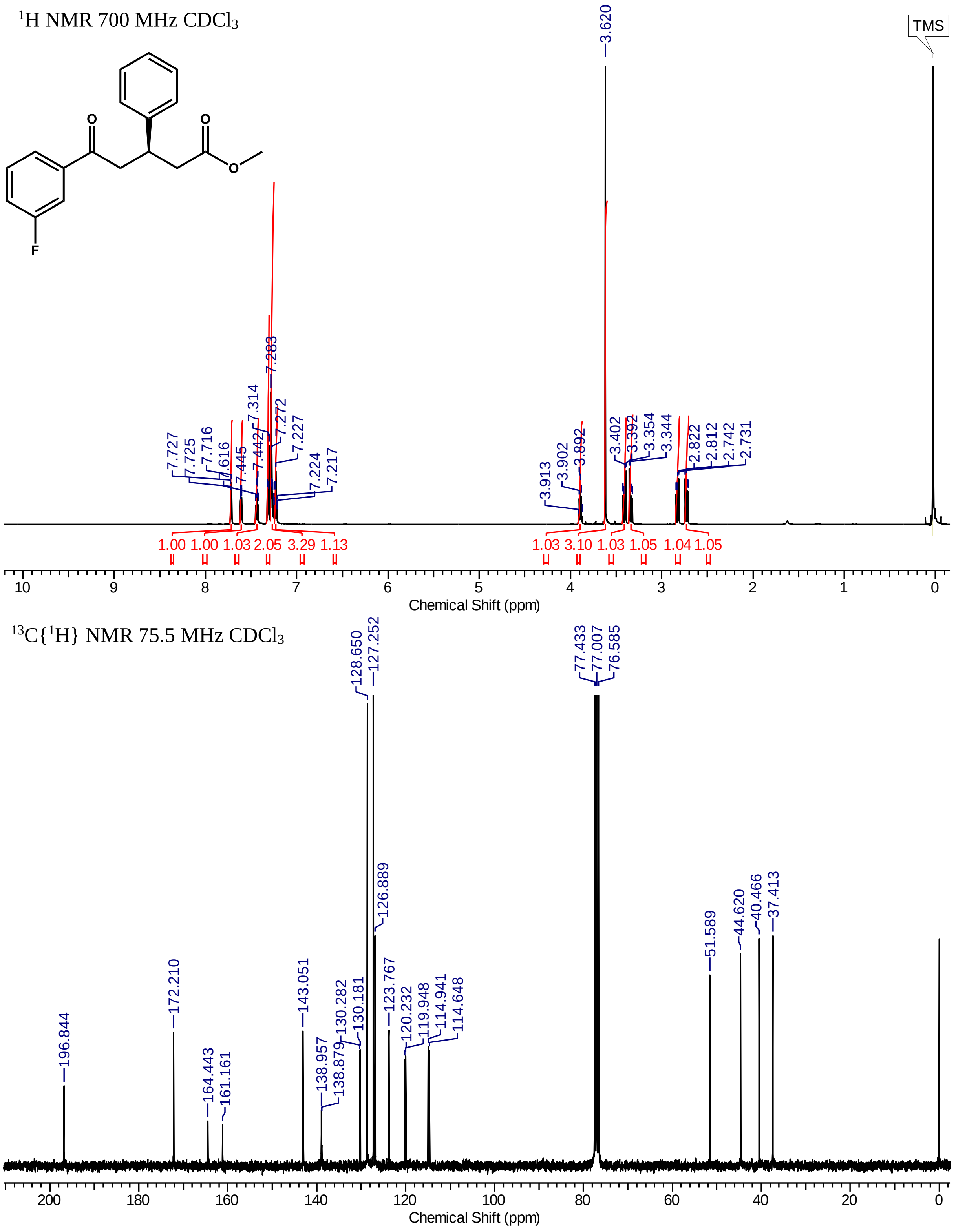

Figure S35. ^{1}H and ^{13}C NMR spectra of compound **4n**.

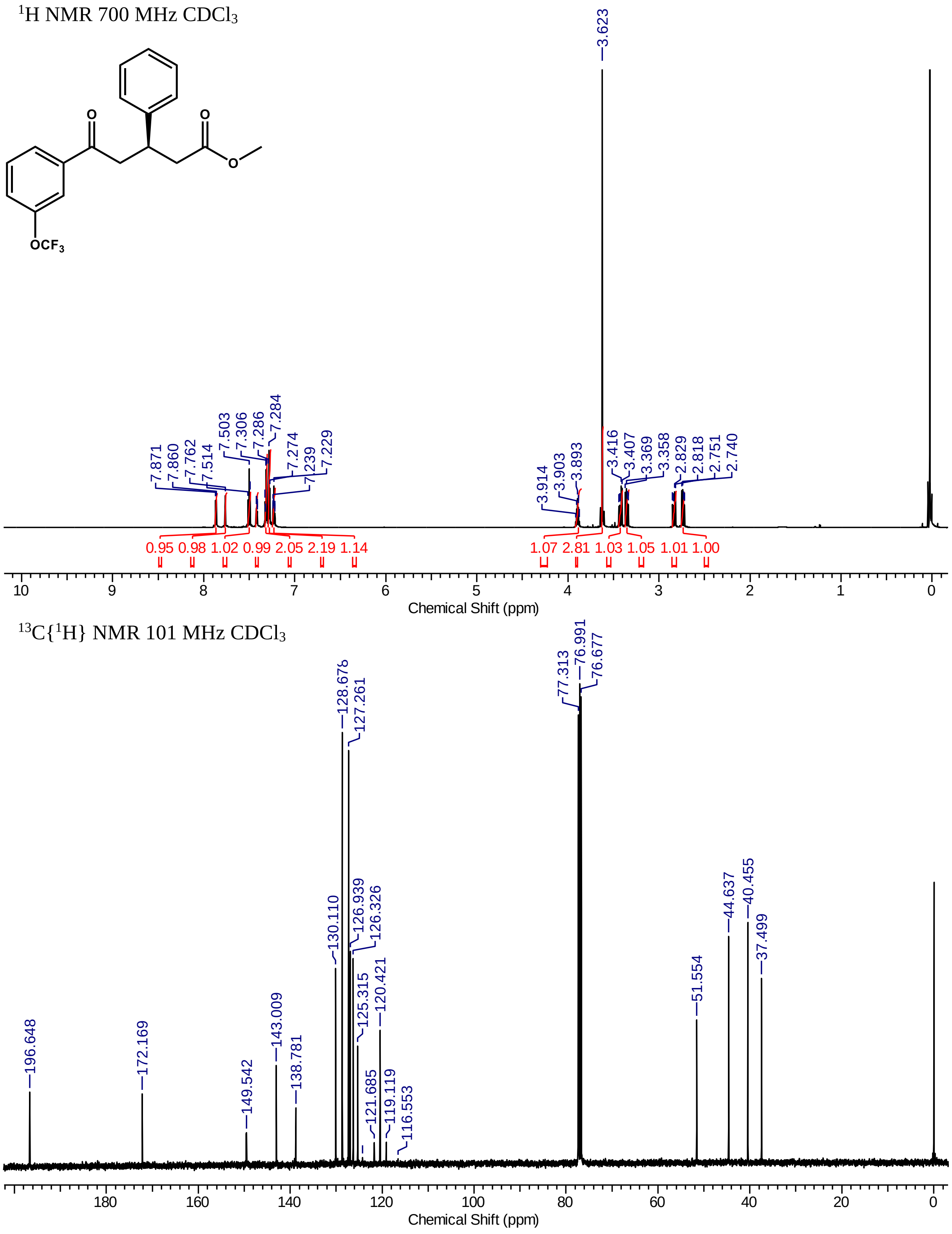

Figure S36. ^{1}H and ^{13}C NMR spectra of compound **4o**.

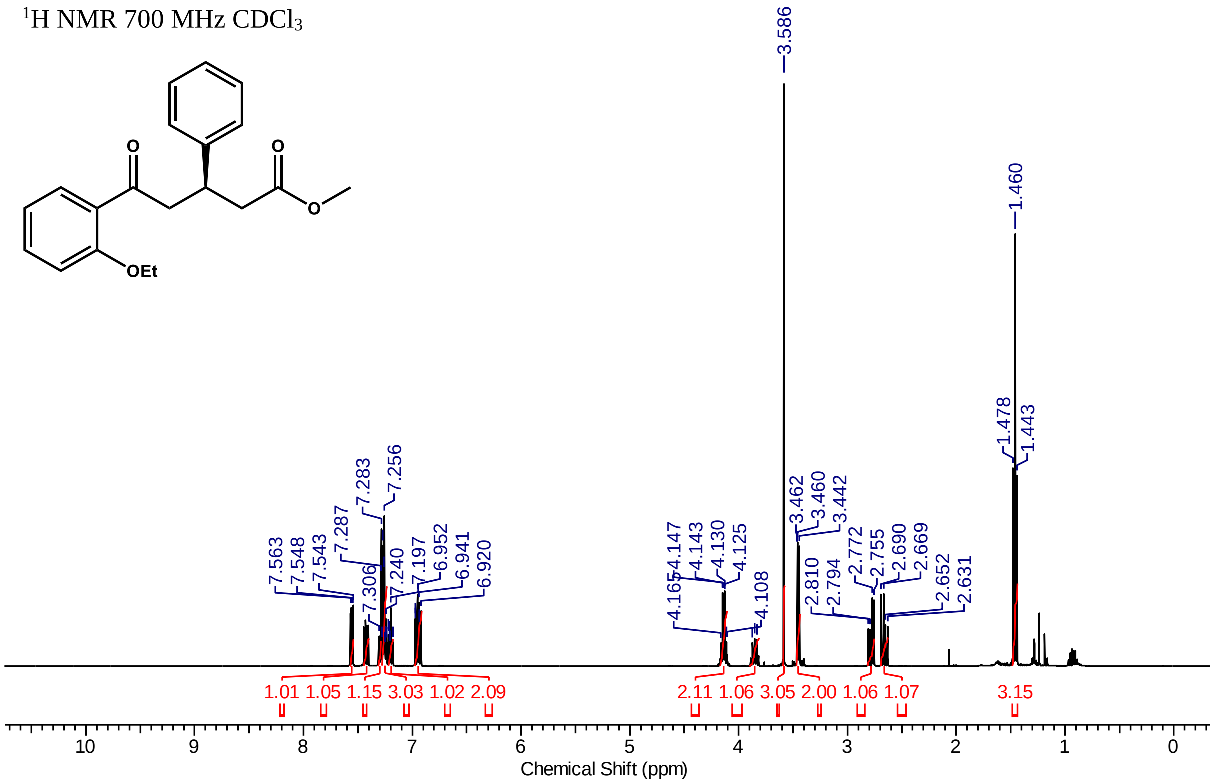

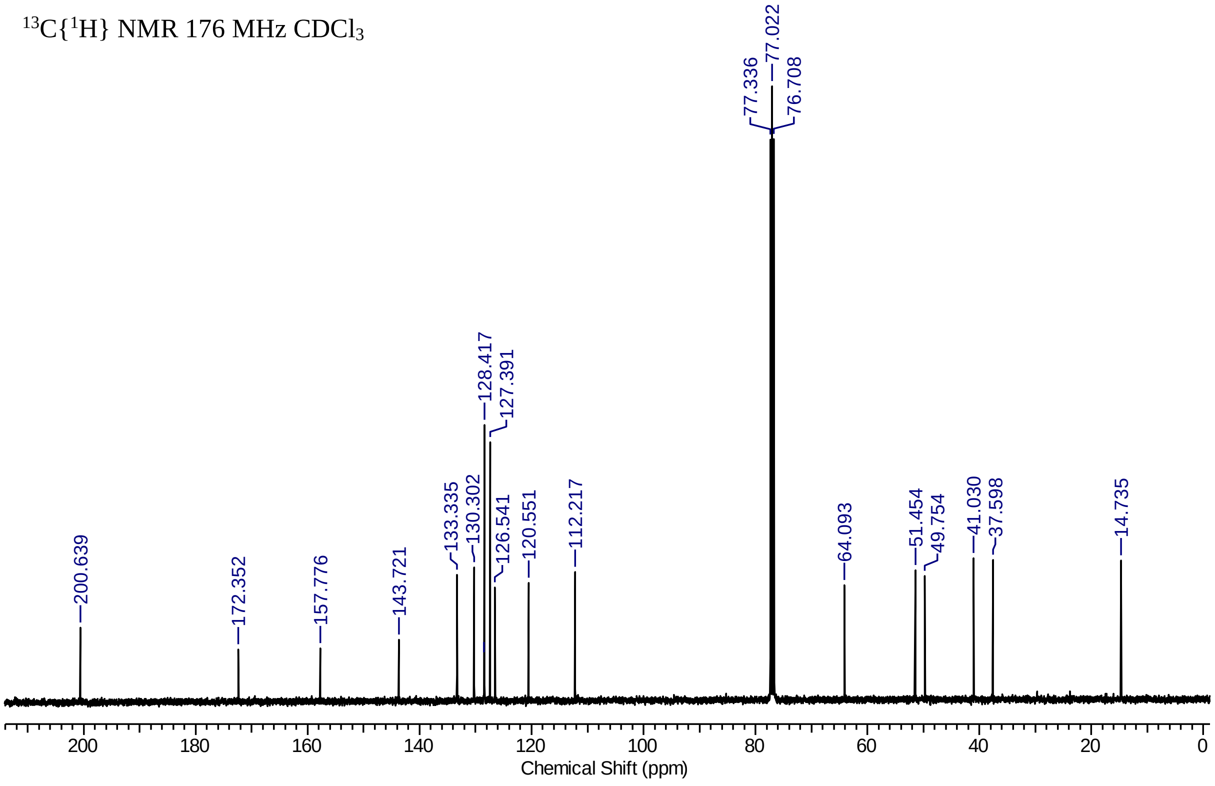

Figure S37. ^{1}H and ^{13}C NMR spectra of compound **4p**.

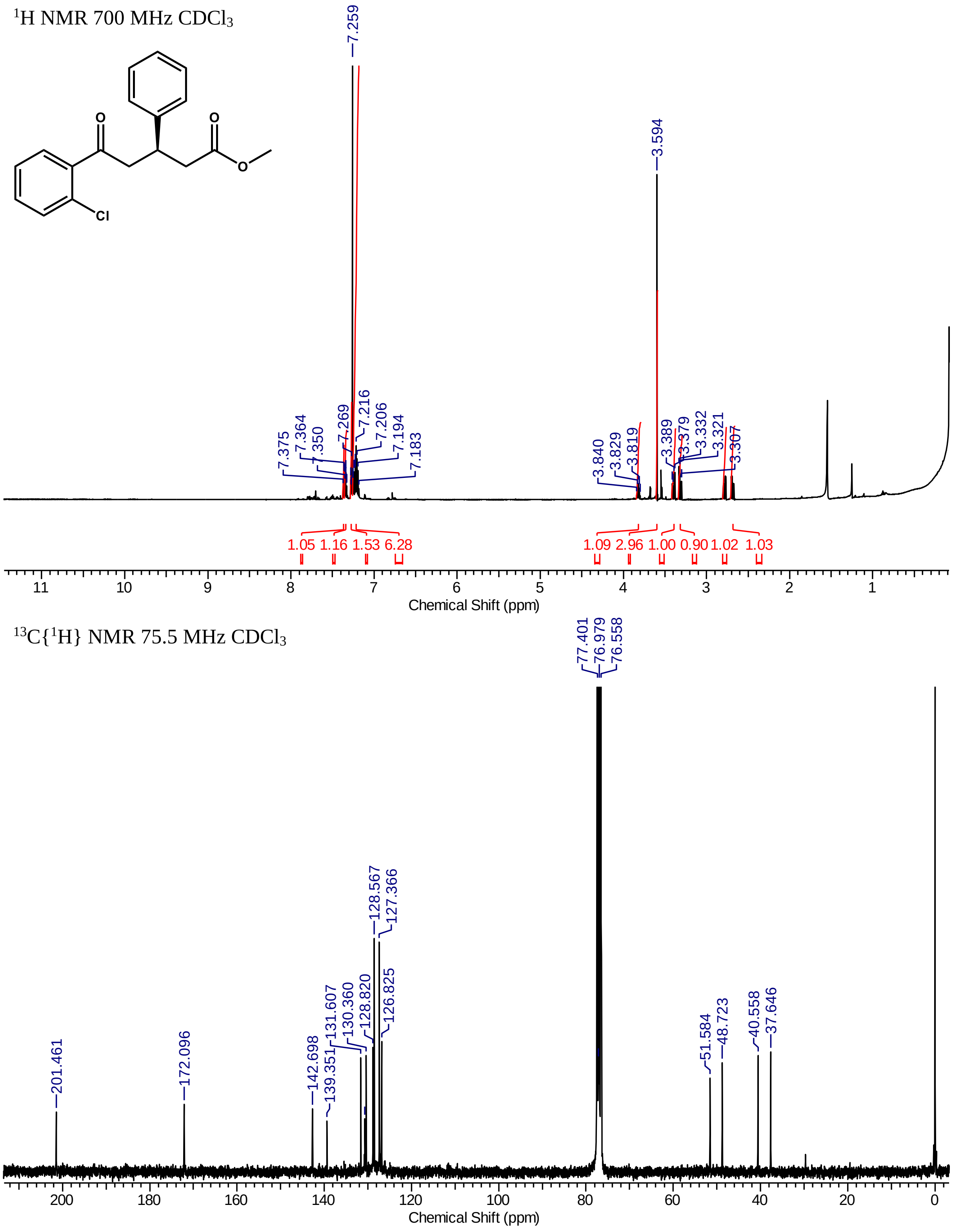

Figure S38. ^{1}H and ^{13}C NMR spectra of compound **4q**.

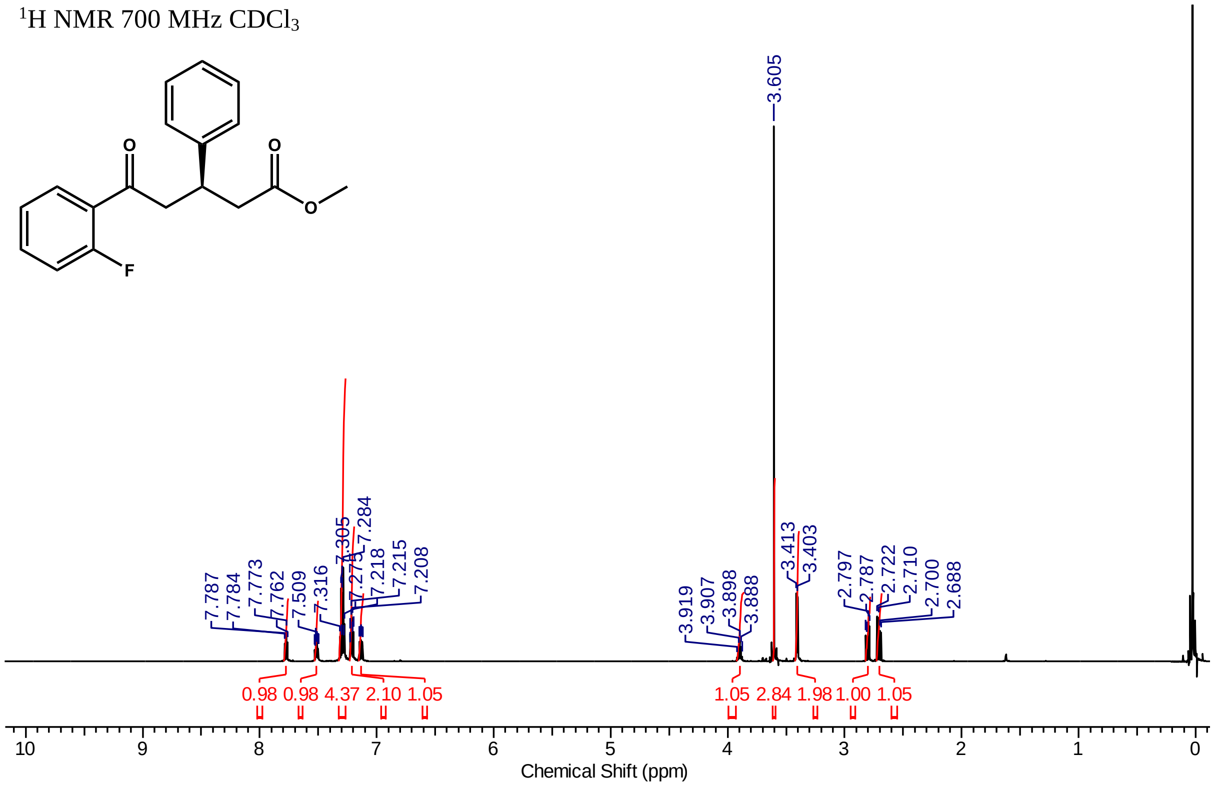

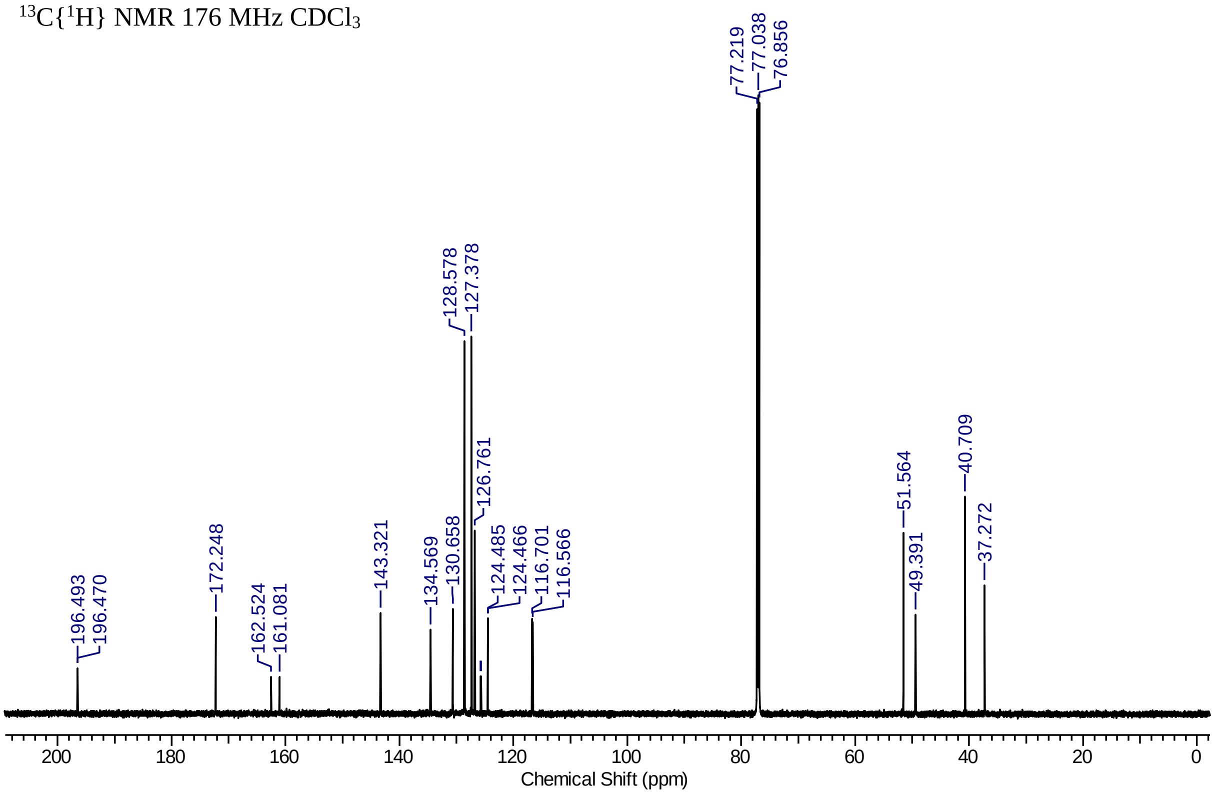

Figure S39. ^{1}H and ^{13}C NMR spectra of compound **4r**.

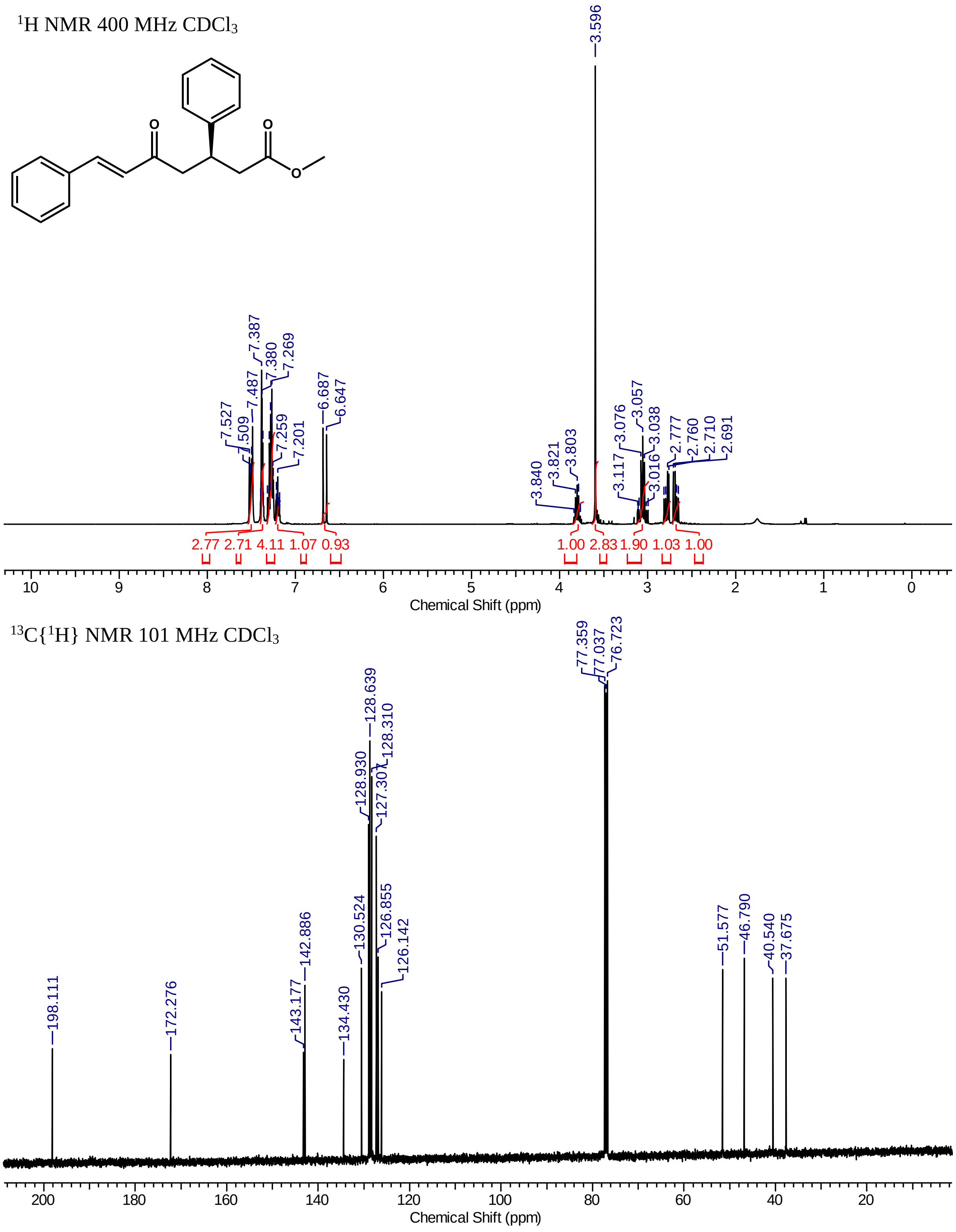

Figure S40. ^{1}H and ^{13}C NMR spectra of compound **4s**.

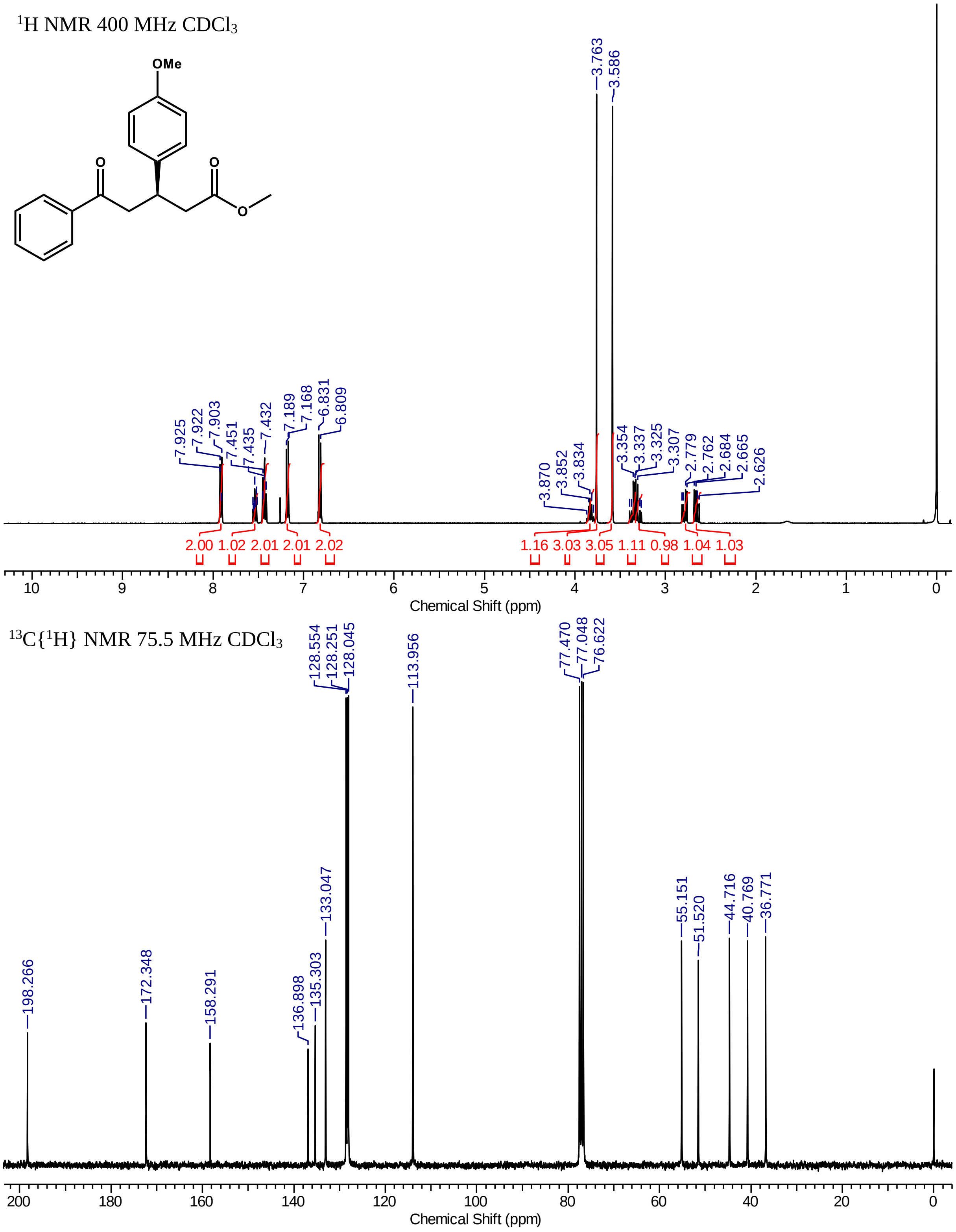

Figure S41. ^{1}H and ^{13}C NMR spectra of compound **4t**.

^{1}H NMR 400 MHz $CDCl_3$

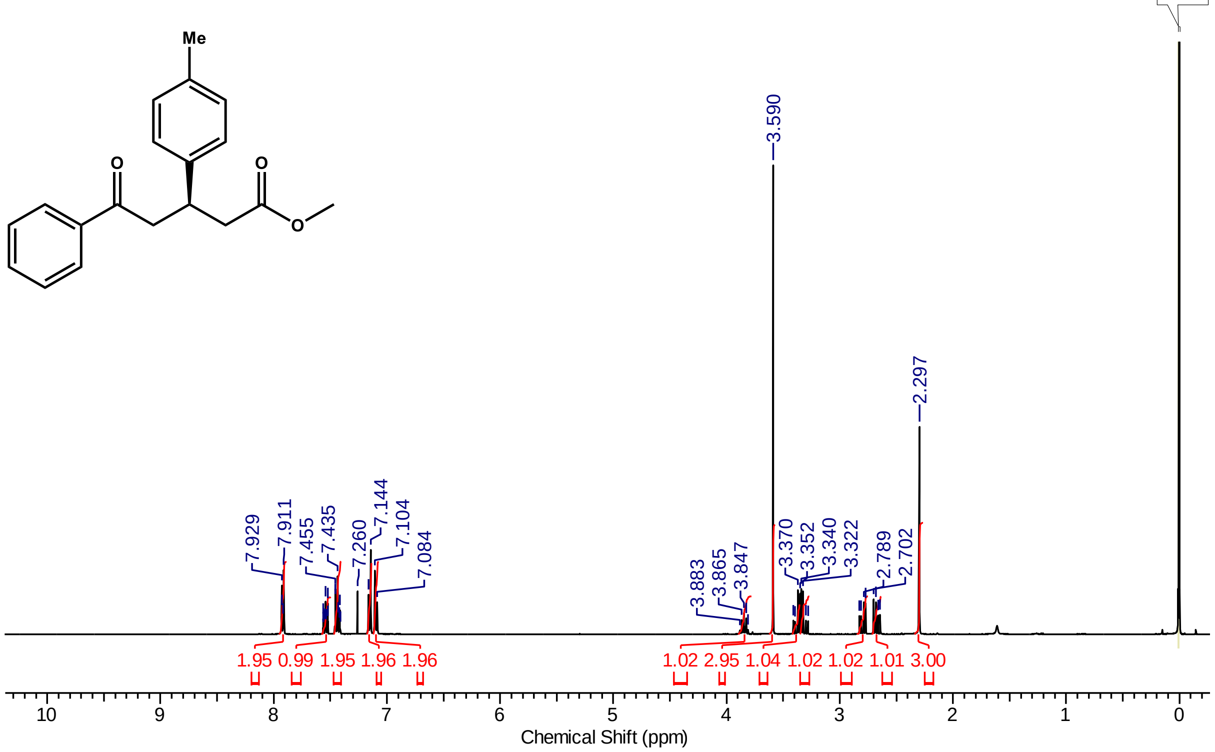

$^{13}C\{^{1}H\}$ NMR 75.5 MHz $CDCl_3$

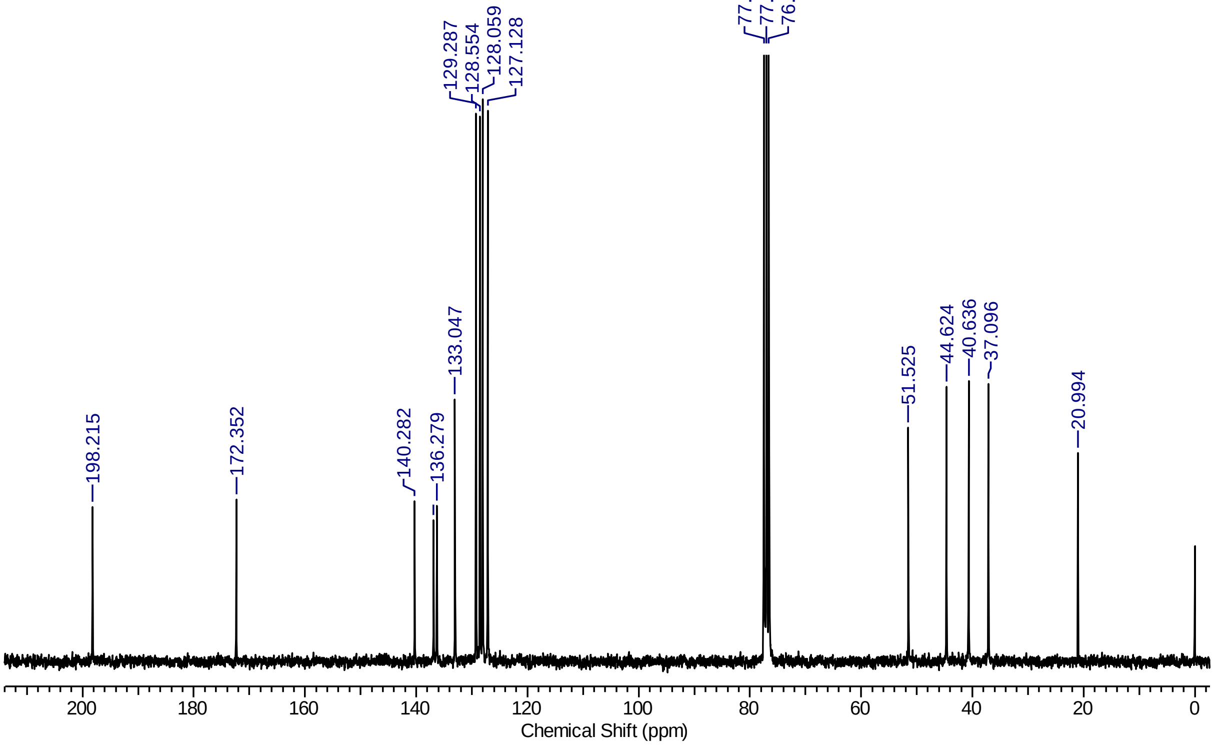

Figure S42. ^{1}H and ^{13}C NMR spectra of compound **4u**.

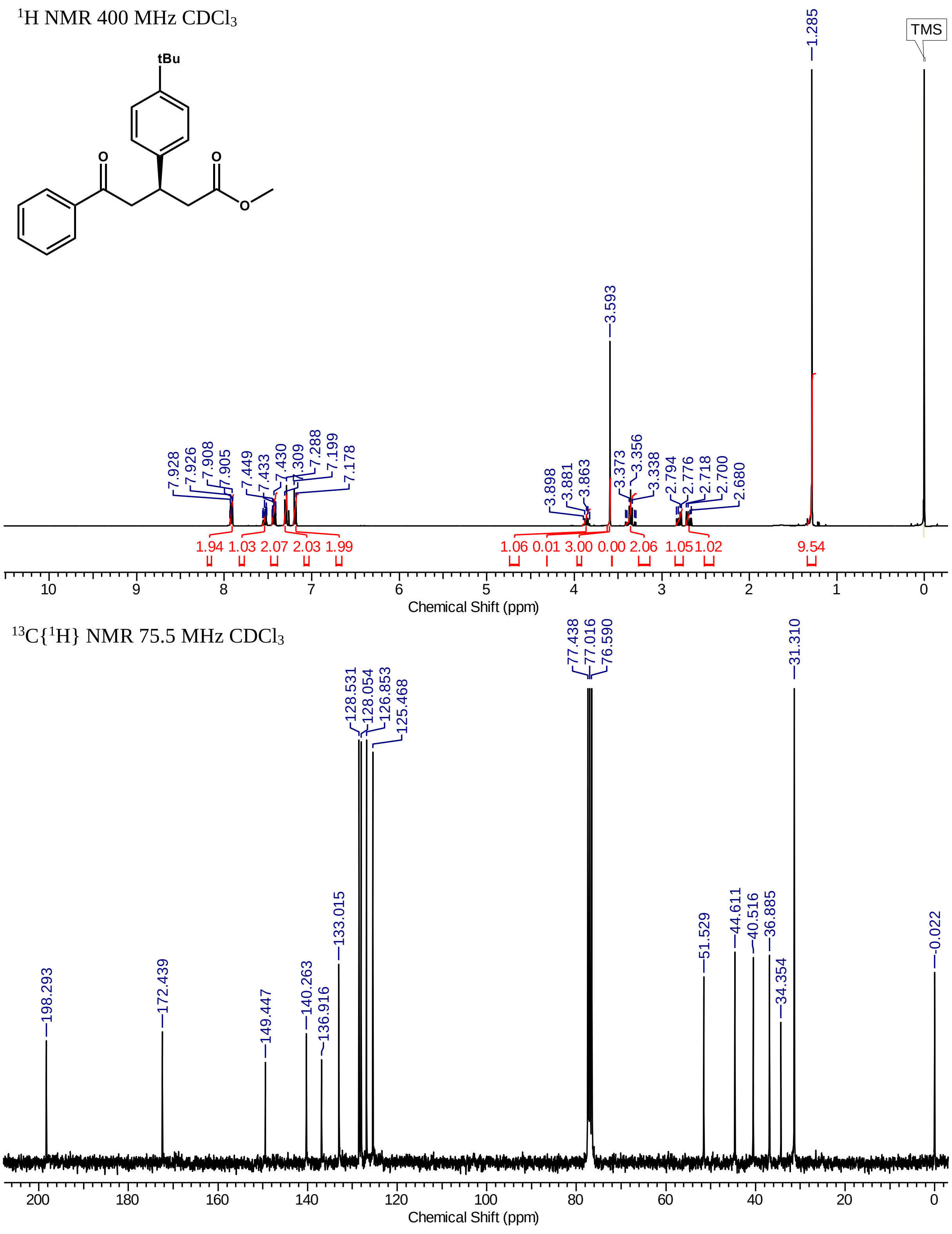

Figure S43. ^{1}H and ^{13}C NMR spectra of compound **4v**.

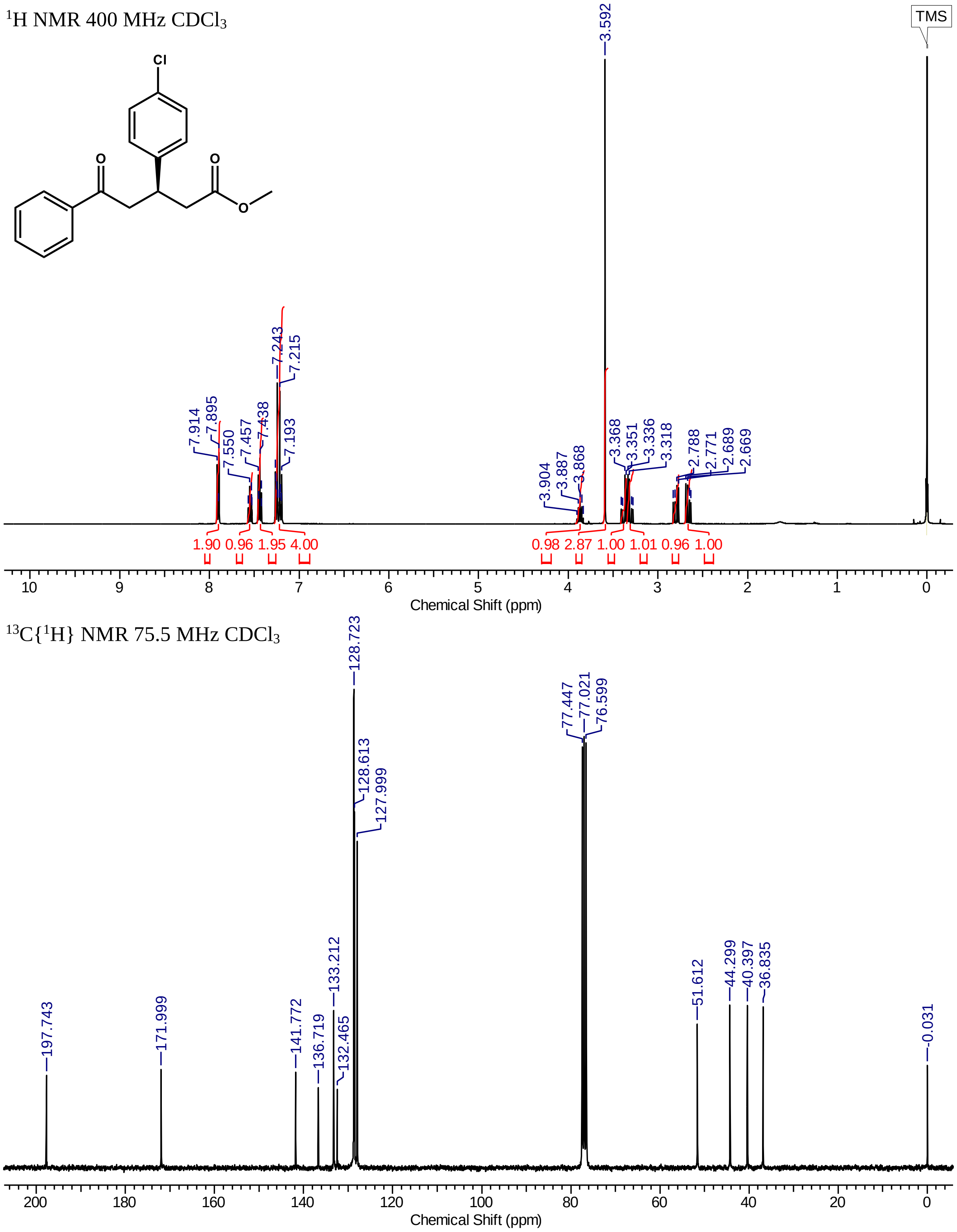

Figure S44. ^{1}H and ^{13}C NMR spectra of compound **4w**.

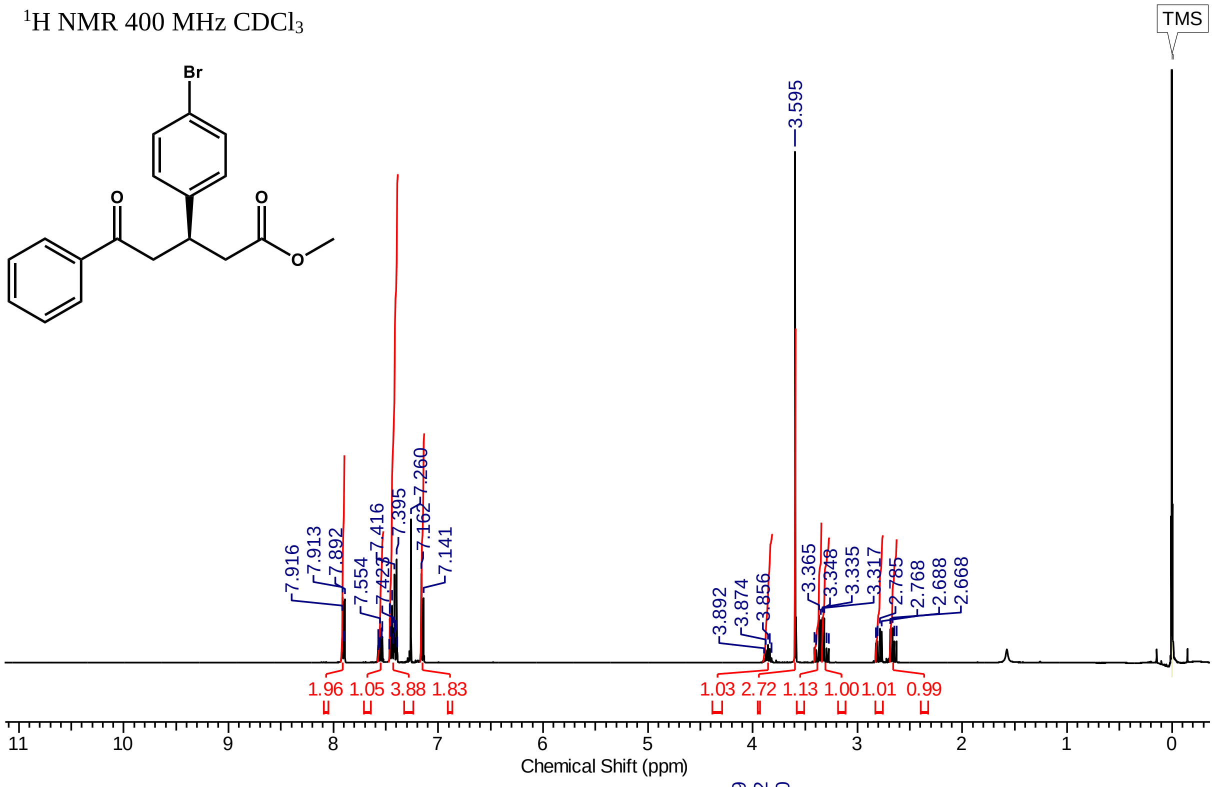

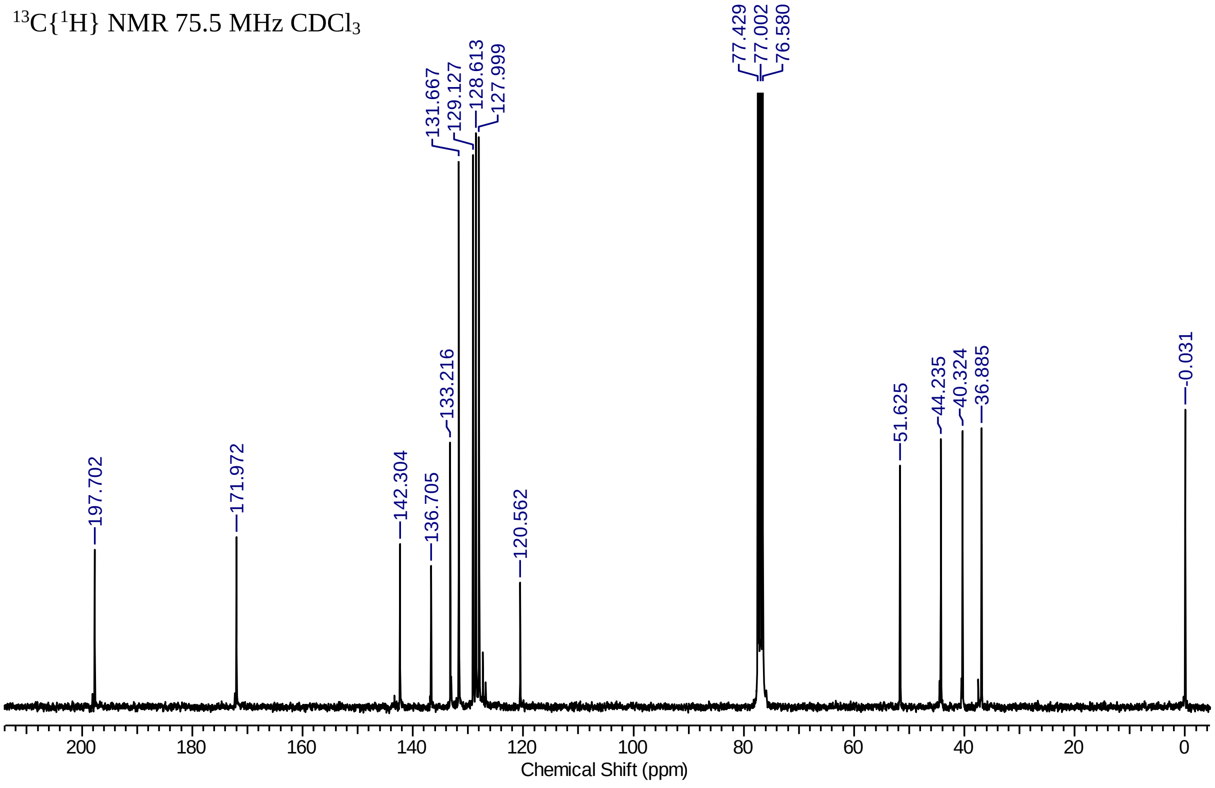

Figure S45. ^{1}H and ^{13}C NMR spectra of compound **4x**.

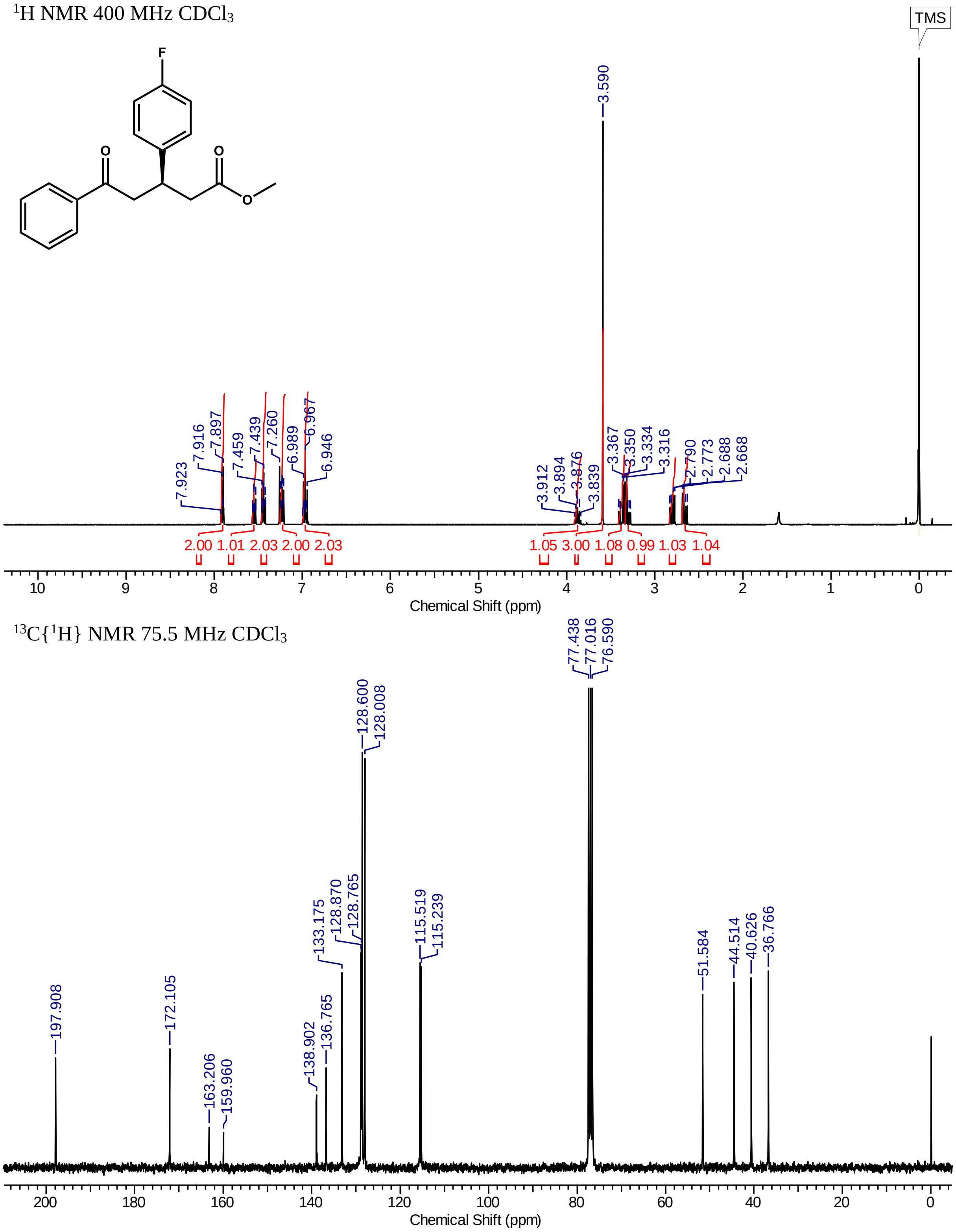

Figure S46. ^{1}H and ^{13}C NMR spectra of compound **4y**.

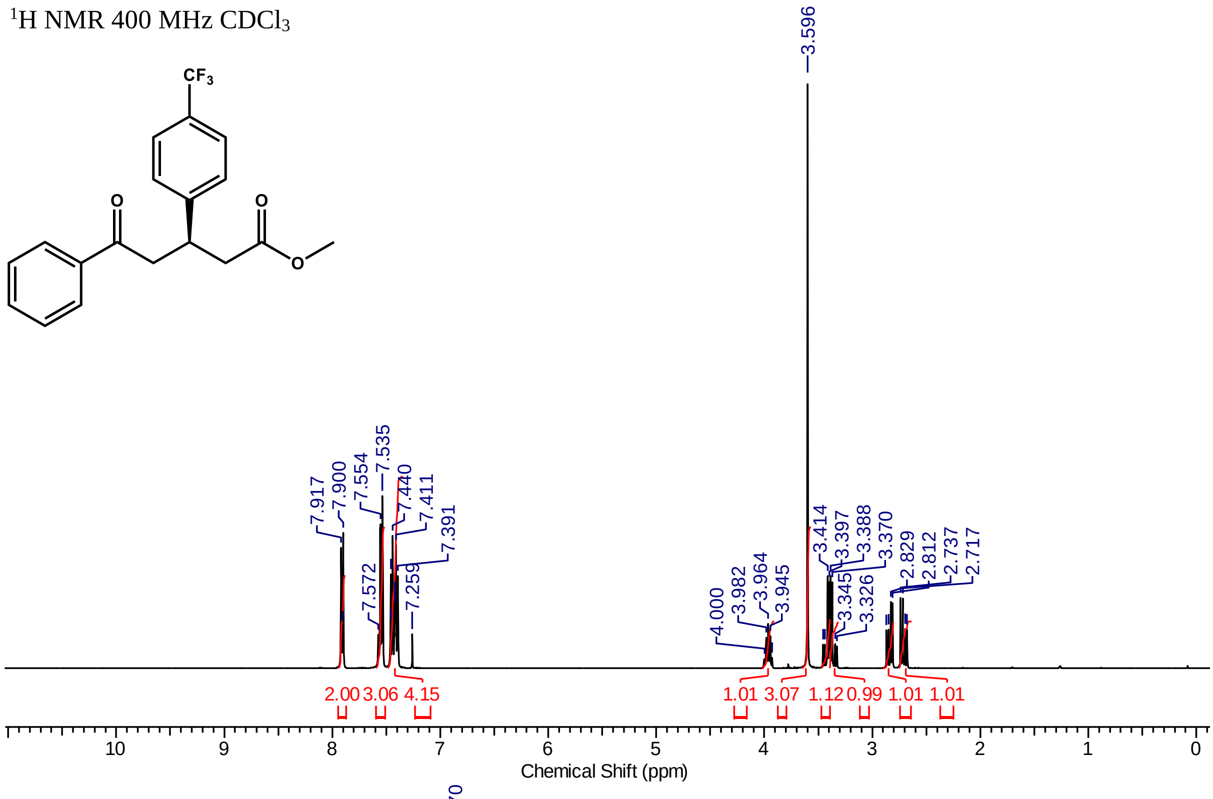

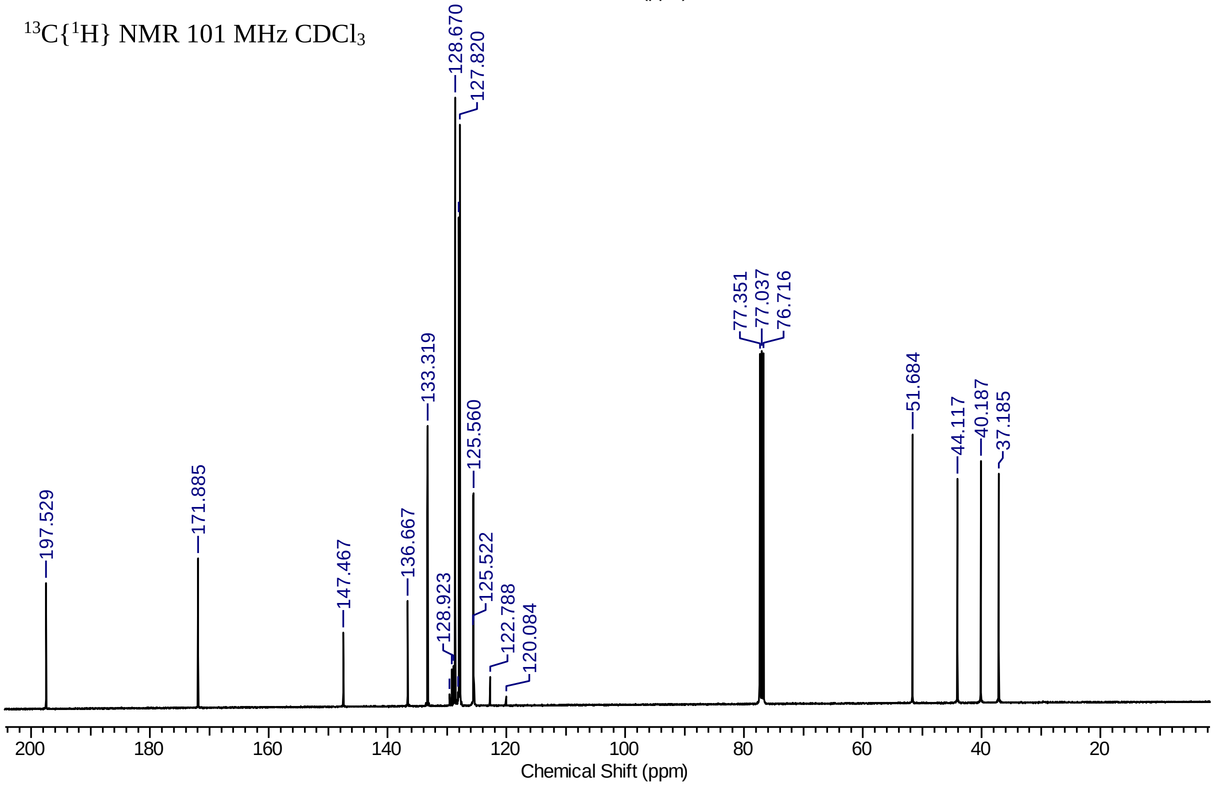

Figure S47. ^{1}H and ^{13}C NMR spectra of compound **4z**.

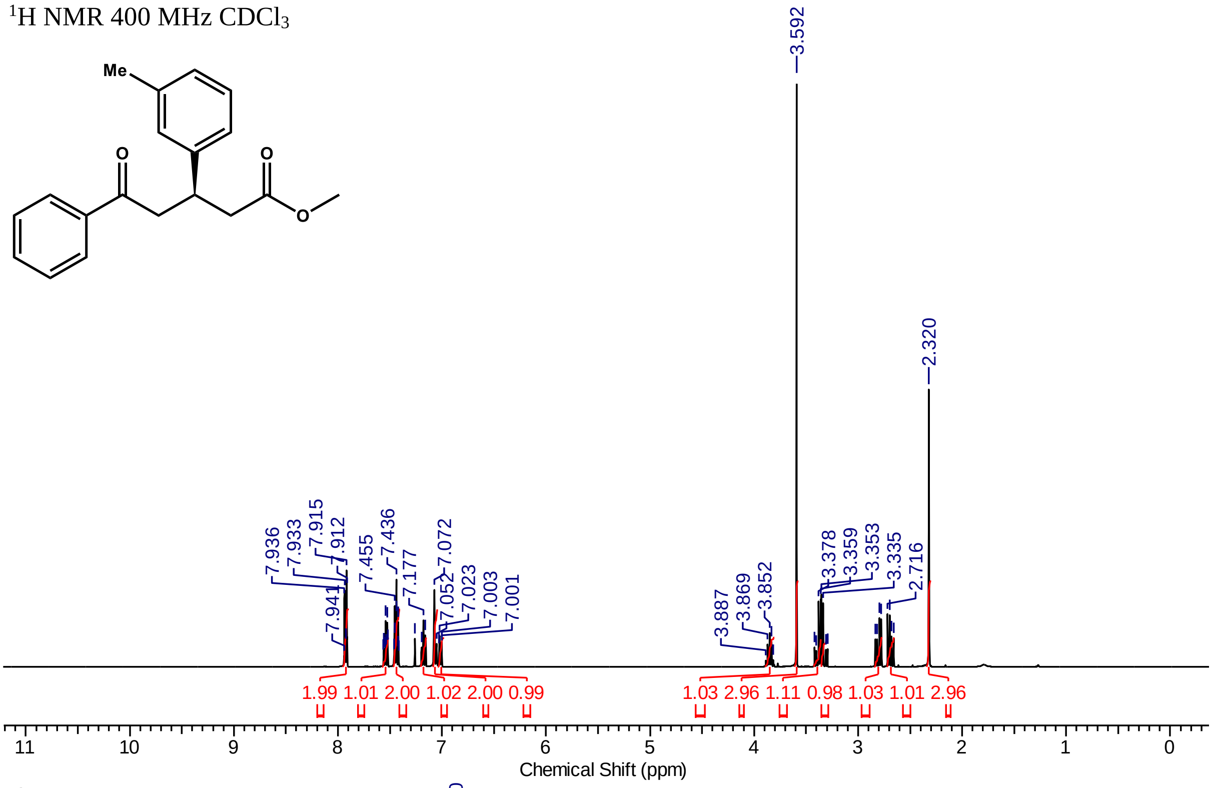

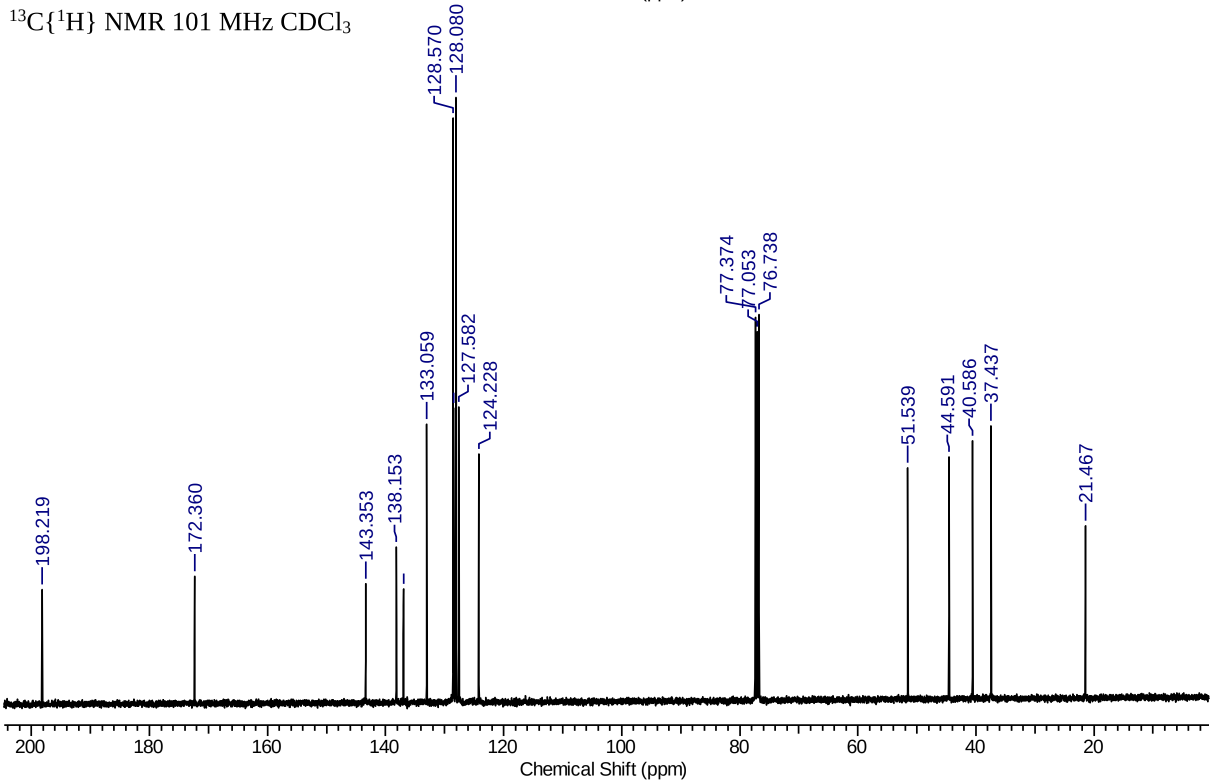

Figure S48. ^{1}H and ^{13}C NMR spectra of compound **4aa**.

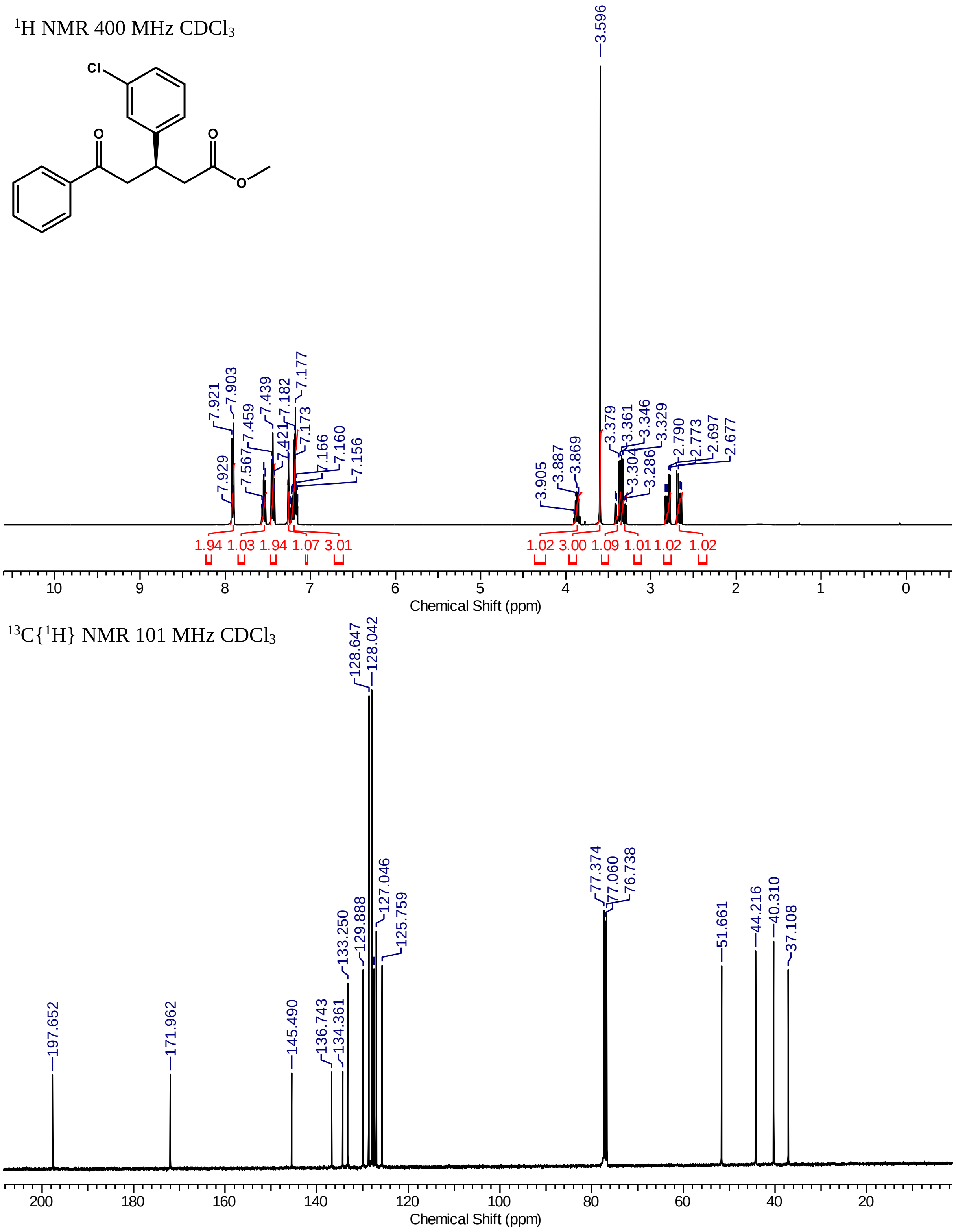

Figure S49. ^{1}H and ^{13}C NMR spectra of compound **4ab**.

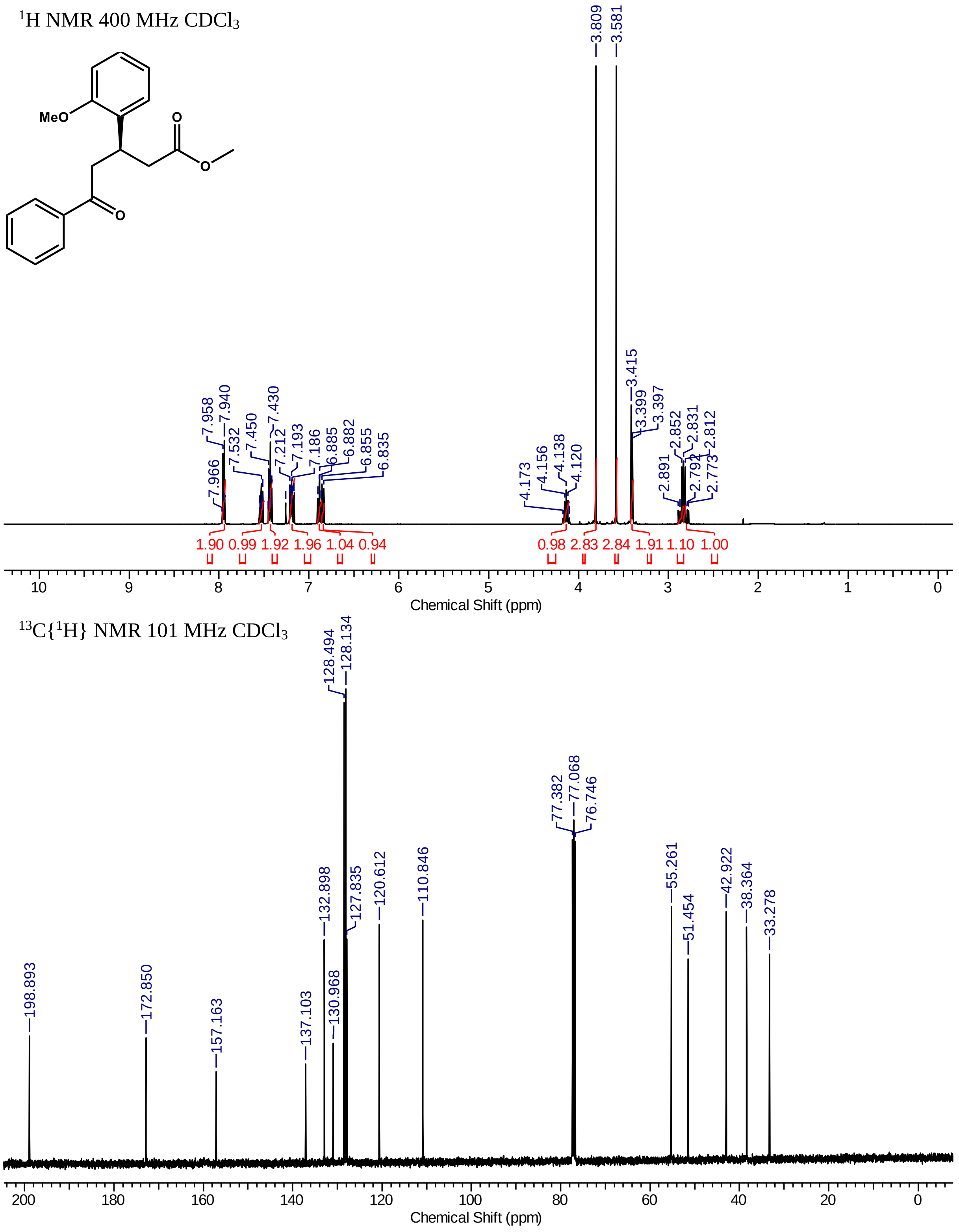

Figure S50. ^{1}H and ^{13}C NMR spectra of compound **4ac**.

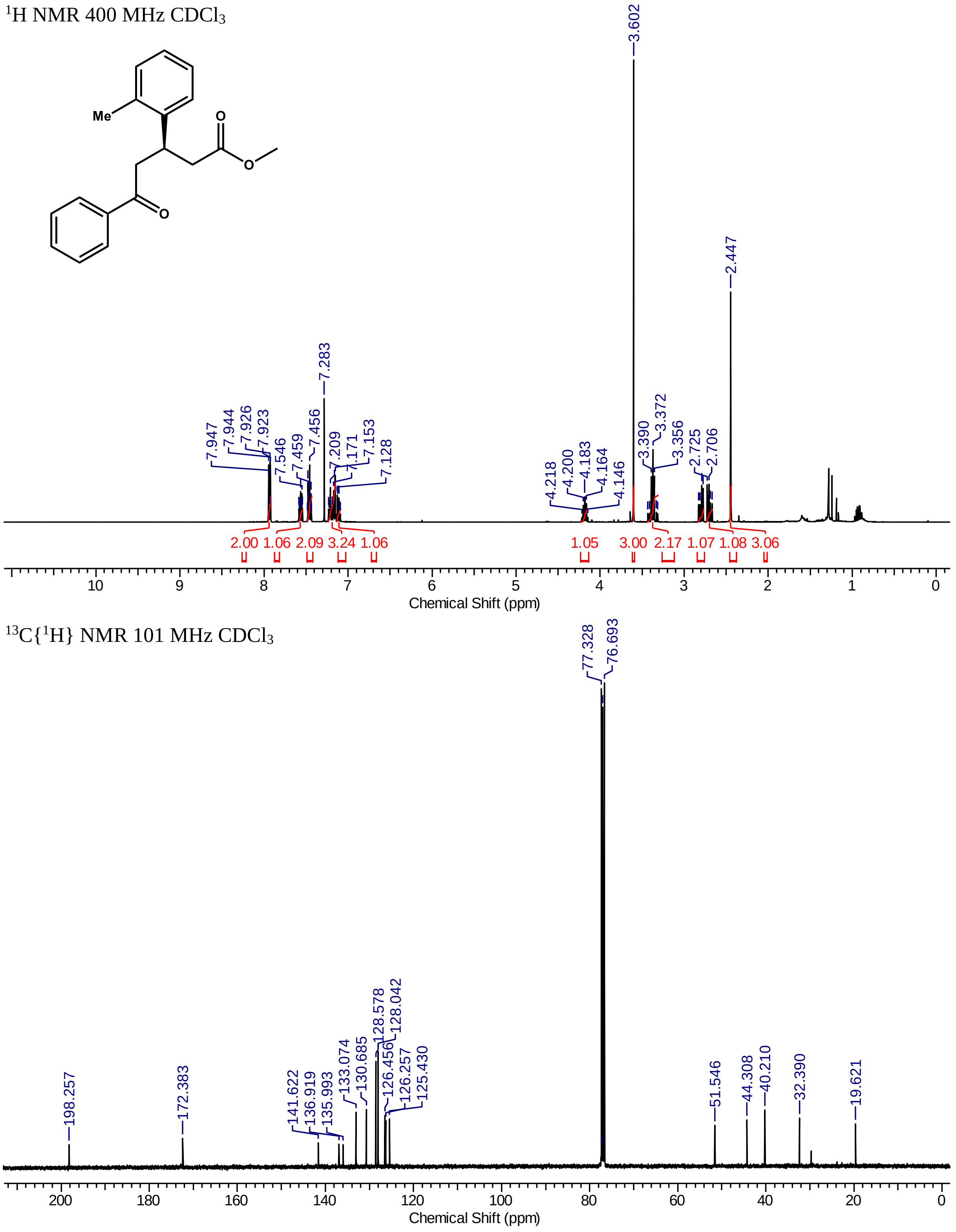

Figure S51. ^{1}H and ^{13}C NMR spectra of compound **4ad**.

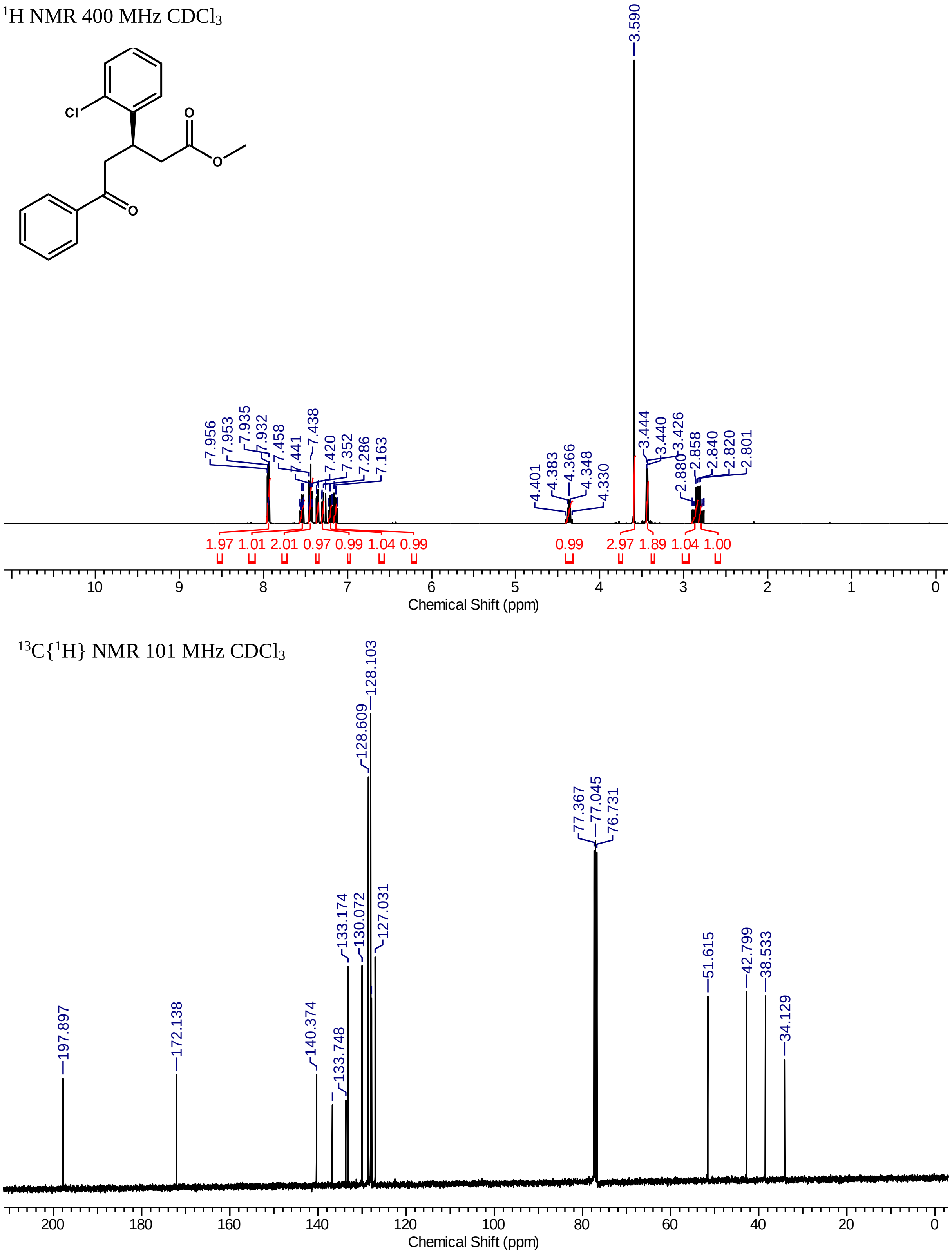

Figure S52. ^{1}H and ^{13}C NMR spectra of compound **4ae**.

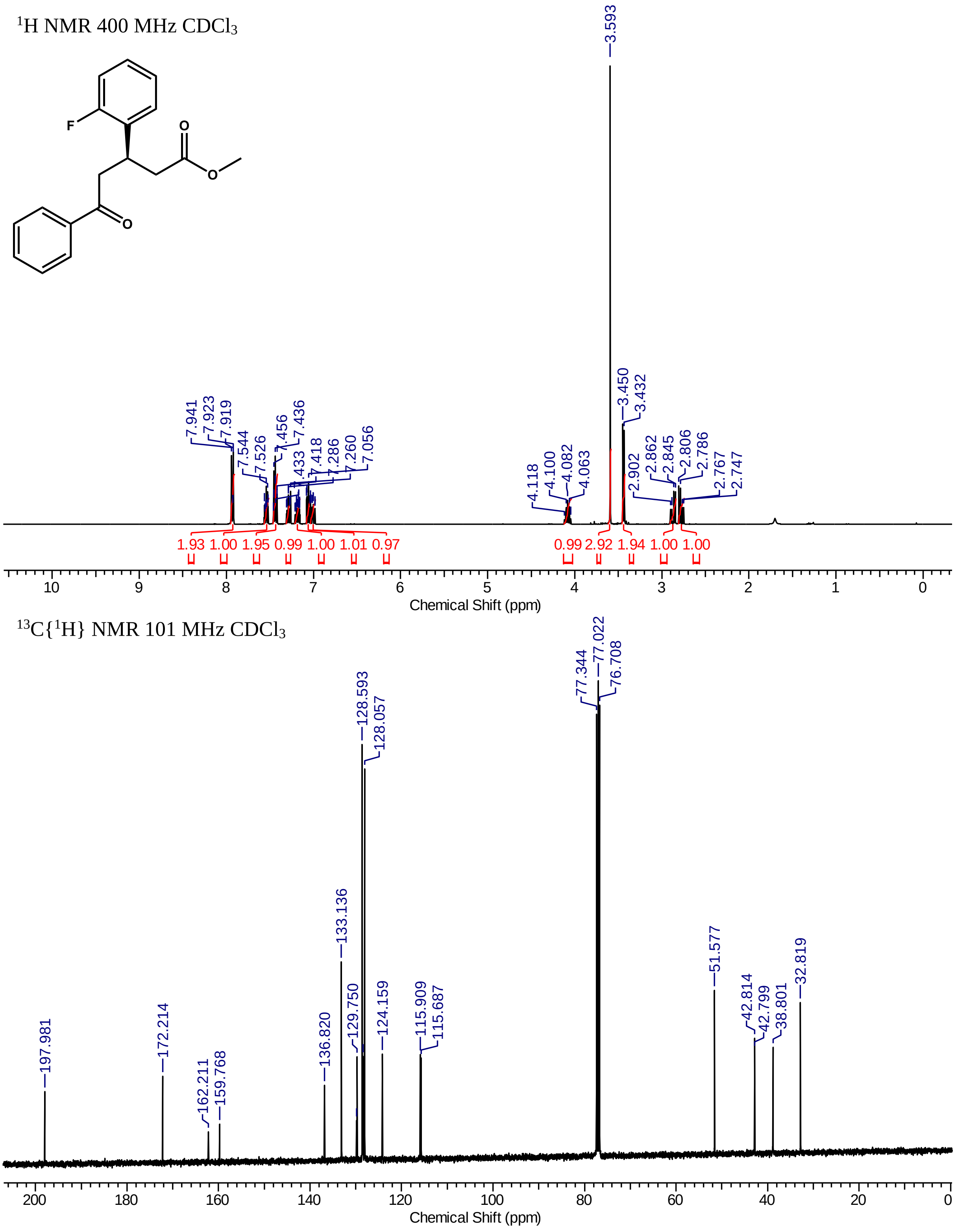

Figure S53. ^{1}H and ^{13}C NMR spectra of compound **4af**.

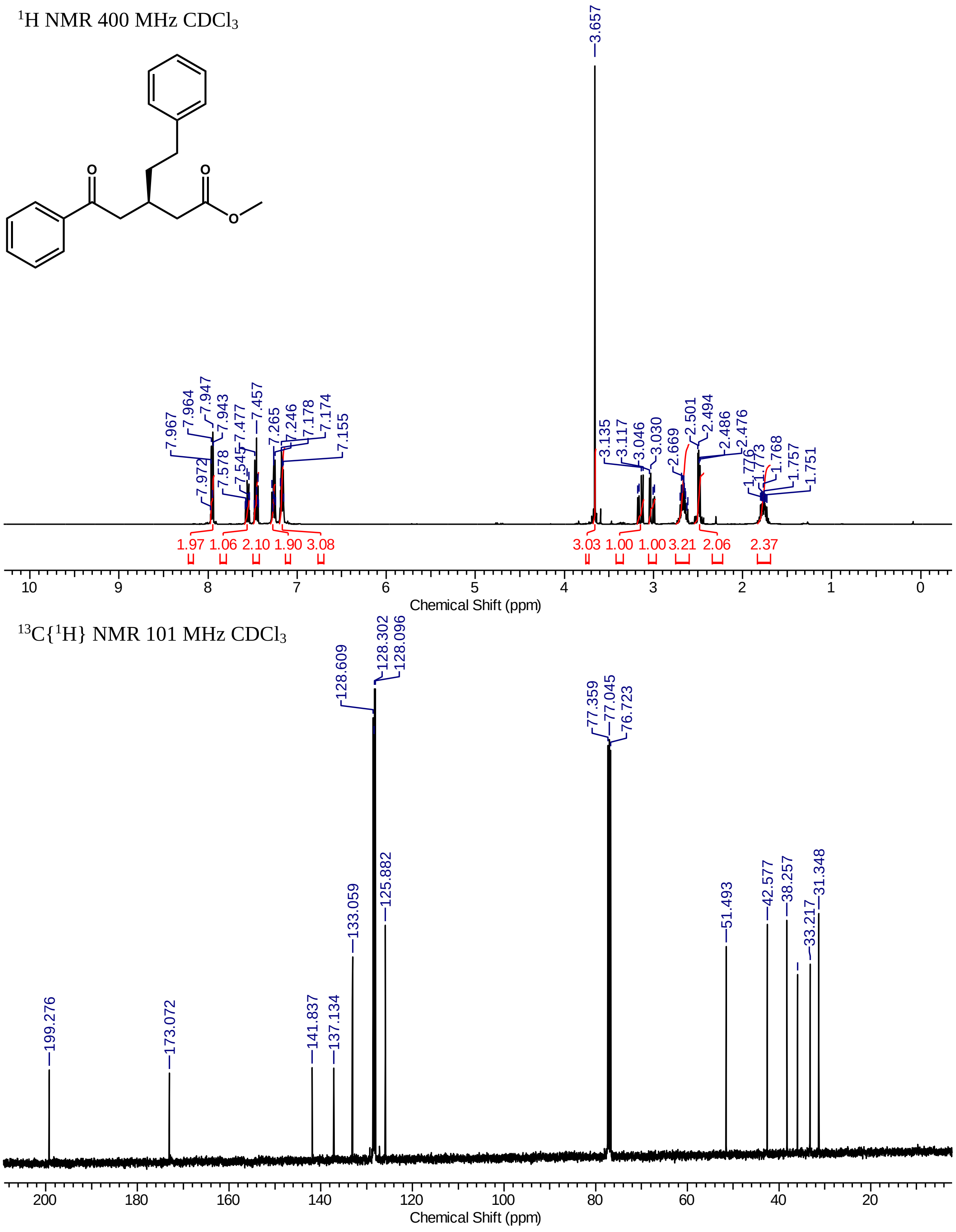

Figure S54. ^{1}H and ^{13}C NMR spectra of compound **4ag**.

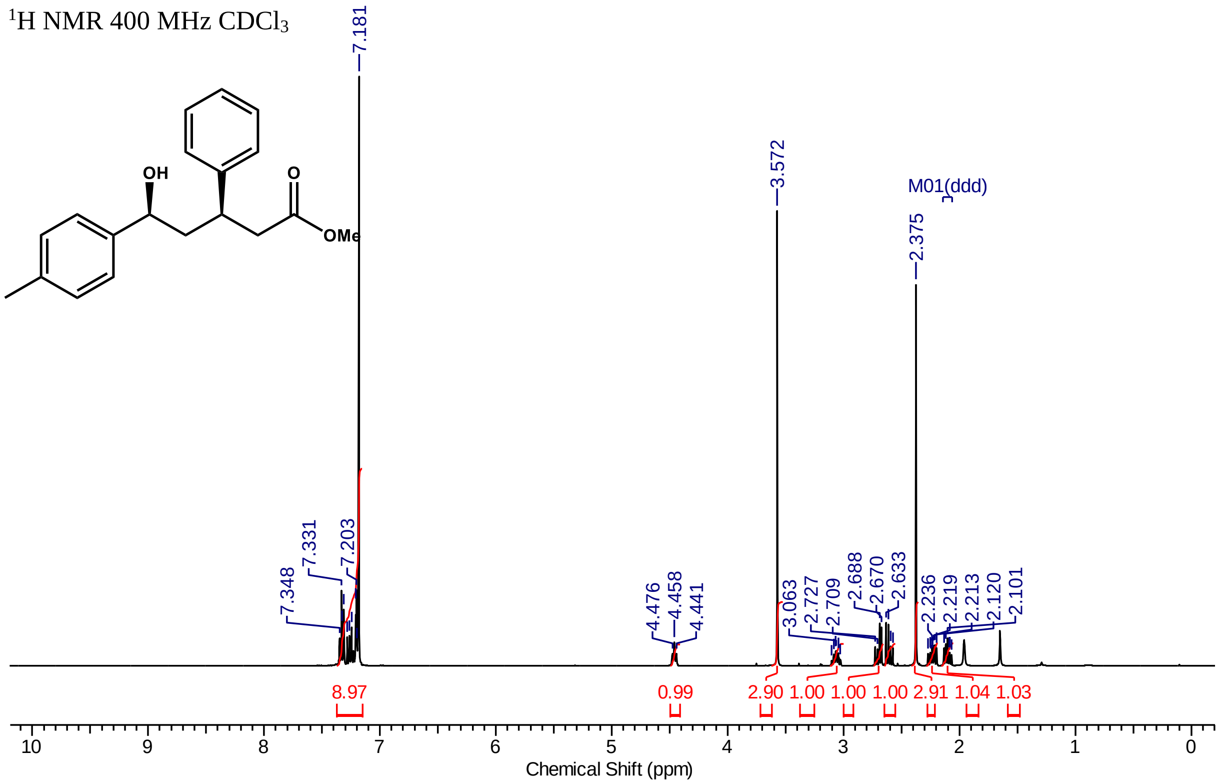

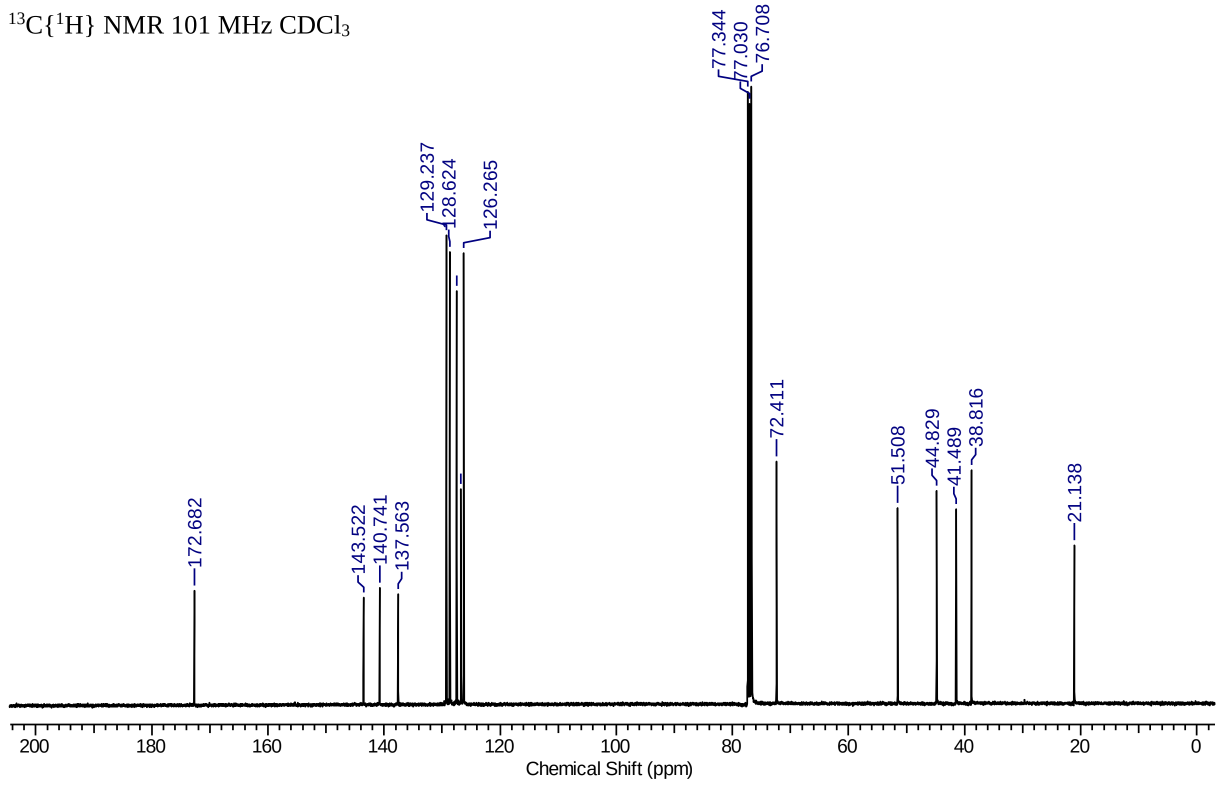

Figure S55. ^{1}H and ^{13}C NMR spectra of compound **5a**.

NOESY NMR 400 MHz $CDCl_3$

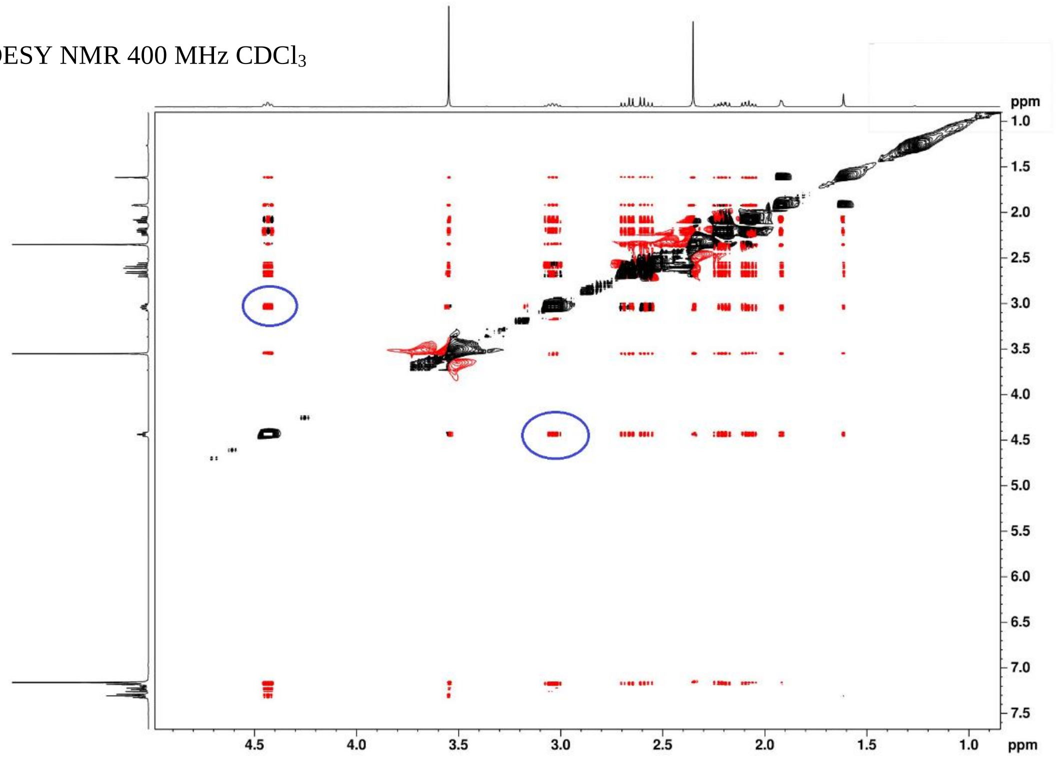

Figure S56. NOESY NMR spectrum of compound **5a**.

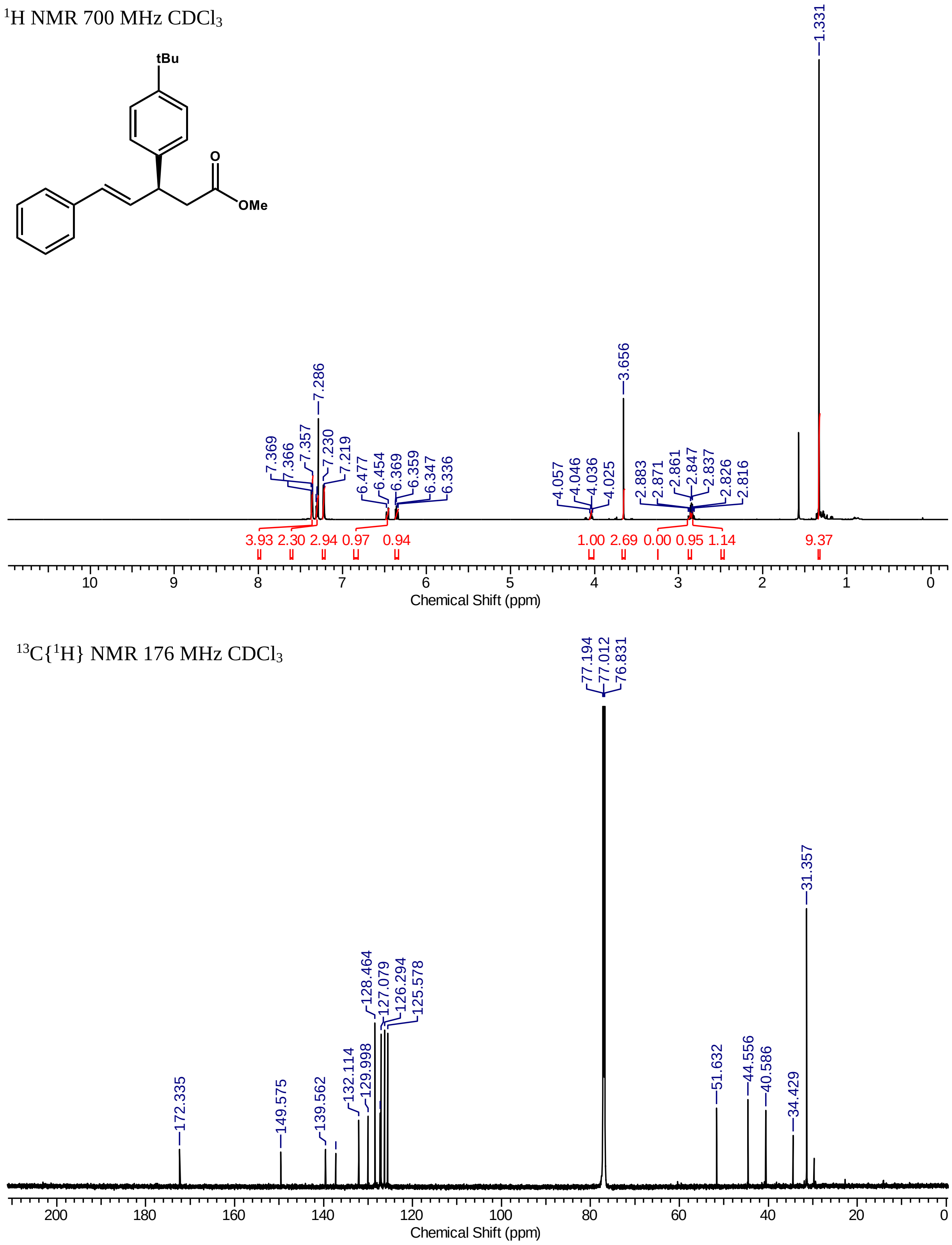

Figure S57. ^{1}H and ^{13}C NMR spectra of compound **5b**.

6. HPLC Chromatograms

Data File: C:\CHEM32\1\DATA\KD\CHIR000363.D
Sample Name: IB-LAK-RAC
Sample Info: Phenomenex Lux Amylose-1, 3 um, 80:20, 1.0 mL/min

->

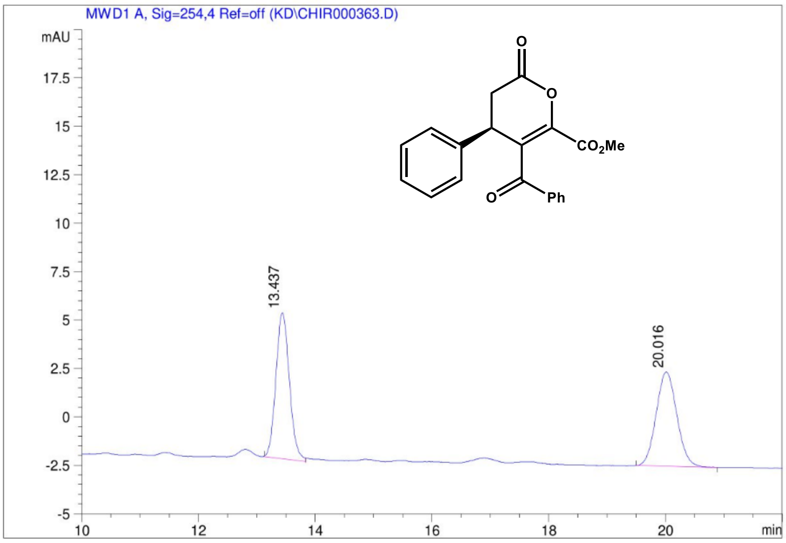

#	Meas. R	Area	Area %
1	13.437	121.448	50.311
2	20.016	119.948	49.689

Data File: C:\CHEM32\1\DATA\KD\CHIR000364.D
Sample Name: IB-LAK-chir
Sample Info: Phenomenex Lux Amylose-1, 3 um, 80:20, 1.0 mL/min

->

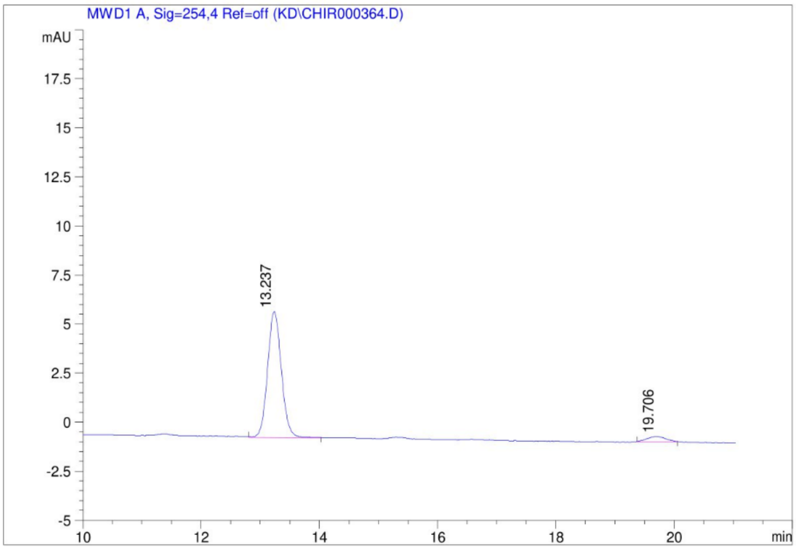

#	Meas. R	Area	Area %
1	13.237	104.111	94.516
2	19.706	6.040	5.484

Figure S58. HPLC chromatograms of **3** (racemic – top, enantioenriched – bottom)

```
Data File:  C:\CHEM32\1\DATA\KD\CHIR000707.D
Sample Name:  IBD-147
Sample Info:  Phenomenex Lux Amylose-1, 3 um, 80:20, 1,0 mL/min, race
              mat
                                                                     ->
=====================================================================
```

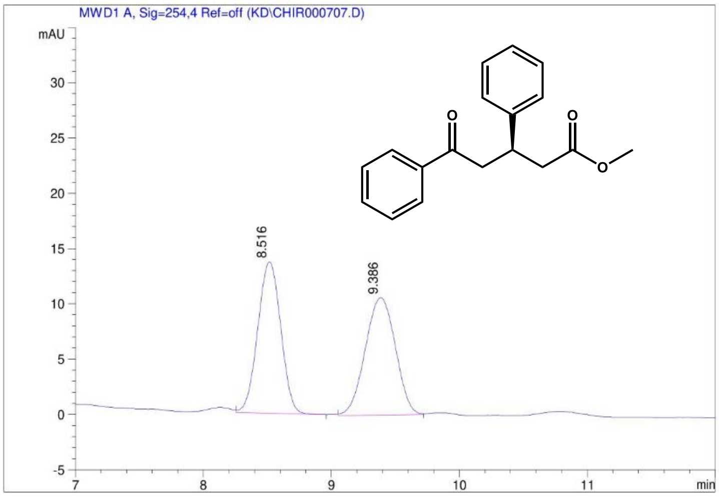

#	Meas. R	Area	Area %
1	8.516	171.497	49.657
2	9.386	173.868	50.343

```
Data File:  C:\CHEM32\1\DATA\KD\CHIR000708.D
Sample Name:  IBD-148
Sample Info:  Phenomenex Lux Amylose-1, 3 um, 80:20, 1,0 mL/min, chir
              alny
                                                                     ->
=====================================================================
```

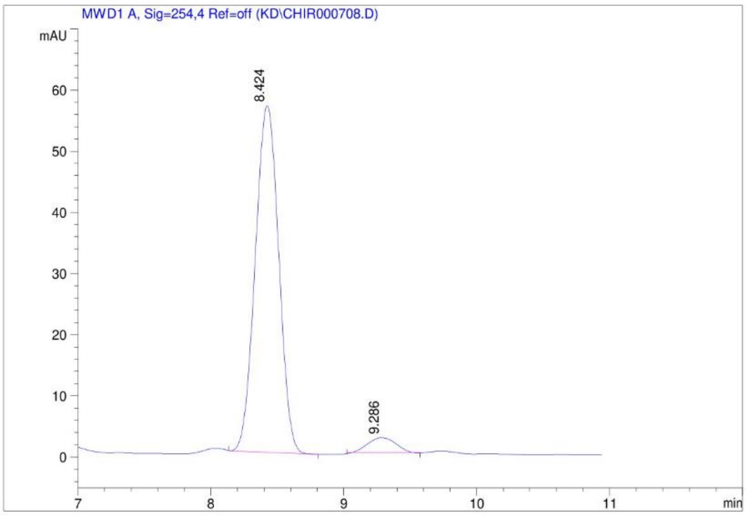

#	Meas. R	Area	Area %
1	8.424	694.465	95.136
2	9.286	35.507	4.864

Figure S59. HPLC chromatograms of **4a** (racemic – top, enantioenriched – bottom).

Data File: C:\CHEM32\1\DATA\IB\CHIR000148.D
Sample Name: IBD-192-RAC
Sample Info: Phenomenex Lux Amylose-1, 3 um, 80:20, 1.0 mL/min

->

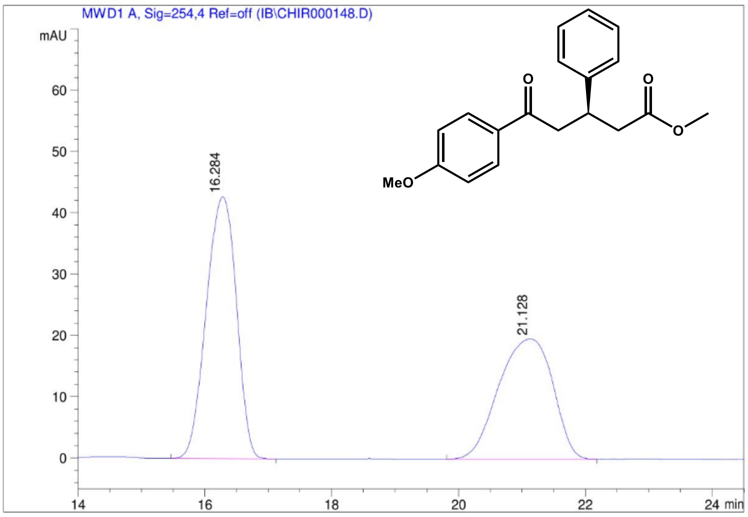

#	Meas. R	Area	Area %
1	16.284	1.411e3	54.817
2	21.128	1.163e3	45.183

Data File: C:\CHEM32\1\DATA\IB\CHIR000149.D
Sample Name: IBD-192-CH
Sample Info: Phenomenex Lux Amylose-1, 3 um, 80:20, 1.0 mL/min

->

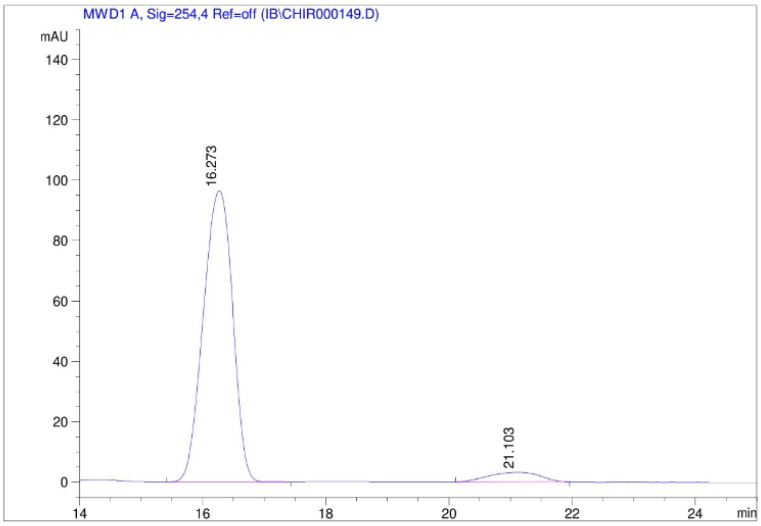

#	Meas. R	Area	Area %
1	16.273	3.196e3	94.587
2	21.103	182.877	5.413

Figure S60. HPLC chromatograms of **4b** (racemic – top, enantioenriched – bottom).

kalibracja wzorcem zewnętrznym

Nazwa analizy: C:\CHROMA\BSZ\IBD-198-rac.chr
Metoda: C:\CHROMA\ANALIZY\gc1.met
Data utworzenia: 11-03-21 12:20 /
Analiza: 3663 ChromaX wersja1.0b

Phenomenex Lux Amylose-1 3 um hexane/IPA 80:20 1.0 mL/min

NUMER PIKU	CZAS RETENCJI [min]	POWIERZCHNIA [µV 1/100 min]	UDZIAŁ % POWIERZCHNI
A/1	17,145	95243	50,698
A/2	23,583	92619	49,302
SUMA		187862	

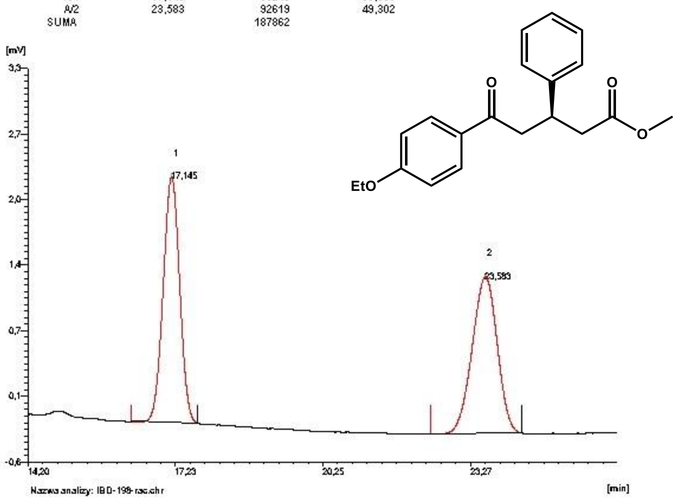

kalibracja wzorcem zewnętrznym

Nazwa analizy: C:\CHROMA\BSZ\ibd-198-.chr
Metoda: C:\CHROMA\ANALIZY\gc1.met
Data utworzenia: 11-03-21 13:09 /
Analiza: 3664 Chr-mod wersja1.0

Phenomenex Lux Amylose-1 3 um hexane/IPA 80:20 1.0 mL/min

NUMER PIKU	CZAS RETENCJI [min]	POWIERZCHNIA [µV 1/100 min]	UDZIAŁ % POWIERZCHNI
A/1	17,102	3705843	95,380
A/2	23,575	179497	4,620
SUMA		3885340	

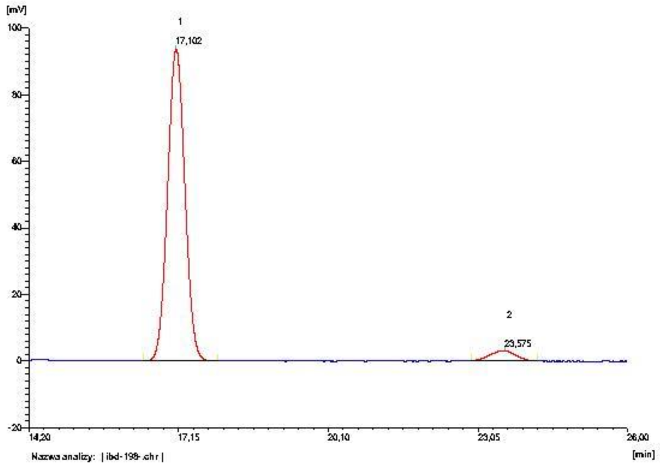

Figure S61. HPLC chromatograms of **4c** (racemic – top, enantioenriched – bottom).

```
Data File:  C:\CHEM32\1\DATA\MS\RAC000168.D
Sample Name:  IBD 196 RAC SPR
Sample Info:  Phenomenex Lux Amylose-1, 3 um, 70:30, 1.0 mL/min
```

->

===

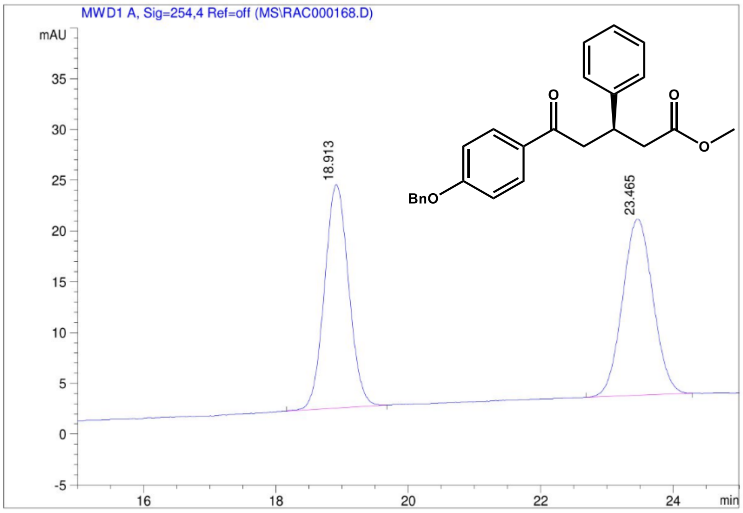

#	Meas. R	Area	Area %
1	18.913	559.037	50.176
2	23.465	555.109	49.824

```
Data File:  C:\CHEM32\1\DATA\IB\CHIR000175.D
Sample Name:  IBD-196-CH
Sample Info:  Phenomenex Lux Amylose-1, 3 um, 70:30, 1.0 mL/min
```

->

===

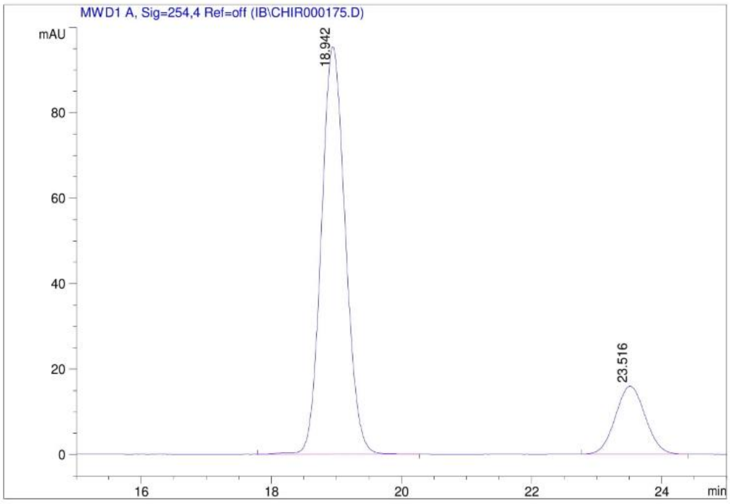

#	Meas. R	Area	Area %
1	18.942	2.443e3	82.813
2	23.516	506.990	17.187

Figure S62. HPLC chromatograms of **4d** (racemic – top, enantioenriched – bottom).

```
Data File:  C:\CHEM32\1\DATA\IB\RAC000020.D
Sample Name:  IBD-190-rac
Sample Info:  Phenomenex Lux Amylose-1, 3 um, 90:10, 1.00 mL/min, p=9
              3bar; T=25st.C
```

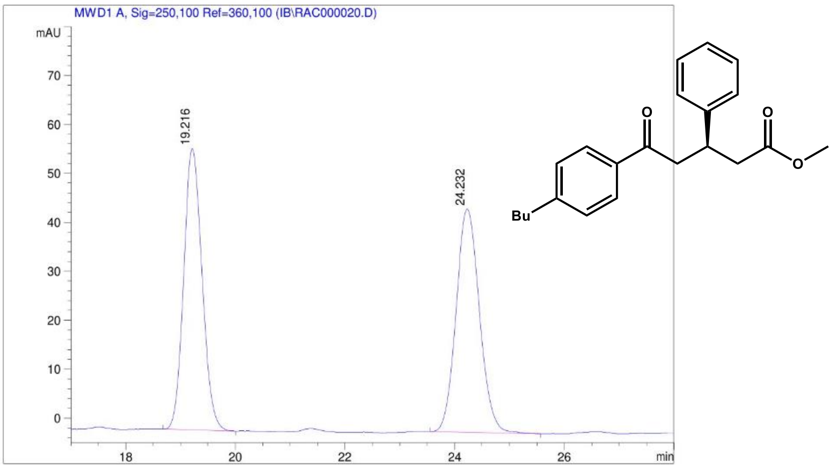

#	Meas. R	Area	Area %
1	19.216	1.337e3	49.767
2	24.232	1.350e3	50.233

```
Data File:  C:\CHEM32\1\DATA\IB\CHIR000021.D
Sample Name:  IBD-190-chir
Sample Info:  Phenomenex Lux Amylose-1, 3 um, 90:10, 1.00 mL/min, p=9
              3bar; T=25st.C
```

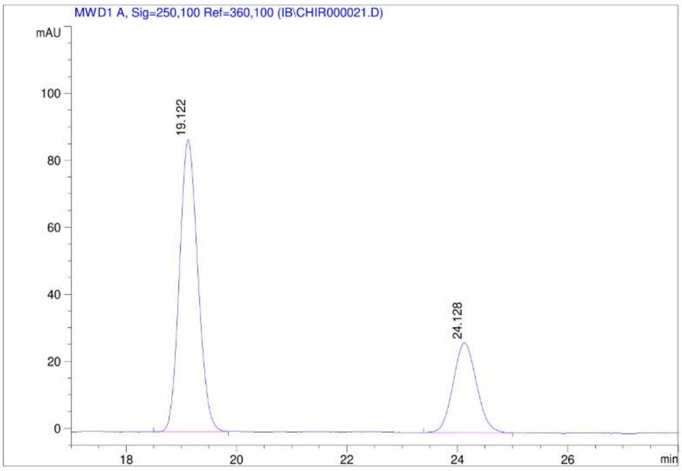

#	Meas. R	Area	Area %
1	19.122	2.020e3	71.878
2	24.128	790.233	28.122

Figure S63. HPLC chromatograms of **4e** (racemic – top, enantioenriched – bottom).

```
Data File:  C:\CHEM32\1\DATA\IB\RAC000119.D
Sample Name:  IBD-185-RAC
Sample Info:  Phenomenex Lux Cellulose-1, 3 um, 90:10, 1.0 mL/min .
```

->

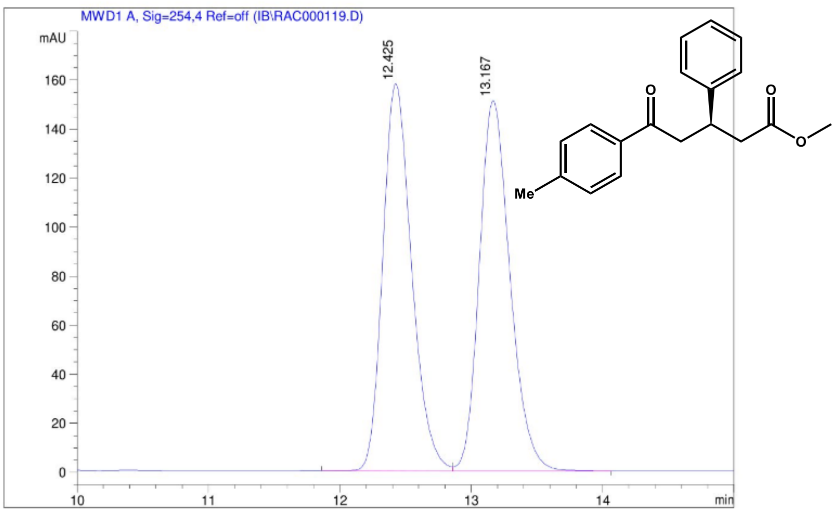

#	Meas. R	Area	Area %
1	12.425	2.411e3	49.571
2	13.167	2.452e3	50.429

```
Data File:  C:\CHEM32\1\DATA\IB\CHIR000120.D
Sample Name:  IBD-185-CHIR
Sample Info:  Phenomenex Lux Cellulose-1, 3 um, 90:10, 1.0 mL/min .
```

->

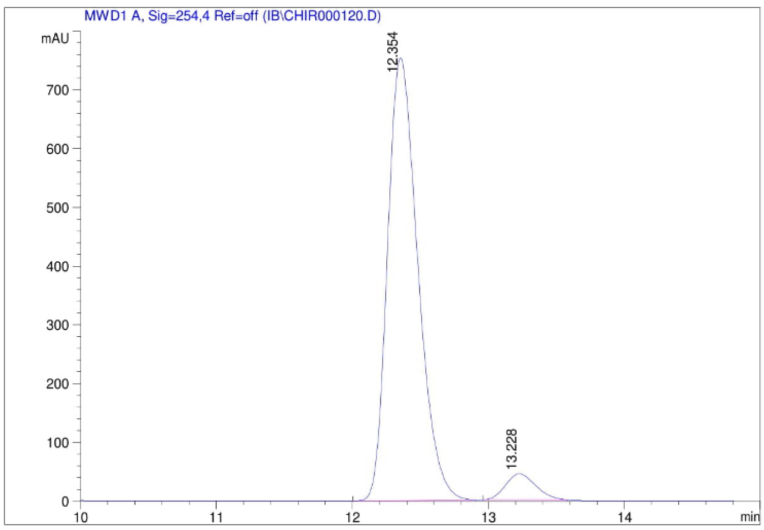

#	Meas. R	Area	Area %
1	12.354	1.170e4	94.457
2	13.228	686.444	5.543

Figure S64. HPLC chromatograms of **4f** (racemic – top, enantioenriched – bottom).

Data File: C:\CHEM32\1\DATA\IB\RAC000109.D
Sample Name: IBD164-rac
Sample Info: Phenomenex Lux Amylose-1, 3 um, 80:20, 1,0 mL/min, achi
ralny

->

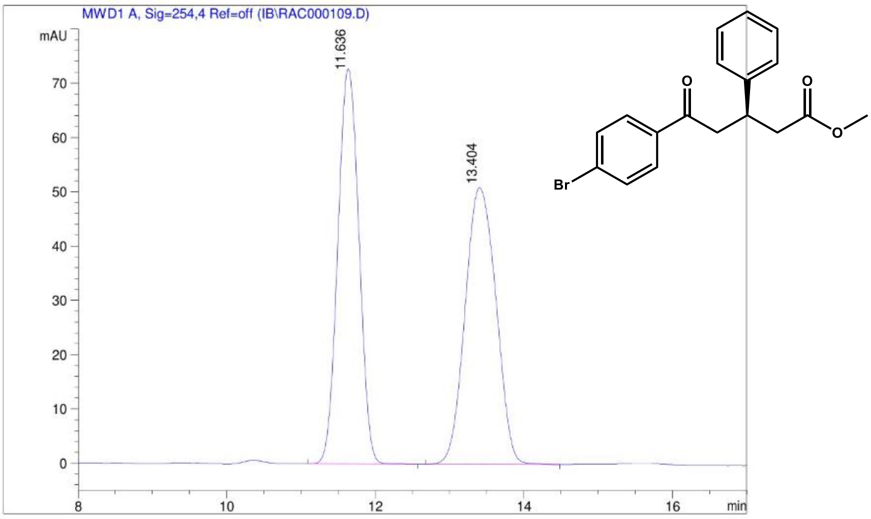

#	Meas. R	Area	Area %
1	11.636	1.444e3	49.449
2	13.404	1.476e3	50.551

Data File: C:\CHEM32\1\DATA\IB\CHIR000113.D
Sample Name: IBD163-CHIR
Sample Info: Phenomenex Lux Amylose-1, 3 um, 80:20, 1,0 mL/min, chir
alny

->

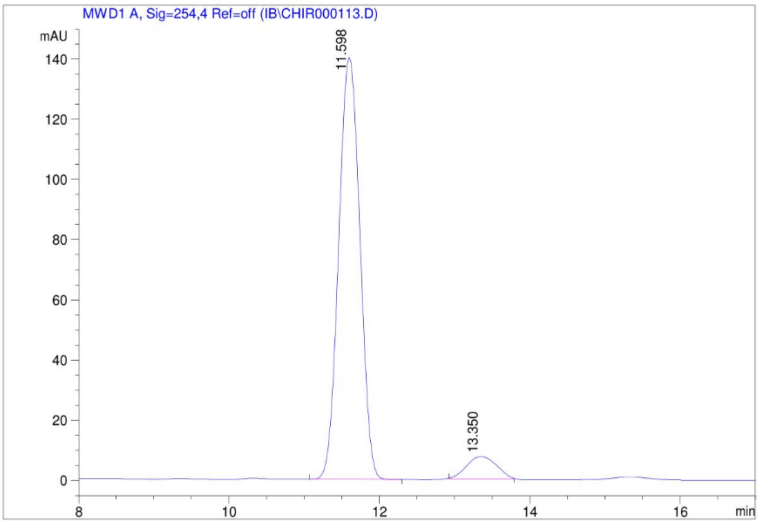

#	Meas. R	Area	Area %
1	11.598	2.754e3	92.876
2	13.350	211.250	7.124

Figure S65. HPLC chromatograms of **4g** (racemic – top, enantioenriched – bottom).

```
Data File:  C:\CHEM32\1\DATA\IB\ACHIR000096.D
Sample Name:  IBD154-rac
Sample Info:  Phenomenex Lux Amylose-1, 3 um, 80:20, 1,0 mL/min, achi
              ralny
                                                                        ->
=========================================================================
```

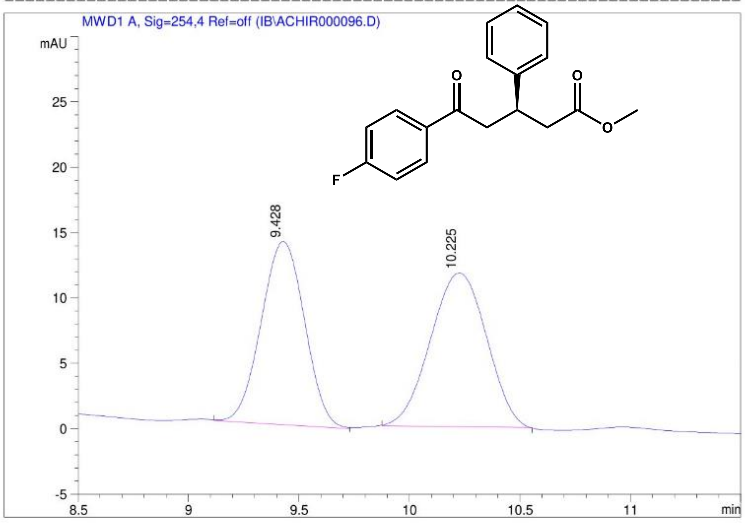

#	Meas. R	Area	Area %
1	9.428	191.400	47.972
2	10.225	207.582	52.028

```
Data File:  C:\CHEM32\1\DATA\IB\RAC000108.D
Sample Name:  IBD161
Sample Info:  Phenomenex Lux Amylose-1, 3 um, 80:20, 1,0 mL/min, achi
              ralny
                                                                        ->
=========================================================================
```

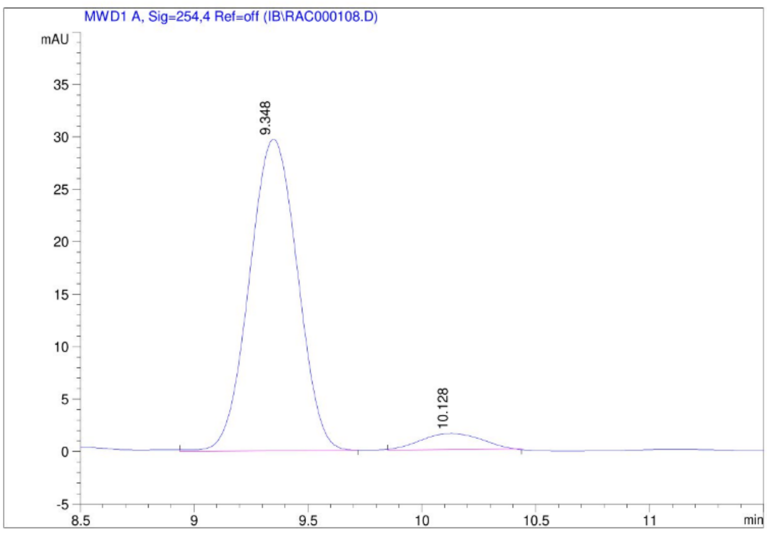

#	Meas. R	Area	Area %
1	9.348	435.587	93.871
2	10.128	28.439	6.129

Figure S66. HPLC chromatograms of **4h** (racemic – top, enantioenriched – bottom).

```
Data File:  C:\CHEM32\1\DATA\IB\RAC000111.D
Sample Name:  IBD-180-RAC
Sample Info:  Phenomenex Lux Amylose-1, 3 um, 80:20, 1.0 mL/min .
```

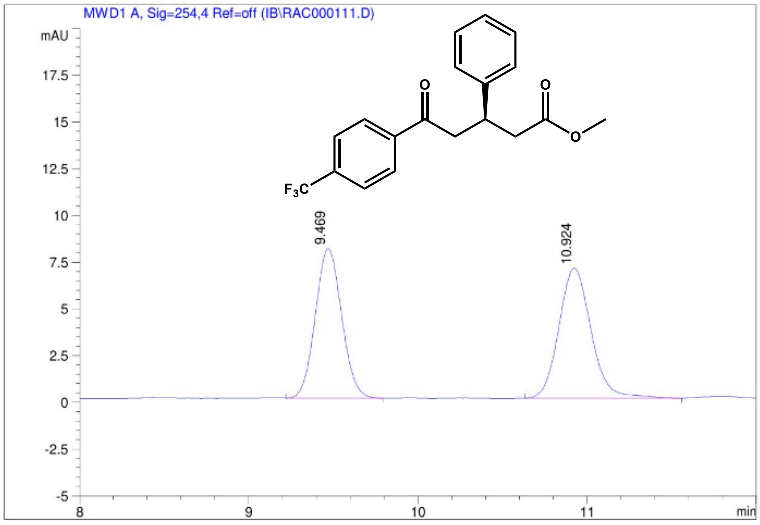

#	Meas. R	Area	Area %
1	9.469	86.562	48.438
2	10.924	92.145	51.562

```
Data File:  C:\CHEM32\1\DATA\IB\CHIR000112.D
Sample Name:  IBD-180-CHIR
Sample Info:  Phenomenex Lux Amylose-1, 3 um, 80:20, 1.0 mL/min .
```

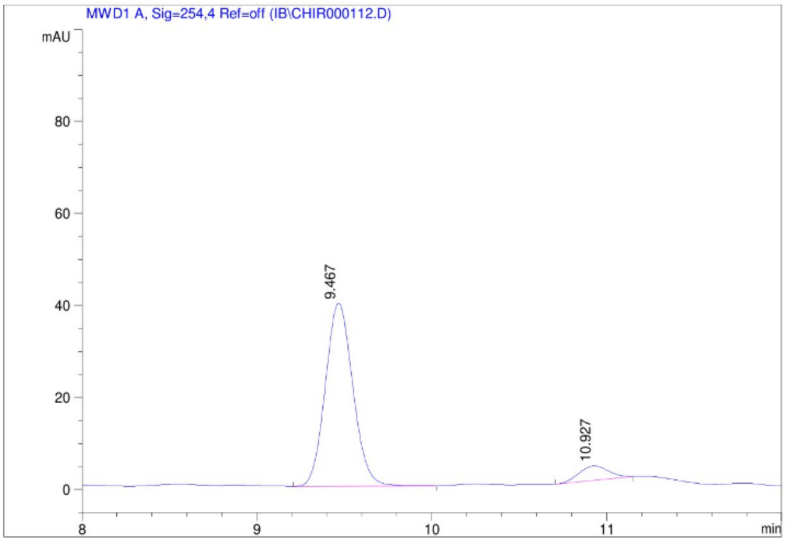

#	Meas. R	Area	Area %
1	9.467	434.793	92.199
2	10.927	36.788	7.801

Figure S67. HPLC chromatograms of **4i** (racemic – top, enantioenriched – bottom).

Phenomenex Amylose Lux 1, hexane 90:10 IPA, 1 mL/min, 254 nm

NUMER PIKU	CZAS RETENCJI [min]	POWIERZCHNIA [µV 1/100 min]	UDZIAŁ % POWIERZCHNI
A/1	11,960	428550	50,608
A/2	13,633	418249	49,392
SUMA		846799	

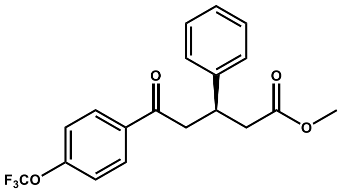

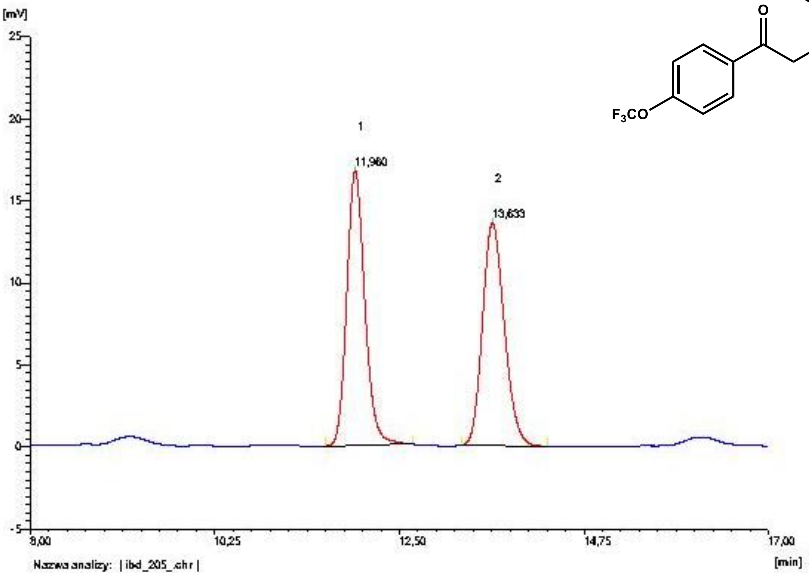

Phenomenex Amylose Lux 1, hexane 90:10 IPA, 1 mL/min, 254 nm

NUMER PIKU	CZAS RETENCJI [min]	POWIERZCHNIA [µV 1/100 min]	UDZIAŁ % POWIERZCHNI
A/1	11,958	2110817	82,037
A/2	13,647	462176	17,963
SUMA		2572993	

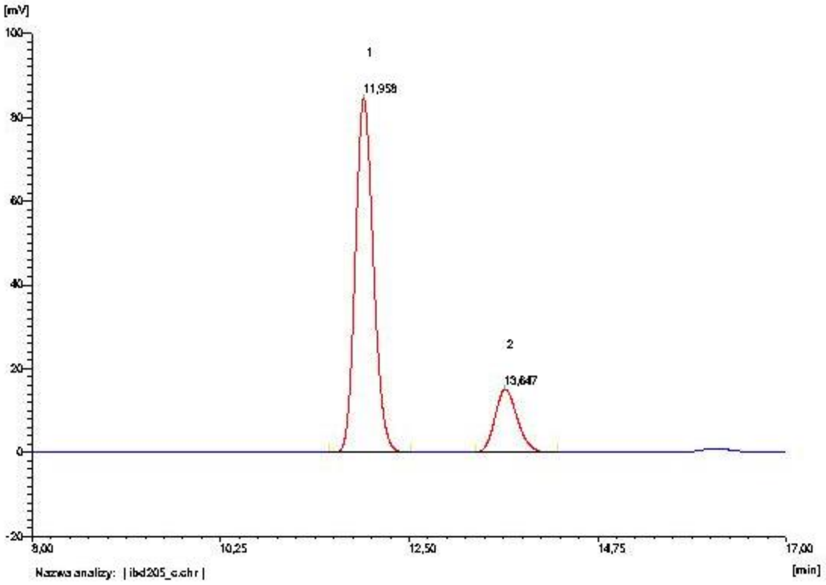

Figure S68. HPLC chromatograms of **4j** (racemic – top, enantioenriched – bottom).

```
Data File:   C:\CHEM32\1\DATA\IB\CHIR000029.D
Sample Name:   IBD-170-RAC
Sample Info:   Phenomenex Lux Cellulose-1, 3 um, 90:10, 1,0 mL/min .
```

->

==

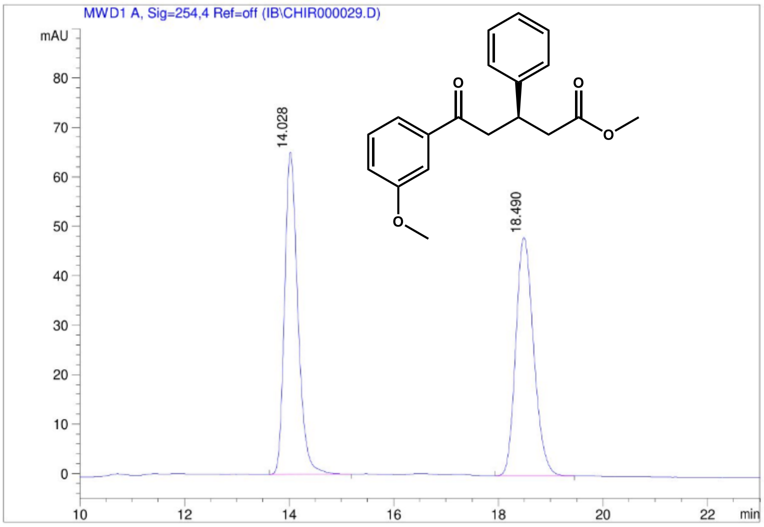

#	Meas. R	Area	Area %
1	14.028	1.150e3	50.032
2	18.490	1.149e3	49.968

```
Data File:   C:\CHEM32\1\DATA\IB\CHIR000030.D
Sample Name:   IBD-170-CH
Sample Info:   Phenomenex Lux Cellulose-1, 3 um, 90:10, 1,0 mL/min .
```

->

==

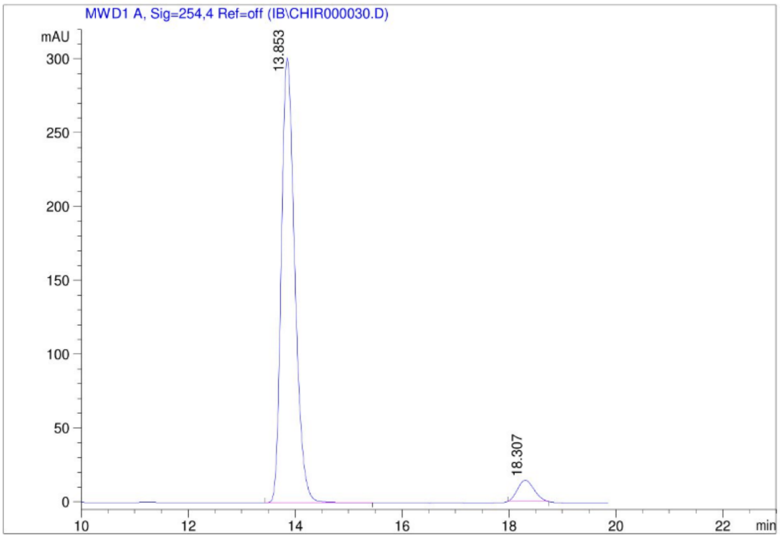

#	Meas. R	Area	Area %
1	13.853	5.260e3	94.859
2	18.307	285.075	5.141

Figure S69. HPLC chromatograms of **4k** (racemic – top, enantioenriched – bottom).

Data File: C:\CHEM32\1\DATA\IB\CHIR000048.D
Sample Name: IB-177-RAC
Sample Info: Phenomenex Lux Cellulose-1, 3 um, 80:20, 1,0 mL/min .

->

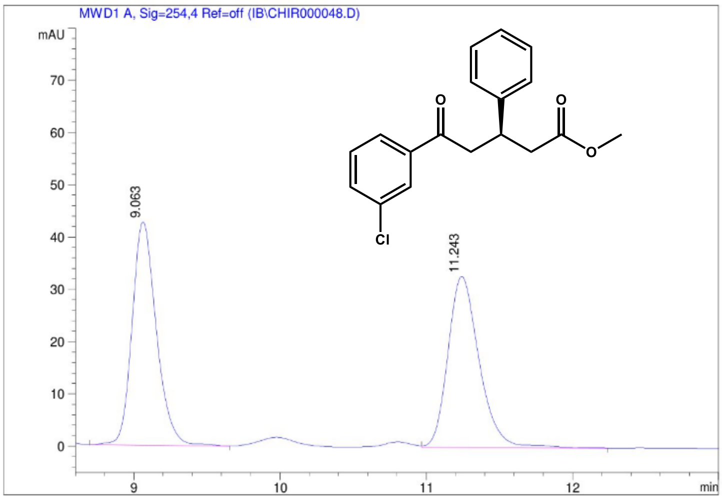

#	Meas. R	Area	Area %
1	9.063	494.092	50.357
2	11.243	487.082	49.643

Data File: C:\CHEM32\1\DATA\IB\CHIR000049.D
Sample Name: IB-177-CH
Sample Info: Phenomenex Lux Cellulose-1, 3 um, 80:20, 1,0 mL/min .

->

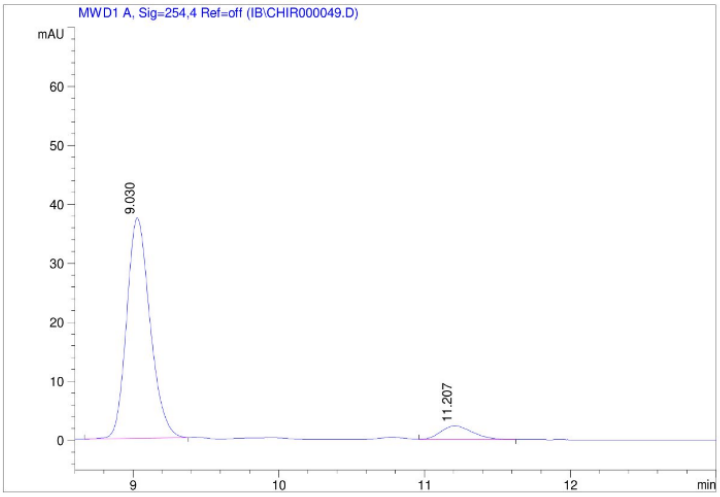

#	Meas. R	Area	Area %
1	9.030	416.894	92.139
2	11.207	35.566	7.861

Figure S70. HPLC chromatograms of **4l** (racemic – top, enantioenriched – bottom).

Data File: C:\CHEM32\1\DATA\IB\CHIR000042.D
Sample Name: IB-175-rac
Sample Info: Phenomenex Lux Cellulose-1, 3 um, 90:10, 1,0 mL/min .

->

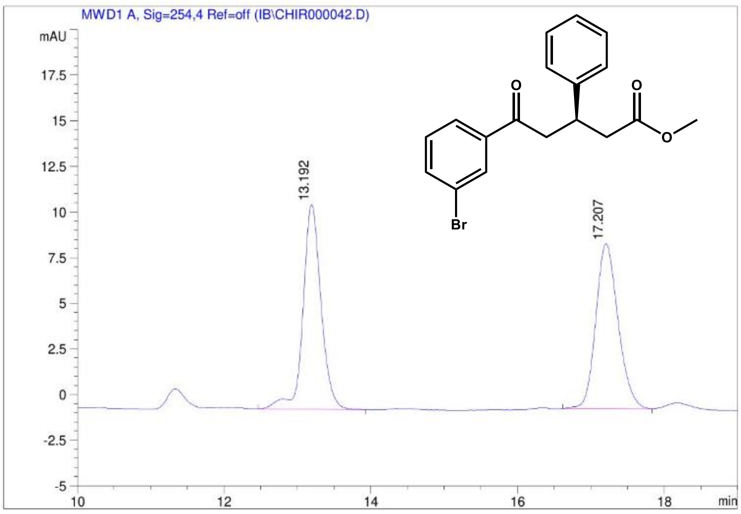

#	Meas. R	Area	Area %
1	13.192	192.726	50.179
2	17.207	191.352	49.821

Data File: C:\CHEM32\1\DATA\IB\CHIR000043.D
Sample Name: IB-175-chir
Sample Info: Phenomenex Lux Cellulose-1, 3 um, 90:10, 1,0 mL/min .

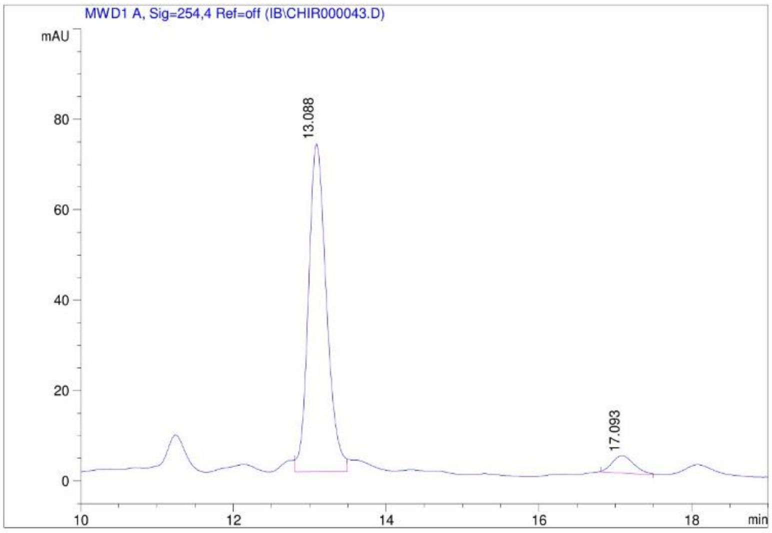

#	Meas. R	Area	Area %
1	13.088	1.187e3	93.940
2	17.093	76.563	6.060

Figure S71. HPLC chromatograms of **4m** (racemic – top, enantioenriched – bottom).

```
Data File:  C:\CHEM32\1\DATA\IB\CHIR000121.D
Sample Name:  IBD-184-RAC
Sample Info:  Phenomenex Lux Cellulose-1, 3 um, 90:10, 1.0 mL/min .
```

->

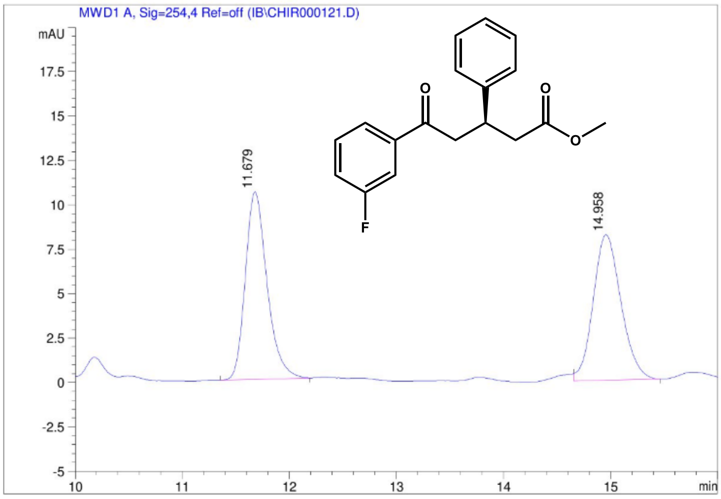

#	Meas. R	Area	Area %
1	11.679	148.669	50.126
2	14.958	147.919	49.874

```
Data File:  C:\CHEM32\1\DATA\IB\CHIR000122.D
Sample Name:  IBD-184-CHIR
Sample Info:  Phenomenex Lux Cellulose-1, 3 um, 90:10, 1.0 mL/min .
```

->

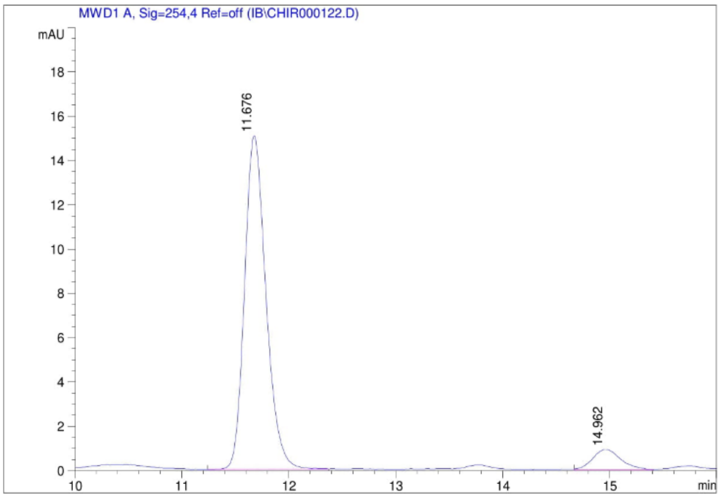

#	Meas. R	Area	Area %
1	11.676	209.464	92.411
2	14.962	17.201	7.589

Figure S72. HPLC chromatograms of **4n** (racemic – top, enantioenriched – bottom).

```
Data File:  C:\CHEM32\1\DATA\IB\RAC000196.D
Sample Name:  IBD-206-rac
Sample Info:  Phenomenex Lux Cellulose-1, 3 um, 90:10, 1.0 mL/min
```

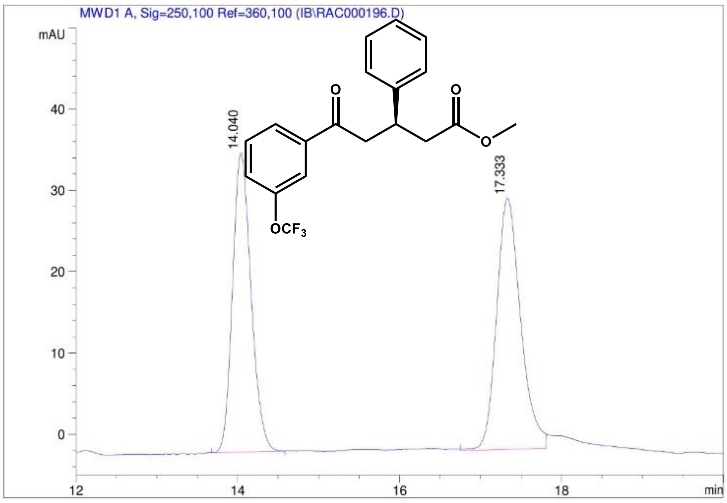

#	Meas. R	Area	Area %
1	14.040	584.129	47.731
2	17.333	639.670	52.269

```
Data File:  C:\CHEM32\1\DATA\IB\CHIR000198.D
Sample Name:  IBD-206-ch
Sample Info:  Phenomenex Lux Cellulose-1, 3 um, 90:10, 1.0 mL/min
```

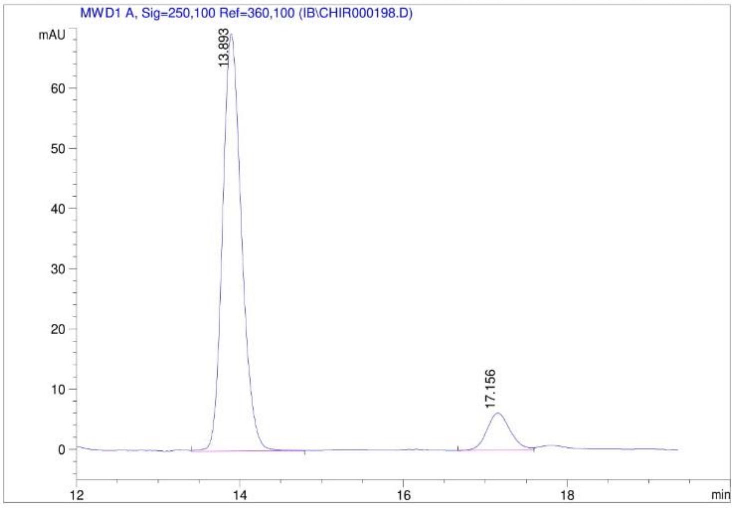

#	Meas. R	Area	Area %
1	13.893	1.111e3	90.136
2	17.156	121.635	9.864

Figure S73. HPLC chromatograms of **4o** (racemic – top, enantioenriched – bottom).

```
Data File:  C:\CHEM32\1\DATA\IB\RAC000031.D
Sample Name:  IBD-188-rac
Sample Info:  Phenomenex Lux Amylose-1, 3 um, 90:10, 1.00 mL/min, p=9
              3bar; T=25st.C
```

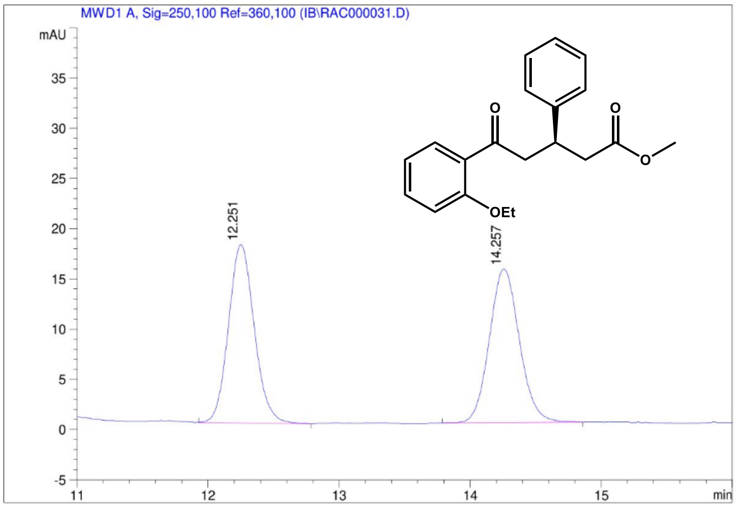

#	Meas. R	Area	Area %
1	12.251	242.714	49.769
2	14.257	244.972	50.231

```
Data File:  C:\CHEM32\1\DATA\IB\RAC000032.D
Sample Name:  IBD-188-chir
Sample Info:  Phenomenex Lux Amylose-1, 3 um, 90:10, 1.00 mL/min, p=9
              3bar; T=25st.C
```

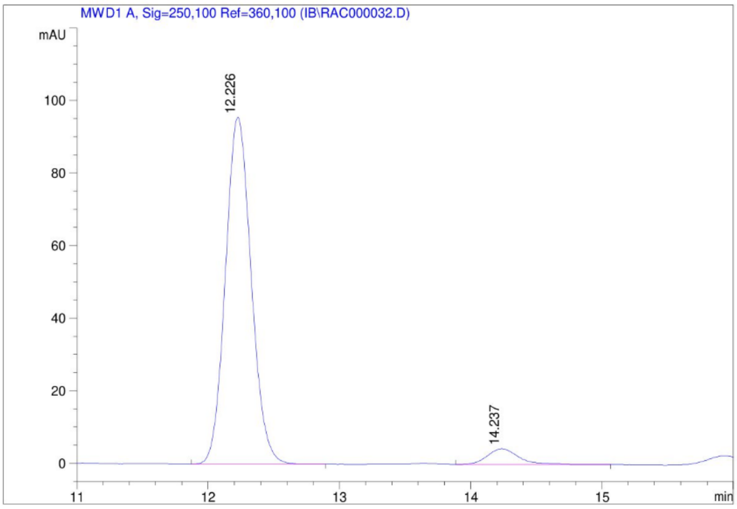

#	Meas. R	Area	Area %
1	12.226	1.304e3	94.698
2	14.237	73.023	5.302

Figure S74. HPLC chromatograms of **4p** (racemic – top, enantioenriched – bottom).

```
Data File:   C:\CHEM32\1\DATA\IB\RAC020296.D
Sample Name:   IBD-179-rac
Sample Info:   Phenomenex Lux Cellulose-1, 3 um, 90:10, 1.00 mL/min, r
               ac, p=105bar; T=25st.C
```

===

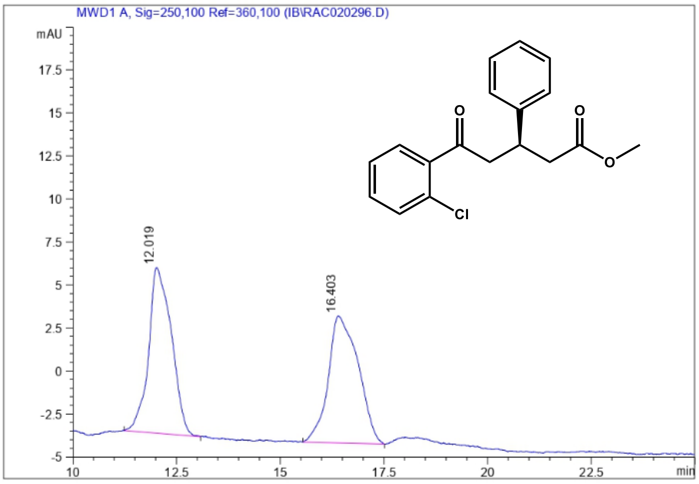

#	Meas. R	Area	Area %
1	12.019	369.487	49.858
2	16.403	371.587	50.142

```
Data File:   C:\CHEM32\1\DATA\IB\CHIR020303.D
Sample Name:   IBD-179-ch
Sample Info:   Phenomenex Lux Cellulose-1, 3 um, 90:10, 1.00 mL/min, r
               ac, p=105bar; T=25st.C
```

===

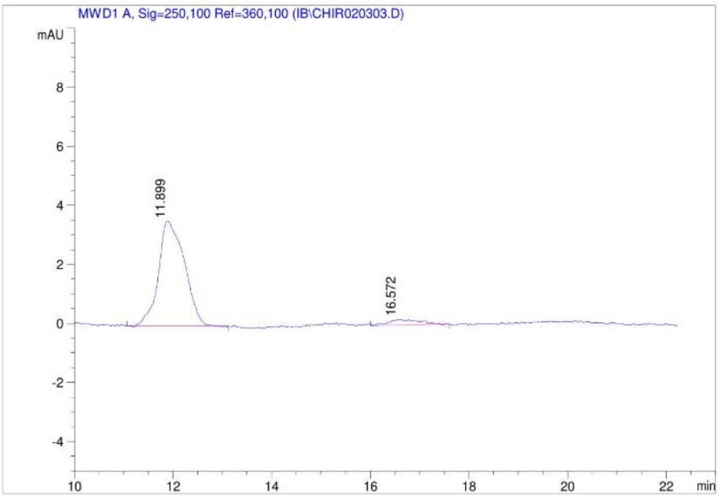

#	Meas. R	Area	Area %
1	11.899	129.389	94.111
2	16.572	8.097	5.889

Figure S75. HPLC chromatograms of **4q** (racemic – top, enantioenriched – bottom).

Phenomenex Lux Amylose 1-3 um, hex 95:5 IPA,0.8 mL/min.254 nm

NUMER PIKU	CZAS RETENCJI [min]	POWIERZCHNIA [µV 1/100 min]	UDZIAŁ % POWIERZCHNI
A/1	15,868	304821	52,589
A/2	18,383	274813	47,411
SUMA		579634	

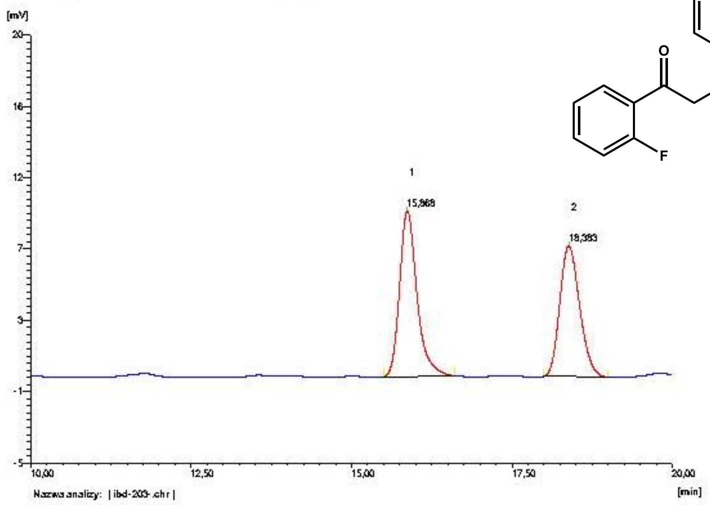

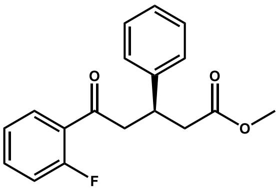

Phenomenex Lux Amylose 1-3 um, hex 95:5 IPA, 0.8 mL/min, 254 nm

NUMER PIKU	CZAS RETENCJI [min]	POWIERZCHNIA [µV 1/100 min]	UDZIAŁ % POWIERZCHNI
A/1	15,967	699336	94,467
A/2	18,510	40960	5,533
SUMA		740296	

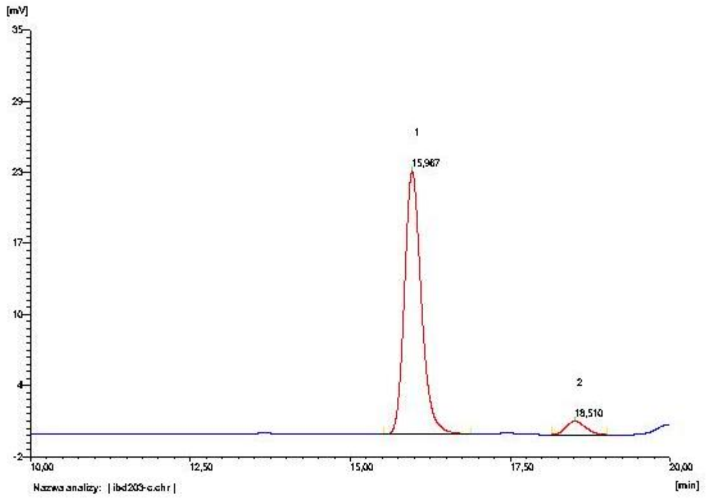

Figure S76. HPLC chromatograms of **4r** (racemic – top, enantioenriched – bottom).

```
Data File:  C:\CHEM32\1\DATA\IB\RAC000497.D
Sample Name:  IBD-246-rac
Sample Info:  Phenomenex Lux Cellulose-1, 3 um, 90:10, 1,0 mL/min
```

->

===

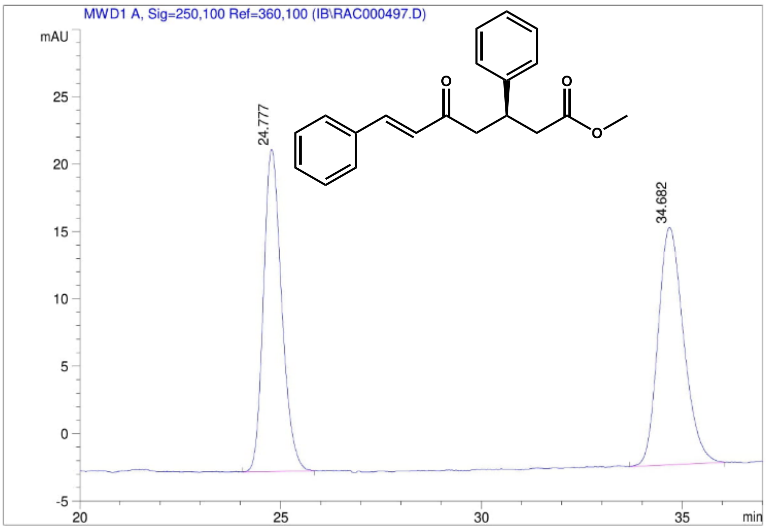

#	Meas. R	Area	Area %
1	24.777	752.620	49.104
2	34.682	780.091	50.896

```
Data File:  C:\CHEM32\1\DATA\IB\CHIR000526.D
Sample Name:  IBD-246-chir
Sample Info:  Phenomenex Lux Cellulose-1, 3 um, 90:10, 1,0 mL/min
```

===

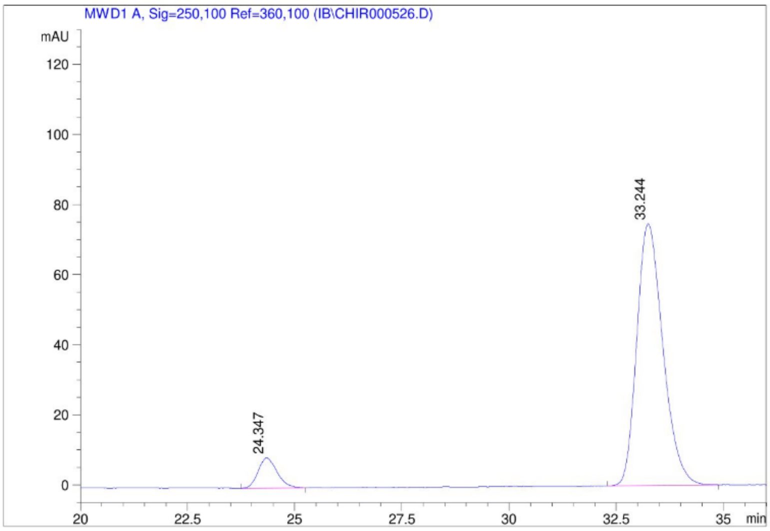

#	Meas. R	Area	Area %
1	24.347	266.487	7.767
2	33.244	3.165e3	92.233

Figure S77. HPLC chromatograms of **4s** (racemic – top, enantioenriched – bottom).

```
Data File:  C:\CHEM32\1\DATA\IB\RAC000266.D
Sample Name:  IBD-218-RAC
Sample Info:  Phenomenex Lux Amylose-1, 3 um, 90:10, 1,0 mL/min
```

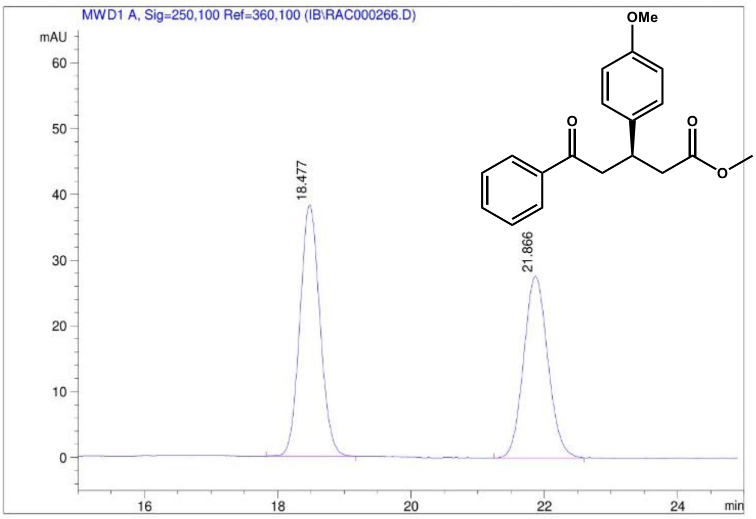

#	Meas. R	Area	Area %
1	18.477	798.564	53.401
2	21.866	696.857	46.599

```
Data File:  C:\CHEM32\1\DATA\IB\CHIR000267.D
Sample Name:  IBD-218-CHIR
Sample Info:  Phenomenex Lux Amylose-1, 3 um, 90:10, 1,0 mL/min
```

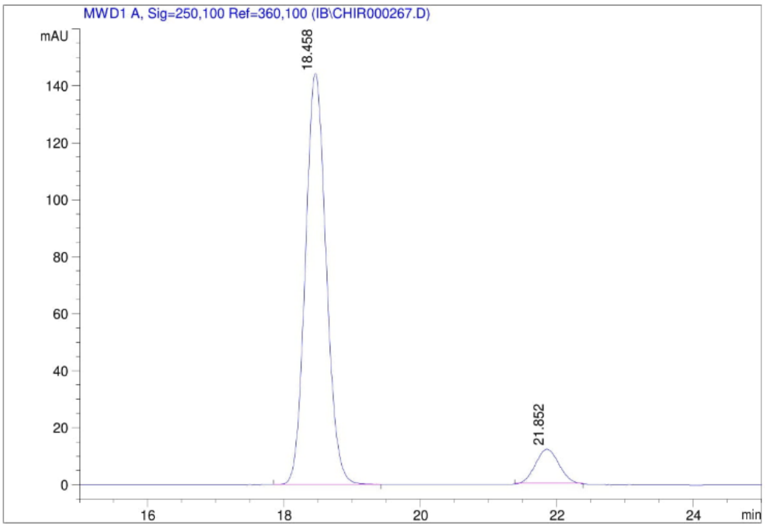

#	Meas. R	Area	Area %
1	18.458	3.049e3	91.592
2	21.852	279.851	8.408

Figure S78. HPLC chromatograms of **4t** (racemic – top, enantioenriched – bottom).

Data File: C:\CHEM32\1\DATA\IB\RAC000259.D
Sample Name: IBD-215-Rac
Sample Info: Phenomenex Lux Amylose-1, 3 um, 90:10, 1,0 mL/min

->

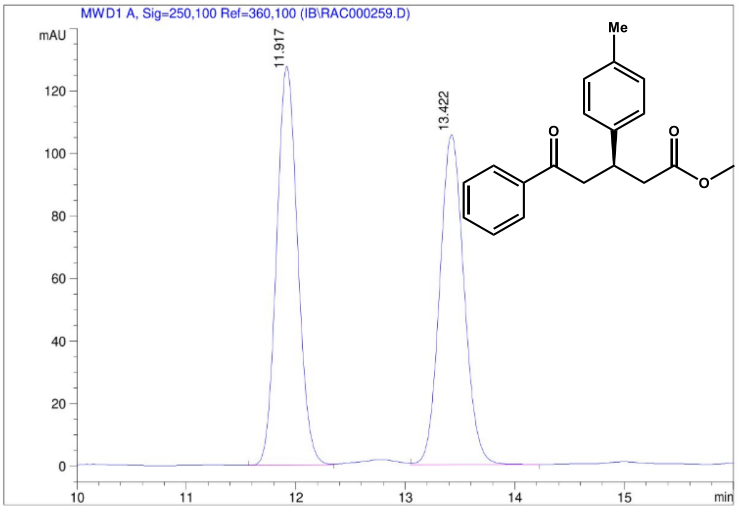

#	Meas. R	Area	Area %
1	11.917	1.705e3	51.131
2	13.422	1.629e3	48.869

Data File: C:\CHEM32\1\DATA\IB\CHIR000261.D
Sample Name: IBD-215-CHIR
Sample Info: Phenomenex Lux Amylose-1, 3 um, 90:10, 1,0 mL/min

->

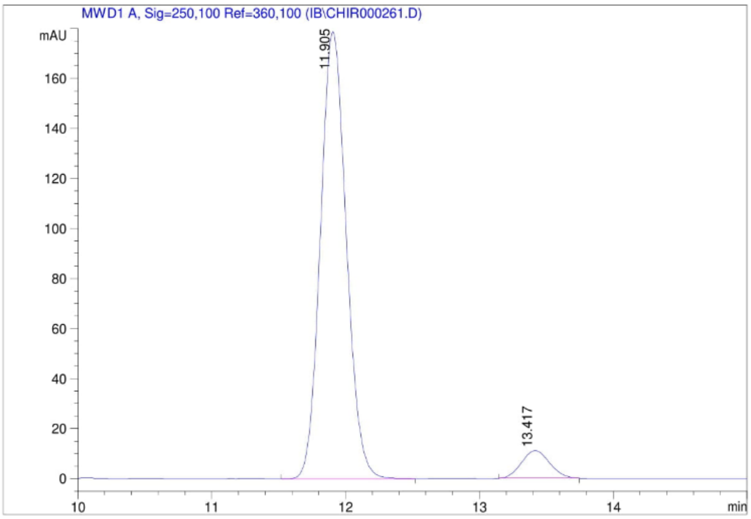

#	Meas. R	Area	Area %
1	11.905	2.400e3	93.872
2	13.417	156.681	6.128

Figure S79. HPLC chromatograms of **4u** (racemic – top, enantioenriched – bottom).

Data File: C:\CHEM32\1\DATA\IB\RAC000333.D
Sample Name: IBD-220-RAC
Sample Info: Phenomenex Lux Amylose-1, 3 um, 90:10, 1.0 mL/min

->

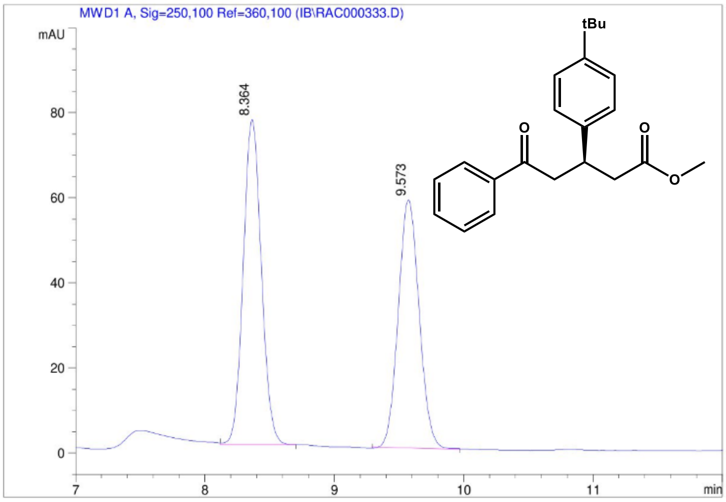

#	Meas. R	Area	Area %
1	8.364	742.423	53.206
2	9.573	652.952	46.794

Data File: C:\CHEM32\1\DATA\IB\RAC000334.D
Sample Name: IBD-220-CHIR
Sample Info: Phenomenex Lux Amylose-1, 3 um, 90:10, 1.0 mL/min

->

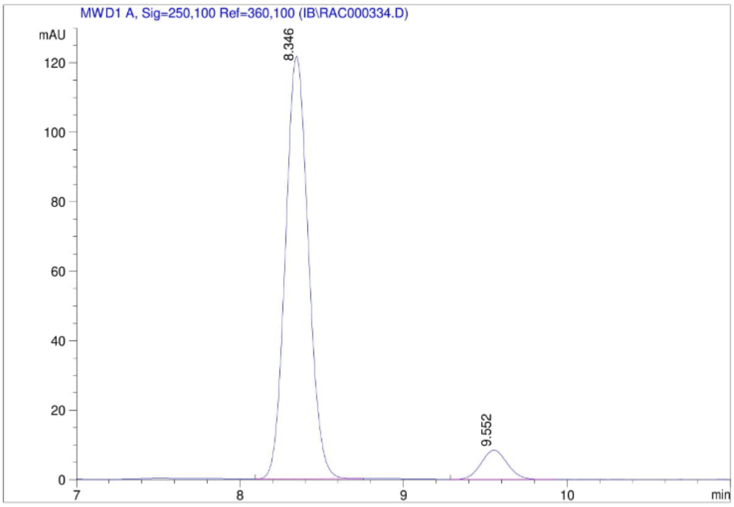

#	Meas. R	Area	Area %
1	8.346	1.184e3	92.670
2	9.552	93.630	7.330

Figure S80. HPLC chromatograms of **4v** (racemic – top, enantioenriched – bottom).

Data File: C:\CHEM32\1\DATA\IB\RAC000290.D
Sample Name: IBD-217-rac
Sample Info: Phenomenex Lux Amylose-1, 3 um, 90-10, 1,0 mL/min

->

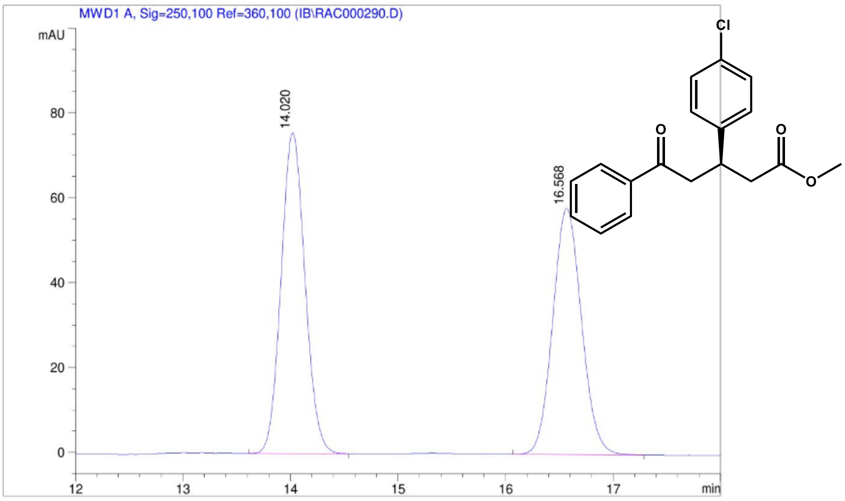

#	Meas. R	Area	Area %
1	14.020	1.168e3	51.949
2	16.568	1.081e3	48.051

Data File: C:\CHEM32\1\DATA\IB\CHIR000291.D
Sample Name: IBD-217-CH
Sample Info: Phenomenex Lux Amylose-1, 3 um, 90-10, 1,0 mL/min

->

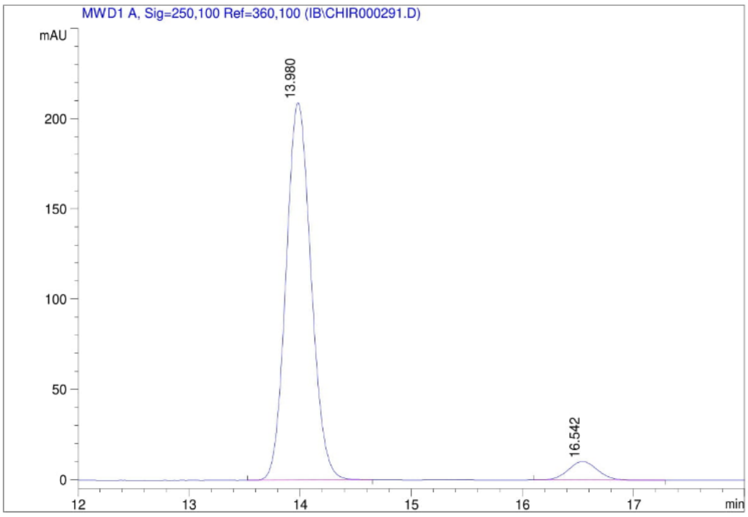

#	Meas. R	Area	Area %
1	13.980	3.265e3	94.549
2	16.542	188.211	5.451

Figure S81. HPLC chromatograms of **4w** (racemic – top, enantioenriched – bottom).

```
Data File:  C:\CHEM32\1\DATA\IB\RAC000206.D
Sample Name:  IBD-214-rac
Sample Info:  Phenomenex Lux Amylose-1, 3 um, 90:10, 1,0 mL/min
```

->

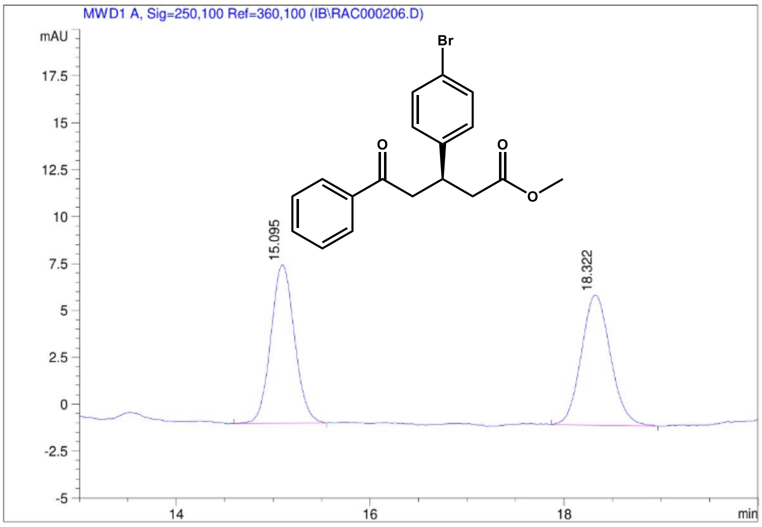

#	Meas. R	Area	Area %
1	15.095	143.571	49.719
2	18.322	145.192	50.281

```
Data File:  C:\CHEM32\1\DATA\IB\CHIR000207.D
Sample Name:  IBD-214-chir
Sample Info:  Phenomenex Lux Amylose-1, 3 um, 90:10, 1,0 mL/min
```

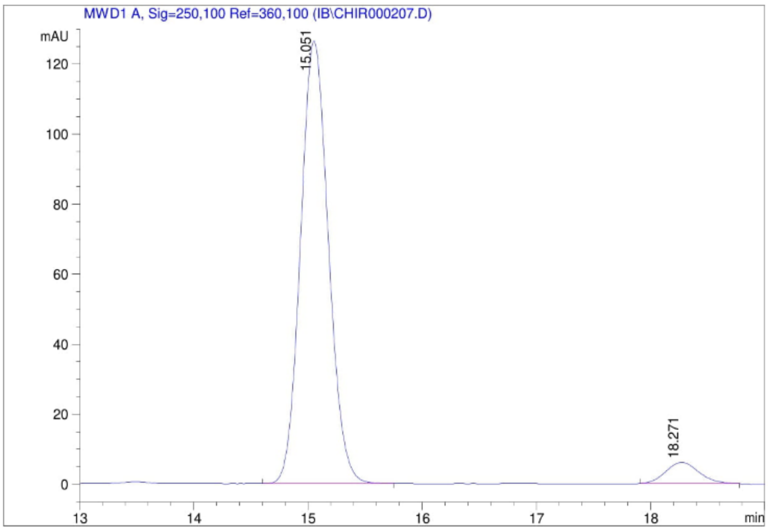

#	Meas. R	Area	Area %
1	15.051	2.144e3	94.693
2	18.271	120.143	5.307

Figure S82. HPLC chromatograms of **4x** (racemic – top, enantioenriched – bottom).

```
Data File:  C:\CHEM32\1\DATA\IB\RAC000321.D
Sample Name:  IBD-219-rac
Sample Info:  Phenomenex Lux Amylose-1, 3 um, 90:10, 1.0 mL/min
```

->

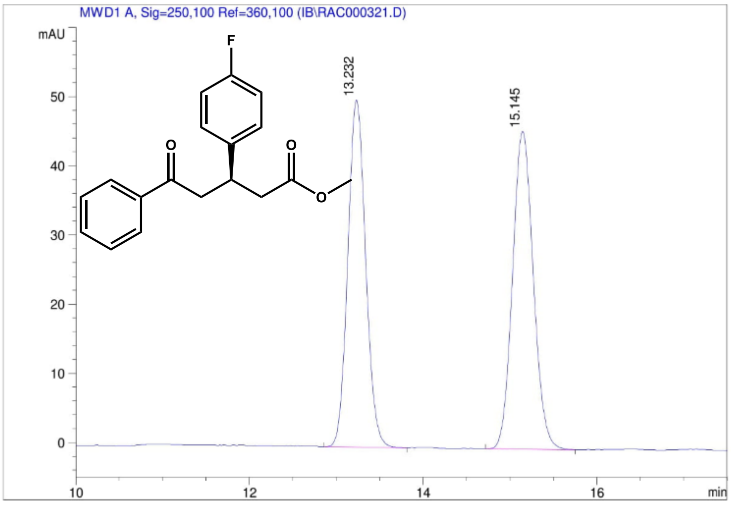

#	Meas. R	Area	Area %
1	13.232	717.462	48.408
2	15.145	764.650	51.592

```
Data File:  C:\CHEM32\1\DATA\IB\CHIR000332.D
Sample Name:  IBD-219-CH
Sample Info:  Phenomenex Lux Amylose-1, 3 um, 90:10, 1.0 mL/min
```

->

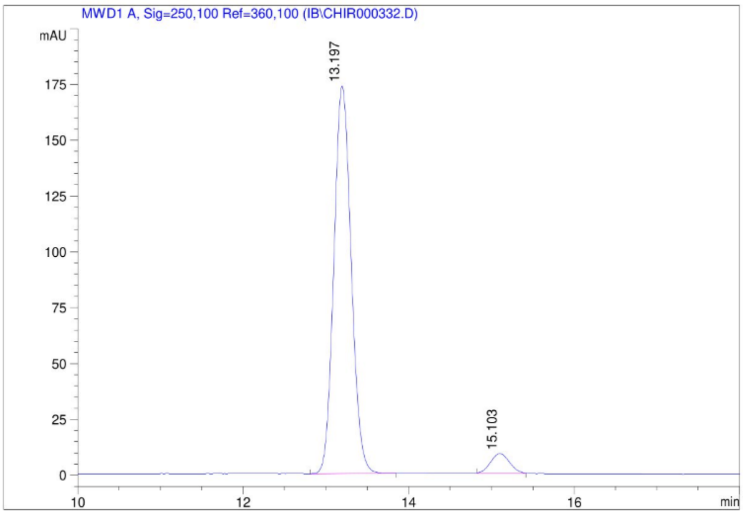

#	Meas. R	Area	Area %
1	13.197	2.518e3	94.530
2	15.103	145.685	5.470

Figure S83. HPLC chromatograms of **4y** (racemic – top, enantioenriched – bottom).

```
Data File:  C:\CHEM32\1\DATA\IB\RAC000369.D
Sample Name:  IBD-233-rac
Sample Info:  Phenomenex Lux Amylose-1, 3 um, 90:10, 1,0 mL/min
```

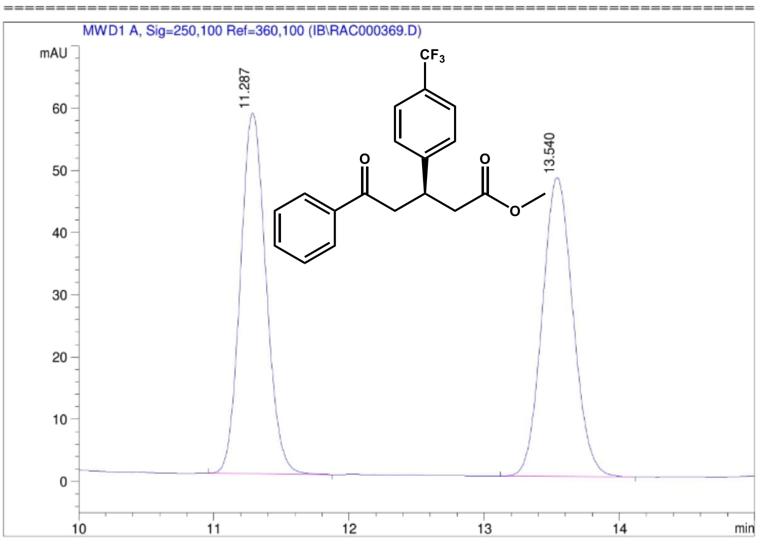

#	Meas. R	Area	Area %
1	11.287	763.723	50.094
2	13.540	760.871	49.906

```
Data File:  C:\CHEM32\1\DATA\IB\CHIR000373.D
Sample Name:  IBD-233-CH
Sample Info:  Phenomenex Lux Amylose-1, 3 um, 90:10, 1,0 mL/min
```

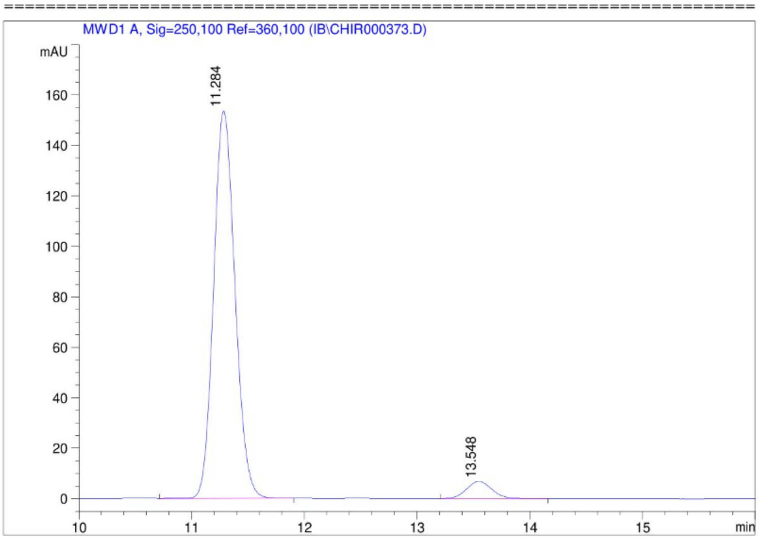

#	Meas. R	Area	Area %
1	11.284	2.025e3	94.972
2	13.548	107.196	5.028

Figure S84. HPLC chromatograms of **4z** (racemic – top, enantioenriched – bottom).

Data File: C:\CHEM32\1\DATA\IB\RAC000418.D
Sample Name: IBD-236-RAC
Sample Info: Phenomenex Lux Cellulose-1, 3 um, 90:10, 1,0 mL/min

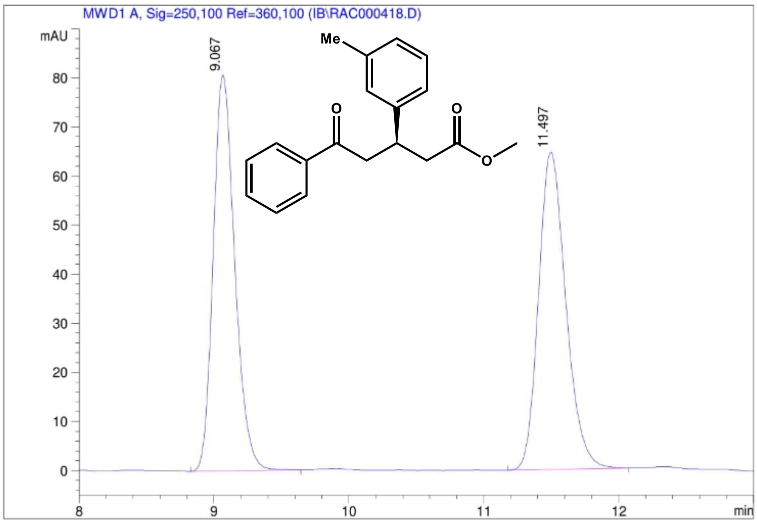

#	Meas. R	Area	Area %
1	9.067	894.112	49.566
2	11.497	909.767	50.434

Data File: C:\CHEM32\1\DATA\IB\RAC000419.D
Sample Name: IBD-236-ch
Sample Info: Phenomenex Lux Cellulose-1, 3 um, 90:10, 1,0 mL/min

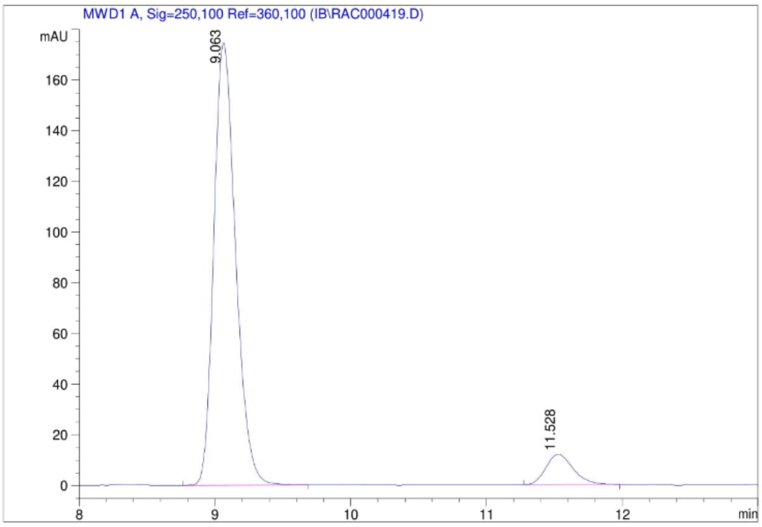

#	Meas. R	Area	Area %
1	9.063	1.949e3	92.170
2	11.528	165.534	7.830

Figure S85. HPLC chromatograms of **4aa** (racemic – top, enantioenriched – bottom).

```
Data File:  C:\CHEM32\1\DATA\IB\RAC000370.D
Sample Name:  IBD-234-rac
Sample Info:  Phenomenex Lux Amylose-1, 3 um, 90:10, 1,0 mL/min
```

->

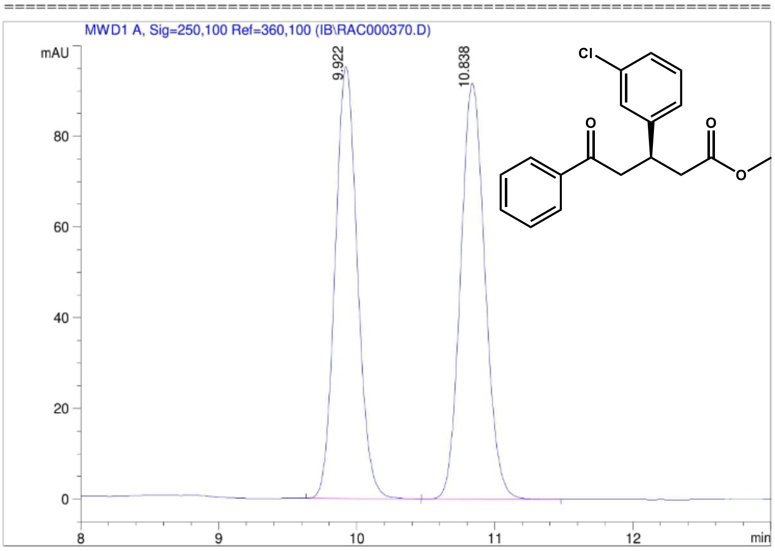

#	Meas. R	Area	Area %
1	9.922	1.069e3	48.685
2	10.838	1.127e3	51.315

```
Data File:  C:\CHEM32\1\DATA\IB\CHIR000371.D
Sample Name:  IBD-234-ch
Sample Info:  Phenomenex Lux Amylose-1, 3 um, 90:10, 1,0 mL/min
```

->

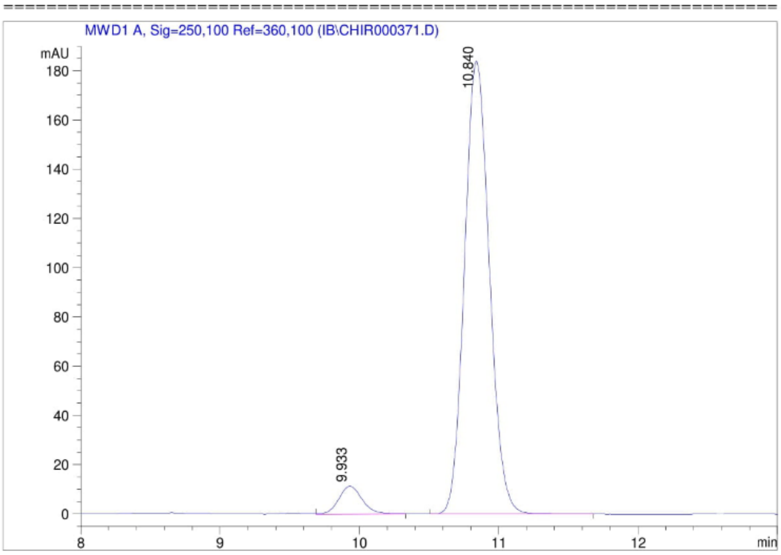

#	Meas. R	Area	Area %
1	9.933	132.770	5.492
2	10.840	2.285e3	94.508

Figure S86. HPLC chromatograms of **4ab** (racemic – top, enantioenriched – bottom).

Data File: C:\CHEM32\1\DATA\IB\RAC000539.D
Sample Name: IBD-248-rac
Sample Info: Phenomenex Lux Cellulose-1, 3 um, 80:20, 1,0 mL/min

->

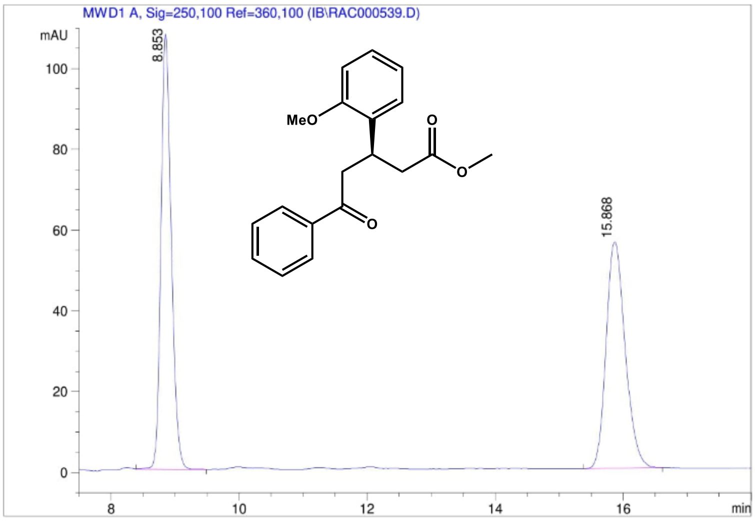

#	Meas. R	Area	Area %
1	8.853	1.209e3	50.984
2	15.868	1.162e3	49.016

Data File: C:\CHEM32\1\DATA\IB\CHIR000540.D
Sample Name: IBD-238-chir
Sample Info: Phenomenex Lux Cellulose-1, 3 um, 80:20, 1,0 mL/min

->

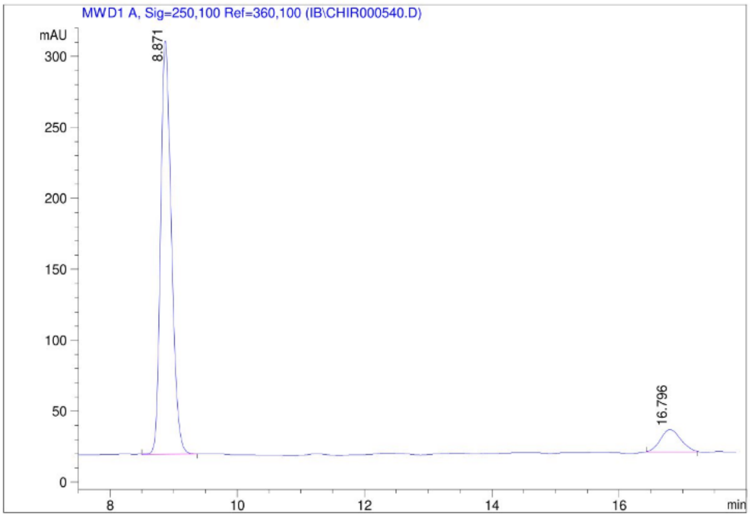

#	Meas. R	Area	Area %
1	8.871	3.426e3	90.668
2	16.796	352.647	9.332

Figure S87. HPLC chromatograms of **4ac** (racemic – top, enantioenriched – bottom).

Data File: C:\CHEM32\1\DATA\IB\RAC000374.D
Sample Name: IBD-235-rac
Sample Info: Phenomenex Lux Amylose-1, 3 um, 90:10, 1,0 mL/min

->

===

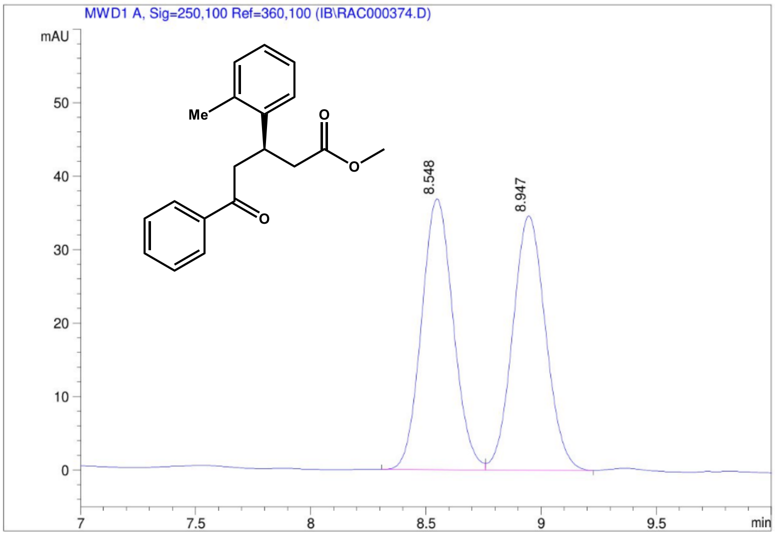

#	Meas. R	Area	Area %
1	8.548	351.952	50.546
2	8.947	344.354	49.454

Data File: C:\CHEM32\1\DATA\IB\CHIR000407.D
Sample Name: IBD-235-CH
Sample Info: Phenomenex Lux Amylose-1, 3 um, 90:10, 1,0 mL/min

->

===

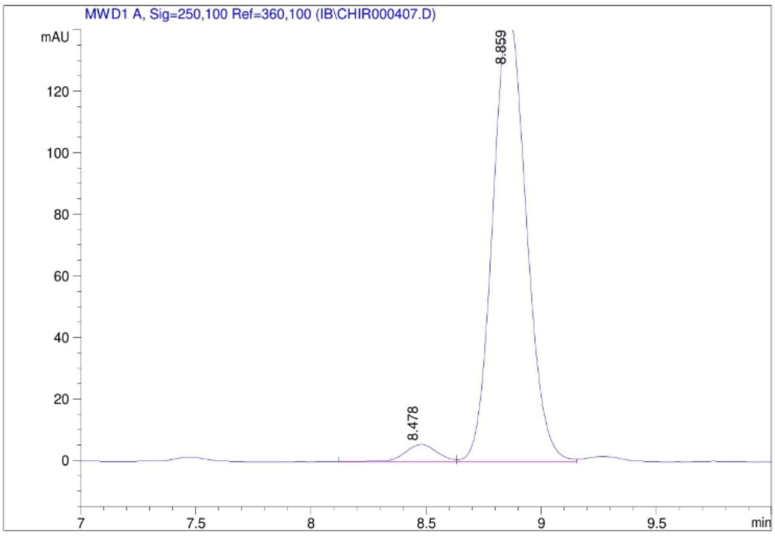

#	Meas. R	Area	Area %
1	8.478	54.142	3.549
2	8.859	1.471e3	96.451

Figure S88. HPLC chromatograms of **4ad** (racemic – top, enantioenriched – bottom).

```
Data File:  C:\CHEM32\1\DATA\IB\RAC000478.D
Sample Name:  IBD-237-rac
Sample Info:  Phenomenex Lux Cellulose-1, 3 um, 90:10, 1,0 mL/min
```

->

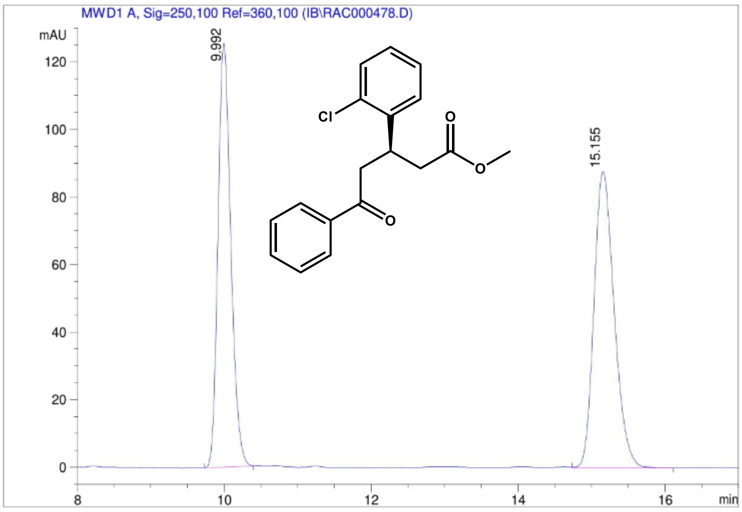

#	Meas. R	Area	Area %
1	9.992	1.521e3	47.467
2	15.155	1.683e3	52.533

```
Data File:  C:\CHEM32\1\DATA\IB\CHIR000479.D
Sample Name:  IBD-237-CHIR
Sample Info:  Phenomenex Lux Cellulose-1, 3 um, 90:10, 1,0 mL/min
```

->

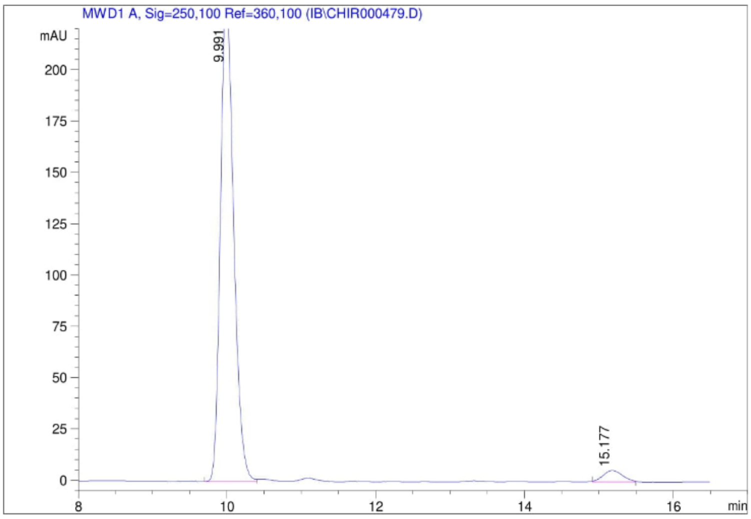

#	Meas. R	Area	Area %
1	9.991	2.912e3	96.510
2	15.177	105.301	3.490

Figure S89. HPLC chromatograms of **4ae** (racemic – top, enantioenriched – bottom).

```
Data File:  C:\CHEM32\1\DATA\IB\RAC000441.D
Sample Name:  IBD-239-rac
Sample Info:  Phenomenex Lux Cellulose-1, 3 um, 90:10, 1,0 mL/min
```

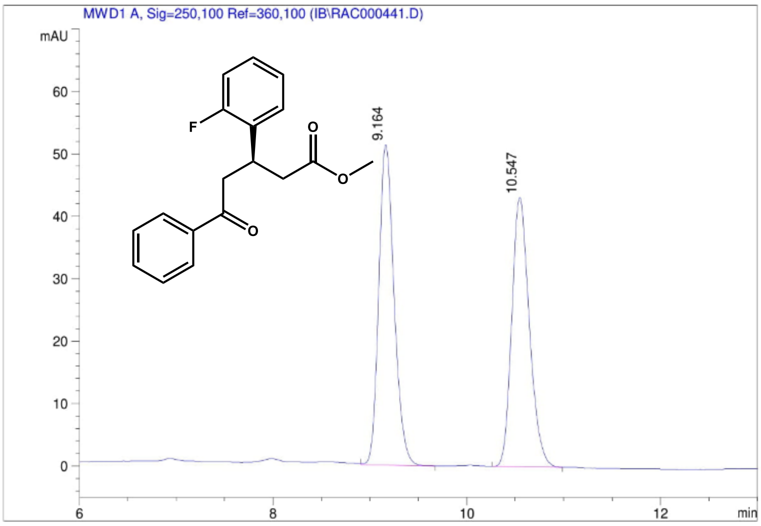

#	Meas. R	Area	Area %
1	9.164	564.087	50.861
2	10.547	544.996	49.139

```
Data File:  C:\CHEM32\1\DATA\IB\CHIR000450.D
Sample Name:  IBD-239-chir
Sample Info:  Phenomenex Lux Cellulose-1, 3 um, 90:10, 1,0 mL/min
```

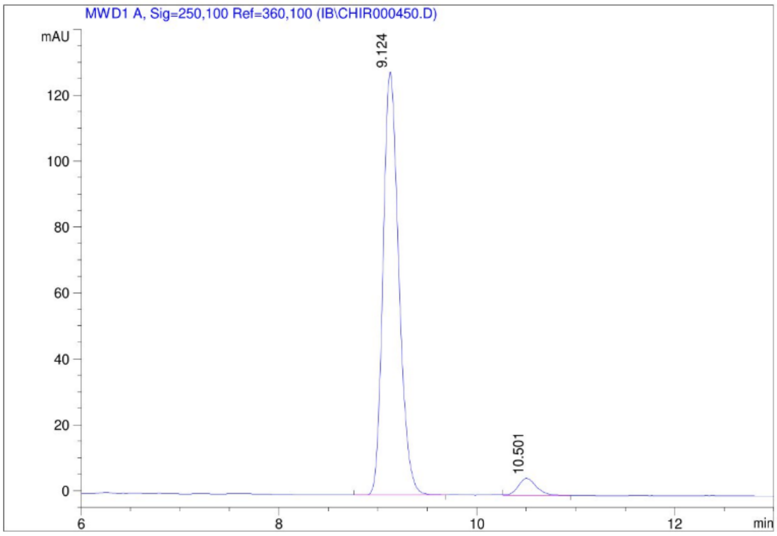

#	Meas. R	Area	Area %
1	9.124	1.421e3	95.612
2	10.501	65.201	4.388

Figure S90. HPLC chromatograms of **4af** (racemic – top, enantioenriched – bottom).

Data File: C:\CHEM32\1\DATA\IB\RAC000455.D
Sample Name: IBD-240-rac
Sample Info: Phenomenex Lux Cellulose-1, 3 um, 90:10, 1,0 mL/min

->

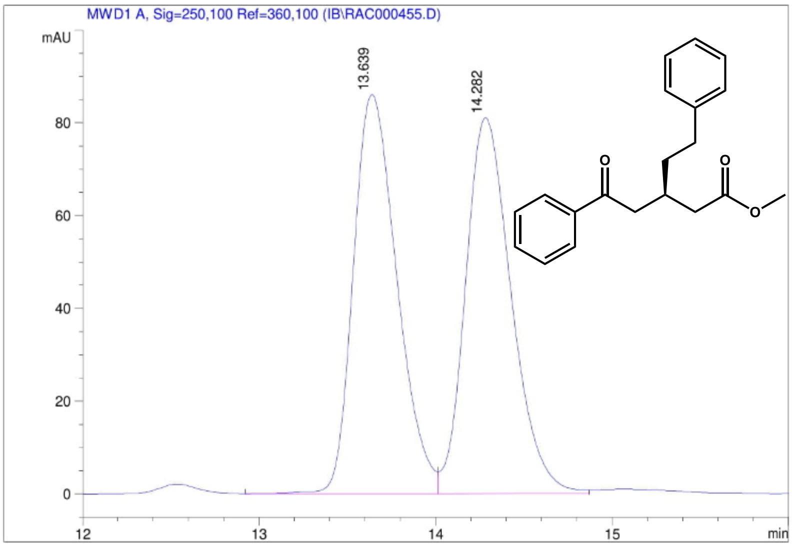

#	Meas. R	Area	Area %
1	13.639	1.478e3	50.272
2	14.282	1.462e3	49.728

Data File: C:\CHEM32\1\DATA\IB\CHIR000456.D
Sample Name: IBD-240-chir
Sample Info: Phenomenex Lux Cellulose-1, 3 um, 95:5, 1,0 mL/min

->

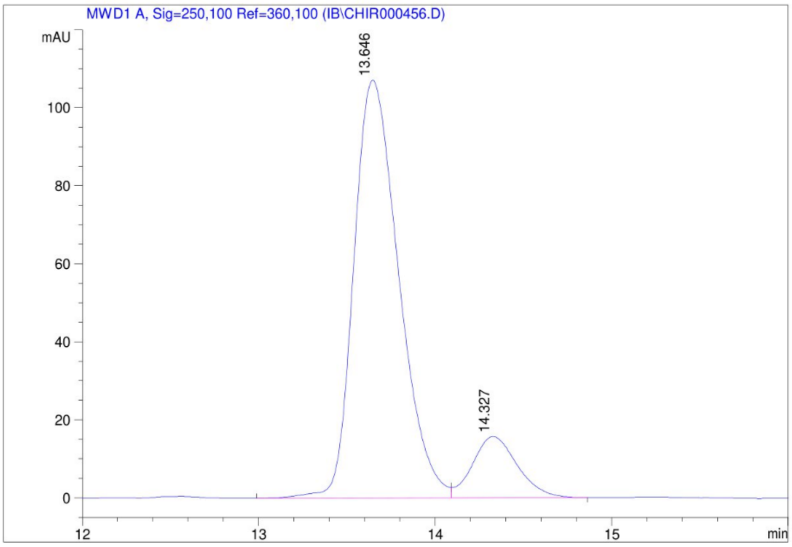

#	Meas. R	Area	Area %
1	13.646	1.897e3	87.266
2	14.327	276.884	12.734

Figure S91. HPLC chromatograms of **4ag** (racemic – top, enantioenriched – bottom).

Data File: C:\CHEM32\1\DATA\IB\RAC010681.D
Sample Name: IBD-547-Rac
Sample Info: Phenomenex Lux Cellulose-1, 3 um, 95:5, 0.50 mL/min, p= 54bar; T=25st.C

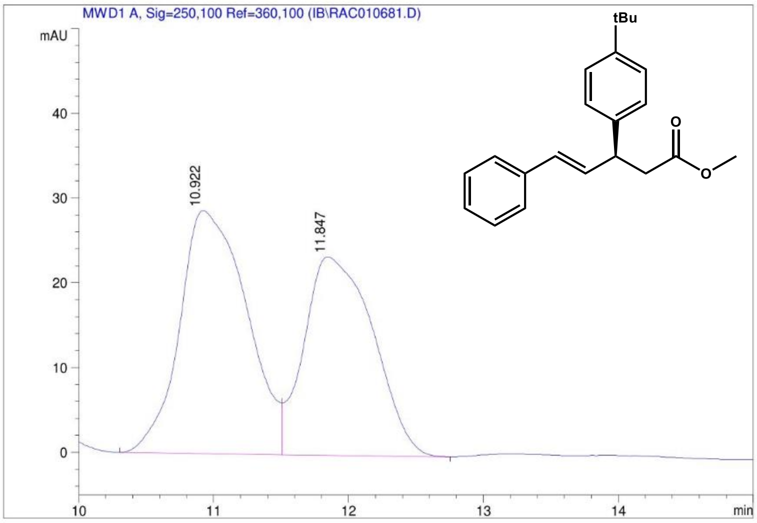

#	Meas. R	Area	Area %
1	10.922	992.662	54.296
2	11.847	835.569	45.704

Data File: C:\CHEM32\1\DATA\IB\CHIR010682.D
Sample Name: IBD-541
Sample Info: Phenomenex Lux Cellulose-1, 3 um, 95:5, 0.50 mL/min, p= 54bar; T=25st.C

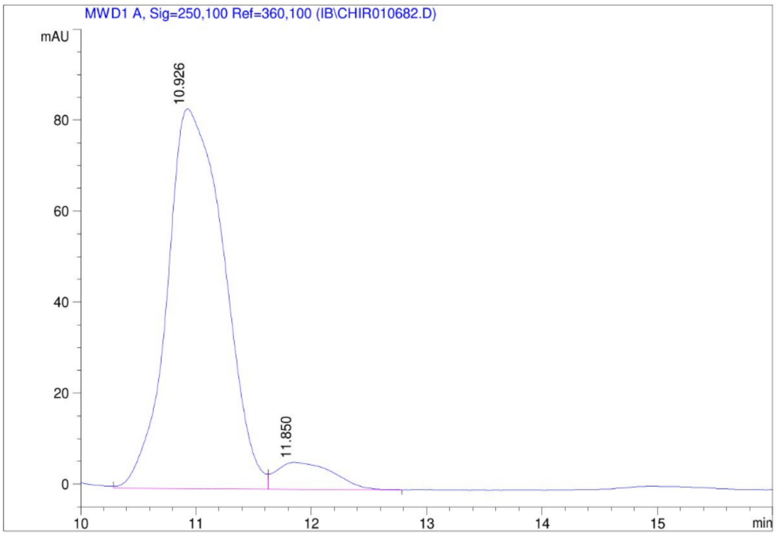

#	Meas. R	Area	Area %
1	10.926	2.862e3	93.411
2	11.850	201.846	6.589

Figure S92. HPLC chromatograms of **5b** (racemic – top, enantioenriched – bottom)

7. References

[1] K. Dzieszkowski, M. Słotwiński, K. Rafińska, T.M. Muzioł, Z. Rafiński; NHC-catalyzed enantioselective C2-functionalization of 3-hydroxychromenones via α,β-unsaturated acyl azoliums *Chem. Commun.* **2021**, 57, 9999.

[2] Z. Wang, T. Song, Y. Feng, Z. Guo, Y. Fan, W. Xu, L. Liu, A. Wang, Z. Zhang; Bcl-2/MDM2 Dual Inhibitors Based on Universal Pyramid-Like α-Helical Mimetics *J. Med. Chem.* **2016**, 59, 7, 3152–3162.

[3] Current Patent Assignee: IRM LLC - WO2006/84176, 2006, A2; Location in patent: Page/Page column 25-26.

[4] M. J. Drysdale, S. L. Hind, M. Jansen, J. F. Reinhard, Jr. Synthesis and SAR of 4-Aryl-2-hydroxy-4-oxobut-2-enoic Acids and Esters and 2-Amino-4-aryl-4-oxobut-2-enoic Acids and Esters: Potent Inhibitors of Kynurenine-3-hydroxylase as Potential Neuroprotective Agents *J. Med. Chem.* **2000**, 43, 123-127.

[5] X.-H. Jiang, L.-D. Song, Y.-Q. Long Highly Efficient Preparation of Aryl β-Diketo Acids with tert-Butyl Methyl Oxalate *J. Org. Chem.* **2003**, 68, 19, 7555–7558.

[6] Current Patent Assignee: VETTORE - US2018/162822, 2018, A1; Location in patent: Paragraph 0540; 0541

[7] T. R. K. Reddy, C. Li, X. Guo, H. K. Myrvang, P. M. Fischer, L. V. Dekker; Design, Synthesis, and Structure−Activity Relationship Exploration of 1-Substituted 4-Aroyl-3-hydroxy-5-phenyl-1H-pyrrol-2(5H)-one Analogues as Inhibitors of the Annexin A2−S100A10 Protein Interaction *J. Med. Chem.* **2011**, 54, 7, 2080–2094.

[8] L.N. Kurkovskaya, N.N. Shapet'ko, Y.S. Andreichikov *et al.*; *J. Struct Chem* **1973** *13, 958-963.*

[9] Rigaku Oxford Diffraction. CrysAlisPro Software System, version 1.171.38.41; Rigaku Corporation: Oxford, UK 2015.

[10] G.M. Sheldrick, *Acta Crystallogr.* **2008**, A64, 112–122.

[11] G.M. Sheldrick, *Acta Crystallogr.* **2015**, C71, 3–8.

[12] C. F. Macrae, I. J. Bruno, J. A. Chisholm, P. R. Edgington, P. McCabe, E. Pidcock, L. Rodriguez-Monge, R. Taylor, J. Van De Streek, P. A. Wood, *J. Appl. Cryst.* **2008**, 41, 466–470.

[13] http://www.povray.org/.
